# An integrated screening system for the selection of exemplary substrates for natural and engineered cytochrome P450s

**DOI:** 10.1038/s41598-019-54473-8

**Published:** 2019-12-02

**Authors:** Naoki Kanoh, Ayano Kawamata-Asano, Kana Suzuki, Yusuke Takahashi, Takeshi Miyazawa, Takemichi Nakamura, Takashi Moriya, Hiroyuki Hirano, Hiroyuki Osada, Yoshiharu Iwabuchi, Shunji Takahashi

**Affiliations:** 1 Graduate School of Pharmaceutical Sciences, Tohoku University, 6-3 Aza-Aoba, Aramaki, Aoba-ku, Sendai 980-8578 Japan; 2 Institute of Medicinal Chemistry, Hoshi University, 2-4-1 Ebara, Shinagawa-ku, Tokyo 142-8501 Japan; 3 Chemical Biology Research Group, RIKEN Center for Sustainable Resource Science, 2-1 Hirosawa, Wako, Saitama 351-0198 Japan; 4 Molecular Structure Characterization Unit, Technology Platform Division, RIKEN Center for Sustainable Resource Science, 2-1 Hirosawa, Wako, Saitama 351-0198 Japan; 5 Chemical Resource Development Research Unit, Technology Platform Division, RIKEN Center for Sustainable Resource Science, 2-1 Hirosawa, Wako, Saitama 351-0198 Japan; 6 Natural Product Biosynthesis Research Unit, RIKEN Center for Sustainable Resource Science, 2-1 Hirosawa, Wako, Saitama 351-0198 Japan

**Keywords:** Biocatalysis, Biocatalysis

## Abstract

Information about substrate and product selectivity is critical for understanding the function of cytochrome P450 monooxygenases. In addition, comprehensive understanding of changes in substrate selectivity of P450 upon amino acid mutation would enable the design and creation of engineered P450s with desired selectivities. Therefore, systematic methods for obtaining such information are required. Herein, we developed an integrated P450 substrate screening system for the selection of “exemplary” substrates for a P450 of interest. The established screening system accurately selected the known exemplary substrates and also identified previously unknown exemplary substrates for microbial-derived P450s from a library containing sp^3^-rich synthetic small molecules. Synthetically potent transformations were also found by analyzing the reactions and oxidation products. The screening system was applied to analyze the substrate selectivity of the P450 BM3 mutants F87A and F87A/A330W, which acquired an ability to hydroxylate non-natural substrate steroids regio- and stereoselectively by two amino acid mutations. The distinct transition of exemplary substrates due to each single amino acid mutation was revealed, demonstrating the utility of the established system.

## Introduction

Cytochrome P450s (P450s) constitute an exceptional superfamily of monooxygenases found in living organisms from microorganisms to plants to humans, and are responsible for drug metabolism and biosynthesis of secondary metabolites^1^. To date, a growing number of P450 genes have been identified across many biological species, with nearly 500,000 entries in GenBank ascribed to P450s as of early 2018. A large amount of data on substrate selectivity has been experimentally acquired for human P450s^2^ and some particular microbial P450s^3^. Such experimental data and associated computations have been used to develop a number of drug metabolism prediction programs for human xenobiotic P450s^4^. On the other hand, many “orphan” P450s for which the natural substrate(s) and function(s) are unknown have also been reported^5,6^. For example, about a quarter of the human P450s (13 of 57) are considered to be orphans^7^, and only 2.4% of the more than 7,500 streptomycete P450s have been functionally characterized^8^.

Development of P450 mutants that oxidize unnatural substrates and produce useful chemicals has attracted much attention recently^3,9,10^. Some P450s and their mutants are being utilized^11–13^ or developed^14^ as practical catalysts for the oxidative transformation of particular useful small molecules. Among the P450s, P450 BM3 (CYP102A1) from *Bacillus megaterium*^15,16^, which catalyzes the oxidation of long-chain fatty acids at the ω-1, ω-2, and ω-3 positions, is a well-studied starting point for these efforts^3^. P450 BM3 is a fusion protein in which a P450 heme domain and a diflavin NADPH-P450 reductase domain are covalently linked to produce a highly efficient electron transport system. High activity of P450 BM3 and its good functional expression in *Escherichia coli* have made it an attractive platform for engineering aimed at the selective oxidation of unnatural substrates and the production of useful compounds^17^. Several guidelines for selecting the point of mutation have been developed to achieve the desired transformation and selectivity^17,18^. However, generation of the active mutants mainly relies on random- or saturation mutagenesis, since reliable knowledge of how substrate selectivity changes upon amino acid mutagenesis is still lacking. Designing the function of P450s would be possible if sufficient data on the influence of amino acid mutations in the substrate-binding pocket on substrate selectivity were accumulated for the P450 of interest. In any case, a method capable of screening substrates for a P450 of interest from a compound library would be of use for obtaining such data, and thus is strongly needed. However, the criteria that must be met for compounds to be substrates for a P450 of interest are elusive and highly dependent on the nature of P450 species. Thus, if common criteria were set and substrates that satisfy the criteria were obtained, such “exemplary” substrates will be not only good references for comparing P450’s activity but also good starting points for designing the function of P450s having desired activity.

Herein, by combining the four established methods, we constructed an integrated P450 substrate screening system for the selection of exemplary substrates that (1) are typically recognized by the P450 enzyme, (2) induce type I spectral change, (3) are rapidly oxidized in a highly coupled manner, and (4) are converted specifically to a limited number of their oxidized products. The effectiveness of the screening system was demonstrated by the discovery of new exemplary substrates for microbial P450s from an in-house sp^3^-rich small molecule library. The established system was utilized to explore the substrate selectivity of P450 BM3 (F87A) and P450 BM3 (F87A/A330W) mutants, both of which were shown to have the ability to hydroxylate steroids in a regio- and stereoselective manner^19^. Although roles of many active site amino acids are well understood and transition in substrate selectivity of some P450 BM3 mutants are known^3,20,21^, comprehensive analysis of the transition of exemplary substrates for P450 mutants has not been reported. In the present study, comparison of exemplary substrates for P450 BM3 wild-type (WT), P450 BM3 (F87A), and P450 BM3 (F87A/A330W) was performed, demonstrating a distinct transition of exemplary substrates due to each single amino acid mutation.

## Results

### Construction of an integrated P450 substrate screening system

**Figure 1:**
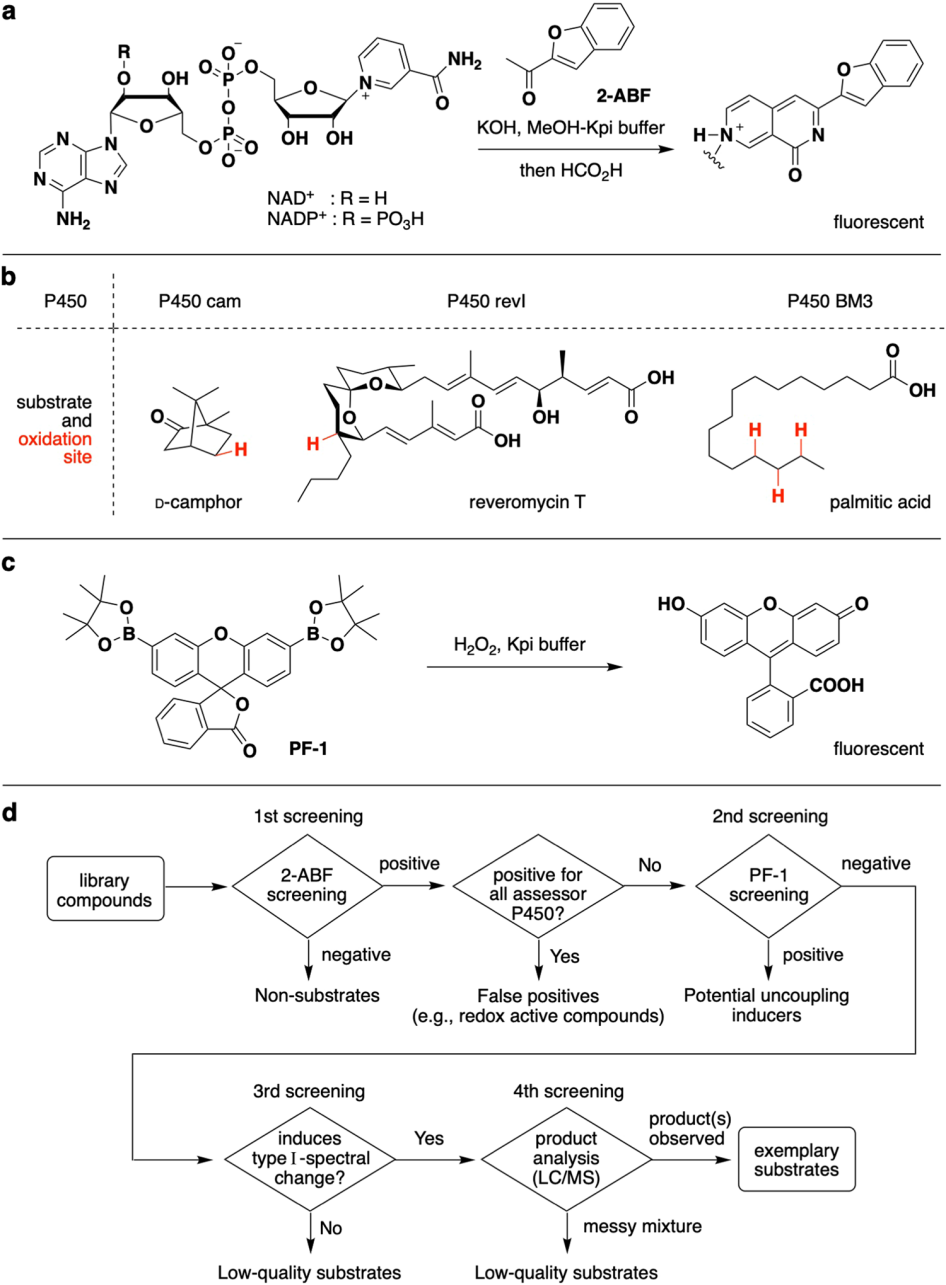
Summary of the integrated P450 substrate screening system. (**a**) The amount of NAD(P)^+^ generated during P450-mediated substrate oxidation is quantified by an addition of 2-acetylbenzofuran (2-ABF), which affords fluorescent 2,7-naphtyridinone. (**b**) Assessor P450s and their substrates. The oxidation site on each substrate is shown in red. (**c**) H_2_O_2_ generated by an uncoupling reaction is detected by using a fluorescent H_2_O_2_ probe peroxyfluor-1 (PF-1). (**d**) Flowchart diagram of the integrated P450 substrate screening system. The screening system consists of a four-step selection procedure.

To date, several methods for screening potential substrates for a P450 of interest have been developed^22,23^ based on (1) the electron shuttling mechanism from nicotinamide adenine dinucleotide (NADH) or nicotinamide adenine dinucleotide phosphate (NADPH) to P450 and (2) the P450 catalytic substrate oxidation cycle. These include methods for quantifying O_2_ consumption^24^, NAD(P)H consumption^25,26^, and NAD(P)^+^ production^27^, and methods for detecting oxidized products^28,29^. Among them, the detection/quantification of NAD(P)^+^ has been thought to be an acceptable method for a high-throughput primary screen of P450 substrates^23^ because it requires only the detection of NAD(P)^+^ by using inexpensive chemical reagents such as methyl ketones^30,31^ and hydroxide^32,33^ regardless of the substrate type. Previously, therefore, we sought sensitive NAD(P)^+^ detection reagents and platforms^34^ to be used as high-throughput P450 substrate screens, and found that 2-acetylbenzofuran (2-ABF), which generates fluorescent 2,7-naphthyridinone derivatives upon reaction with NAD(P)^+^, is a more sensitive, efficient, and inexpensive NAD(P)^+^ detection reagent compared with the pre-existing reagent (Fig. 1a)^35^. 2-ABF reacts with NAD^+^ 20 times faster than acetophenone, the standard reagent, and detects NAD^+^ with 1000-fold greater sensitivity^35^. The NAD(P)^+^ detection method using 2-ABF, termed the 2-ABF method, is thought to be well-suited for microtiter plate-based screening of P450 substrates.

However, a major issue of the NAD(P)^+^ measuring method yet to be addressed is the high incidence of false positives, a practical disadvantage that was pointed out earlier^23^. We conjectured that there are two major sources of this problem. The first is the redox-positive nature inherent to some compounds and their degradation products. Compounds that oxidize NAD(P)H directly or via electron transfer proteins generate NAD(P)^+^ without involving P450. Such compounds tend to yield positive results regardless of the P450 tested. Thus, we planned to eliminate these sorts of false positives by applying the 2-ABF method to several types of P450s whose substrate(s) are structurally different. Toward this end, we chose the following P450s as “assessor” P450s (Fig. 1b): (1) P450 cam^36^ (a.k.a., CYP101) from *Pseudomonas putida*, whose natural substrate is a cyclic terpene D-camphor, (2) P450 revI^37^ from *Streptomyces* sp. SN-593, whose natural substrate is reveromycin T, which is responsible for biosynthesis of the antibiotic polyketide reveromycin A^38^, and (3) P450 BM3. Compounds found to be positive for all three assessor P450s would be regarded as false positives and thus eliminated from the hit compounds (see the Experimental section for details).

The second potential source of the false positives is the presence of uncoupling reactions. Certain types of small molecules are recognized, but not oxidized by a particular P450. Instead, molecular oxygen and electrons from NAD(P)H are consumed to generate superoxide anion radical (.O_2_^−^), hydrogen peroxide (H_2_O_2_) or water^39^. Among them, short-lived superoxide radical (t_1/2_ = 10^−5^ s)^40^ is known to be readily converted to hydrogen peroxide^41^, but quantification of the water generated by uncoupling pathways is difficult because of its abundance. Thus, to eliminate these uncoupling inducers, we decided to utilize a H_2_O_2_ detection system as a second screen. Among H_2_O_2_ detecting reagents, we chose Peroxyfluor-1 (PF-1; Fig. 1c), which was originally developed by Chang *et al*. as an imaging agent for cellular hydrogen peroxide^42^. Although high concentrations of P450 and electron transfer proteins affected the detection of H_2_O_2_, we succeeded in minimizing this negative influence by optimizing the concentration of each component (Supporting Fig. 1). The sensitivity and the fluorogenic character were found to be suitable for microtiter plate-based screening (Supporting Fig. 2).

Thus, the high-throughput procedure to screen substrate candidates for P450s of interest is as follows (Fig. 1d). Each test compound is first incubated with a P450 reaction mixture (the P450 of interest, NAD(P)H, and redox partners (if necessary) in Kpi buffer), then 2-ABF is added, and the resultant fluorescence is recorded to screen compounds that promote the generation of NAD(P)^+^ (first screen). Among the hit compounds, those found to be positive for all assessor P450s (P450 cam, P450 BM3, and P450 revI) are eliminated as false positives. Then, each of the resulting first hit compounds are incubated with the P450 reaction mixture in the presence of PF-1 to examine whether the compounds promote H_2_O_2_ generation (i.e., uncoupling) during the incubation with P450. Positives for the PF-1 assay are eliminated as potential uncoupling inducers (second screen). Both the first and second screens can be run using 96- or 384-well microtiter plates. Then, each selected substrate candidate is tested to see whether it induces a type I spectral change^43,44^ as a substrate for the P450 of interest (third screen). The third hit compounds are individually reacted with the P450, the products are analyzed by LC/MS, and compounds efficiently converted to a single or several products are finally selected as exemplary substrates for the particular P450. It should be noted that compounds that degraded by multiple oxidations at multiple sites are eliminated at the fourth screen. Such compounds are not exemplary compounds in our definition because they are no use for further applications.

### Selection of an sp^3^-rich small molecule library for P450 substrate screening

Selection of a small molecule library having an adequate chemical space and compound diversity is another key feature of the successful screening of P450 substrates. We are particularly interested in microbial P450s and their mutants that participate (or would participate) in the production of sp^3^-rich secondary metabolites (or useful chiral building blocks) and have a high substrate selectivity. Although there are many sources of library compounds, including academic institutions and commercial vendors, small molecule libraries suitable for the screening of P450 substrates should differ from drug candidate libraries that mainly consist of sp^2^-rich relatively flat molecules.

We herein decided to utilize an in-house small molecule library mainly consisting of synthetic intermediates generated during the synthesis of natural products and organocatalysts. We have been involved in the design of organocatalysts^45^ and the total synthesis of bioactive secondary metabolites, including terpenes^46,47^, alkaloids^48,49^, polyketides^50–52^, and others^53,54^. During these synthetic studies, large numbers of intermediates, derivatives, and model compounds were prepared, and therefore many of them are stocked at our laboratories. The target natural products and organocatalysts are usually sp^3^-rich compounds, and thus their synthetic intermediates should possess the same sp^3^-rich character. Moreover, many of these synthetic intermediates contain structural motifs of secondary metabolites that would be oxidized by P450 enzymes.

**Figure 2:**
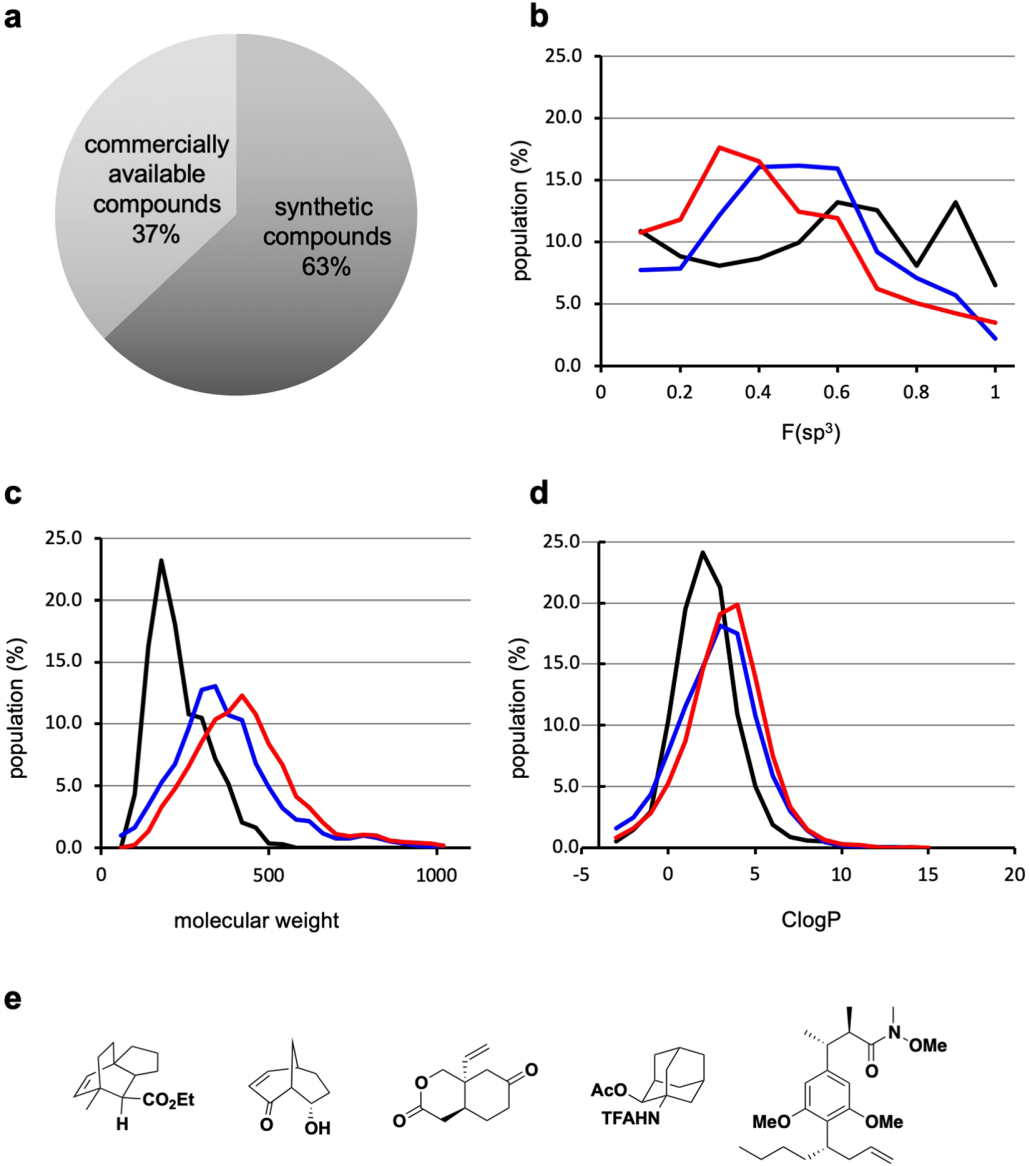
Summary of the in-house pilot library. (**a**) Breakdown of the total 1,047 compounds, (**b**–**d**) Comparison of the in-house pilot library (black line) with the drug bank library (total 8,855 compounds, blue line) and a library from a commercial vendor (total 1,738 compounds, red line) in terms of the (**b**) F(sp^3^) value, (**c**) molecular weight and (**d**) ClogP value. (e) Example of library compounds in the in-house pilot library. TFA: trifluoroacetyl.

Consequently, we constructed a pilot library consisting of 1,047 compounds, two-thirds of which are synthetic compounds, as described above (Fig. 2a; the structures of all compounds are shown in Supporting Fig. 3). The values of the distribution of molecular weight, ClogP, and Fsp^3^ (the fraction of sp^3^-carbon)^55^ from our in-house library, and their comparison with the corresponding values from the drug bank (https://www.drugbank.ca) and a representative commercial vendor are summarized in Fig. 2b–d. These data indicated that our library consisted of relatively small and sp^3^-rich molecules (Fig. 2e).

### Evaluation of the integrated P450 substrate screening system

**Figure 3:**
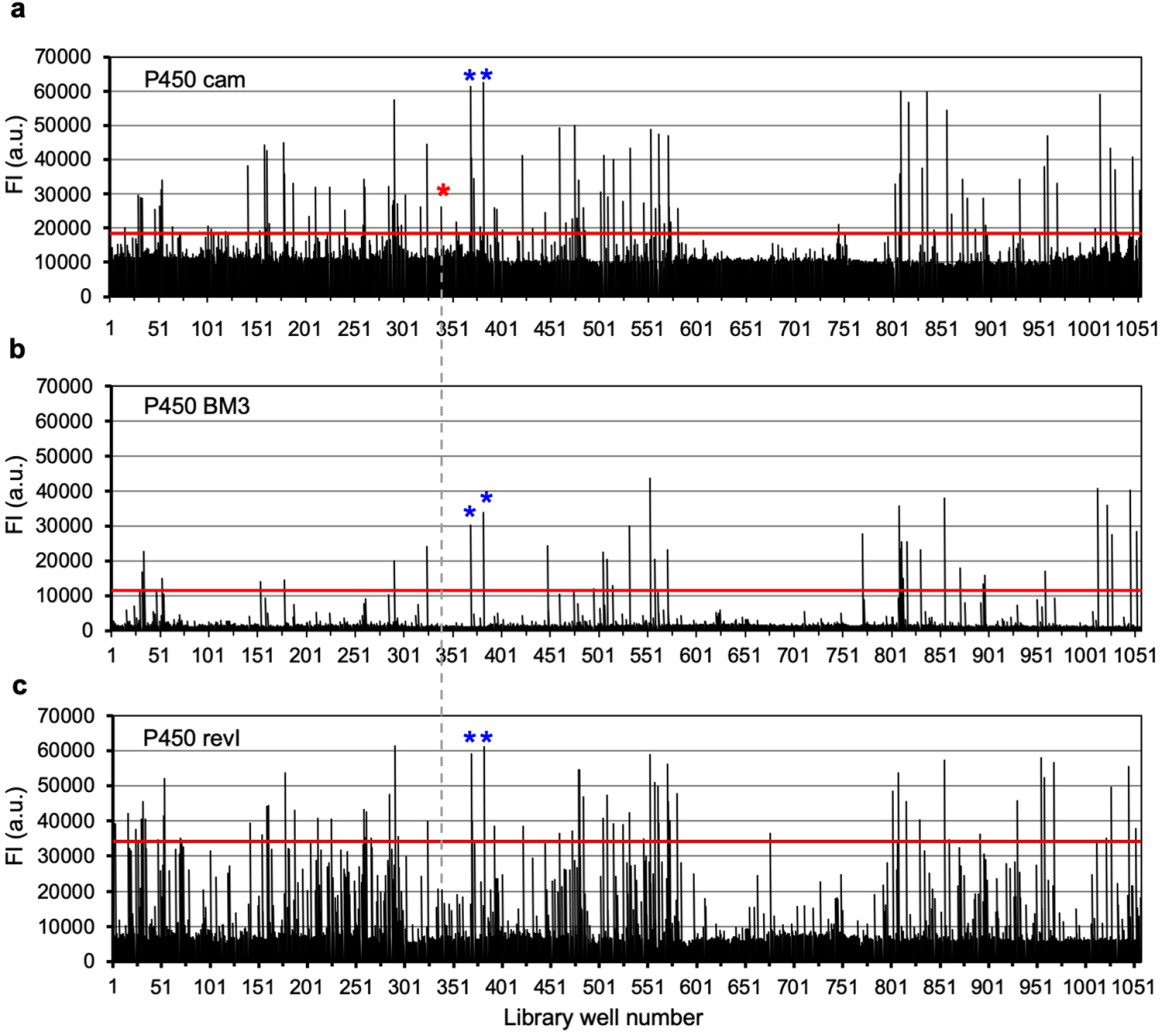
Results of first screening of P450 substrates by using the 2-acetylbenzofuran (2-ABF) method. Compounds in 96-well plates were incubated with (**a**) NADH in the presence of P450 cam, putidaredoxin, and putidaredoxin reductase, (**b**) NADPH in the presence of P450 BM3, or (**c**) NADPH in the presence of P450 revI, ferredoxin, and ferredoxin reductase. The NAD^+^ or NADP^+^ generated in each well was reacted with 2-ABF to generate fluorescent products, and their fluorescence is shown (mean of 2 replicates). Red lines indicate the threshold in each case (see Materials and Methods for details). Blue asterisks and a red asterisk indicate the example of common hit compounds (false positives) and a hit compound unique for P450 cam, respectively.

Next, we examined the crucial question of whether substrates for a P450 of interest were correctly judged by using the integrated P450 substrate screening system. For this purpose, P450 cam, an assessor P450, was used as a model for substrate screening. Each compound in the pilot library was incubated with NADH in the presence of P450 cam and its electron transfer partners (putidaredoxin and putidaredoxin reductase)^56^. The library compounds were also incubated with NADPH in the presence of P450 BM3 or P450 revI and its electron transfer partners. The resultant solutions were evaluated by the 2-ABF method. The results are summarized in Fig. 3 (see also Supporting Tables 1–1~1–3). The threshold values for each substrate screening were set as described in the Materials and Methods section, and compounds yielding hits for all three P450 species were eliminated as false positives. For example, the signals with a blue asterisk in Fig. 3a–c are commonly found in all P450s, and were therefore eliminated. These two compounds labeled with blue asterisks were analyzed in detail (data not shown), and were found not to be common substrates for these P450s. On the other hand, the signal with a red asterisk in Fig. 3a is unique to P450 cam, thus signaling selection of the corresponding compound as one of the hit compounds. As a result, 35 compounds were selected as first hit compounds for P450 cam.

**Figure 4:**
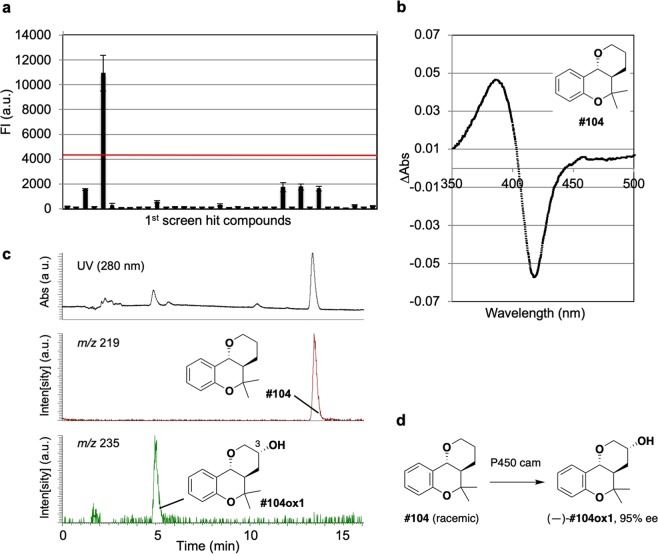
Screening and identification of the P450 cam substrate. (**a**) Results of the second screening of P450 cam substrates by using PF-1 as an indicator (mean of 3 replicates, error bars show standard deviation). The red line indicates the threshold (mean + 2 SD). (**b**) Substrate-induced type I spectral change observed for an addition of compound **#104** (10 mM) to P450 cam (3 mM). (**c**) HPLC and selected ion chromatograms of the *in vitro* reaction of P450 cam with compound **#104**. (**d**) P450 cam-mediated reaction of racemic compound **#104** proceeds in a site-, diastereo- and enantioselective manner to give enantiopure (—)-**#104ox1**.

Next, the second screening using PF-1 was applied to the first hits from the P450 cam substrate screening, and one compound was eliminated as a potential uncoupling inducer (Fig. 4a; see also Supporting Fig. 4 for compound numbers). The remaining second hits were then tested to determine whether they induce type I spectral change (i.e., a reduction in the Soret absorption band at ca. 420 nm and a corresponding increase in the maximum at ca. 390 nm with an isosbestic point at ca. 407 nm) on P450 cam and whether they are converted to a single or several major products upon incubation with P450 cam. As a result, two compounds were finally screened as exemplary substrates for P450 cam. The first compound was the natural substrate d-camphor, demonstrating that the integrated screening system works successfully. The second compound was fused tetrahydrobenzopyran (library compound **#104**; see the inset in Fig. 4b)^57,58^. Compound **#104** was recognized by P450 cam to induce typical type-I spectral change (Fig. 4b; see also Supporting Fig. 5) and then selectively oxidized by P450 cam to produce a mono-oxidation product **#104ox1** (Fig. 4c). The structure of **#104ox1** was unambiguously determined by NMR analysis (Supporting Fig. 6) and chemical synthesis (Supporting Scheme 1 and Supporting Methods).

**Figure 5:**
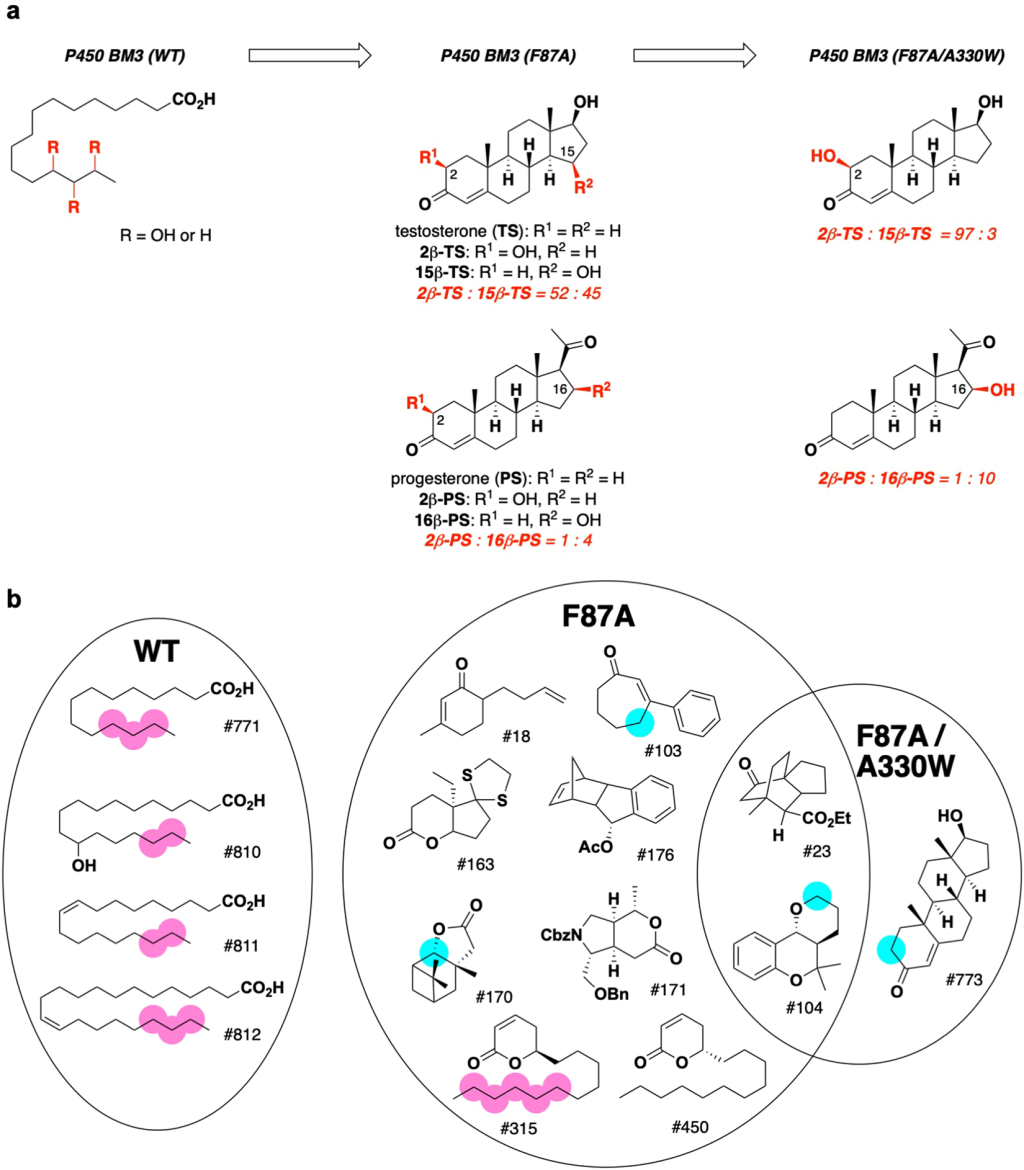
Substrate selectivity analysis of laboratory-evolved P450 BM3 mutants. (**a**) Stepwise introduction of two amino acid mutations enabled P450 BM3 to oxidize testosterone (TS) and progesterone (PS) in a highly regio- and stereoselective manner (these data were taken from ref. ^19^). (**b**) Exemplary substrates identified from the in-house library for P450 BM3(WT), P450 BM3(F87A) and BM3(F87A/A330W). Red and blue circles indicate the oxidized positions determined by GC/MS (red circles) and NMR analyses (blue circles).

Since the stock compound **#104** was racemic and was not entirely consumed during the enzymatic reaction, we considered the possibility of kinetic resolution by P450 cam. Thus, both enantiomers of **#104** were prepared in an optically active form (Supporting Scheme 1 and Supporting Methods) and were tested independently for kinetic analysis (see the Materials and Methods section). The kinetic experiments using both enantiomers clarified that P450 cam selectively recognizes and oxidizes (–)-**#104**: The reaction apparently followed Michaelis-Menten kinetics. The *K*_m_ and *k*_cat_ values for (–)-**#104** were 155 ± 16 μM and 0.61 ± 0.02 min^−1^, respectively, giving a catalytic efficiency of 3.9 × 10^−3^ min^−1^ μM^−1^. (+)-**#104** showed a higher *K*_m_ value (403 ± 39 μM) and a lower *k*_cat_ values (0.025 ± 0.001 min^−1^) than (–)-**#104**, giving a catalytic efficiency of 6.2 × 10^−5^ min^−1^ μM^−1^. The selectivity factor *E* amounts to 63^59^. In the 10 mg scale reaction, incubation of racemic **#104** with P450 cam produced (–)-**#104ox1** (30% yield, 95% ee) along with recovered starting material (38%, 78% ee) (Fig. 4d and Supporting Fig. 7).

Taken together, these results demonstrate that the integrated P450 substrate screening can be used not only for discriminating known natural substrate(s), but also for exploring previously unidentified exemplary substrate(s).

### Analysis of the transition of exemplary substrates for laboratory-evolved P450 mutants

We then applied the integrated P450 substrate screening system to analyze the transition of exemplary substrates for laboratory-evolved P450 BM3 mutants. The mutants we chose in this study were P450 BM3 (F87A) and P450 BM3 (F87A/A330W). Reetz and co-workers reported that the P450 BM3 (F87A) mutant is capable of oxidizing testosterone to generate 2β- and 15β-hydroxytestosterone in a 52:45 ratio, whereas the introduction of an additional mutation A330W led to an enzyme having excellent 2β-selectivity (Fig. 5a)^19^. A similar trend was observed in the oxidation of progesterone: P450 BM3 (F87A) oxidized progesterone to give 2β- and 16β-hydroxyprogesterone in a 1:4 ratio and the selectivity was improved to 1:10 when P450 BM3 (F87A/A330W) was used (Fig. 5a). The observed selectivity in steroid hydroxylation by P450 BM3 mutants was explained by molecular dynamics simulation and docking analysis^19^. However, it is also of particular interest to know what change in the exemplary substrates occurred during the successive introduction of mutations.

To analyze the substrate selectivity of each enzyme and thus the transition in substrate selectivity, exemplary substrates for P450 BM3 (WT) and the two mutants were screened from the in-house pilot library by using the integrated substrate screening system. The numbers of selected compounds in each constituent screening process are summarized in Table 1 (See also Supporting Figs. 8–15 and Supporting Tables 1–4 and 1–5.

**Table 1:** Number of hit compounds in each constituent process for the screening of exemplary substrates for P450 BM3 (WT) and the two mutants.

	WT	F87A	F87A/A330W
First screening	8	38	28
Second screening	8	36	26
Third screening	5	17	5
Forth screening	4	10	3

As a result, 4, 10, and 3 compounds were selected as exemplary substrates for P450 BM3 (WT), P450 BM3 (F87A), and P450 BM3 (F87A/A330W), respectively (Fig. 5b). Two compounds were found to be common exemplary substrates for the two mutant enzymes. No overlap of exemplary substrates was observed between P450 BM3 (WT) and P450 BM3 (F87A).

As expected^3^, the long chain fatty acids were selected as exemplary substrates for P450 BM3 (WT). Some of them possess a hydroxyl group or a *cis*-double bond in the middle of the carbon chain, suggesting that the substrate-binding pocket is flexible or spacious enough to accept such substrates. GC/MS analysis of the oxidized products of all exemplary substrates showed that P450 BM3 (WT)-catalyzed oxidation takes place at the ω − 1 to ω − 3 positions (pink circles in Fig. 5b: Also see Supporting Methods and Supporting Figs. 16–19), depending on the substrate, as observed for fatty acid substrates having a saturated aliphatic chain.

On the other hand, the structures of exemplary substrates for P450 BM3 (F87A) were significantly different from those for the P450 BM3 (WT). The P450 BM3 (WT) is known to recognize the terminal carboxylic acid moiety of fatty acids with Y51 and R47 residues at the substrate-binding site, and these residues remain unchanged in P450 BM3 (F87A). It should be noted that fatty acids including lauric acid (#772) and myristic acid (#771) were still ranked highly in the first screening, although they did not qualify as exemplary substrates (Supporting Table 1–4). Among the exemplary substrates for P450 BM3 (F87A), only two compounds, **#315** and **#450**, which were found to be an enantiomeric pair of a 6-membered unsaturated lactone, possessed a long alkyl chain as with the P450 BM3 (WT) natural substrates. However, unlike in the fatty acid oxidation by P450 BM3 (WT), compound **#315** is oxidized by P450 BM3 (F87A) at the ω − 1 to ω − 5 positions, as revealed by GC/MS techniques (Supporting Fig. 20).

**Figure 6:**
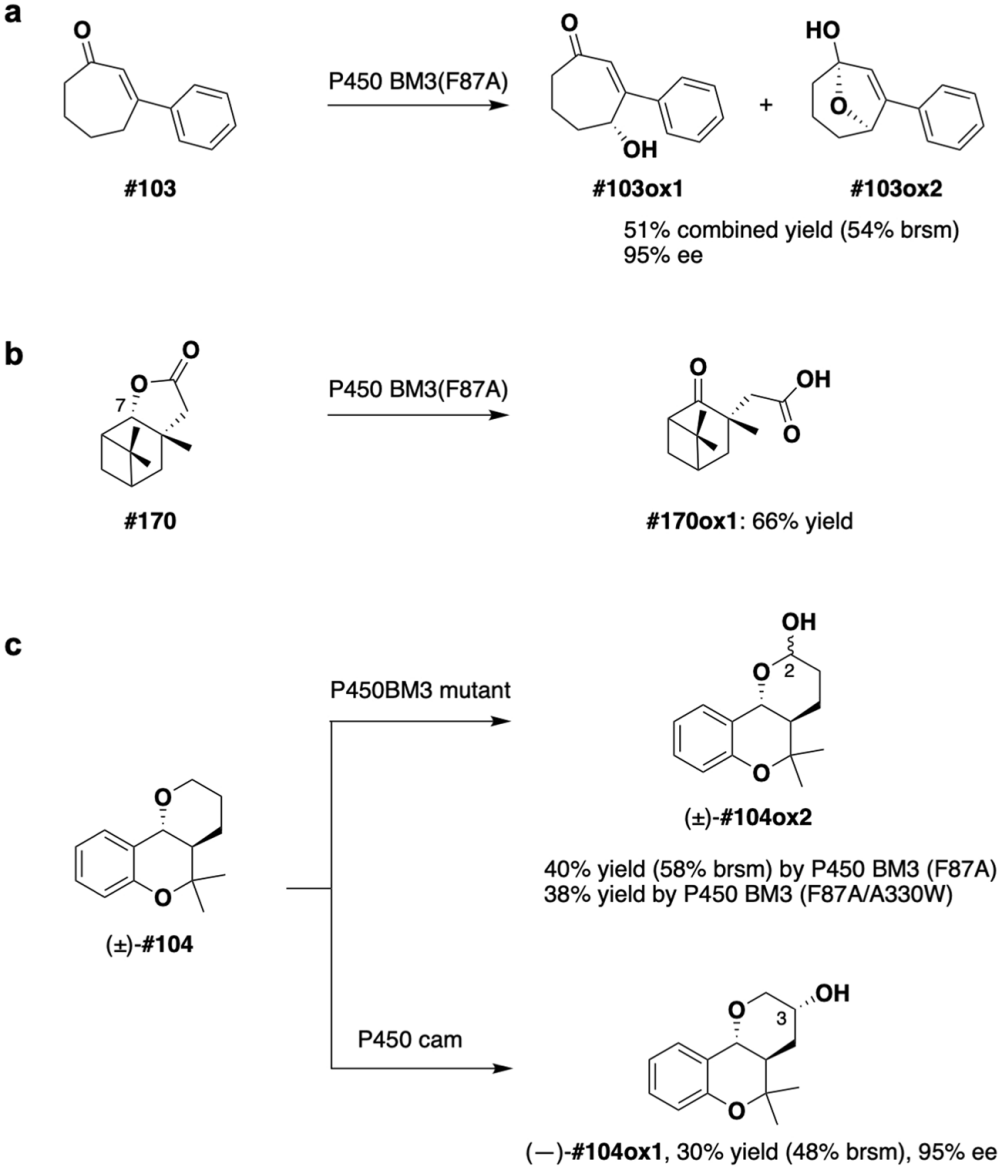
Unique reactivities of P450 BM3 mutants and P450 cam found in this study. (**a**) Enantio- and g-selective C-H oxidation of b-substituted cycloheptenone. (**b**) Oxidation of a highly hindered C-H bond. (**c**) Site-selective C-H oxidation by different P450 species. brsm = based on recovered starting material.

A variety of cyclic compounds took priority as exemplary substrates for P450 BM3 (F87A). The unique reactivity of P450 BM3 (F87A) was uncovered by analyzing the structure of the reaction products of the selected exemplary substrates (Fig. 6). For example, compound **#103**^60,61^ was oxidized by P450 BM3 (F87A) at the γ-position in an enantioselective manner to give γ-hydroxyenone **#103ox1** as an equilibrium mixture with hemiacetal **#103ox2** in > 95% ee (Fig. 6a). The structure and enantiomeric excess of **#103ox1** were determined by ^1^H-NMR analyses after leading to its acetate and MTPA esters, respectively (Supporting Figs. 21–23 and Supporting Methods). Oxidation of **#170** occurred at a hindered C-7 position to give the unique keto-carboxylic acid **#170ox1** (Fig. 6b). The structure of **#170ox1** was again determined by NMR analyses (Supporting Figs. 24–26). Among the exemplary substrates for P450 BM3 (F87A/A330W), two compounds overlapped with the exemplary substrates for P450 BM3 (F87A), as described above. Both enzymes were found to catalyze the oxidation of **#104** at the C-2 position to afford enantiomeric pairs of hemiacetals (±)-**#104ox2** (Fig. 6c, Supporting Figs. 27 and 28 and Supporting Methods) with almost no enantioselectivity. Only testosterone (**#773**) was unique for the P450 BM3 (F87A/A330W) mutant (Fig. 5d). Progesterone was included in the pilot library, but it did not qualify as an exemplary substrate by our integrated substrate screening system. We evaluated the activities (oxidation rates, conversions, and coupling efficiencies) of P450 BM3 (F87A/A330W) towards compound **#104**, testosterone and progesterone, and found that these factors of the former two compounds were indeed superior to those of progesterone (Supporting Table 3). These results are in good agreement with the fact that the laboratory evolution by Reetz *et al*.^19^ was performed to achieve selective oxidation of testosterone, not of progesterone.

Taken together, the analysis of P450 BM3 (WT) and the two P450BM3 mutants using the integrated P450 substrate screening system revealed that the exemplary substrates underwent a distinct transition via stepwise amino acid changes. In addition, some selective oxidations that could not be achieved with other oxidants were discovered during this analysis.

## Conclusion

The concept described in this study complements previous approaches to studying the substrate acceptance of P450 enzymes, including the Urlacher approach based on cluster screening^62^, and the high-throughput fingerprinting technique developed by the group of Fasan^63,64^. We have constructed an integrated P450 substrate screening system by combining two microtiter plate-based methods utilizing two fluorogenic probes (2-ABF and PF-1) and conventional spectroscopic- and LC-MS-based methods. Combinatorial use of the developed substrate screening system and in-house small molecule library containing sp^3^-rich synthetic compounds enabled the discovery of new P450 exemplary substrates and synthetically useful transformations. Enantioselective C-H oxidation^65,66^ (i.e., **#103** → **#103ox1** in this study) and C-H oxidative kinetic resolution^67^ (i.e., **#104** → **#104ox1**) are important topics in synthetic organic chemistry and chiral separation chemistry. Thus, the discovered reactions provide good starting points for future research, including for the preparation of new chiral building blocks and the development of practical chiral separation methods.

Our newly developed method would also allow the selection of uncoupling inducers, although this application was not fully analyzed in this study. Understanding of the structural differences between exemplary substrates and uncoupling inducers for a P450 of interest is very important for designing P450 molecules having high coupling efficiency.

Using the integrated P450 substrate screening system, we were able to analyze the transition of exemplary substrates for P450 BM3 mutants. This analysis revealed that the exemplary substrates underwent a distinct transition via successive amino acid mutations: the exemplary substrates changed from long-chain fatty acids (4 hits for the WT) via diverse cyclic/polycyclic compounds having a short or no side chain (10 hits for the F87A mutant) to a narrow set of polycyclic compounds, including testosterone (3 hits for the F87A/A330W mutant). Most of the previous studies focused on fairly small sets of similar substrates or substrate candidates. To the best of our knowledge, the present study is only the second case^68^ in which more than 1,000 structurally diverse compounds were screened against a P450 enzyme. Information from these efforts will complement the structural information obtained from X-ray analysis and molecular dynamics simulation.

Finally, we believe that the present approach should work nicely in ultra-high-throughput droplet microfluidic devices^69–72^ or in conjunction with the nanogram-scale crystalline sponge method^73^, which would allow, inter alia, massive numbers of P450 mutants to be assessed for catalytic profiling against thousands of small- or mid-sized molecules in the near future.

## Materials and Methods

### Materials

PeroxyFluor-1 (PF-1)^42^ and 2-acetylbenzofuran (2-ABF)^35^ were synthesized according to the reported procedures. Kanamycin, isopropyl-β-d-thiogalactopyranoside (IPTG), and 5-aminolevulinic acid were purchased from commercial suppliers. Lysozyme, DNase, and protein inhibitor cocktail (PI cocktail) were purchased from Roche Diagnostics. Spinach ferredoxin (SFdx) and spinach ferredoxin reductase (SFdR) were purchased from Sigma Aldrich.

### DNA constructs

DNA constructs of P450 cam, putidaredoxin (Pdx), and putidaredoxin reductase (PdR) were gifts from Prof. Shingo Nagano (Tottori University). The construct of P450 BM3 (WT) was from Prof. Stephen G. Sligar (University of Illinois), Prof. Yoshihito Watanabe (Nagoya University), and Prof. Osami Shoji (Nagoya University). The constructs of P450 BM3 (F87A) and P450 BM3 (F87A/A330W) were from Prof. Manfred T. Reetz (Max Planck Institute for Coal Research). The construct of P450 revI was prepared according to the reported method^37^.

### Protein expression and purification

#### P450 cam, Pdx, PdR, P450 BM3 (WT), P450 BM3 (F87A), P450 BM3 (F87A/A330W), and P450 revI

These proteins were expressed and purified according to the previously reported method with some modifications^19,34,35,37^. See Supporting Methods for details.

### P450 substrate screening procedure

#### First screening: Measurement of NAD(P)^+^ production using the 2-acetylbenzofuran method

An aqueous solution of NAD(P)H (250 μM, 10 μL; final concentration: 50 µM) was added to wells of 96-well round-bottomed fluorescence plates (Nunc) each containing a solution consisting of a library molecule (200 µM), P450, and electron transfer proteins (the concentrations of the proteins are summarized in Table 2) in potassium phosphate buffer (50 mM, pH 7.4, 51 µL) containing DMSO (1 µL). The plates were incubated at 25 °C for 30 min. After incubation, the plates were cooled to 4 °C (on ice), and a cooled (0 °C) solution of 2-ABF in EtOH (25 mM, 20 µL) and cooled (0 °C) aqueous KOH (0.5 M, 20 µL) were added to each well. After incubating at 4 °C for 20 min, 90% formic acid (90 µL) was added to each well, and the plates were allowed to warm to 25 °C for 20 min. The fluorescence of generated 2,7-naphthyridinone derivatives in each well was measured by a Synergy Mx microplate reader (BioTek) with excitation/emission wavelengths set at 421/480 nm. Each experiment was repeated twice (n = 2), and the average of the two experiments was calculated for each library compound. A threshold value for the P450 cam substrate screening (red line in Fig. 3a) was set by using a statistic called the interquartile range (IQR) at the Q3 (third quartile) plus 1.5 times IQR, whereas the thresholds for the P450 BM3 and P450 revI and P450 BM3 mutants (red lines in Fig. 3b,c) were set as the mean plus 2 times the SD (standard deviation) in each case. When the threshold value for P450 cam substrate screen was set as mean + 2 × SD, no hit compounds were obtained after the third screening. In general, the interquartile range is used in case the data variation is large. Library compounds inducing stronger fluorescence above the threshold were selected as hit compounds. Among these, compounds inducing fluorescence signals stronger than the value of Q3 + 1.5 × IQR or the mean + SD, whichever was lower, in all three assessor P450s were regarded as false positives and thus eliminated.

**Table 2:** Protein concentration used in the first screening.

P450	ferredoxin	ferredoxin reductase
P450 cam (100 nM)	Pdx (200 nM)	PdR (100 nM)
P450 revI (40 nM)	SFdx (4.0 µg/mL)	SFdR (40 mU/mL)
P450 BM3 (WT) (4 nM)	—	—
P450 BM3 (F87A) (50 nM)	—	—
P450 BM3 (F87A/A330W) (50 nM)	—	—

#### Second screening: Measurement of H_2_O_2_ production using PF-1

An aqueous solution of NAD(P)H (250 μM, 10 μL; final concentration: 50 µM) was added to wells of a 96-well round-bottomed fluorescence plate (Nunc) each containing a solution consisting of a hit compound (200 μM), P450, electron transfer proteins (the concentrations of the proteins are summarized in Table 2), and PF-1 (50 μM) in a potassium phosphate buffer (50 mM, pH 7.4, 52 μL) containing DMSO (2 μL) at 25 °C. The plate was incubated at the same temperature for 30 min. The fluorescence intensity of each well was read on a microplate reader with excitation/emission wavelengths set at 490/516 nm, respectively. Each experiment was repeated three times (n = 3), and the average and standard deviation was calculated for each test compound. The threshold values were set to mean + 2 SD. Library compounds inducing stronger fluorescence than the threshold were regarded as potential uncoupling inducers and eliminated from the hit compounds. The remaining compounds were thus selected as hit compounds.

#### Third screening: Measurement of substrate-induced spectral change in the Soret band

One hundred µL of P450 solution (final concentration: 3 or 5 µM) in potassium phosphate buffer (50 mM, pH 7.4, 100 µL) was transferred to a micro rectangular quartz cell (JASCO). To obtain background spectra, an absorbance spectrum (350–500 nm) was measured by a V-630BIO spectrophotometer (JASCO). A solution of each test compound (10 mM in DMSO, 0.1 µL; final concentration: 10 µM) was added to each well, and the resulting solution was mixed by repeatedly pipetting up and down. The absorbance spectrum of each well was measured again to obtain sample spectra, and the difference spectra were calculated by subtracting background spectra from sample spectra. Test compounds inducing type I spectral change with difference spectral maxima of ca. 390 nm and minima of about 420 nm were selected as hit compounds. This screening can also be done using a 96-well quartz plate (NSG) and a Synergy Mx microplate reader (BioTek).

#### Fourth screening: P450-mediated oxidation and LC/MS analysis of the monoxidation product

Typically, oxidation of a hit compound using purified P450 enzyme was performed by incubating a solution of P450 (P450 BM3 and its mutants: 3 µM; P450 cam: 0.75 µM in the presence of Pdx (1.5 μM) and PdR (0.75 μM)), test compound (500 µM), and NAD(P)H (230 µM) in potassium phosphate buffer (100 mM, pH 7.4, 5% glycerol, 5% DMSO, 500 μL) at 25 °C for 2–12 h with orbital shaking at 250 rpm. The progress of the oxidation reaction was monitored by LC/MS analysis. The reaction was stopped by an addition of EtOAc (500 μL). Aqueous 0.1 *N* HCl (50 μL) was then added when the test compound was long chain carboxylic acid. The aqueous layer was extracted with EtOAc (500 μL × 3). The combined organic layers were concentrated in vacuo. The residue was diluted to 100 or 500 μg/mL, and subjected to LC/MS analysis using an Exactive mass spectrometer (Thermo) equipped with an Accela LC system (Thermo). Compounds converted to single or several monoxidation products were regarded as good substrates.

LC/MS conditions

Column: Inertsil ODS-3 (ϕ 4.6 mm × 75 mm, GL science)

Flow rate: 400 μL/min

Eluent A: CH_3_CN containing 0.05% HCO_2_H

Eluent B: 0.05% aqueous HCO_2_H

Elution conditions:

Method A: 50% A/0–5 min, 50–90% A/5–15 min, 90% A/15–20 min. Ionization mode: ESI (+).

Method B (only for **#811, #812**): 50% A/0–5 min, 50–100% A/5–15 min, 100% A/15–35 min. Ionization mode: ESI (–).

Method C (only for **#104**): 20% A/0–5 min, 20–50% A/5–15 min, 50–90% A/15–20 min, 90% A/20–21 min. Ionization mode: ESI (+).

Method D (only for **#773**): 50% A/0–5 min, 50–90% A/5–25 min, 90–100% A/25–26 min. Ionization mode: ESI (+).

### Kinetic assays

Kinetic assays were performed in 1.5-mL tubes with a final reaction volume of 0.1 mL containing potassium phosphate buffer (100 mM, pH 7.4), NADH (230 µM), Pdx (2.77 µM), PdR (1.1 µM), P450 cam (25 nM), and substrates. Substrate concentrations of (–)-**#104** and (+)-**#104** were varied from 50 to 1000 μM and 100–2500 μM, respectively. After preincubation at 25 °C for 2 min, the reactions were initiated by the addition of P450 cam. After incubation for 20 min, the reaction was terminated by the rapid addition of MeCN. After centrifugation for 30 min at 15,000 rpm, the supernatant was subjected to UPLC/MS (ACQUITY UPLC H-Class (Waters)/API3200 (AB SCIEX)) under the conditions described below. The product peak was calculated from the standard curve, which was obtained from the standards (–)-**#104** and (+)-**#104**. The enzyme-specific activity (μmol product formed/min/μmol of enzyme) was calculated by time-dependent product formation. The kinetic constants were calculated by a nonlinear regression fit to the Michaelis-Menten equation using SigmaPlot12 software (Systat Software Inc., USA).

UPLC/MS conditions:

Column: XTera® MS C18 column, 5 μm, ϕ 2.1 × 150 mm (Waters)

Flow rate: 0.7 mL/min

Solvent A: 0.05% formic acid

Solvent B: MeCN

Method: 10%–100% B/0–1.9 min, 100% B/1.9–2.86 min, 10% B/2.87–4 min.

Ionization mode: ESI (+).

## Supplementary information

Supporting information

## References

[1] Nelson DR (2009). The cytochrome P450 homepage. Hum. Genomics.

[2] Guengerich, F. P. Human cytochrome P450 enzymes, 3^rd^ Edition. [Ortiz de Montellano, P. R. (ed.)], *Cytochrome P450: Structure, Mechanism, and Biochemistry*, Chapter 10, 377–530 (Kluwer Academic/Plenum Publishers, 2005).

[3] Whitehouse CJC, Bell SG, Wong LL (2012). P450BM3 (CYP102A1): Connecting the dots. Chem. Soc. Rev..

[4] Kirchmair J (2015). Predicting drug metabolism: Experiment and/or computation?. Nat. Rev. Drug Discov..

[5] Guengerich FP, Tang ZM, Cheng QA, Salamanca-Pinzon SG (2011). Approaches to deorphanization of human and microbial cytochrome P450 enzymes. Biochim. Biophys. Acta Proteins Proteom..

[6] Guengerich FP, Tang ZM, Salamanca-Pinzon SG, Cheng QA (2010). Characterizing proteins of unknown function: Orphan cytochrome P450 enzymes as a paradigm. Mol. Interv..

[7] Guengerich FP, Cheng Q (2011). Orphans in the human cytochrome P450 superfamily: Approaches to discovering functions and relevance in pharmacology. Pharmacol. Rev..

[8] Rudolf JD, Chang CY, Ma M, Shen B (2017). Cytochromes P450 for natural product biosynthesis in *Streptomyces*: sequence, structure, and function. Nat. Prod. Rep..

[9] Girvan HM, Munro AW (2016). Applications of microbial cytochrome P450 enzymes in biotechnology and synthetic biology. Curr. Opin. Chem. Biol..

[10] Fasan R (2012). Tuning P450 enzymes as oxidation catalysts. ACS Catal..

[11] Peterson DH, Murray HC (1952). Microbiological oxygenation of steroids at carbon-11. J. Am. Chem. Soc..

[12] Sasaki J (1992). Transformation of vitamin D3 to 1α,25-dihydroxyvitamin D3 via 25-hydroxyvitamin D3 using *Amycolata* sp. strains. Appl. Microbiol. Biotechnol..

[13] Sasaki J, Mikami A, Mizoue K, Omura S (1991). Transformation of 25-hydroxyvitamin D3 and 1α-hydroxyvitamin D3 to 1α,25-dihydroxyvitamin D3 by using *Streptomyces* sp. strains. Appl. Environ. Microbiol..

[14] Fasan R, Chen MM, Crook NC, Arnold FH (2007). Engineered alkane-hydroxylating cytochrome P450(BM3) exhibiting nativelike catalytic properties. Angew. Chem. Int. Ed..

[15] Narhi LO, Fulco AJ (1986). Characterization of a catalytically self-sufficient 119,000 dalton cytochrome P450 monooxygenase induced by barbiturates in *Bacillus megaterium*. J. Biol. Chem..

[16] Boddupalli SS, Pramanik BC, Slaughter CA, Estabrook RW, Peterson JA (1992). Fatty acid monooxygenation by P450BM3 - Product identification and proposed mechanisms for the sequential hydroxylation Rreactions. Arch. Biochem. Biophys..

[17] Jung ST, Lauchli R, Arnold FH (2011). Cytochrome P450: Taming a wild type enzyme. Curr. Opin. Biotechnol..

[18] Reetz MT (2011). Laboratory evolution of stereoselective enzymes: A prolific source of catalysts for asymmetric reactions. Angew. Chem. Int. Ed..

[19] Kille S, Zilly FE, Acevedo JP, Reetz MT (2011). Regio- and stereoselectivity of P450-catalysed hydroxylation of steroids controlled by laboratory evolution. Nat.Chem..

[20] Butler CF (2013). Key mutations alter the cytochrome P450 BM3 conformational landscape and remove inherent substrate bias. J. Biol. Chem..

[21] Peters MW, Meinhold P, Glieder A, Arnold FH (2003). Regio- and enantioselective alkane hydroxylation with engineered cytochromes P450 BM-3. J. Am. Chem. Soc..

[22] Rabe KS, Gandubert VJ, Spengler M, Erkelenz M, Niemeyer CM (2008). Engineering and assaying of cytochrome P450 biocatalysts. Anal. Bioanal. Chem..

[23] Ansede JH, Thakker DR (2004). High-throughput screening for stability and inhibitory activity of compounds toward cytochrome P450-mediated metabolism. J. Pharm. Sci..

[24] Olry A, Schneider-Belhaddad F, Heintz D, Werck-Reichhart D (2007). A medium-throughput screening assay to determine catalytic activities of oxygen-consuming enzymes: A new tool for functional characterization of cytochrome P450 and other oxygenases. Plant J..

[25] Gorsky LD, Koop DR, Coon MJ (1984). On the stoichiometry of the oxidase and monooxygenase reactions catalyzed by liver microsomal cytochrome P450 - products of oxygen reduction. J. Biol. Chem..

[26] Staudt H, Lichtenberger F, Ullrich V (1974). Role of NADH in uncoupled microsomal monoxygenations. Eur. J. Biochem..

[27] Tsotsou GE, Cass AEG, Gilardi G (2002). High throughput assay for cytochrome P450BM3 for screening libraries of substrates and combinatorial mutants. Biosens. Bioelectron..

[28] Furuya T (2008). Characterization of orphan monooxygenases by rapid substrate screening using FT-ICR mass spectrometry. Chem. Biol..

[29] de Rond T (2019). A high-throughput mass spectrometric enzyme activity assay enabling the discovery of cytochrome P450 biocatalysts. Angew. Chem. Int. Ed..

[30] Huff JW (1947). The Fluorescent condensation product of N1-methylnicotinamide and acetone. 1. Synthesis and properties. J. Biol. Chem..

[31] Clark BR, Halpern RM, Smith RA (1975). Fluorimetric method for quantitation in picomole range of N1-methylnicotinamide and nicotinamide in serum. Anal. Biochem..

[32] Kaplan NO, Colowick SP, Barnes CC (1951). Effect of alkali on diphosphopyridine nucleotide. J. Biol. Chem..

[33] Guilbert CC, Johnson SL (1971). Isolation and characterization of fluorescent alkali product from diphosphopyridine nucleotide. Biochemistry.

[34] Takayama H (2011). Detection of cytochrome P450 substrates by using a small-molecule droplet array on an NADH-immobilized solid surface. Chembiochem.

[35] Moriya T, Kawamata A, Takahashi Y, Iwabuchi Y, Kanoh N (2013). An improved fluorogenic NAD(P)^+^ detection method using 2-acetylbenzofuran: Its origin and application. Chem. Commun..

[36] Katagiri M, Ganguli BN, Gunsalus IC (1968). A soluble cytochrome P450 functional in methylene hydroxylation. J. Biol. Chem..

[37] Takahashi S (2014). Structure-function analyses of cytochrome P450revI involved in reveromycin. A biosynthesis and evaluation of the biological activity of its substrate, reveromycin T. J. Biol. Chem..

[38] Takahashi S (2011). Reveromycin A biosynthesis uses RevG and RevJ for stereospecific spiroacetal formation. Nat. Chem. Biol..

[39] Loida PJ, Sligar SG (1993). Molecular recognition in cytochrome P450 - Mechanism for the control of uncoupling reactions. Biochemistry.

[40] Winkler, B. S., Boulton, M. E., Gottsch, J. D. & Sternberg, P. Oxidative damage and age-related macular degeneration. *Mol. Vis*. **5** (1999). PMC1773059 10562656

[41] Miyamoto M (2015). Membrane anchor of cytochrome P450 reductase suppresses the uncoupling of cytochrome P450. Chem. Pharm. Bull..

[42] Chang MCY, Pralle A, Isacoff EY, Chang CJ (2004). A selective, cell-permeable optical probe for hydrogen peroxide in living cells. J. Am. Chem. Soc..

[43] Schenkman JB, Remmer H, Estabrook RW (1967). Spectral studies of drug interaction with hepatic microsomal cytochrome. Mol. Pharmacol..

[44] Peterson JA (1971). Camphor binding by *Pseudomonas putida* cytochrome P450. Arch. Biochem. Biophys..

[45] Iwabuchi Y (2013). Discovery and exploitation of AZADO: The highly active catalyst for alcohol oxidation. Chem. Pharm. Bull..

[46] Kanoh N, Sakanishi K, Iimori E, Nishimura K, Iwabuchi Y (2011). Asymmetric total synthesis of (−)-scabronine G via intramolecular double Michael reaction and Prins cyclization. Org. Lett..

[47] Kawasumi M, Kanoh N, Iwabuchi Y (2011). Concise entry to both enantiomers of 8-oxabicyclo[3.2.1]oct-3-en-2-one based on novel oxidative etherification: Formal synthesis of (+)-sundiversifolide. Org. Lett..

[48] Ikeda S, Shibuya M, Kanoh N, Iwabuchi Y (2009). Synthetic studies on daphnicyclidin A: Enantiocontrolled construction of the BCD ring system. Org. Lett..

[49] Ikeda, S., Shibuya, M. & Iwabuchi, Y. Asymmetric total synthesis of martinelline and martinellic acid. *Chem. Commun*. 504–506 (2007). 10.1039/b611121a 17252109

[50] Kanoh N (2016). Asymmetric total synthesis of heronamides A–C: Stereochemical confirmation and impact of long-range stereochemical communication on the biological activity. Chem. Eur. J..

[51] Uesugi S (2015). Total synthesis and biological evaluation of irciniastatin A (a.k.a. psymberin) and irciniastatin B. J. Org. Chem..

[52] Kanoh N (2014). A concise and unified strategy for synthesis of the C1-C18 macrolactone fragments of FD-891, FD-892 and their analogues: Formal total synthesis of FD-891. Org. Lett..

[53] Yamakoshi H (2010). Total synthesis and determination of the absolute configuration of (−)-idesolide. Org. Lett..

[54] Yamakoshi H (2009). Syntheses of naturally occurring cytotoxic [7.7]paracyclophanes, (−)-cylindrocyclophane A and its enantiomer, and implications for biological activity. Org. Biomol. Chem..

[55] Lovering F, Bikker J, Humblet C (2009). Escape from flatland: Increasing saturation as an approach to improving clinical success. J. Med. Chem..

[56] Gunsalus IC, Wagner GC (1978). Bacterial P-450cam methylene monooxygenase components: Cytochrome m, putidaredoxin, and putidaredoxin reductase. Methods Enzymol..

[57] Miyazaki H, Honda K, Asami M, Inoue S (1999). Stereoselective synthesis of pyrano[3,2-c]benzopyrans via intramolecular cycloaddition of *o*-quinonemethides generated from salicylaldehydes and unsaturated alcohols under very mild conditions. J. Org. Chem..

[58] Inoue, S., Asami, M., Honda, K. & Miyazaki, H. A facile synthesis of angularly-fused 3,4,4α,10β-tetrahydro-2*H*,5*H*-pyrano[3,2-*c*][1]benzopyrans by the one-pot condensation between salicylaldehydes and unsaturated alcohols. *Chem. Lett*. 889–890 (1996).

[59] Straathof AJJ, Jongejan JA (1997). The enantiomeric ratio: origin, determination and prediction. Enzyme Microb. Tech..

[60] Shibuya M, Tomizawa M, Iwabuchi Y (2008). TEMPO/NalO_4_-SiO_2_: A catalytic oxidative rearrangement of tertiary allylic alcohols to β-substituted α,β-unsaturated ketones. Org. Lett..

[61] Shibuya M, Tomizawa M, Iwabuchi Y (2008). Oxidative rearrangement of tertiary allylic alcohols employing oxoammonium salts. J. Org. Chem..

[62] von Buhler C, Le-Huu P, Urlacher VB (2013). Cluster screening: An effective approach for probing the substrate space of uncharacterized cytochrome P450s. Chembiochem.

[63] Zhang KD, El Damaty S, Fasan R (2011). P450 fingerprinting method for rapid discovery of terpene hydroxylating P450 catalysts with diversified regioselectivity. J. Am. Chem. Soc..

[64] Zhang KD, Shafer BM, Demars MD, Stern HA, Fasan R (2012). Controlled oxidation of Rremote sp^3^ C-H bonds in artemisinin via P450 catalysts with fine-tuned regio- and stereoselectivity. J. Am. Chem. Soc..

[65] Ammann SE, Liu W, White MC (2016). Enantioselective allylic C-H oxidation of terminal olefins to isochromans by palladium(II)/chiral sulfoxide catalysis. Angew. Chem. Int. Ed..

[66] Neufeld K, Henssen B, Pietruszka J (2014). Enantioselective allylic hydroxylation of ω-alkenoic acids and esters by P450 BM3 monooxygenase. Angew. Chem. Int. Ed..

[67] Russell, T. A. & Vedejs, E. In *Separation of enantiomers: Synthetic methods* (Ed. by M. Todd) 217–266 (Wiley-VCH, 2014).

[68] Bleif S (2011). Identification of CYP106A2 as a regioselective allylic bacterial diterpene hydroxylase. Chembiochem.

[69] Agresti JJ (2010). Ultrahigh-throughput screening in drop-based microfluidics for directed evolution. Proc. Natl. Acad. Sci. USA.

[70] Baret JC (2009). Fluorescence-activated droplet sorting (FADS): efficient microfluidic cell sorting based on enzymatic activity. Lab Chip.

[71] Mazutis L (2013). Single-cell analysis and sorting using droplet-based microfluidics. Nat. Protoc..

[72] Mazutis L, Baret JC, Griffiths AD (2009). A fast and efficient microfluidic system for highly selective one-to-one droplet fusion. Lab Chip.

[73] Inokuma Y (2013). X-ray analysis on the nanogram to microgram scale using porous complexes. Nature.

