# Supplementary material for: An integrated screening system for the selection of exemplary substrates for natural and engineered cytochrome P450s
Source: Sci Rep. 2019 Dec 2;9:18023. doi: 10.1038/s41598-019-54473-8 (PMC6888865; doi:10.1038/s41598-019-54473-8)
Supplement: Supplementary file 1 — Supporting information [file 41598_2019_54473_MOESM1_ESM.pdf]

Supporting Information

An integrated screening system for the selection of exemplary substrates for natural and engineered cytochrome P450s

Naoki Kanoh,[1,2] * Ayano Kawamata-Asano,[1] Kana Suzuki,[1] Yusuke Takahashi,[1] Takeshi Miyazawa,[3] Takemichi Nakamura,[4] Takashi Moriya,[1] Hiroyuki Hirano,[5] Hiroyuki Osada,[3,5] Yoshiharu Iwabuchi,[1] and Shunji Takahashi[6]

[1] Graduate School of Pharmaceutical Sciences, Tohoku University, 6-3 Aza-Aoba, Aramaki, Aoba-ku, Sendai 980-8578, Japan

[2] Institute of Medicinal Chemistry, Hoshi University, 2-4-1 Ebara, Shinagawa-ku, Tokyo 142-8501, Japan

[3] Chemical Biology Research Group, RIKEN Center for Sustainable Resource Science, 2-1 Hirosawa, Wako, Saitama 351- 0198, Japan

[4] Molecular Structure Characterization Unit, Technology Platform Division, RIKEN Center for Sustainable Resource Science, 2-1 Hirosawa, Wako, Saitama 351- 0198, Japan

[5] Chemical Resource Development Research Unit, Technology Platform Division, RIKEN Center for Sustainable Resource Science, 2-1 Hirosawa, Wako, Saitama 351- 0198, Japan

[6] Natural Product Biosynthesis Research Unit, RIKEN Center for Sustainable Resource Science, 2-1 Hirosawa, Wako, Saitama 351- 0198, Japan

Table of Contents

Supporting Methods

Protein expression and purification……………………………………………………………………1

Chemistry…………………………………………………………………………………………….2

Enzymatic oxidation of exemplary substrates, and derivatization of the oxidation products for structural elucidation and optical purity determination……………………………………………..5

Structure estimation of oxidized products by GC/MS…………………………………………..9

Supporting Figure 1. Effect of the concentration of P450 and its electron transfer proteins on the detection of H_2O_2 using Peroxyfluor-1 (PF-1) ……………………………………………………10

Supporting Figure 2. Detection of H_2O_2 produced by uncoupled turnover of P450 cam using Peroxyfluor-1………………………………………………………………………………...11

Supporting Figure 3. Structure of in-house library compounds…………..………………………12

Supporting Figure 4. Results of the second screening of P450 cam substrates by using PF-1 as an indicator.……………………………………………………………………………………..56

Supporting Figure 5. Substrate-induced spectral changes observed for an addition of a hit compound to P450 cam…………………………………………………………………….......................57

Supporting Figure 6. NMR spectrum of **#104ox1**……..…………...58

Supporting Figure 7. Chiral HPLC analysis of **#104** derived from **#104ox1** and **#104ox1** …………62

Supporting Figure 8. Results of first screening of P450 substrates by using 2-acetylbenzofuran (2-ABF) method.....……………………...……………………………………………………………64

Supporting Figure 9. Results of second screening of P450 substrates by using PF-1..……………..65

Supporting Figure 10. Substrate-induced spectral changes observed for an addition of the hit compound to P450 BM3…..…...…………………………………….…..……………………...66

Supporting Figure 11. Substrate-induced spectral changes observed for an addition of the hit compound to P450 BM3 (F87A)…..…..……………………………...….……………………...67

Supporting Figure 12. Substrate-induced spectral changes observed for an addition of the hit compound to P450 BM3 (F87A/A330W)…..…..……….…..…...………………...……………………...69

Supporting Figure 13. LC/MS analysis of P450 BM3-mediated mono-oxidation products of **#771**, **#810, #811**, and **#812**………………………………………………………………………………....70

Supporting Figure 14. LC/MS analysis of P450 BM3 (F87A)-mediated mono-oxidation products………………..……………………………………………………………………..71

Supporting Figure 15. LC/MS analysis of P450 BM3 (F87A/A330W)-mediated mono-oxidation products……………………..……………………………………………………..74
Supporting Figure 16. GC/MS analysis of P450 BM3-mediated mono-oxidation products of compound **#771**……………………………………………………………………....75
Supporting Figure 17. GC/MS analysis of P450 BM3-mediated mono-oxidation products of compound **#810**………………………………………………………………………76
Supporting Figure 18. GC/MS analysis of P450 BM3-mediated mono-oxidation products of compound **#811**………………………………………………………………………77
Supporting Figure 19. GC/MS analysis of P450 BM3-mediated mono-oxidation products of compound **#812**………………………………………………………………………78
Supporting Figure 20. GC/MS analysis of P450 BM3-mediated mono-oxidation products of compound **#315**………………………………………………………………………79
Supporting Figure 21. ^{1}H-NMR spectrum of an equilibrium mixture of **#103ox1** and **#103ox2**…..80
Supporting Figure 22. NMR spectra of **(–)-S8**………………………………………………..81
Supporting Figure 23. Mosher ester analysis of **#103ox1**………………………………………85
Supporting Figure 24. NMR spectra of **#170**…………………………………………………….87
Supporting Figure 25. NMR spectra of **#170ox1**……………………………………………...91
Supporting Figure 26. NMR spectra of **(+)-S11**………………………………………………….95
Supporting Figure 27. NMR spectra of **#104ox2**……………………………………………..96
Supporting Figure 28. NMR spectra of **S12**…………………………………………………..97
Supporting Scheme 1. Synthesis of optically active **#104** and **#104ox1**…………………………..100
Supporting Table 1. First screening results of substrates for P450 cam, P450 revI, P450 BM3, P450 BM3 (F87A), and P450 BM3 (F87A/A330W)……………………………………………..101
Supporting Table 2. Oxidation rates, conversions and coupling efficiencies of P450 BM3 (F87A/A330W) toward compound **#104**, testosterone, and progesterone…………………………106

Supporting Methods

Protein expression and purification

P450 cam, Pdx, PdR, P450 BM3 (WT), P450 revI

These proteins were expressed and purified as reported previously.[1]

P450 BM3 (F87A) and P450 BM3 (F87A/A330W)

P450 BM3 (F87A) and P450 BM3 (F87A/A330W) were expressed and purified according to the previously reported method[2] with some modifications. *E. coli* BL21(DE3)pLysS cells (Merck) containing the appropriate construct were grown in LB medium (total 1.8 L, 5.0 g/L yeast extract, 10 g/L tryptone, 10 g/L NaCl) containing kanamycin (30 mg/L) with shaking at 37 °C. After cultivation at 37 °C with orbital shaking at 110 rpm and upon reaching an OD_{600} of 0.8–0.9, IPTG (23.8 mg/L) and 5-aminolevulinic acid (27.8 mg/L) were added, and the temperature was set at 30 °C. After 24 h of protein expression, the cells were collected by centrifugation at 6000 x g, 4 °C for 3 min. The pellets were frozen and stored at –78 °C until further processing. For lysis of the bacterial cells, the pellet was thawed on ice, suspended by shaking in 5 mL of 20 mM Tris-HCl buffer (pH 7.4) per 0.45 L of initial culture, and subjected to lysis by adding lysozyme (4 mg/mL) and DNase (20 μg/mL) on ice for 1 h. PI cocktail (Roche Diagnostics) was added, and the lysate was sonicated ten times on ice for 10 sec with 1-min intervals between each sonication. The lysate was centrifuged at 12,000 x g for 20 min at 4 °C, and the supernatant was collected. The following chromatography steps were performed using an ÄKTA start system (GE Healthcare) at 4 °C. The lysate was subjected to anion exchange chromatography using TOYOPEARL® DEAE-650M resin (ϕ 3 cm x 15 cm; Tosoh Bioscience) as a stationary phase equilibrated with elution buffer A (shown below). The elution was performed as follows (flow rate: 5 mL/min; injection volume: 25 mL; elution buffer A: 0.1 M KCl in 20 mM Tris-HCl buffer (pH 7.4); elution buffer B: 0.35 M KCl in 20 mM Tris-HCl buffer (pH 7.4); elution conditions: 100% A/0-30 min, 100-0% A/30-180 min, 100% B/180-210 min; detection: UV 280 nm; 1 fraction = 14 mL). The red fractions obtained at ca. 0.25 M KCl were combined and concentrated to a final volume of 1.5 mL with an Amicon Ultra centrifugal filter 10K device (Millipore). The sample was further purified by gel filtration using Sephacryl S100 HR gel (ϕ 3 cm x 15 cm; GE Healthcare) under the following elution conditions (flow rate: 5 mL/min; injection volume: 2 mL; elution buffer: 20 mM Tris-HCl buffer (pH 7.4); detection: UV 280 nm; 1 fraction = 14 mL for 0-7.5 min, 2 mL after 7.5 min). The red fractions containing the P450 BM3 mutant (checked by SDS-PAGE) were combined and concentrated by using an Amicon Ultra centrifugal filter 10K device and diluted with 20 mM Tris-HCl buffer containing 10% glycerol. The purity of protein

was confirmed by SDS-PAGE. The amount of functional P450 was calculated using the extinction coefficient of 91 $mM^{-1}cm^{-1}$ at 450 nm.[3]

Chemistry

Synthesis of optically active #104 and #104ox1

Oxazolidinone (+)-S2

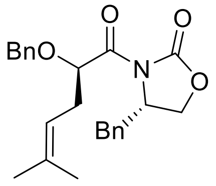

To a cooled (-78 °C) solution of diisopropylamine (1.85 mL, 13.2 mmol) in THF (10 mL) was added *n*-BuLi (1.56 M in hexane, 8.4 mL, 13.2 mmol) at the same temperature. The resulting solution was warmed to 0 °C and stirred for 30 min, then it was cooled to -78 °C. To the solution was added a solution of oxazolidinone **(+)-S1**[4] in THF (1.01 M in THF, 10 mL, 10.1 mmol) dropwise. The resulting solution was stirred at -78 °C for 1 h, then prenyl iodide (13.1 M in THF, 10 mL, 13.1 mmol) was added. After being stirred at -78 °C for 8 h, the reaction was quenched with saturated aqueous NH_4Cl (5 mL) and H_2O (5 mL). The mixture was allowed to warm to rt, and extracted with Et_2O twice. The combined organic layers were washed with brine, dried over anhydrous $MgSO_4$, and concentrated *in vacuo*. Purification by flash chromatography (EtOAc/ Hexane = 1/ 3) provided 1.86 g (4.68 mmol, 46%) of adduct **(+)-S2** as yellow oil.

(+)-S2: $[\alpha]^{20}_D$ + 71.4 (*c* 1.00, $CHCl_3$); IR (neat): 1781 cm^{-1}, 1708 cm^{-1}; ^{1}H-NMR (400 MHz, $CDCl_3$) δ 1.64 (3H, s), 1.71 (3H, s), 2.53 – 2.56 (2H, m), 2.67 (1H, dd, *J* = 13.6, 9.7 Hz), 3.22 (1H, dd, *J* = 13.6, 3.2 Hz), 4.16 (2H, d, *J* = 5.6 Hz), 4.52 (1H, d, *J* = 11.6 Hz), 4.58 – 4.61 (1H, m),4.62 (1H, d, *J* = 11.6 Hz), 5.14 (1H, dd, *J* = 6.8, 5.3 Hz), 5.27 (1H, dd, *J* = 7.2, 7.2 Hz), 7.18 – 7.38 (10H, m); ^{13}C-NMR (100 MHz, $CDCl_3$) δ 17.7, 25.6, 31.6, 37.4, 54.6, 66.4, 72.3, 76.9, 118.3, 127.1, 127.5, 127.9, 128.0, 128.7, 129.15, 129.19, 134.6, 134.8, 137.5, 152.8, 172.5; HR-MS (ESI) calcd. for $C_{24}H_{27}NO_4Na$ (M^+ +Na): 416.1832, found: 416.1806.

Alcohol (–)-S3[5]

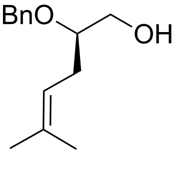

To a cooled (0 °C) solution of **(+)-S2** (1.86 g, 4.68 mmol) in THF (23 mL) was added $LiAlH_4$ (373 mg, 9.83 mmol) at the same temperature. After being stirred at 0 °C for 30 min, the reaction was quenched with saturated aqueous NH_4Cl (5 mL) and H_2O (20 mL), and the mixture was extracted with Et_2O twice. The combined organic layers were washed with brine, dried over anhydrous $MgSO_4$, and concentrated *in vacuo*. Purification of the residue by flash chromatography (EtOAc: Hexane = 1:4) gave 784 mg (3.56 mmol, 76%) of **(–)-S3** as yellow oil.

(–)-S3: $[\alpha]^{18}_D$ - 25 (*c* 0.47, $CHCl_3$); IR (neat): 3420 cm^{-1}; ^{1}H-NMR (400 MHz, $CDCl_3$) δ 1.62 (3H, s), 1.70 (3H, s), 1.97 (1H, brs) 2.19 – 2.26 (1H, m), 2.39 – 2.36 (1H, m), 3.49 – 3.55 (2H, m), 3.64 – 3.68 (1H, m), 4.54 (1H, d, *J* = 11.8 Hz), 4.67 (1H, d, *J* = 11.8 Hz), 5.10 – 5.15 (1H, m), 7.27 – 7.38 (5H, m); ^{13}C-NMR (100 MHz, $CDCl_3$) δ 17.8, 25.8, 29.5, 64.2, 71.5, 79.8, 119.4, 127.7, 127.8, 128.5, 134.2, 138.4; HR-MS (ESI) calcd. for $C_{14}H_{20}O_2Na$ (M^+ +Na): 243.1356, found: 243.1355.

Tetrahydropyranochromene (–)-S5

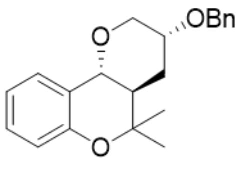

To a solution of salicylaldehyde **S4** (157.5 mg, 1.29 mmol) in benzene (1.0 mL) was added $HC(OMe)_3$ (105.8 mg, 1.85 mmol) and *p*-TsOH (23.4 mg, 0.123 mmol) at ambient temperature. After being stirred for 10 min at room temperature, then **(–)-S3** (1.23 M, 1 mL, 1.23 mmol) was added. After being stirred at the same temperature for 3 h, the reaction was quenched with saturated aqueous $NaHCO_3$ (2 mL) and the mixture was extracted with Et_2O twice. The combined organic layers were washed with brine, dried over anhydrous $MgSO_4$, and concentrated *in vacuo.* Purification of the residue by flash chromatography (Et_2O: Hexane = 1:16) gave 104 mg (0.321 mmol, 26%) of **(–)-S5** as brown oil.

(–)-S5: $[\alpha]^{18}_D$ - 61 (*c* 0.71, $CHCl_3$); IR (neat): 2975 cm^{-1}; ^{1}H-NMR (400 MHz, $CDCl_3$) δ 1.18 (3H, s), 1.36 (3H, s), 1.43 (1H, ddd, *J* = 13.0, 10.1, 2.9 Hz), 2.11 (1H, ddd, *J* = 13.2, 8.7, 2.9 Hz), 2.23 (1H, ddd, *J* = 11.8, 10.1, 2.9 Hz), 3.59 – 3.63 (1H, m), 3.64 (1H, dd, *J* = 12.1, 1.5 Hz), 4.18 (1H, d, *J* = 10.6 Hz), 4.25 (1H, ddd, *J* = 12.1, 8.7, 1.5 Hz), 4.49 (1H, d, *J* = 12.6 Hz), 4.60 (1H, d, *J* = 12.1 Hz), 6.69 (1H, dd, *J* = 8.2, 1.4 Hz), 6.81 (1H, ddd, *J* = 7.7, 7.2, 1.4 Hz), 7.07 (1H, ddd, *J* = 8.7, 6.8, 1.4 Hz), 7.16 – 7.36 (5H, m), 7.42 (1H, d, *J* = 7.7 Hz); ^{13}C-NMR (100 MHz, $CDCl_3$) δ 20.2, 27.3, 29.5, 39.4, 69.4, 70.3, 71.4, 73.6, 78.0, 116.6, 119.8, 122.1, 126.4, 127.52, 127.55, 128.3, 128.8, 138.6, 152.8; HR-MS (ESI) calcd. for $C_{21}H_{24}O_3Na$ (M^+ +Na): 347.1618, found: 347.1600.

Alcohol (–)-#104ox1

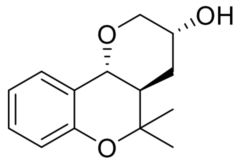

To a solution of **(–)-S5** (49.0 mg, 0.151 mmol) in MeOH (2.0 mL) was added 10% Pd/C (10 mg) under an Ar atmosphere. The flask was evacuated and backfilled with hydrogen. The reaction mixture was stirred at room temperature for 3 h under H_2 atmosphere. Then the mixture was filtered through a Celite pad. The filtrate was concentrated *in vacuo*. Water was added to the residue, and the mixture extracted with EtOAc twice. The combined organic extracts were washed with brine, dried over anhydrous $MgSO_4$, and concentrated *in vacuo*. Purification by flash chromatography (EtOAc : Hexane = 1 : 2) provided 31.5 mg (0.135 mmol, 89%) of **(–)-#104ox1** as white solid.

(–)-#104ox1: $[\alpha]^{20}_D$ - 50 (*c* 0.075, $CHCl_3$); mp 143 °C; IR (neat): 3340 cm^{-1}; ^{1}H-NMR (400 MHz, $CDCl_3$) δ 1.19 (3H, s), 1.38 (3H, s), 1.51 (1H, ddd, *J* = 13.0, 10.1, 2.9 Hz), 2.03 (1H, ddd, *J* = 13.4, 5.3, 2.4 Hz), 2.11 (1H, ddd, *J* = 12.0, 10.6, 3.4 Hz), 2.25 (1H, d, *J* = 7.2 Hz), 3.82 (1H, dd, *J* = 12.3, 1.2 Hz), 3.95 – 4.01(1H, m), 4.09 (1H, ddd, *J* = 12.3, 8.2, 1.5 Hz), 4.23 (1H, d, *J* = 10.7 Hz), 6.78 (1H, dd, *J* = 8.0, 1.0 Hz), 6.90 (1H, ddd, *J* = 7.7, 7.2, 1.0 Hz), 7.17 (1H, ddd *J* = 8.2, 7.2, 1.0 Hz), 7.42 (1H, d, *J* = 7.7 Hz) ; ^{13}C-NMR (100 MHz, $CDCl_3$) δ 20.3, 27.3, 31.9, 39.4, 65.3, 72.7, 74.0, 78.1, 116.9, 119.9, 121.7, 126.0, 129.1, 152.8; HR-MS (ESI) calcd. for $C_4H_{18}O_3Na$ (M^+ +Na): 257.1148, found: 257.1132

Mesylate (–)-S6

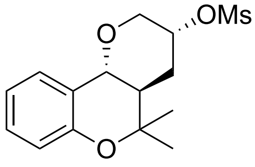

To a solution of **(–)-#104ox1** (11.6 mg, 0.0495 mmol) in a 1:1 mixture of Et_2O and pyridine (2 mL) was added MsCl (56.70 mg, 0.495 mmol) at 0 °C. After being stirred for 10 min, then Et_3N (50.09 mg, 0.495 mmol) was added. After being stirred at 0 °C for 1 h, the reaction was quenched with H_2O (2 mL) and the mixture was extracted with Et_2O twice. The combined organic layers were washed with brine, dried over anhydrous $MgSO_4$, and concentrated *in vacuo.* Purification of the residue by flash chromatography (EtOAc: Hexane = 1: 2) gave 12.5 mg (0.0400 mmol, 81%) of **(–)-S6** as yellow oil.

(–)-S6: $[\alpha]^{19}_D$ – 48.9 (*c* 0.322, $CHCl_3$); IR (neat): 1353 cm^{-1}; ^{1}H-NMR (400 MHz, $CDCl_3$) δ 1.19 (3H, s）, 1.40 (3H, s), 1.67 (1H, ddd, *J* = 13.0, 10.1, 2.9 Hz), 2.22 (1H, ddd, *J* = 12.0, 10.4, 2.9 Hz), 2.33 (1H, ddd, *J* = 14.0, 11.1, 2.9 Hz), 3.06 (3H, s）, 3.85 (1H, dd, *J* = 13.0, 1.2 Hz), 4.27 (1H, d, *J* = 10.6 Hz), 4.34 ~ 4.37 (1H, m), 6.80 (1H, d, *J* = 8.2 Hz), 6.91 (1H, dd, *J* = 8.2, 7.2 Hz), 7.18 (1H, dd, *J* = 8.2, 7.2 Hz), 7.41 (1H, d, *J* = 7.7 Hz); ^{13}C-NMR (100 MHz, $CDCl_3$) δ 20.1,

27.3, 30.7, 39.1, 39.2, 69.8, 73.5, 74.2, 77.6, 116.9, 120.1, 121.2, 126.2, 129.3; HR-MS (ESI) calcd. for $C_{15}H_{20}O_5SNa$ (M^+ +Na): 335.0924, found: 335.0905.

Tetrahydropyranochromene (–)-#104

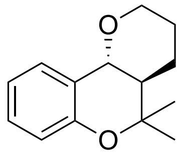

To a solution of **(–)-S6** (9.5 mg, 0.0304 mmol) in THF (2.0 mL) was added $LiBHEt_3$ (1.06 M in THF, 764 μL, 0.810 mmol) at room temperature. After stirred for 6 h under reflux, then resulting mixture was allowed to cool to room temperature and quenched with saturated aqueous NH_4Cl (2 mL). The mixture was extracted with Et_2O twice. The combined organic layer was washed with brine, dried with $MgSO_4$, then concentrated *in vacuo.* Purification of the residue by flash chromatography (EtOAc: Hexane = 1: 40) gave 4.1 mg (0.0188 mmol, 62%) of **(–)-#104** as white solid. Optical purity of the sample was analyzed by chiral HPLC by using DAICEL CHIRALCEL® OD-H column and found to be 99% ee.

(–)-#104: $[\alpha]^{19}_D$ –50 (*c* 0.15, $CHCl_3$); mp 83 °C; IR (neat): 2976 cm^{-1}; ^1H-NMR (400 MHz, $CDCl_3$) δ 1.20 (3H, s), 1.30 – 1.36 (1H, m), 1.39 (3H, s), 1.68 – 1.80 (3H, m), 1.90 – 1.96 (1H, m), 3.65 (1H, ddd, *J* = 11.3, 7.7, 3.4 Hz), 4.13 – 4.18 (1H, m), 4.20 (1H, d, *J* = 10.6 Hz), 6.76 (1H, d, *J* = 8.2 Hz), 6.89 (1H, dd, *J* = 7.7, 7.2 Hz), 7.15 (1H, dd, *J* = 7.2, 7.2 Hz), 7.41 (1H, d, *J* = 7.7 Hz); ^{13}C-NMR (100 MHz, $CDCl_3$) δ 20.4, 25.1, 26.3, 27.6, 45.1, 68.3, 73.6, 77.24, 78.3, 116.7, 119.9, 122.5, 126.0, 128.8; LR-MS (EI) *m/z* 218.1 (M^+) (100%); HR-MS (EI) calcd. for $C_{14}H_{18}O_2$ (M^+): 218.1307, found: 218.1302.

(+)-#104ox1 and (+)-#104

(+)-**#104ox1** and (+)-**#104** were also prepared as described above starting from (–)-**S1.** Optical purity of the sample of (+)-**#104** was analyzed by chiral HPLC by using DAICEL CHIRALCEL® OD-H column and found to be 96% ee.

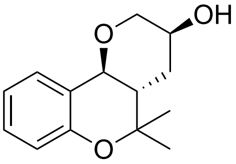

(+)-#104ox1

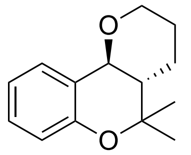

(+)-#104 (96% ee)

(+)-#104ox1: $[\alpha]^{20}_D$ + 47 (*c* 0.050, $CHCl_3$).

(+)-#104: $[\alpha]^{20}_D$ + 47 (*c* 0.040, $CHCl_3$).

Enzymatic oxidation of exemplary substrates, and derivatization of the oxidation products for structural elucidation and optical purity determination

P450 cam-mediated oxidation of #104

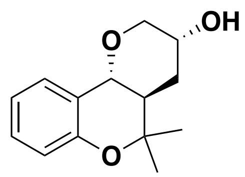

A solution of **#104** (100 mM in DMSO, 500 µL, 50 µmol) was added to the mixture of purified P450cam (30.9 µM in potassium phosphate buffer, 809 µL, 25 nmol), purified Pdx (176 µM in potassium phosphate buffer, 227 µL, 40 nmol), purified PdR (456 µM in potassium phosphate buffer, 55.8 µL, 25 nmol) and NADH (100 mM in MQ, 1.00 mL, 100 µmol). The resultant mixture was incubated at 25 °C for 12 h with horizontal shaking at 110 rpm. The mixture was extracted with AcOEt (15 mL × 3), dried over Na_2SO_4 and concentrated under reduced pressure. The residue was purified by flash silica gel column chromatography (AcOEt / Hexane = 1 / 6) to give (–)-**#104ox1** (3.52 mg, 15.0 µmol, 30%, 48% brsm, 95% ee) and recovered (+)-**#104** (4.1 mg, 18.9 µmol, 38%, 78% ee). Optical purity of **#104ox1** was determined after removal of the hydroxyl group as described below.

Synthesis of methyl xanthate (–)-S7

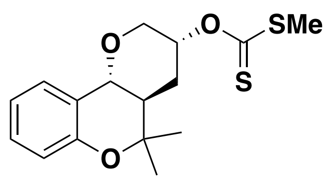

To a flame dried flask was added NaH (60%, 6.00 mg, 145 µmol) and THF (0.5 mL). The mixture was cooled to 0 °C and a solution of (–)-**#104ox1** (1.76 mg, 7.26 µmol) in THF (2 mL) was added dropwise. The reaction mixture was stirred for 5 min at 0 °C, then allowed to warm to room temperature over 1 h. CS_2 (10.0 µL, 145 µmol) was added to the reaction mixture at the same temperature and stirring was continued for an another 1 h. Then, MeI (20.0 µL, 321 µmol) was added to the mixture and stirring was continued for an additional 30 min. The reaction mixture was partitioned between 0.1 N HCl (1 mL) and Et_2O (3 mL), and the aqueous layer was extracted with Et_2O (10 mL × 3) and washed with brine (10 mL). The combined organic extracts were dried over Na_2SO_4 and concentrated under reduced pressure. The residue was purified by flash silica gel column chromatography (AcOEt / Hexane = 1 / 40) to give xanthate (–)-**S7** (2.31 mg, 7.11 µmol, 98%). **S7:** $[\alpha]_D^{29}$ –33 (*c* 0.032, $CHCl_3$); IR (neat): 1731 cm^{-1}; ^{1}H-NMR (400 MHz, $CDCl_3$): δ 7.43 (1H, d, *J* = 7.7 Hz), 7.19 (1H, t, *J* = 7.5 Hz), 6.92 (1H, t, *J* = 7.5 Hz), 6.79 (1H, d, *J* = 7.7 Hz), 5.83 (1H, brs), 4.40 (1H, d, *J* = 13.0 Hz), 4.29 (1H, d, *J* = 10.6 Hz), 3.88 (1H, d, *J* = 13.0 Hz), 2.54 (3H, s), 2.32-2.20 (1H, m), 2.19-2.13 (1H, m), 1.66 (1H, td, *J* = 13.5, 2.9 Hz), 1.39 (3H, s), 1.21 (3H, s); ^{13}C-NMR (100 MHz, $CDCl_3$): δ 218.7, 129.2, 126.3, 123.5, 120.1, 116.9, 89.7, 77.2, 76.6, 73.4, 69.2, 64.4, 40.1, 29.2, 27.4, 20.0; LR-MS (EI) *m/z*: 324 (M^+), HR-MS (EI) *m/z*: [M^+] calcd for $C_{16}H_{20}O_3S_2$ 324.0854, Found 324.0860.

Barton-McCombie deoxygenation of S7. Preparation of (–)-#104

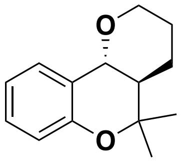

A solution of **S7** (2.10 mg, 6.48 µmmol), AIBN (1.00 mg, 6.09 µmmol) and Bu_3SnH (10.0 µL, 37.2 µmmol) in toluene (2.5 mL) was heated at 130 °C for 10 min. After allowed to cool to room temperature, the resulting solution was concentrated under reduced pressure. The residue was purified by flash silica gel column chromatography (toluene / Hexane = 1 / 8) to give (–)-**#104** (1.06 mg, 4.86 µmol, 75%). Optical purity of (–)-**#104ox1** was determined by Chiral HPLC analysis (Column: DAICEL Chiralcel® OD-H, ϕ 4.6 mm i.d. x 25 mm; Eluent: Hexane / *i*-PrOH = 99 / 1; Flow rate: 0.5 mL/min; Retention time: 10.7 min for (+)-enantiomer, 12.1 min for (–)-enantiomer) (See Supporting Figure 7).

P450 BM3 (F87A)-mediated oxidation of #103

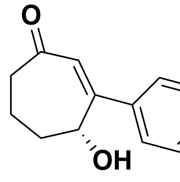

\+

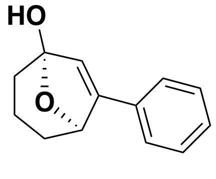

#103ox1 **#103ox2**

P450 (F87A)-expressing *E. coli* lysate was prepared using the pellet of P450 (F87A)-expressing *E. coli* (from 1.8 L culture) and 14 mL of reaction buffer (100 mM potassium phosphate, 5% glycerol, 30 mg/L kanamycin, 250 μM NADPH, pH 7.4) as described in the Experimental section. **#103** (100 mM in DMSO, 450 μL, 45 μmol) was incubated at 25 °C for 48 h with horizontal shaking at 110 rpm. After the incubation, the reaction mixture was extracted with AcOEt (20 mL × 3), dried over Na_2SO_4 and concentrated under reduced pressure. The residue was purified by flash silica gel column chromatography (AcOEt / Hexane = 1 / 6 to 1 / 3) to give an equilibrium mixture of **#103ox1** and **#103ox1** (2.36 mg, 22.8 μmol, 51%, 54% brsm).

Mixture of #103ox1 and #103ox2: IR (neat): 3372, 1656 cm^{-1}; ^{1}H-NMR (400 MHz, $CDCl_3$, selected signals): 6.28 (1H, s, major isomer), 6.19 (0.4H, s, minor isomer), 5.23 (1H, d, *J* = 2.7 Hz, major isomer), 5.00 (0.4H, dd, *J* = 10.2, 4.1 Hz, minor isomer); LR-MS (EI) *m/z* : 202 (M^+), 173 (100%), HR-MS (EI) *m/z* : [M^+] calcd for $C_{13}H_{14}O_2$ 202.0994, Found 202.0978.

Acetylation of an equilibrium mixture of #103ox1 and #103ox1

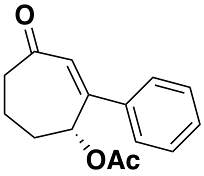

A solution of an equilibrium mixture of **#103ox1** and **#103ox1** (1.00 mg, 4.95 μmol), Ac_2O (1 μL, 10.6 μmol) and DMAP (1.00 mg, 8.19 μmol) in dry pyridine (0.2 mL) was stirred at room temperature for 10 min. The reaction was quenched with saturated aqueous NH_4Cl (0.2 mL) and MeOH (0.2 mL), and the resulting mixture was extracted with Et_2O (10 mL × 3). The combined organic layers were dried over Na_2SO_4, washed with brine (10 mL) and concentrated under reduced pressure. The residue was purified by silica gel column chromatography (AcOEt / Hexane = 1 / 8 to 1 / 6) to give acetate (–)-**S8** (0.89 mg, 3.65 μmol, 74%, 78% brsm).

(–)-**S8**: $[\alpha]_D^{28}$ –11 (*c* 0.053, $CHCl_3$); IR (ATR): 1739 cm^{-1}; ^{1}H-NMR (400 MHz, $CDCl_3$): δ 7.44(2H, dd, *J* = 7.3, 2.0 Hz), 7.36(3H, m), 6.23(1H, s), 6.00(1H, t, *J* = 4.6 Hz), 2.91-2.64(1H, m), 2.64-2.58(1H, m), 2.18-2.12(2H, m) 1.99-1.95(5H, m); ^{13}C-NMR (150 MHz. $CDCl_3$): δ 204.7, 169.8, 151.9, 139.1, 130.6, 129.2, 128.6, 126.9, 73.1, 41.4, 30.0, 20.9, 18.4; LR-MS (EI) *m/z* : 244 (M^+), 202 (100%), HR-MS (EI) *m/z* : [M^+] calcd for $C_{15}H_{16}O_3$ 244.1099, Found 244.1090.

Synthesis of (*S*)-Mosher ester S9

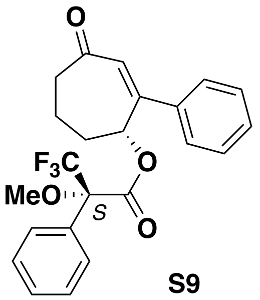

S9

A solution of a mixture of alcohol **#103ox1** and **#103ox2** (1.20 mg, 5.93 μmol), (*S*)-MTPA acid (6.95 mg, 29.6 μmol), 4-dimethylaminopyridine (0.72 mg, 5.93 μmol) and dicyclohexylcarbodiimide (7.34 mg, 35.6 μmol) in CH_2Cl_2 (0.2 mL) was stirred at room temperature for 6 h. The solution was concentrated under reduced pressure. The residue was purified by silica gel column chromatography (AcOEt / Hexane = 1 / 4) to give (*S*)-Mosher ester **S9** (1.30 mg, 3.10 μmol, 52%).

S9: ^{1}H-NMR (400 MHz, $CDCl_3$): δ 7.44-7.41 (2H, m), 7.36-7.34 (3H, m), 7.32-7.25 (5H, m), 6.30 (1H, t, *J* = 4.6 Hz), 6.24 (1H, s), 3.34 (3H, s), 2.75 (1H, dq, *J* = 17.9, 4.0 Hz), 2.56 (1H, dq, *J* = 16.9, 3.4 Hz), 2.28-2.23 (2H, m), 2.02-1.85 (2H, m).

Synthesis of (*R*)-Mosher ester S10

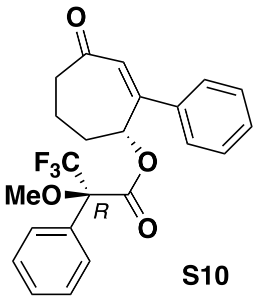

S10

(*R*)-Mosher ester **S10** was synthesized in the manner described above, except that (*R*)-MTPA acid was used (**S10**: 1.62 mg, 3.87 μmol, 65%).

S10: ^{1}H-NMR (400 MHz, $CDCl_3$): δ 7.49-7.47 (2H, m), 7.41-7.39 (3H, m), 7.31-7.22 (5H, m), 6.35 (1H, t, *J* = 3.4 Hz), 6.30 (1H, s), 3.27 (3H, s), 2.64 (1H, dq, *J* = 19.7, 4.8 Hz), 2.53 (1H, dq, *J* = 16.9, 3.4 Hz), 2.17-2,12 (2H, m), 1.91-1.75 (2H, m).

P450 BM3 (F87A)-mediated oxidation of (+)-**#170**

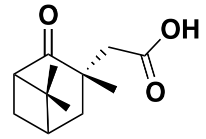

P450 (F87A)-expressing *E. coli* lysate was prepared using the pellet of P450 (F87A)-expressing *E. coli* (from 0.45 L culture) and 5 mL of reaction buffer (100 mM potassium phosphate, 5% glycerol, 30 mg/L kanamycin, 250 μM NADPH, pH 7.4) as described in the Experimental section. Compound (+)-**#170** (100 mM in DMSO, 270 μL, 27 μmol) was added to the lysate, and the resultant mixture was incubated at 25 °C for 22 h with horizontal shaking at 110 rpm. The mixture was extracted with AcOEt (15 mL × 3), dried over Na_2SO_4 and concentrated under reduced pressure. The residue was purified by flash silica gel column chromatography (AcOEt / Hexane = 1 / 2) to give (+)-**#170ox1** (3.75 mg, 17.8 μmol, 66%).

(+)-**#170ox1:** $[\alpha]_D^{28}$ +32 (*c* 0.24, $CHCl_3$); IR (neat): 3180 (br), 1704 cm^{-1}; ^{1}H-NMR (600 MHz, $CDCl_3$): δ 2.98 (1H, d, *J* = 15.1 Hz), 2.72 (1H, t, *J* = 5.1 Hz), 2.62 (1H, s), 2.55-2.50 (1H, m), 2.45 (1H, d, *J* = 15.1 Hz), 2.31-2.28 (1H, m), 2.10 (1H, dd, *J* = 14.1, 4.1 Hz), 1.98 (1H, d, *J* = 10.1 Hz), 1.52 (3H, s), 1.37 (3H, s), 0.82 (3H, s); ^{13}C-NMR (150 MHz, $CDCl_3$): δ 173.6, 58.5, 44.9, 44.3, 42.5, 41.6, 40.9, 36.6, 29.3, 26.3, 25.4, 22.8; HR-MS (ESI) $[M+H]^+$ calcd. for $C_{12}H_{19}O_3$ 211.1322, found 211.1329, $[M-H]^-$ calcd. for $C_{12}H_{17}O_3$ 209.1178, found 209.1172.

Synthesis of Methyl ester (+)-**S11**

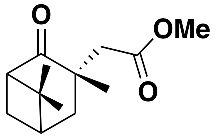

(+)-**#170ox1** (1.93 mg, 9.2 μmol) was dissolved in a 3:1 mixture of benzene/methanol (total 4 mL) under N_2 atmosphere at room temperature. A 2.0 M hexane solution of trimethylsilyldiazomethane (110 μL, 0.22 mmol) was added dropwise via a syringe. After the evolution of gas bubbles ceased, the solution was stirred for another 15 min and then concentrated in vacuo. The residue was purified by flash silica gel chromatography to give methyl ester (+)-**S11** (1.77 mg, 7.90 μmol, 86%).

(+)-**S11**: $[\alpha]_D^{18}$ +32 (*c* 0.089, $CHCl_3$); IR : 1739, 1706 cm^{-1}; ^{1}H-NMR (400 MHz, $CDCl_3$): δ 3.70 (3H, s), 2.91 (1H,d, *J* = 15.4 Hz), 2.66 (1H, t, *J* = 5.4 Hz), 2.53 (1H, quint, *J* = 5.4 Hz), 2.47 (1H, d, *J* = 15.4 Hz), 2.31-2.28 (1H, m), 2.12 (1H, t, *J* = 14.1 Hz), 2.08 (1H, dd, *J* = 14.1, 3.9 Hz), 1.91 (1H, d, *J* = 10.7 Hz), 1.48 (3H, s), 1.36 (3H, s), 0.83 (3H, s); ^{13}C-NMR (150 MHz. $CDCl_3$): δ 216.7, 172.5, 58.5, 51.4, 43.3, 43.1, 42.8, 41.8, 35.3, 30.1, 26.4, 25.7, 22.9; LR-MS (EI) *m/z* : 224 (M^+), HR-MS (EI) *m/z* : [M^+] calcd. for $C_{13}H_{20}O_3$ 224.1412, Found 224.1419.

P450 BM3 (F87A)- and P450 (F87A/A330W)-mediated oxidation of #104

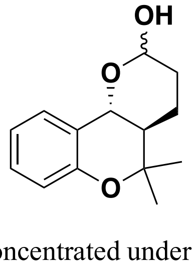

P450 (F87A)-expressing *E. coli* lysate was prepared using the pellet of P450 (F87A)-expressing *E. coli* (from 1.35 L culture) and 10 mL of reaction buffer (100 mM potassium phosphate, 5% glycerol, 30 mg/L kanamycin, 250 μM NADPH, pH 7.4) as described in the Experimental section. **#104** (25.0 mg in 270 μL of DMSO, 115 μmol) was added to the lysate, and the resulting mixture was incubated at 25 °C, 110 rpm for 24 h. The mixture was extracted with AcOEt (20 mL × 3), dried over Na_2SO_4 and concentrated under reduced pressure. The residue was purified by flash silica gel column chromatography (AcOEt / Hexane = 1 / 6) to give

an epimeric mixture of **#104ox2** (12.2 mg, 52.2 μmol, 46%) and recovered **#104** (5.3 mg, 24.4 μmol, 21%). P450 (F87A/A330W)-mediated oxidation was performed as described above to give **#104ox2** (38%). The hemiacetal structure was confirmed by leading to dimethyl acetal **S9** and starting material **#104** as described below.

#104ox2: IR (neat): 3410 (br), 1724 cm^{-1}; ^{1}H-NMR (400 MHz, $CDCl_3$): δ 7.45 (1H, dd, *J* = 8.2, 2.4 Hz), 7.36 (1H, dt, *J* = 7.7, 2.4 Hz), 7.19-7.14 (2H, m), 6.93-6.87 (2H, m), 6.78 (2H, d, *J* = 8.2 Hz), 5.5 (1H, s), 5.03-4.99 (1H, m), 4.95 (1H, d, *J* = 9.2 Hz), 4.72-4.70 (1H, m), 4.34 (1H, d, *J* = 10.6 Hz), 4.31-4.22 (1H, m), 2.85 (1H, d, *J* = 7.2 Hz), 2.54 (1H, t, *J* = 2.4 Hz), 2.14-2.09 (1H, m), 1.99-1.92 (2H, m), 1.77-1.68 (5H, m), 1.41 (3H, s), 1.25 (3H, s), 1.22 (3H, s), 1.19 (3H, s); ^{13}C-NMR (150 MHz, $CDCl_3$): δ 153.1, 152.8, 129.4, 129.0, 128.7, 125.9, 125.5, 122.4, 121.6, 120.0, 119.8, 116.8, 96.5, 91.9, 78.3, 71.4, 64.3, 44.8, 44.0, 33.3, 30.0, 29.7, 27.8, 27.5, 24.0, 20.5, 20.4, 19.5; LR-MS (EI) *m/z* : 234 (M^+), HR-MS (EI) *m/z* : [M^+] calcd for $C_{14}H_{18}O_3$ 234.1256, found 234.1254.

Synthesis of dimethyl acetal S12

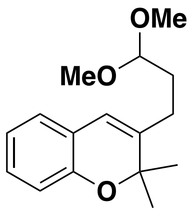

A solution of an epimeric mixture of **#104ox2** (5.00 mg, 21.3 μmol) and *p*-toluenesulfonic acid monohydrate (3.71 mg, 21.6 μmol) and trimethyl orthoformate (11.7 μL, 107 μmol) in MeOH (0.2 mL) was heated at reflux for 3 h. After cooling to room temperature, the resulting solution was concentrated under reduced pressure, extracted with ether (10 mL × 3), washed with brine (10 mL). The combined extracts were dried over Na_2SO_4 and concentrated under reduced pressure. The residue was purified by flash silica gel column chromatography (AcOEt / Hexane = 1 / 15) to give dimethyl acetal **S12** (0.49 mg, 1.9 μmol, 9%).

Dimethyl acetal **S12:** IR (neat): 1734 cm^{-1}; ^{1}H-NMR (400 MHz, $CDCl_3$): δ 7.06 (1H, td, *J* = 7.3, 1.2 Hz), 6.94 (1H, dd, *J* = 7.3, 1.2 Hz), 6.83 (1H, td, *J* = 7.3, 1.2 Hz), 6.77 (1H, d, *J* = 7.3 Hz), 6.10 (1H, s), 4.45 (1H, t, *J* = 5.6 Hz), 3.36 (6H, s), 2.16 (2H, td, *J* = 8.2, 1.4 Hz), 1.90-1.85 (2H, m), 1.42 (3H, s), 1.26 (3H, s); ^{13}C-NMR (150 MHz. $CDCl_3$): δ 146.7, 128.2, 125.6, 121.5, 120.8, 117.6, 116.0, 109.6, 104.1, 89.2, 52.9, 30.4, 29.7, 26.0, 25.8; LR-MS (EI) *m/z* : 262 (M^+), 215 (100%), HR-MS (EI) *m/z* : [M^+] calcd for $C_{16}H_{22}O_3$ 262.1569, Found 262.1570.

BF_3-mediated silane reduction of #104ox2

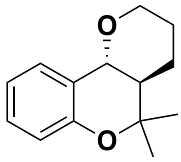

To a solution mixture of an epimeric mixture of **#104ox2** (oxidized by F87A or F87A/A330W) (1.50 mg, 6.40 μmol) and Et_3SiH (3.06 μL, 19.2 μmol) in CH_2Cl_2 (150 μL) was added dropwise $BF_3 \cdot OEt_2$ (2.4 μL, 19.2 μmol) at −78 °C. The reaction was stirred at −78 °C for 20 min and then at 0 °C for 3 h. The reaction was quenched with sat. aq. $NaHCO_3$ (0.2 mL) and extracted with AcOEt (10 mL × 3). The combined organic layers were washed with brine (10 mL), dried over Na_2SO_4 and concentrated under reduced pressure. The residue was purified by flash silica gel column chromatography (AcOEt / Hexane = 1 / 20) to give **#104** (1.34 mg, 6.14 μmol, 96%). Optical purity of **#104ox1** was determined by Chiral HPLC analysis.

Structure estimation of oxidized products by GC/MS

Each hit compound (20 μM) was incubated with P450 BM3(WT) or P450 BM3(F87A) (0.75 ~ 3.75 μM) in reaction buffer (100 mM KPi, 230 μM NADPH, 5% glycerol, pH 7.4, 5 mL) at 25 °C with shaking at 110 rpm. After the incubation for 0 min, 1 min, 10 min, 2 h, and 12 h, the reaction mixture was extracted with AcOEt (500 μL × 3). For the reaction of the carboxylic acid substrate, 0.1 *N* HCl (50 μL) was added to the reaction mixture before extraction. The combined organic layers were dried down *in vacuo*. Pyridine (20 μL) and *N*-methyl-*N*-(trimethylsilyl)trifluoroacetamide (MSTFA) (30 μL) were added to the residue, and the resulting mixture was incubated at 60 °C for 1 h. The reaction mixture was diluted with CH_3CN (50 μL), and analyzed by GC/MS using JMS-T100GCV time-of-flight mass spectrometer (JEOL, Tokyo, Japan) equipped with 7890A GC (Agilent Technologies, Santa Clara, CA, USA) and 7693 autosampler (Agilent) as follows: Five μL of each sample was injected with splitless mode at 250 ºC and analyzed by using HP-5 19091J-413 column (30 m × 0.25 mm id, film thickness 0.25 μm; Agilent Technologies). The carrier gas was helium at a constant flow-rate of 1.5 ml/min. The GC oven temperature was kept at 70 ºC for 4 min, ramped linearly from 70 ºC to 325 ºC at 30 ºC/min, and kept at 325 ºC for 7.5 min. The outlet of the column is directly connected to an electron ionization (EI, 70 eV) / field ionization (FI) combination source of the mass spectrometer and both EI and FI mode spectra were recorded in separate runs. Typical full width at half maximum mass resolution was around 8,000 and typical mass measurement accuracy was 5 mDa after application of single-point drift compensation with a column background peak at *m/z* 207 in EI mode.

The GC/MS data analysis was carried out in the following steps. First, the peaks that would be attributable to the mono-oxidized products, i.e., peaks of hydroxyl derivatives (detected as TMS-ether or TMS-ester) for each substrate were identified by comparing accurate mass chromatograms of the time-course samples. Typically, relative intensity of the peaks corresponding to the oxidized products increased with incubation time; some of the peaks are then decreased due to further oxidation and/or decomposition. Peaks stay the same in the time course were regarded as background and ignored. Both the EI and FI mass spectra of each selected peak were manually examined to confirm presence of M-15 (in EI) and/or $M^{+\bullet}$ (in FI, data not shown) corresponding to the TMS-derivatives of mono-oxidized products. The assignment of hydroxyl positions (Supporting Figures 16 to 20) was carried out based on the general trend that TMS-ethers show characteristic fragmentation at both side of the TMS-O-group both in EI and FI mass spectra.[6]

References:

(1) (a) Takayama, H.; Takahashi, S.; Moriya, T.; Osada, H.; Iwabuchi, Y.; Kanoh, N. *Chembiochem* **2011**, *12*, 2748; (b) Moriya, T.; Kawamata, A.; Takahashi, Y.; Iwabuchi, Y.; Kanoh, N. *Chem. Commun.* **2013**, *49*, 11500; (c) Takahashi, S.; Nagano, S.; Nogawa, T.; Kanoh, N.; Uramoto, M.; Kawatani, M.; Shimizu, T.; Miyazawa, T.; Shiro, Y.; Osada, H. *J. Biol. Chem.* **2014**, *289*, 32446.

(2) Kille, S.; Zilly, F. E.; Acevedo, J. P.; Reetz, M. T. *Nat. Chem.* **2011**, *3*, 738.

(3) Omura, T.; Sato, R. *J. Biol. Chem.* **1964**, *239*, 2370.

(4) Scheid, G.; Ruijter, E.; Konarzycka-Bessler, M.; Bornscheuer, U. T.; Wessjohann, L. A. *Tetrahedron Asymm.* **2004**, *15*, 2861.

(5) Cardillo, G.; Orena, M.; Romero, M.; Sandri, S. *Tetrahedron* **1989**, *45*, 1501.

(6) Furuhashi, T.; Ishii, K.; Tanaka, K.; Weckwerth, W.; Nakamura, T. *Rapid Commun. Mass Spectrom.* **2015**, *29*, 238.

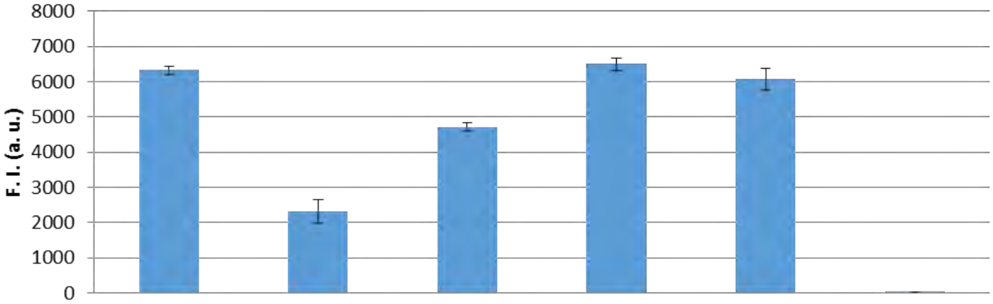

P450cam (nM)	-	2000	200	20	2	-
Pdx (nM)	-	8000	800	80	8	-
PdR (nM)	-	2000	200	20	2	-
Probe (5 µM)	+	+	+	+	+	+
H_2O_2(100 µM)	+	+	+	+	+	-
NAD^+(50 µM)	+	+	+	+	+	+

Supporting Figure 1 Effect of the concentration of P450, and its electron transfer proteins (putidaredoxin and putidaredoxin reductase) on the detection of H_2O_2 using Peroxyfluor-1 (PF-1). H_2O_2 solutions (100 µM) containing P450cam (0–2000 nM), Pdx (0–8000 nM), PdR (0–2000 nM), NAD^+ (50 µM), and PF-1 (5 µM) were incubated at 25 ° C for 30 min. The fluorescence was read as described in the Materials and Methods section.

a)

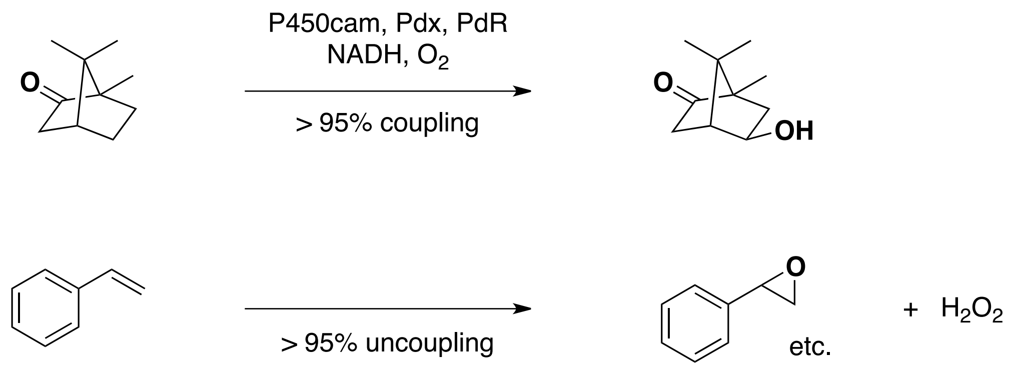

b)

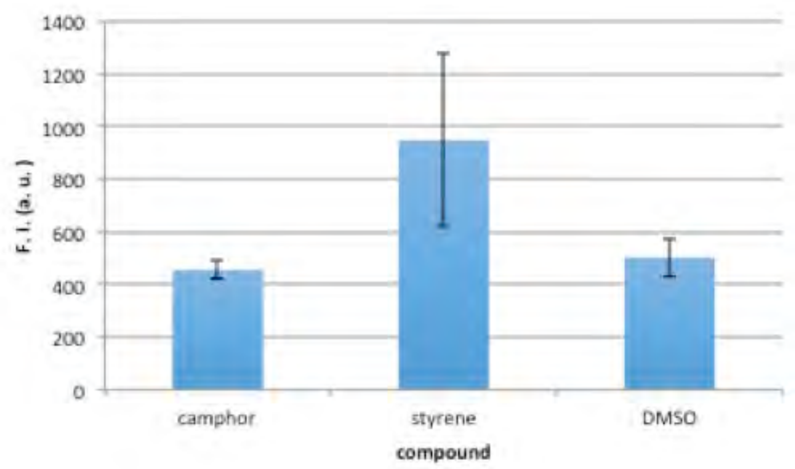

Supporting Figure 2 Detection of H_2O_2 produced by uncoupled turnover of P450 cam using Peroxyfluor-1. (a) Oxidation of styrene is highly uncoupled relative to the oxidation of camphor. See, J. A. Fruetel *et al. J. Am. Chem. Soc.* **1992**, *114*, 6987. (b) Detection of H_2O_2 production. Compound (camphor or styrene, 200 μM) was incubated with P450 cam (100 nM), Pdx (200 nM), PdR (100 nM), PF-1 (60 μM) and NADH (50 μM) in potassium phosphate buffer (50 mM, pH 7.4, 50 μL) at 25 ° C for 30 min. The fluorescence was read as described in the Materials and Methods section (Mean of 3 replicates, error bars show standard deviation.)

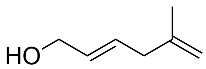

1

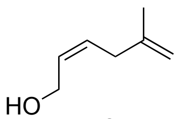

2

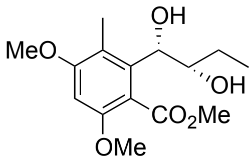

3

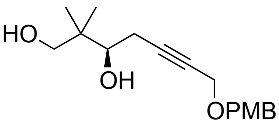

4

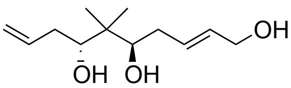

5

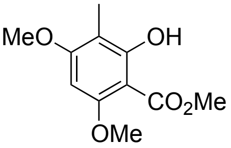

6

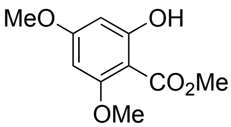

7

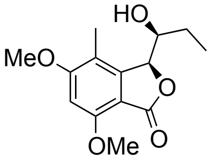

8

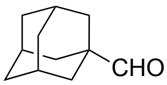

9

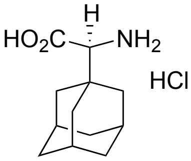

10

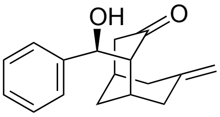

11

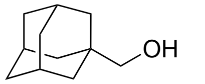

12

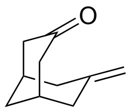

13

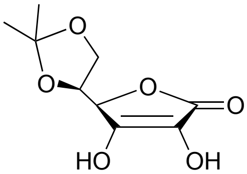

14

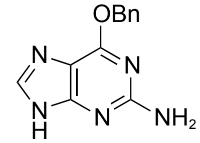

15

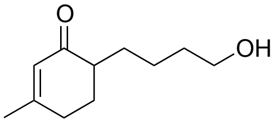

16

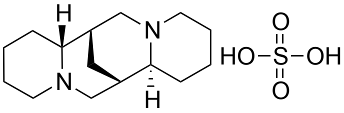

17

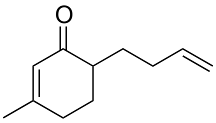

18

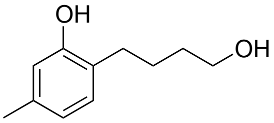

19

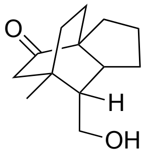

20

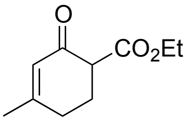

21

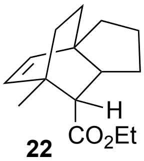

22

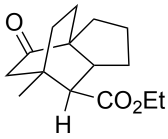

23

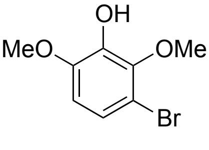

24

Supporting Figure 3-1 Structure of in-house library compound **1** - **24**

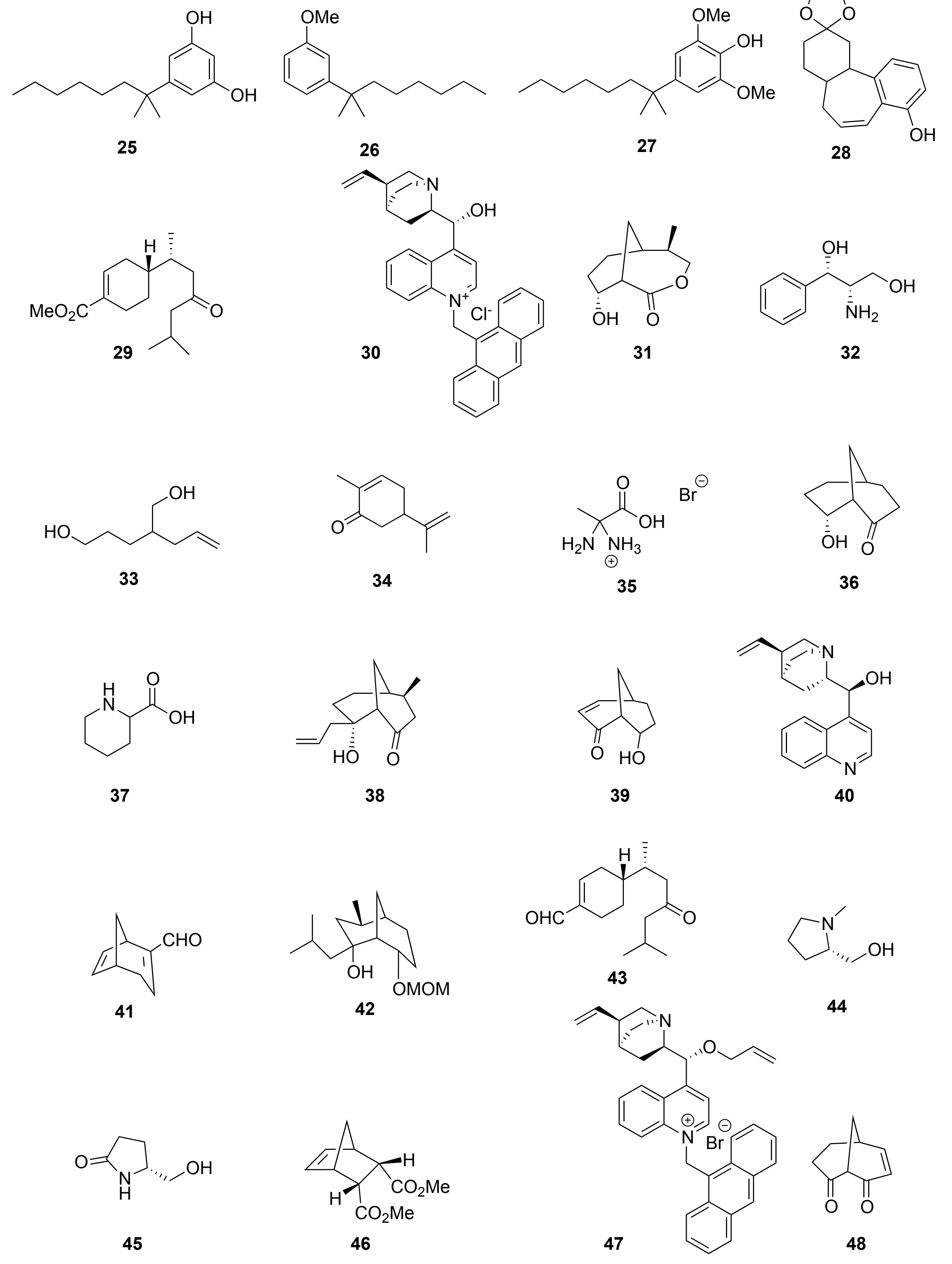

Supporting Figure 3-2 Structure of in-house library compound **25** - **48**

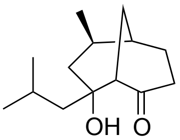
49

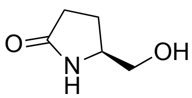
50

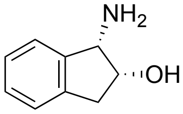
51

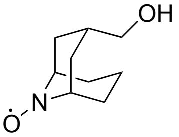
52

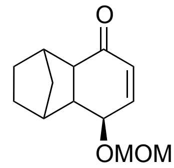
53

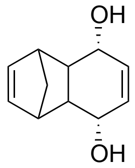
54

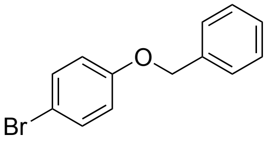
55

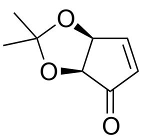
56

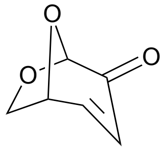
57

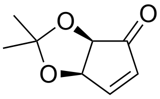
58

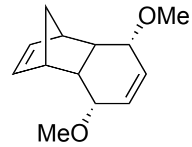
59

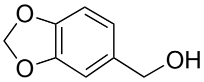
60

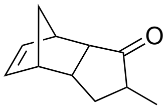
61

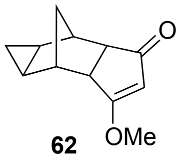
62

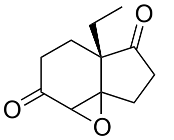
63

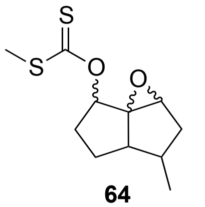
64

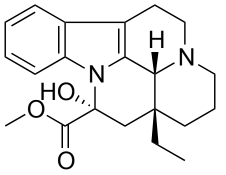
65

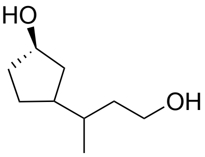
66

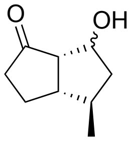
67

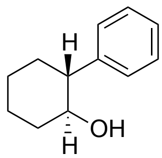
68

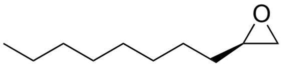
69

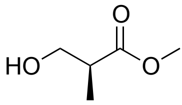
70

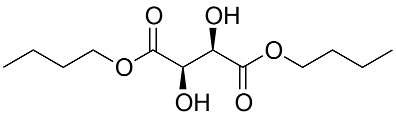
71

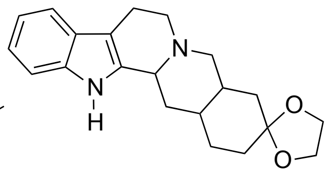
72

Supporting Figure 3-3 Structure of in-house library compound **49** - **72**

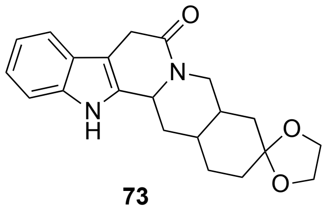

73

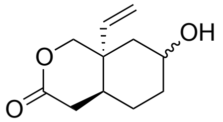

74

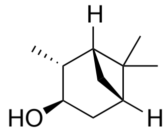

75

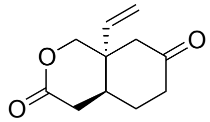

76

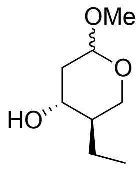

77

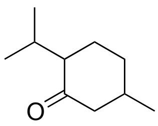

78

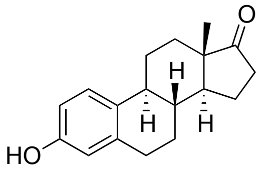

79

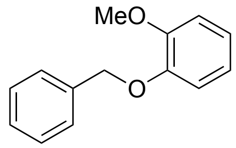

80

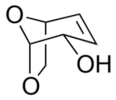

81

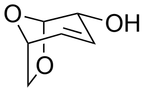

82

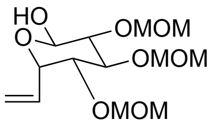

83

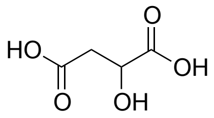

84

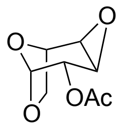

85

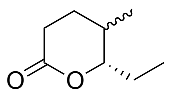

86

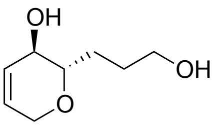

87

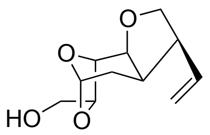

88

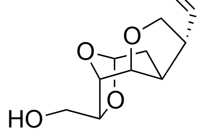

89

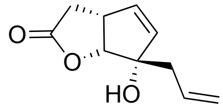

90

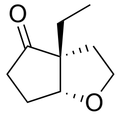

91

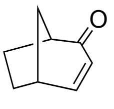

92

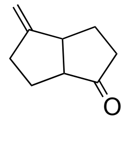

93

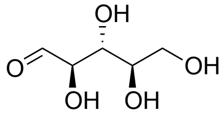

94

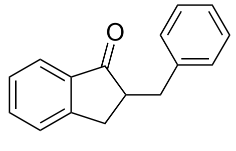

95

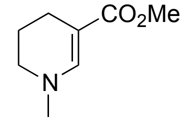

96

Supporting Figure 3-4 Structure of in-house library compound **73** - **96**

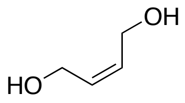

97

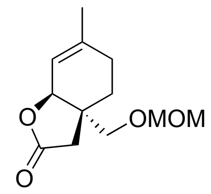

98

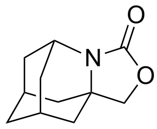

99

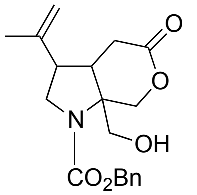

100

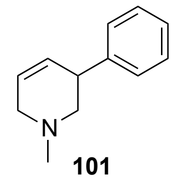

101

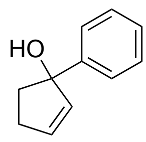

102

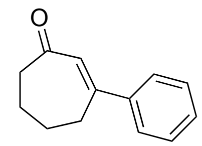

103

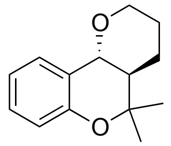

104

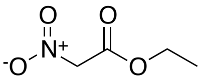

105

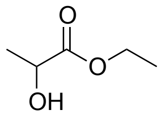

106

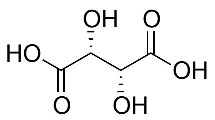

107

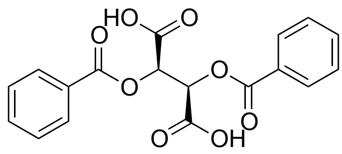

108

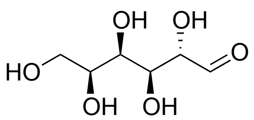

109

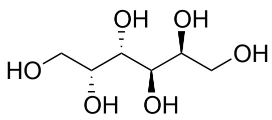

110

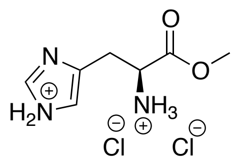

111

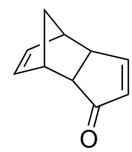

112

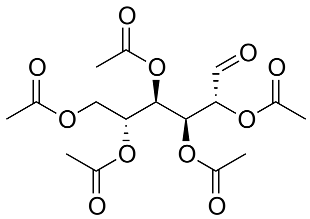

113

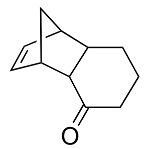

114

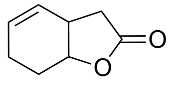

115

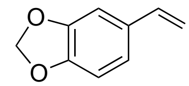

116

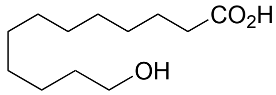

117

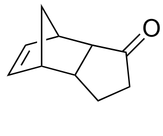

118

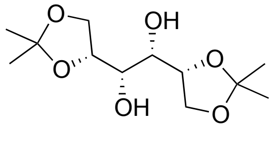

119

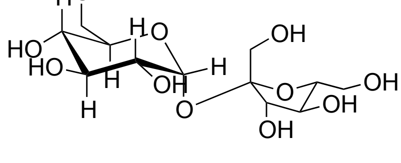

120

Supporting Figure 3-5 Structure of in-house library compound **97** - **120**

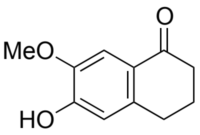

121

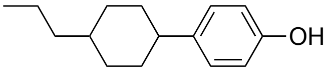

122

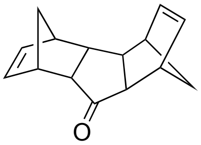

123

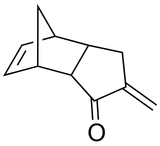

124

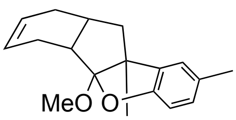

125

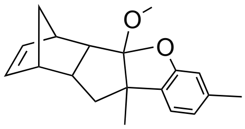

126

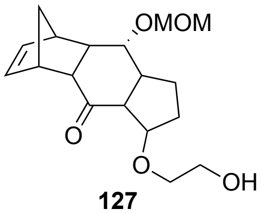

127

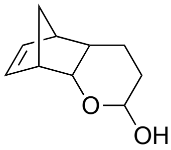

128

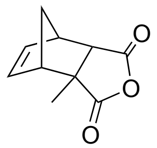

129

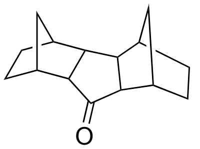

130

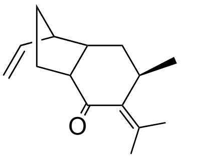

131

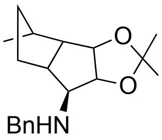

132

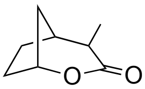

133

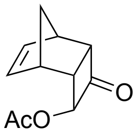

134

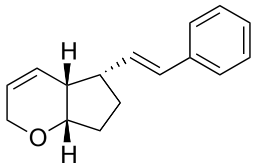

135

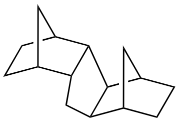

136

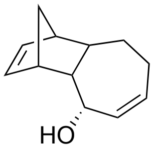

137

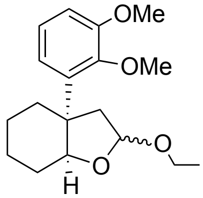

138

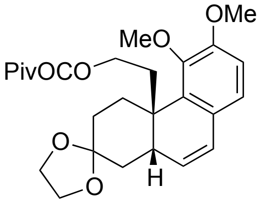

139

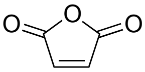

140

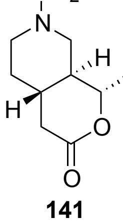

141

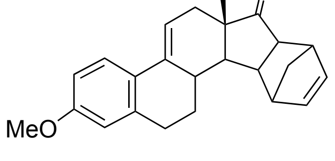

142

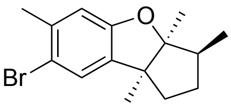

143

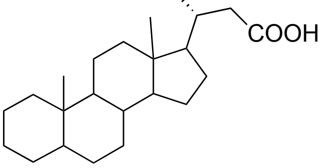

144

Supporting Figure 3-6 Structure of in-house library compound **121** - **144**

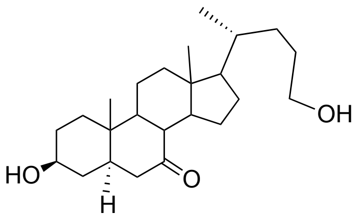

145

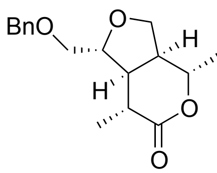

146

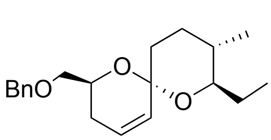

147

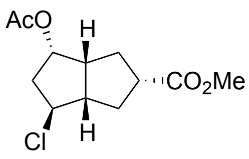

148

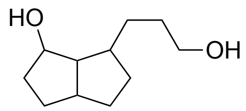

149

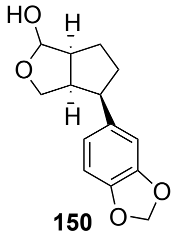

150

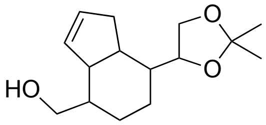

151

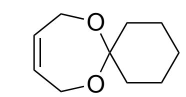

152

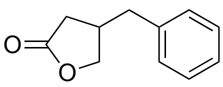

153

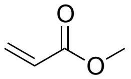

154

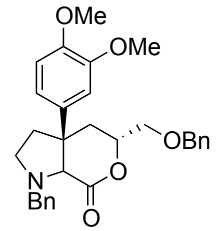

155

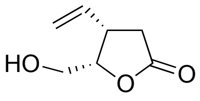

156

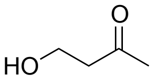

157

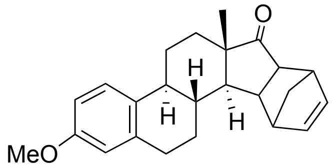

158

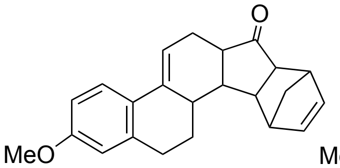

159

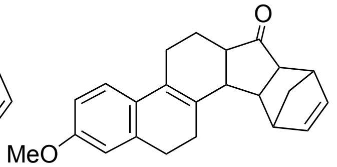

160

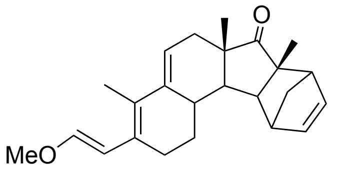

161

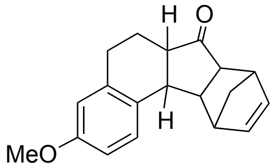

162

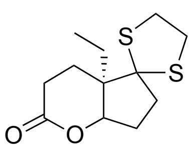

163

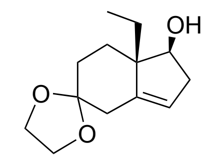

164

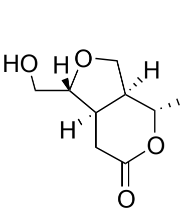

165

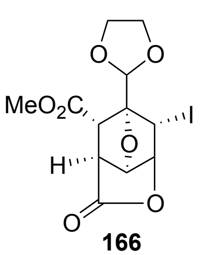

166

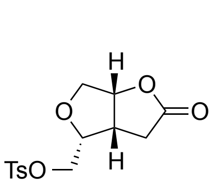

167

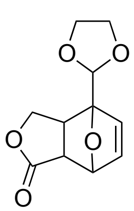

168

Supporting Figure 3-7 Structure of in-house library compound **145** - **168**

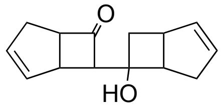

169

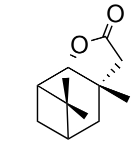

170

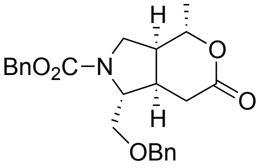

171

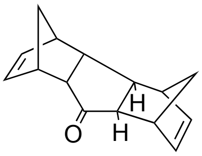

172

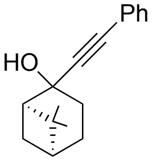

173

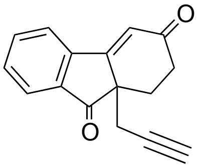

174

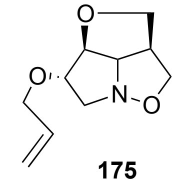

175

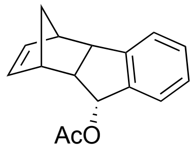

176

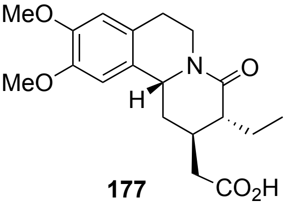

177

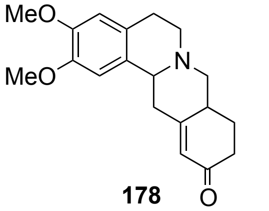

178

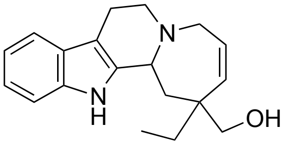

179

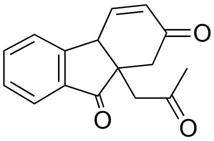

180

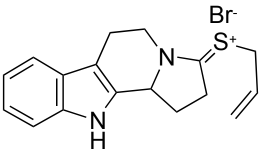

181

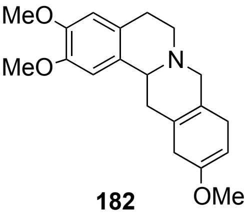

182

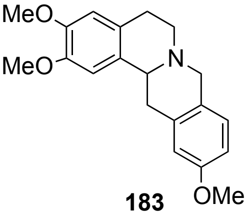

183

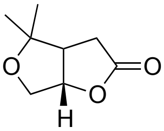

184

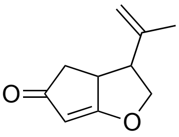

185

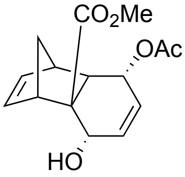

186

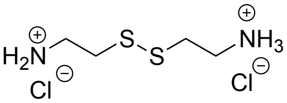

187

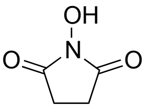

188

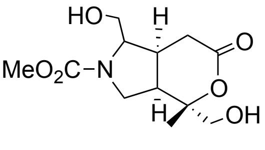

189

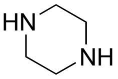

190

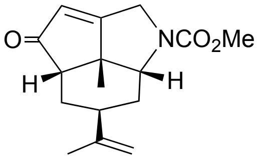

191

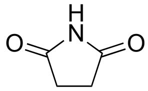

192

Supporting Figure 3-8 Structure of in-house library compound **169** - **192**

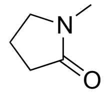

193

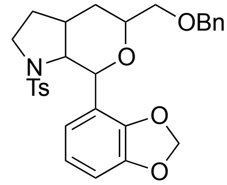

194

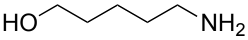

195

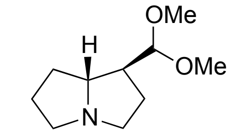

196

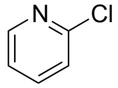

197

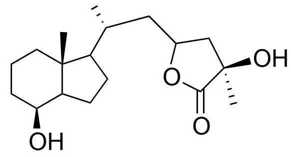

198

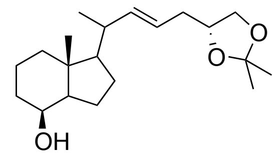

199

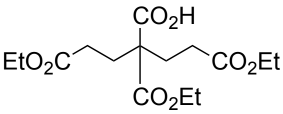

200

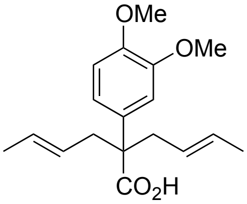

201

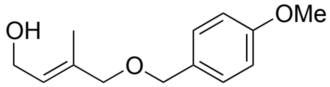

202

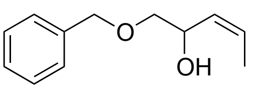

203

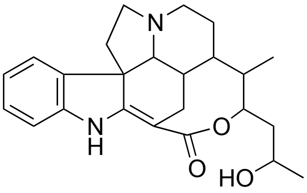

204

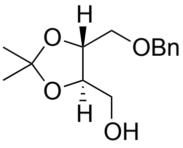

205

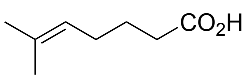

206

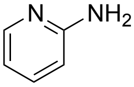

207

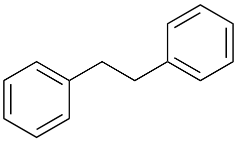

208

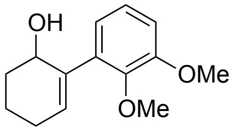

209

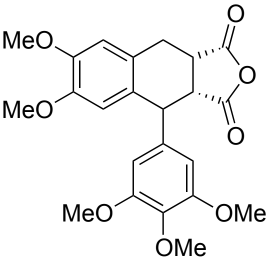

210

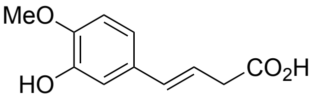

211

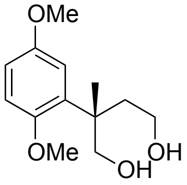

212

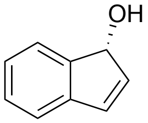

213

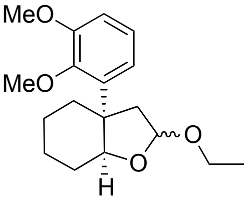

214

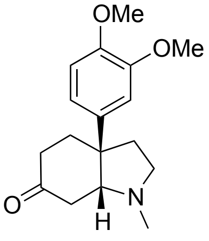

215

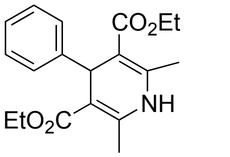

216

Supporting Figure 3-9 Structure of in-house library compound **193** - **216**

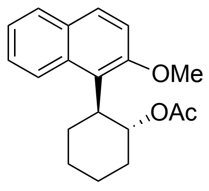

217

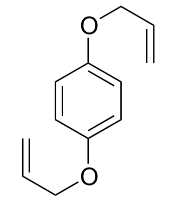

218

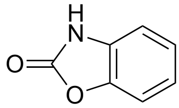

219

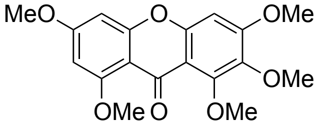

220

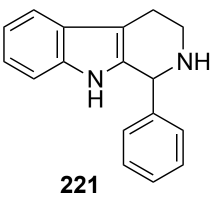

221

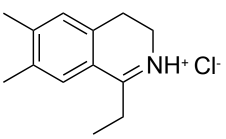

222

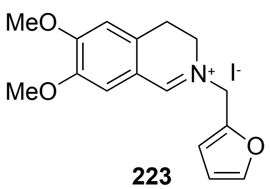

223

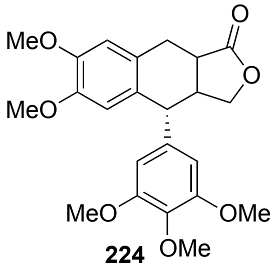

224

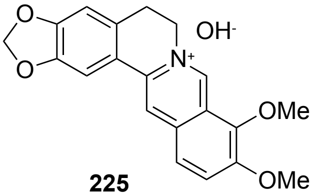

225

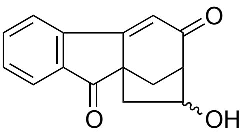

226

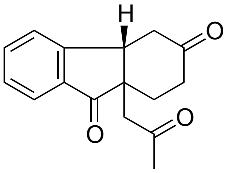

227

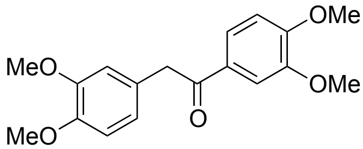

228

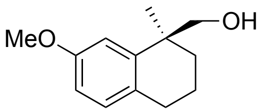

229

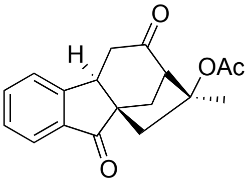

230

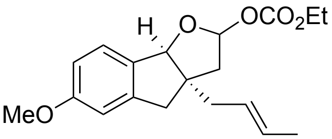

231

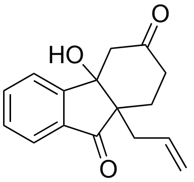

232

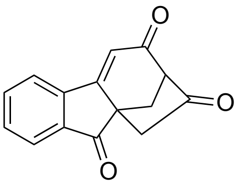

233

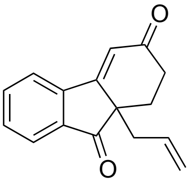

234

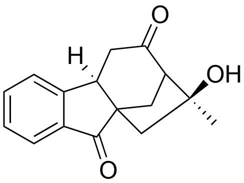

235

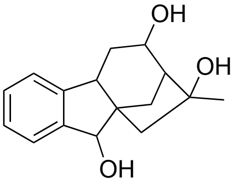

236

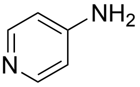

237

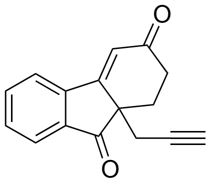

238

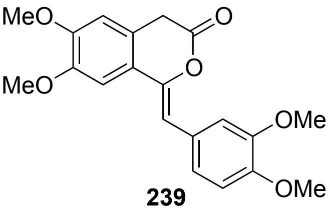

239

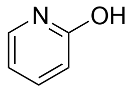

240

Supporting Figure 3-10 Structure of in-house library compound **217** - **240**

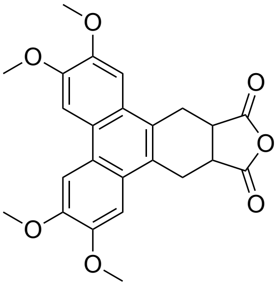

241

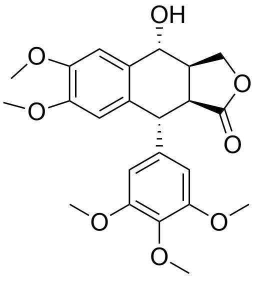

242

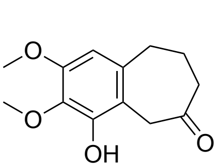

243

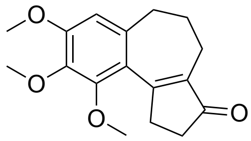

244

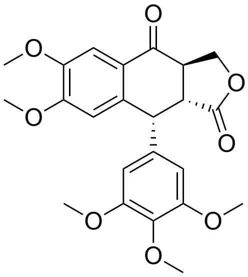

245

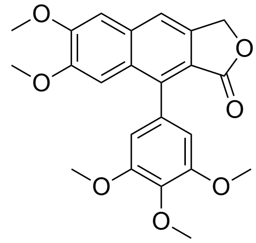

246

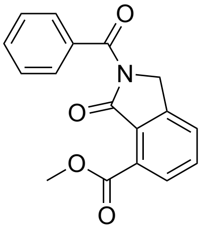

247

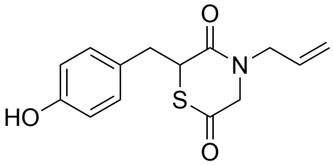

248

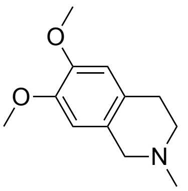

249

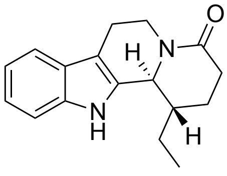

250

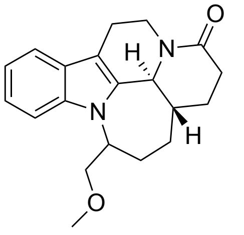

251

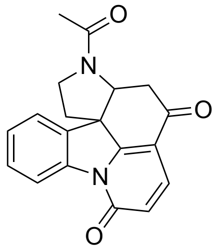

252

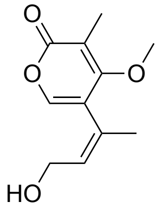

253

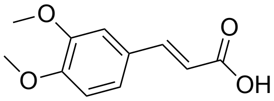

254

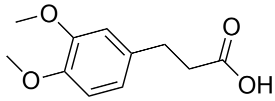

255

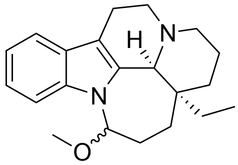

256

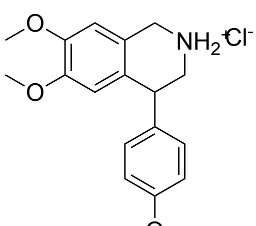

257

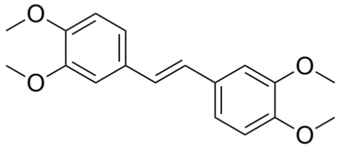

258

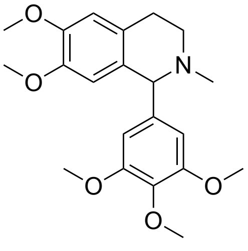

259

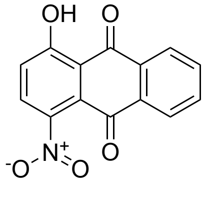

260

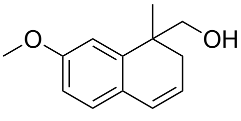

261

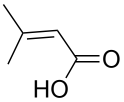

262

(blank)

263

(blank)

264

Supporting Figure 3-11 Structure of in-house library compound **241** - **264**

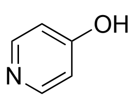
265

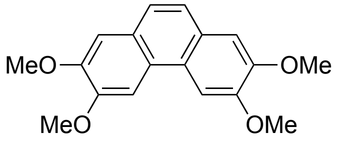
266

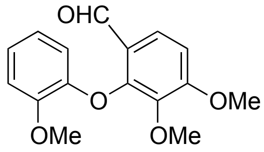
267

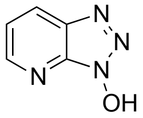
268

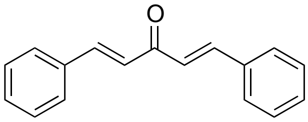
269

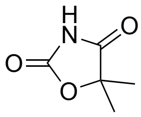
270

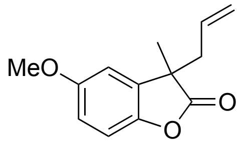
271

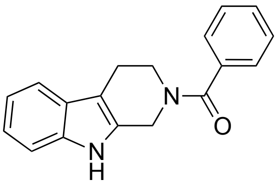
272

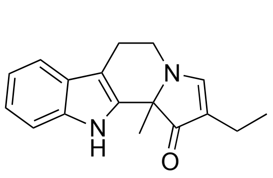
273

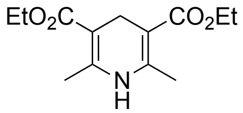
274

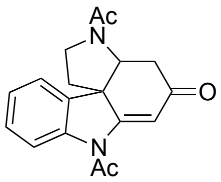
275

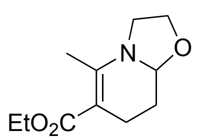
276

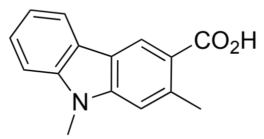
277

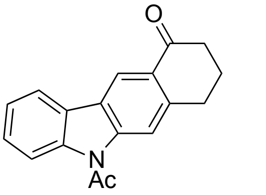
278

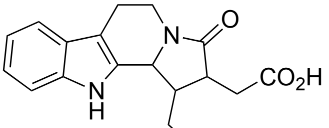
279

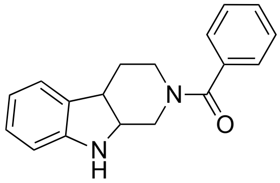
280

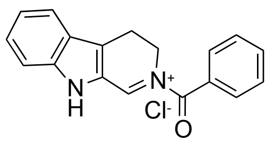
281

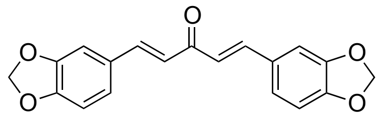
282

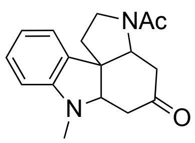
283

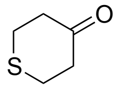
284

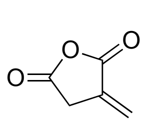
285

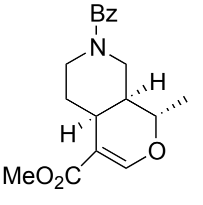
286

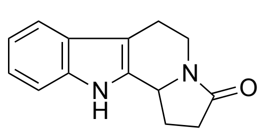
287

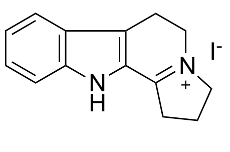
288

Supporting Figure 3-12 Structure of in-house library compound **265** - **288**

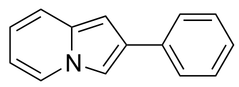

289

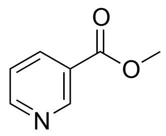

290

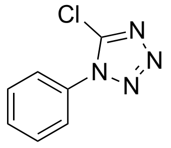

291

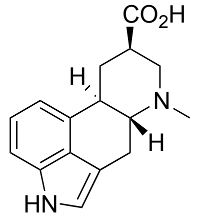

292

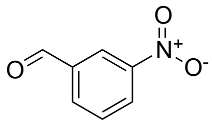

293

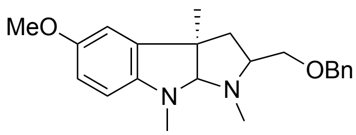

294

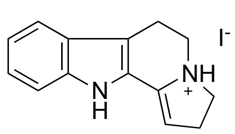

295

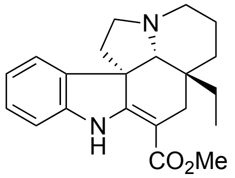

296

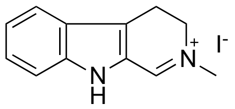

297

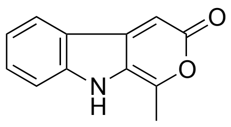

298

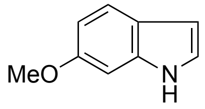

299

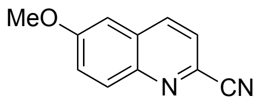

300

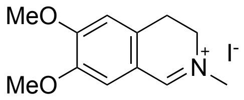

301

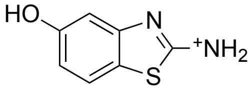

302

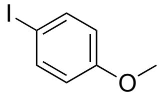

303

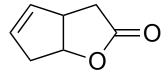

304

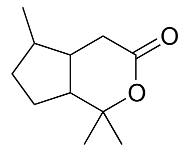

305

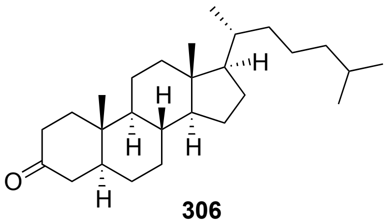

306

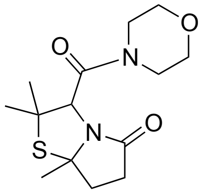

307

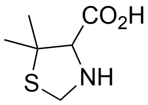

308

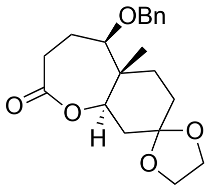

309

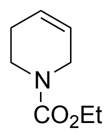

310

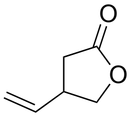

311

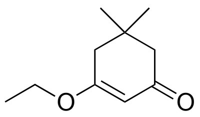

312

Supporting Figure 3-13 Structure of in-house library compound **289** - **312**

(blank)

313

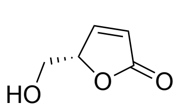

314

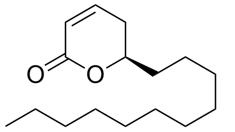

315

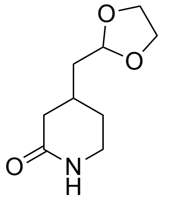

316

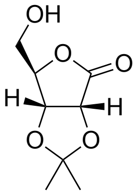

317

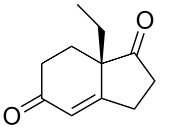

318

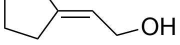

319

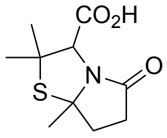

320

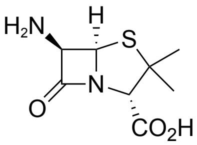

321

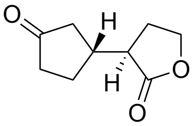

322

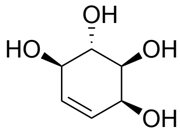

323

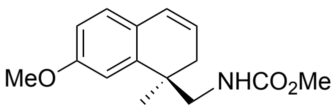

324

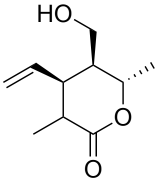

325

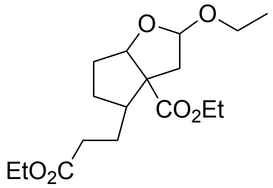

326

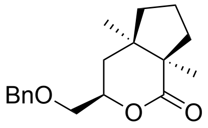

327

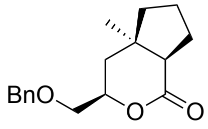

328

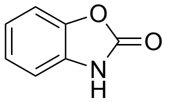

329

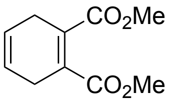

330

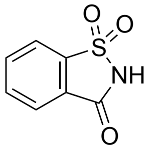

331

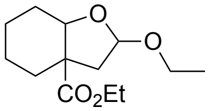

332

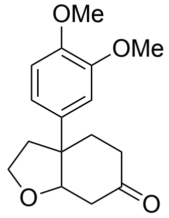

333

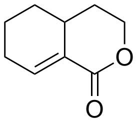

334

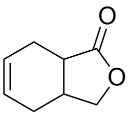

335

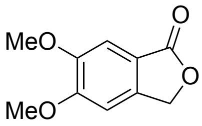

336

Supporting Figure 3-14 Structure of in-house library compound **313** - **336**

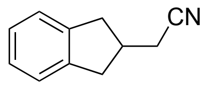

337

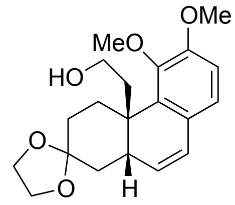

338

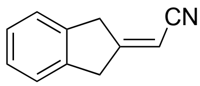

339

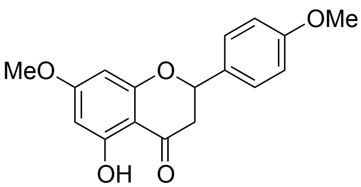

340

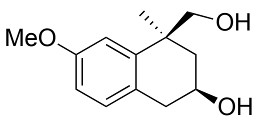

341

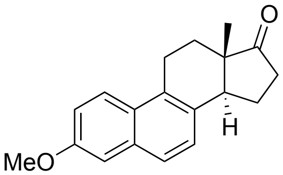

342

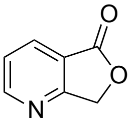

343

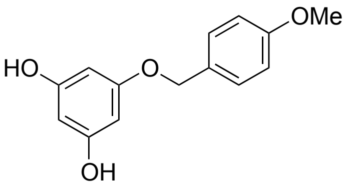

344

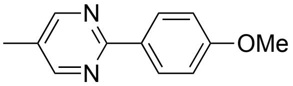

345

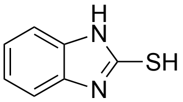

346

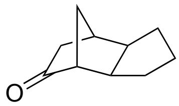

347

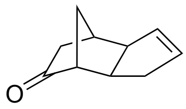

348

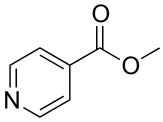

349

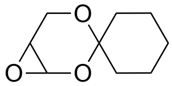

350

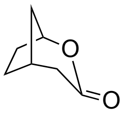

351

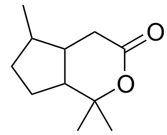

352

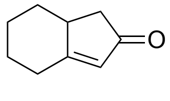

353

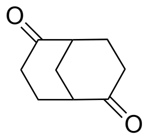

354

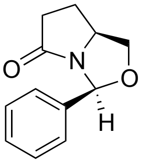

355

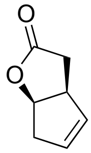

356

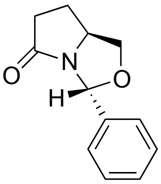

357

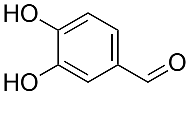

358

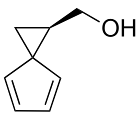

359

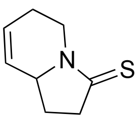

360

Supporting Figure 3-15 Structure of in-house library compound **337** - **360**

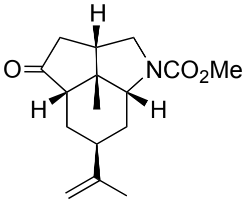

361

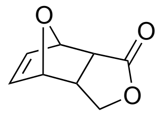

362

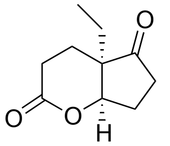

363

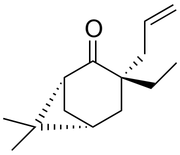

364

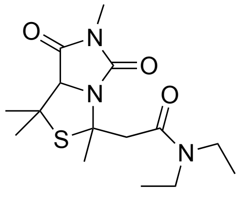

365

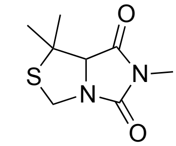

366

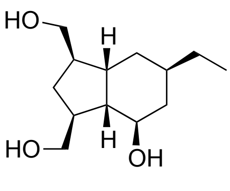

367

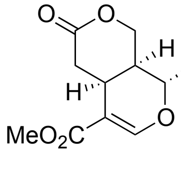

368

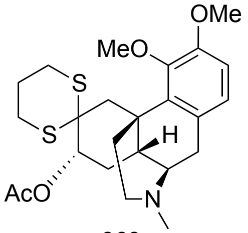

369

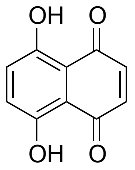

370

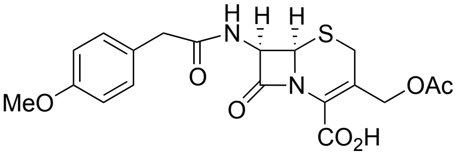

371

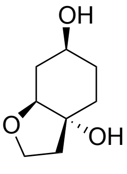

372

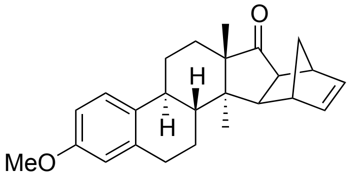

373

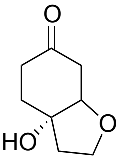

374

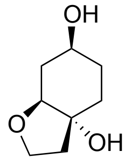

375

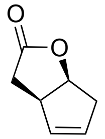

376

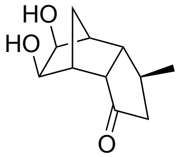

377

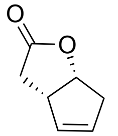

378

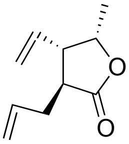

379

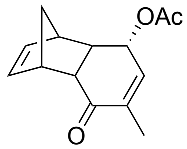

380

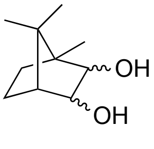

381

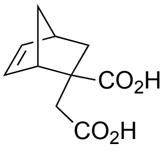

382

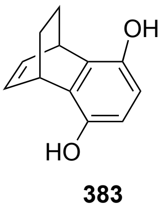

383

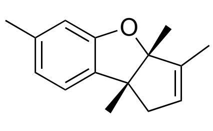

384

Supporting Figure 3-16 Structure of in-house library compound **361** - **384**

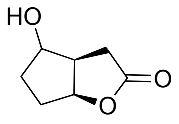

385

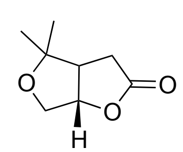

386

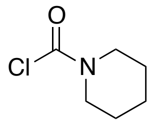

387

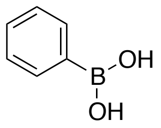

388

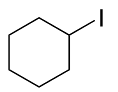

389

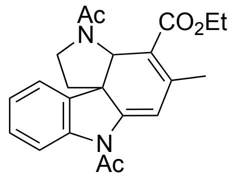

390

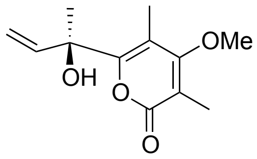

391

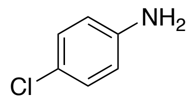

392

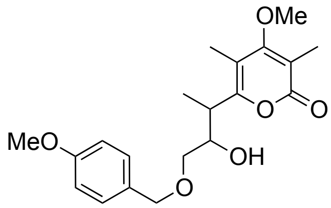

393

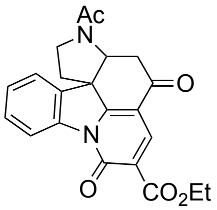

394

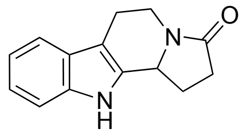

395

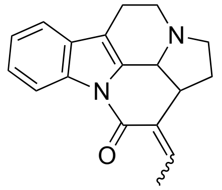

396

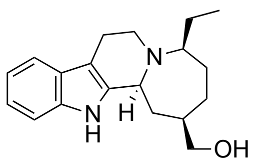

397

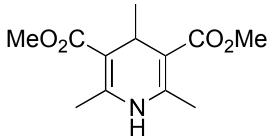

398

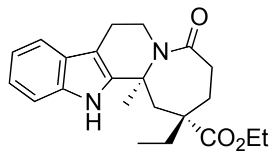

399

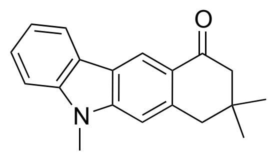

400

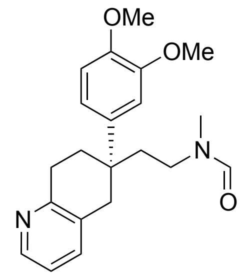

401

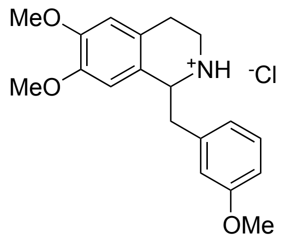

402

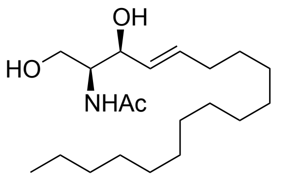

403

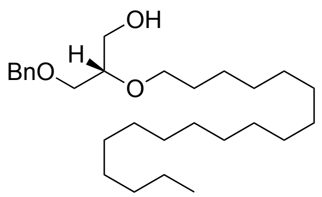

404

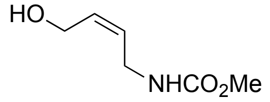

405

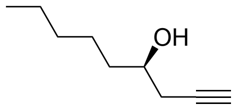

406

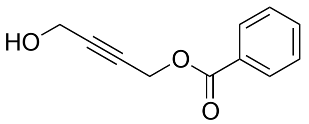

407

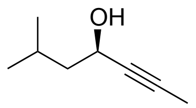

408

Supporting Figure 3-17 Structure of in-house library compound **385** - **408**

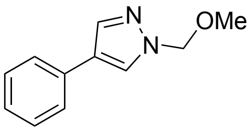

409

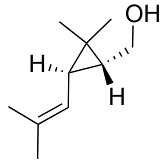

410

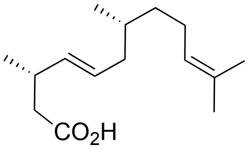

411

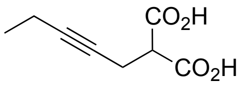

412

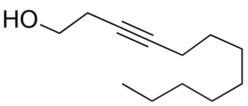

413

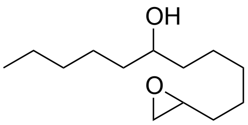

414

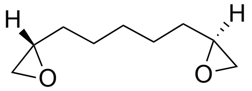

415

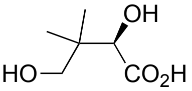

416

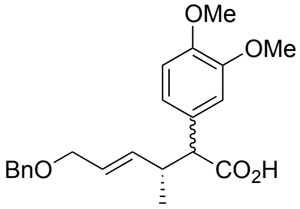

417

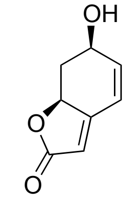

418

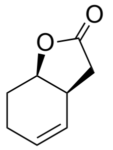

419

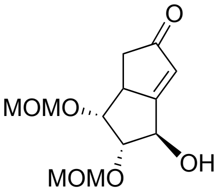

420

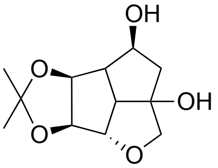

421

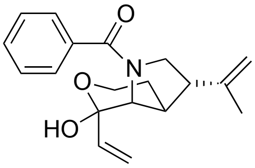

422

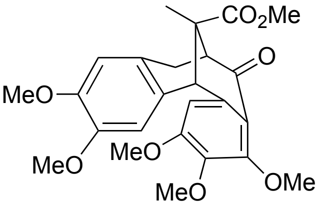

423

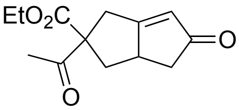

424

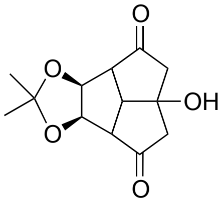

425

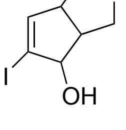

426

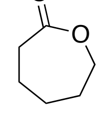

427

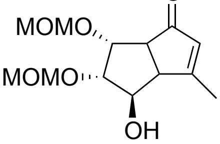

428

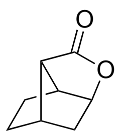

429

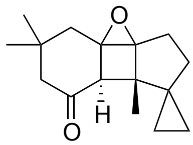

430

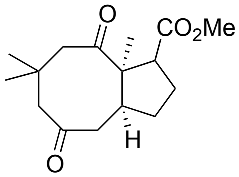

431

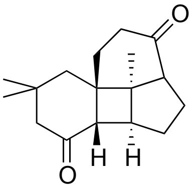

432

Supporting Figure 3-18 Structure of in-house library compound **409** - **432**

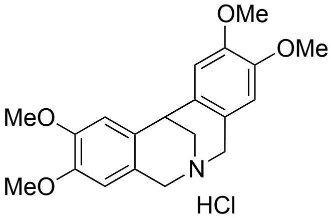

433

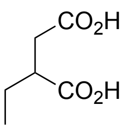

434

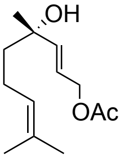

435

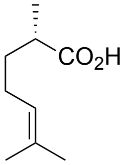

436

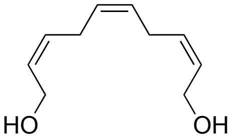

437

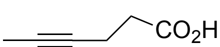

438

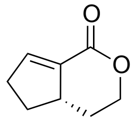

439

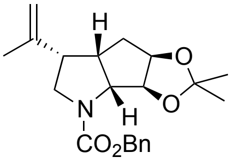

440

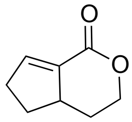

441

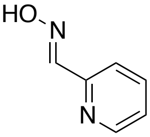

442

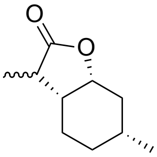

443

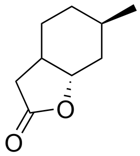

444

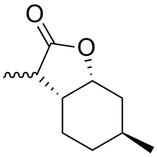

445

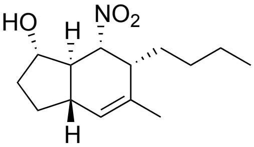

446

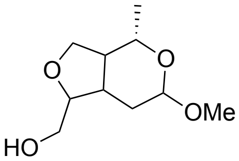

447

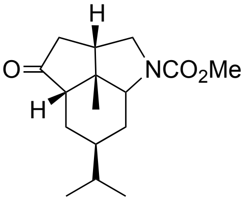

448

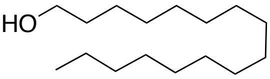

449

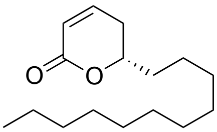

450

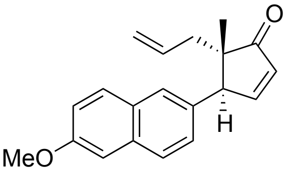

451

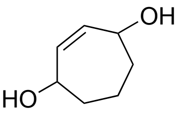

452

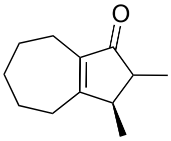

453

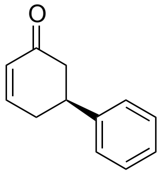

454

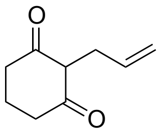

455

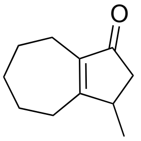

456

Supporting Figure 3-19 Structure of in-house library compound **433** - **456**

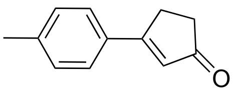

457

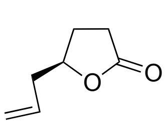

458

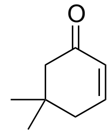

459

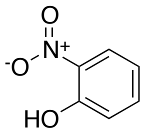

460

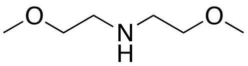

461

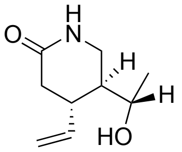

462

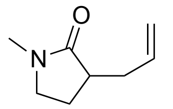

463

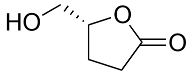

464

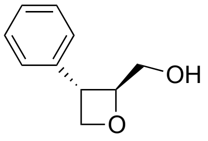

465

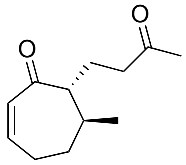

466

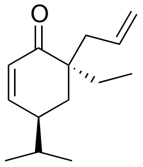

467

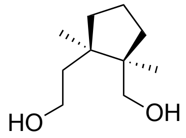

468

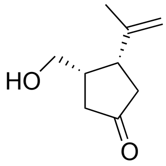

469

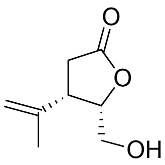

470

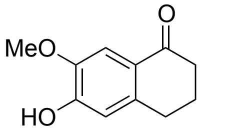

471

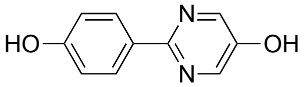

472

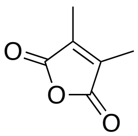

473

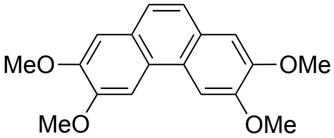

474

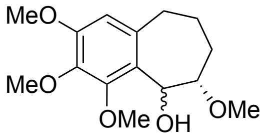

475

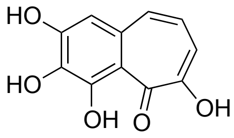

476

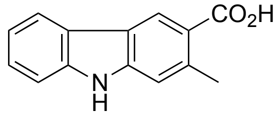

477

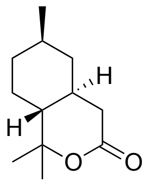

478

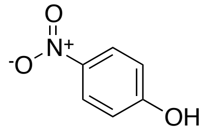

479

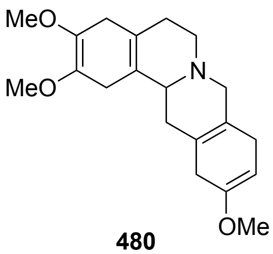

480

Supporting Figure 3-20 Structure of in-house library compound **457** - **480**

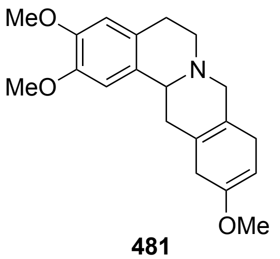
481
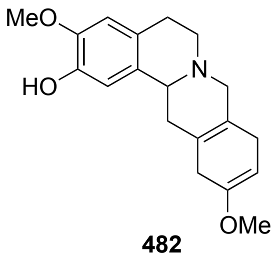
482
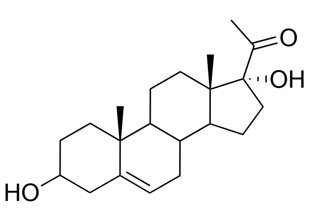
483
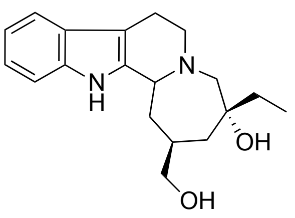
484

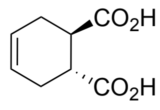
485
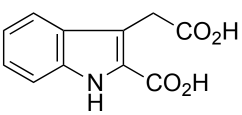
486
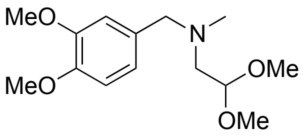
487
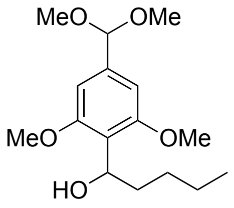
488

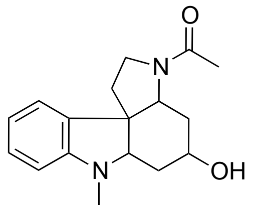
489
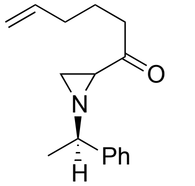
490
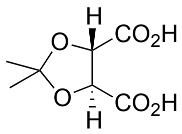
491
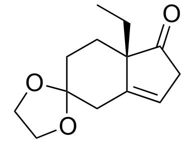
492

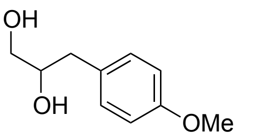
493
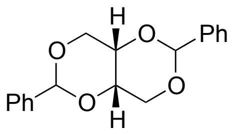
494
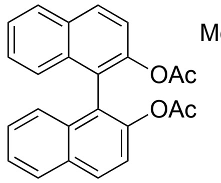
495
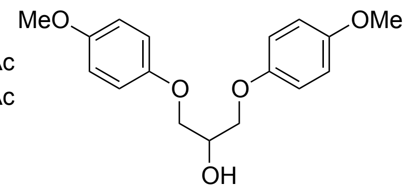
496

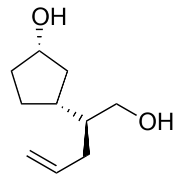
497
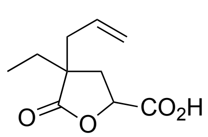
498
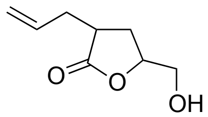
499
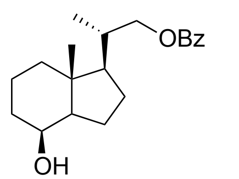
500

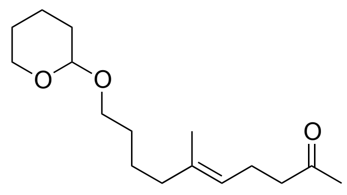
501
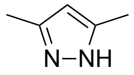
502
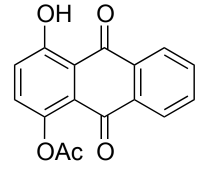
503
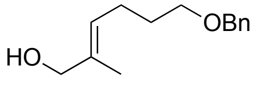
504

Supporting Figure 3-21 Structure of in-house library compound **481** - **504**

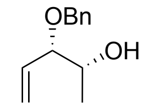

505

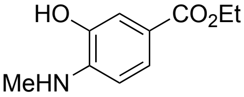

506

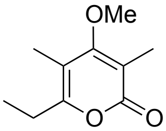

507

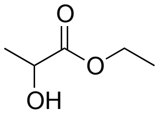

508

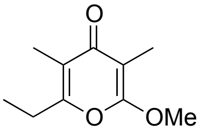

509

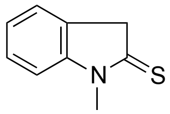

510

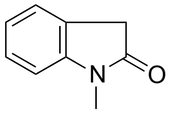

511

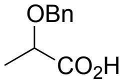

512

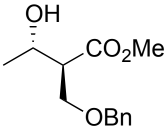

513

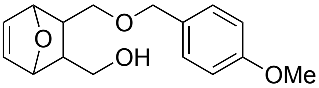

514

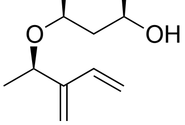

515

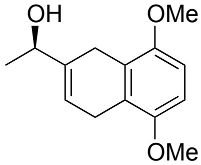

516

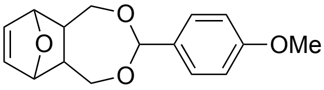

517

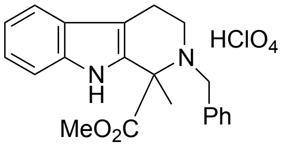

518

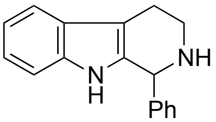

519

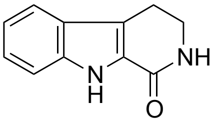

520

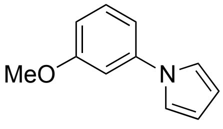

521

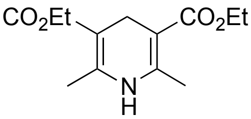

522

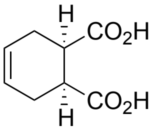

523

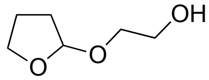

524

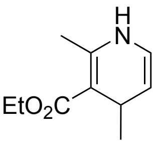

525

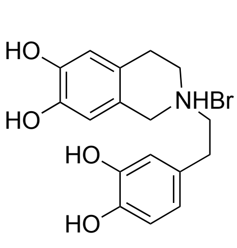

526

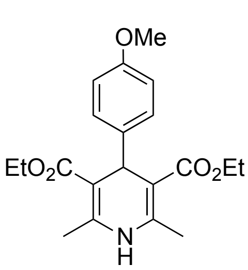

527

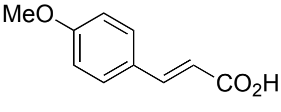

528

Supporting Figure 3-22 Structure of in-house library compound **505** - **528**

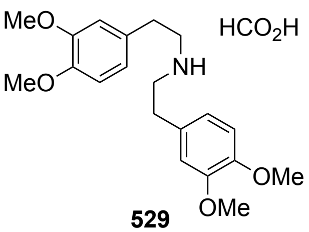

529

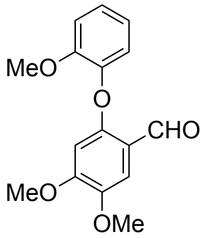

530

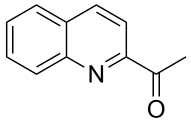

531

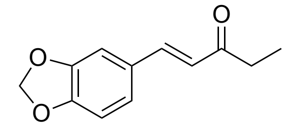

532

(blank)

533

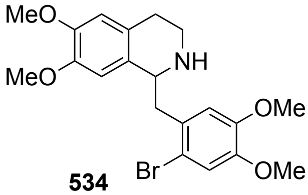

534

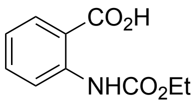

535

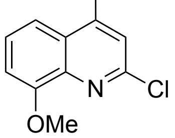

536

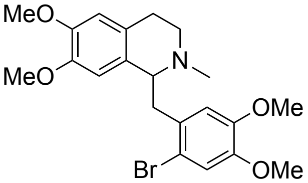

537

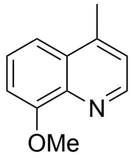

538

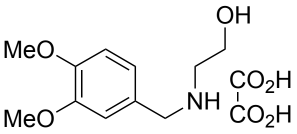

539

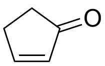

540

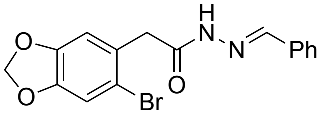

541

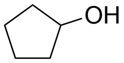

542

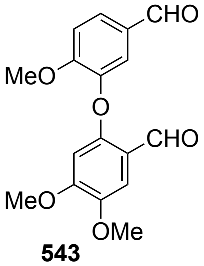

543

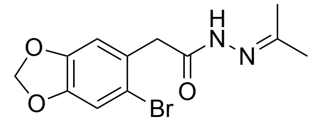

544

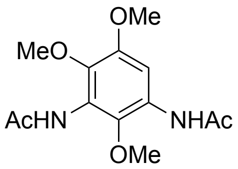

545

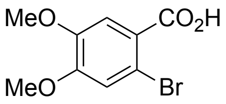

546

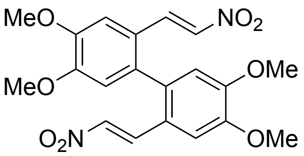

547

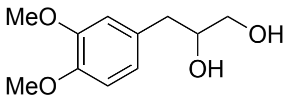

548

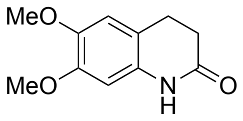

549

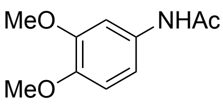

550

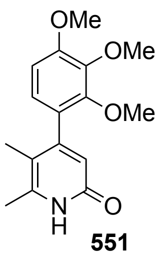

551

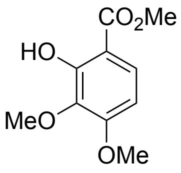

552

Supporting Figure 3-23 Structure of in-house library compound **529** - **551**

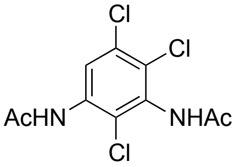

553

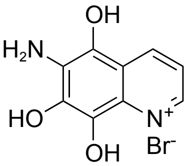

554

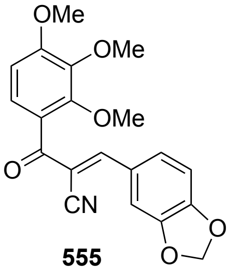

555

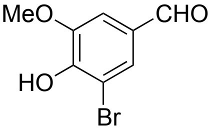

556

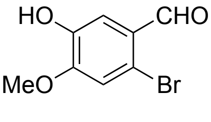

557

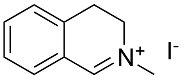

558

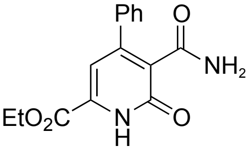

559

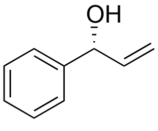

560

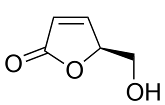

561

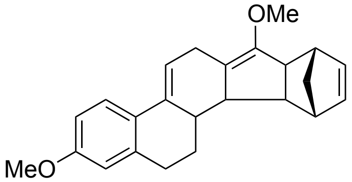

562

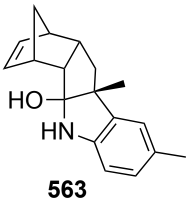

563

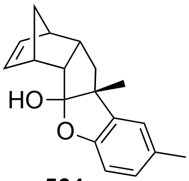

564

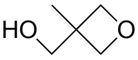

565

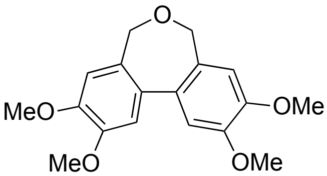

566

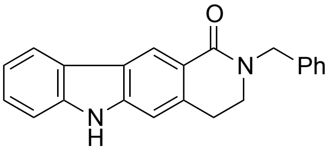

567

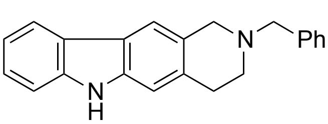

568

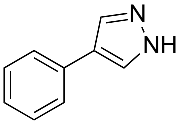

569

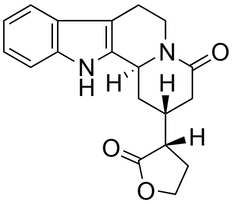

570

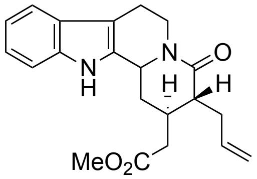

571

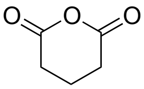

572

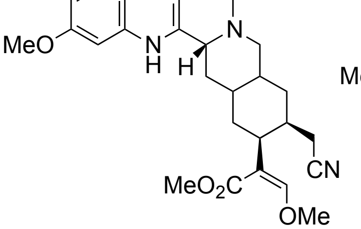

573

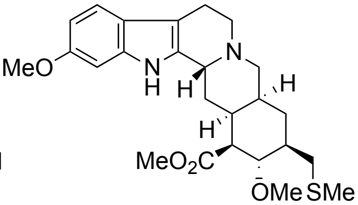

574

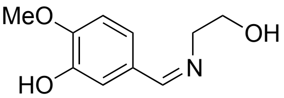

575

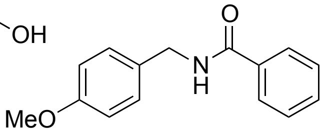

576

Supporting Figure 3-24 Structure of in-house library compound **553** - **576**

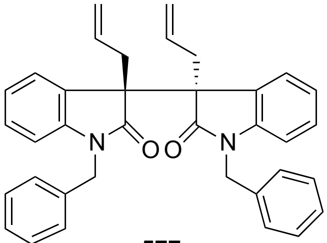

577

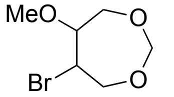

578

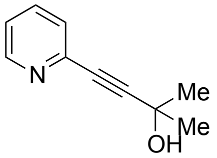

579

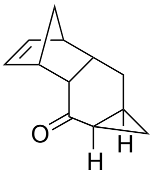

580

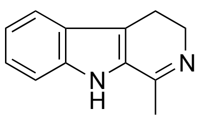

581

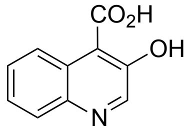

582

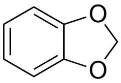

583

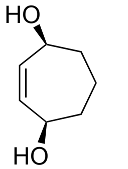

584

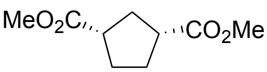

585

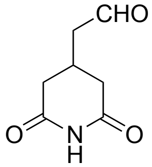

586

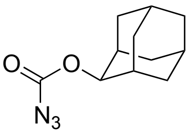

587

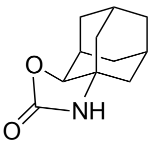

588

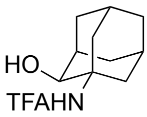

589

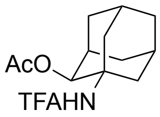

590

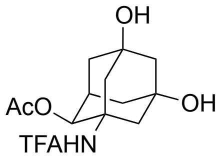

591

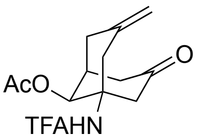

592

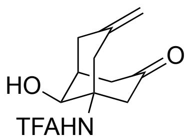

593

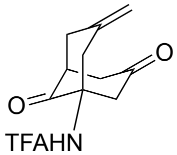

594

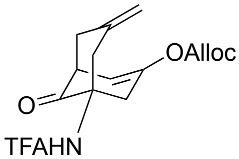

595

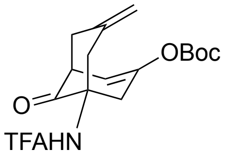

596

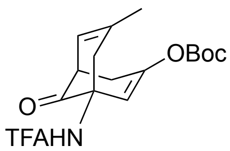

597

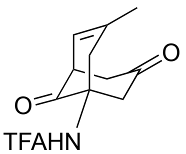

598

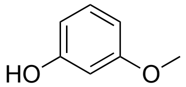

599

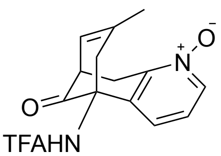

600

Supporting Figure 3-25 Structure of in-house library compound **577** - **600**

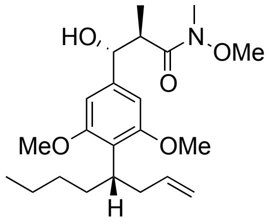

601

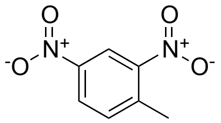

602

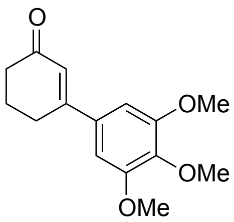

603

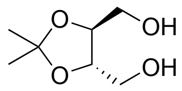

604

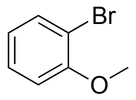

605

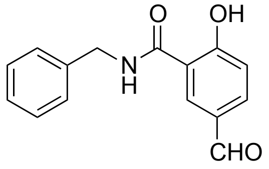

606

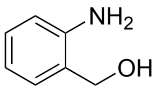

607

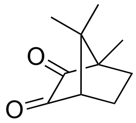

608

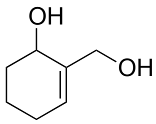

609

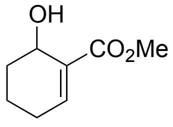

610

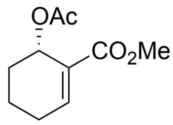

611

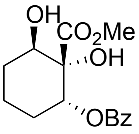

612

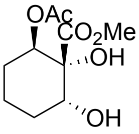

613

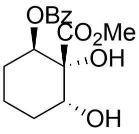

614

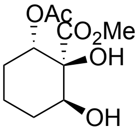

615

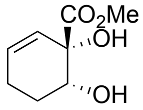

616

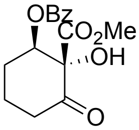

617

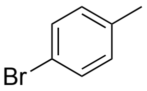

618

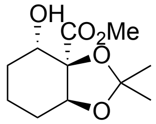

619

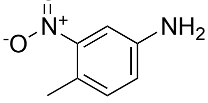

620

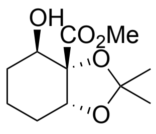

621

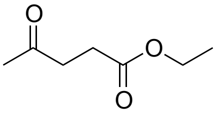

622

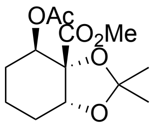

623

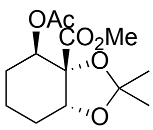

624

Supporting Figure 3-26 Structure of in-house library compound **601** - **624**

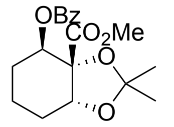

625

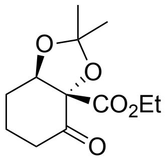

626

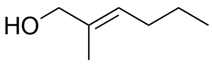

627

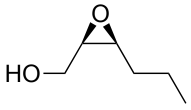

628

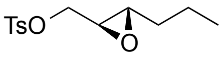

629

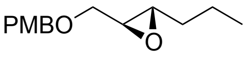

630

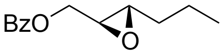

631

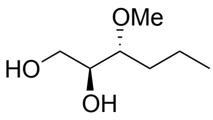

632

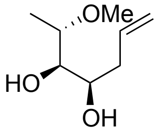

633

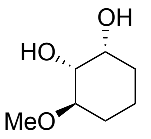

634

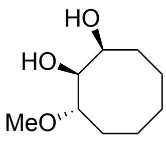

635

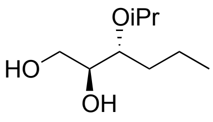

636

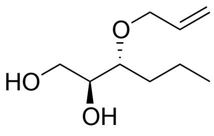

637

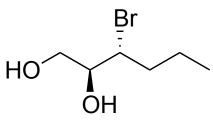

638

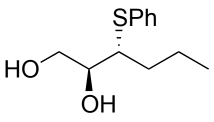

639

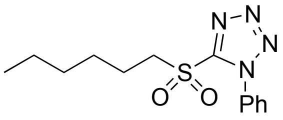

640

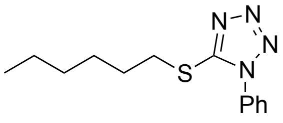

641

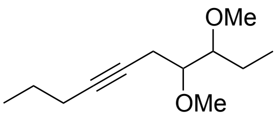

642

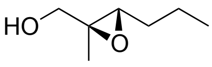

643

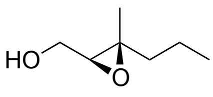

644

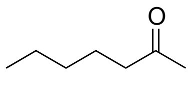

645

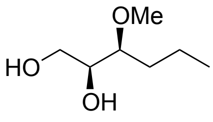

646

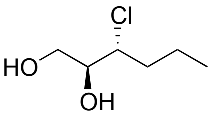

647

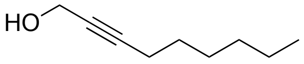

648

Supporting Figure 3-27 Structure of in-house library compound **625** - **648**

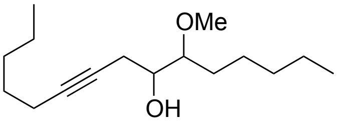
649

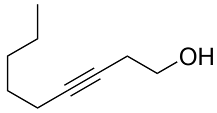
650

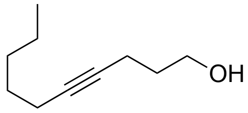
651

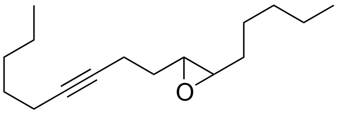
652

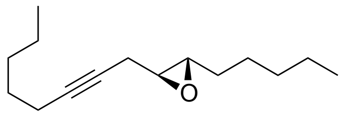
653

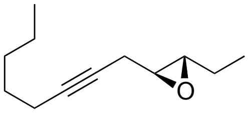
654

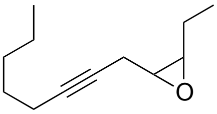
655

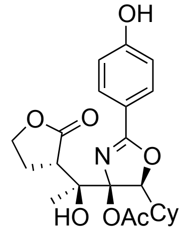
656

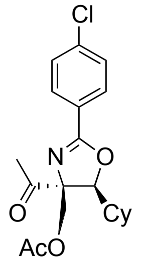
657

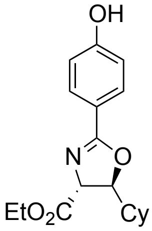
658

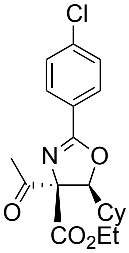
659

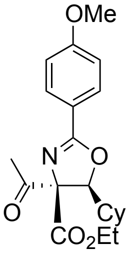
660

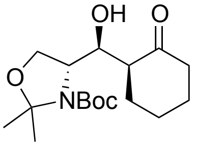
661

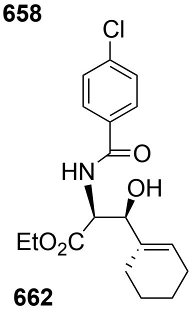
662

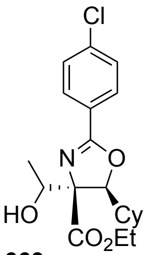
663

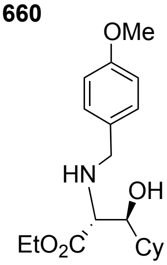
664

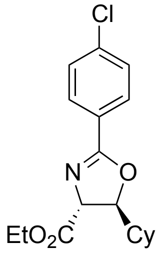
665

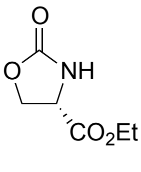
666

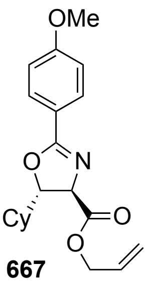
667

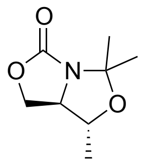
668

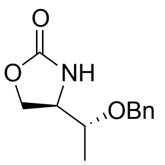
669

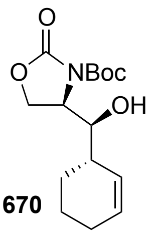
670

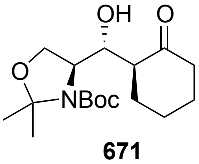
671

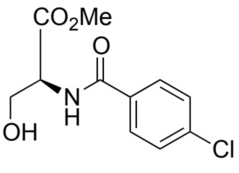
672

Supporting Figure 3-28 Structure of in-house library compound **649** - **672**

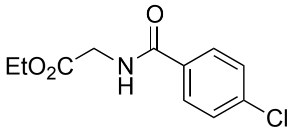

673

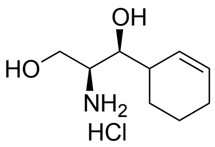

674

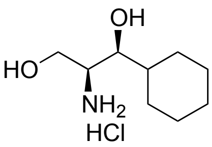

675

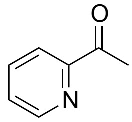

676

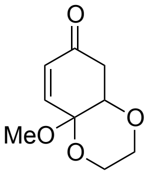

677

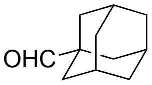

678

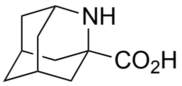

679

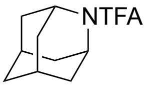

680

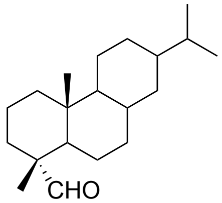

681

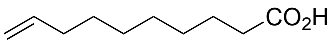

682

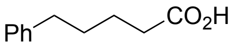

683

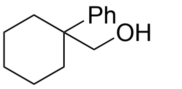

684

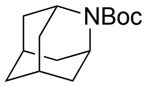

685

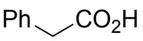

686

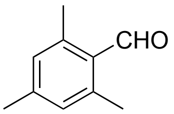

687

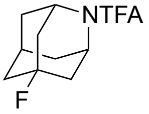

688

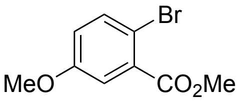

689

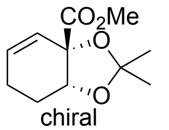

690

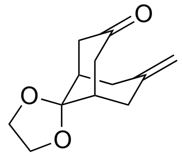

691

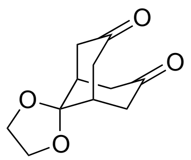

692

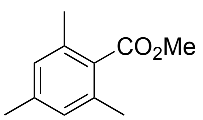

693

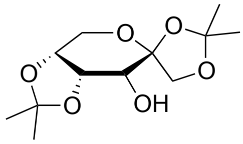

694

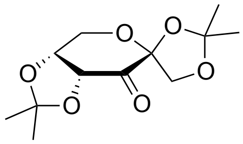

695

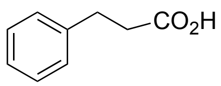

696

Supporting Figure 3-29 Structure of in-house library compound **673** - **696**

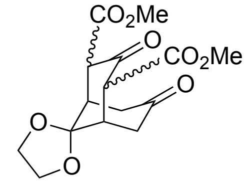

697

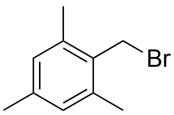

698

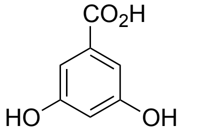

699

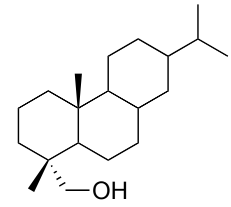

700

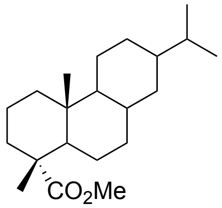

701

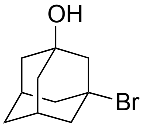

702

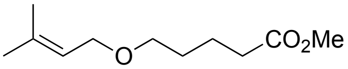

703

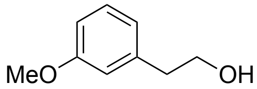

704

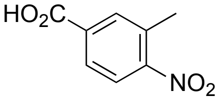

705

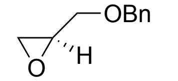

706

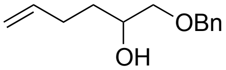

707

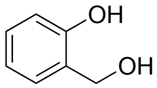

708

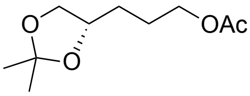

709

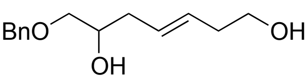

710

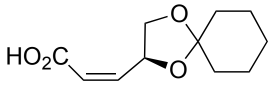

711

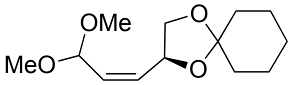

712

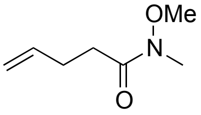

713

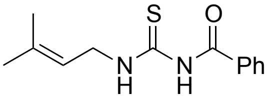

714

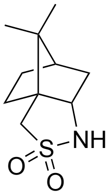

715

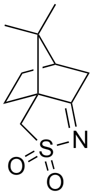

716

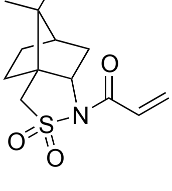

717

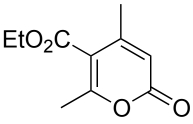

718

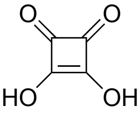

719

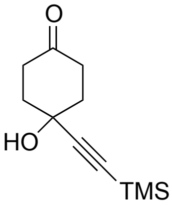

720

Supporting Figure 3-30 Structure of in-house library compound **697** - **720**

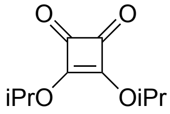

721

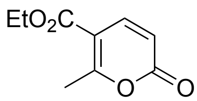

722

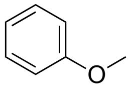

723

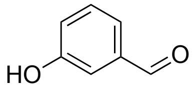

724

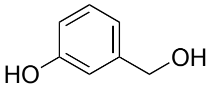

725

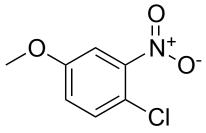

726

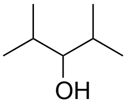

727

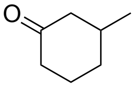

728

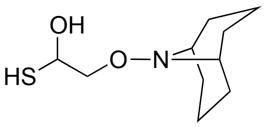

729

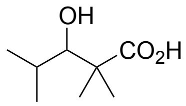

730

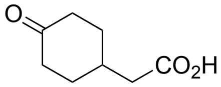

731

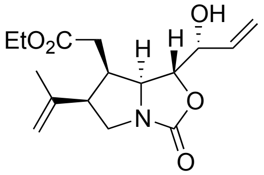

732

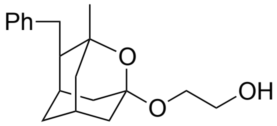

733

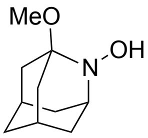

734

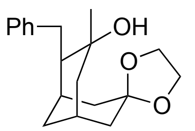

735

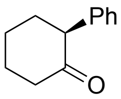

736

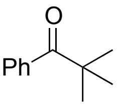

737

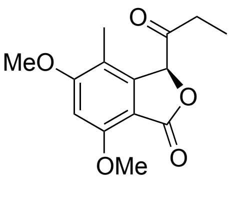

738

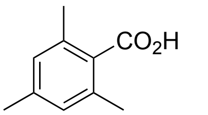

739

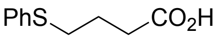

740

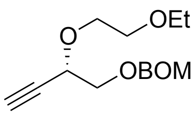

741

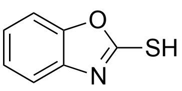

742

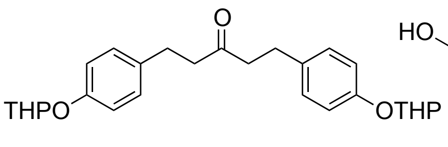

743

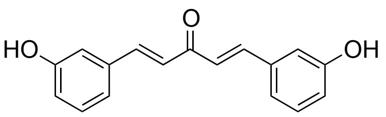

744

Supporting Figure 3-31 Structure of in-house library compound **721** - **744**

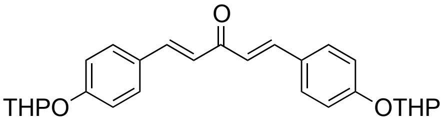

745

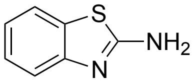

746

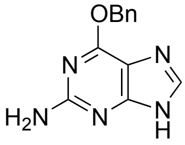

747

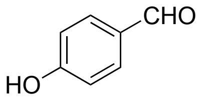

748

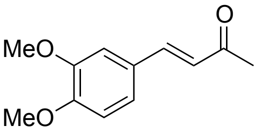

749

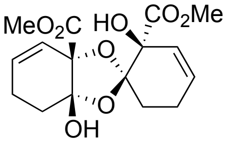

750

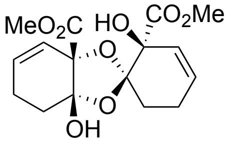

751

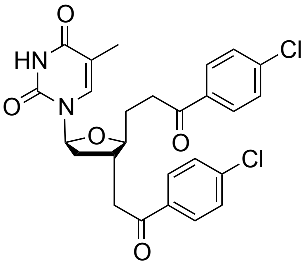

752

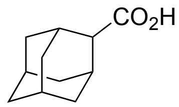

753

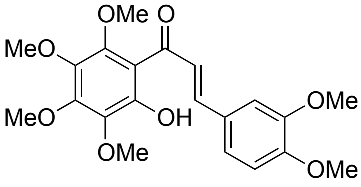

754

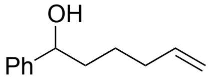

755

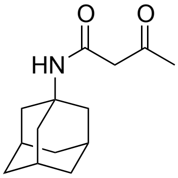

756

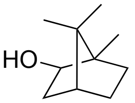

757

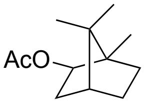

758

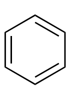

759

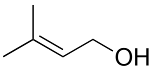

760

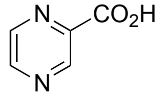

761

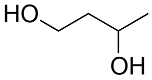

762

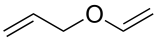

763

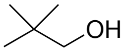

764

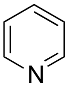

765

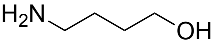

766

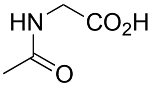

767

(blank)

768

Supporting Figure 3-32 Structure of in-house library compound **745** - **768**

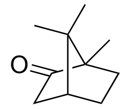

769

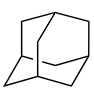

770

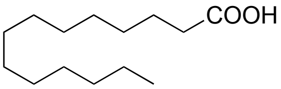

771

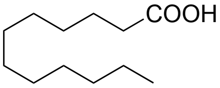

772

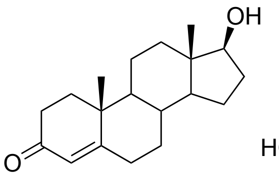

773

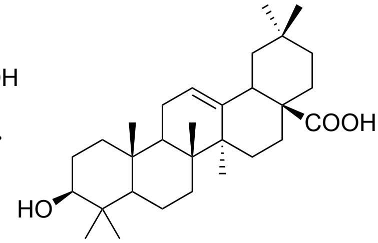

774

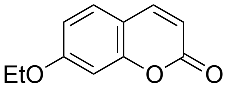

775

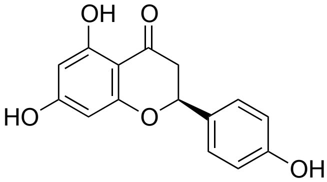

776

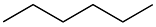

777

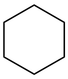

778

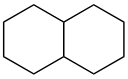

779

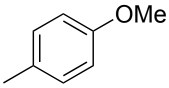

780

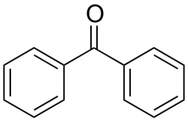

781

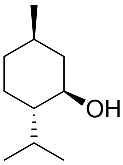

782

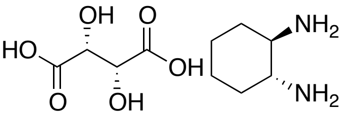

783

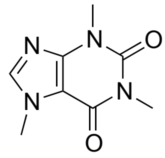

784

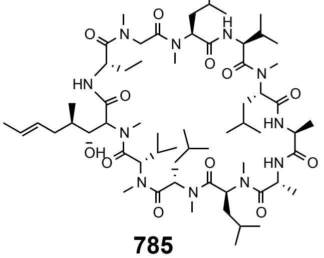

785

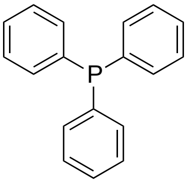

786

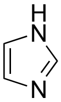

787

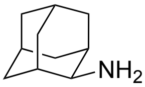

788

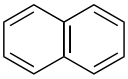

789

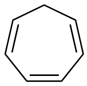

790

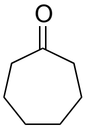

791

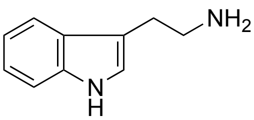

792

Supporting Figure 3-33 Structure of in-house library compound **769** - **792**

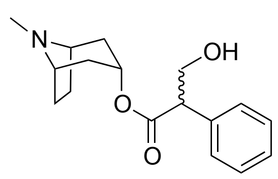

793

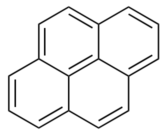

794

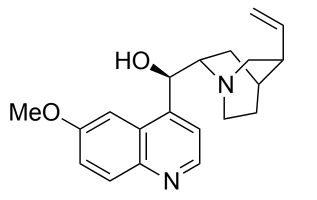

795

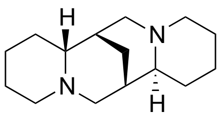

796

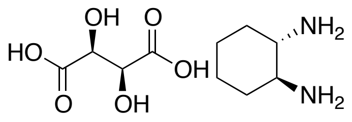

797

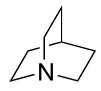

798

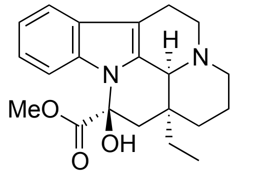

799

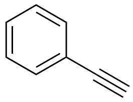

800

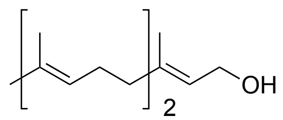

801

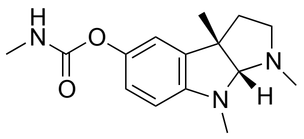

802

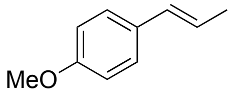

803

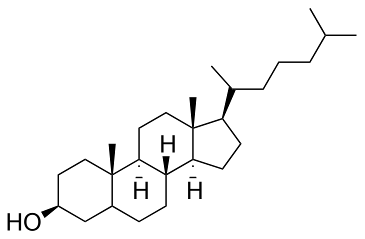

804

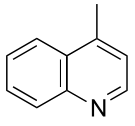

805

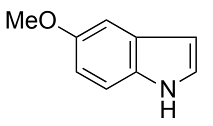

806

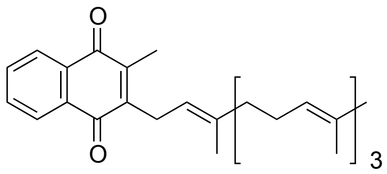

807

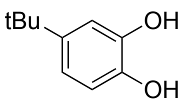

808

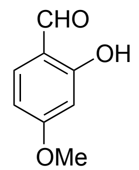

809

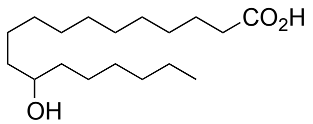

810

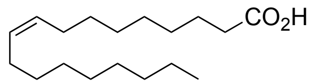

811

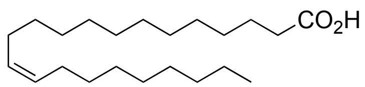

812

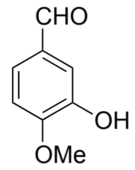

813

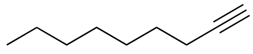

814

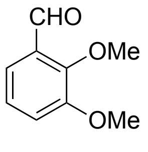

815

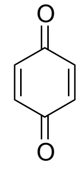

816

Supporting Figure 3-34 Structure of in-house library compound **793** - **816**

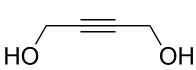

817

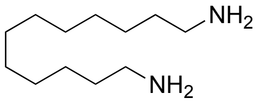

818

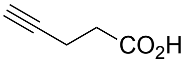

819

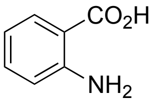

820

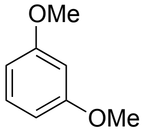

821

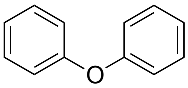

822

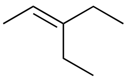

823

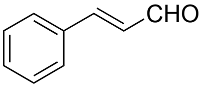

824

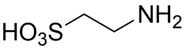

825

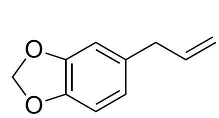

826

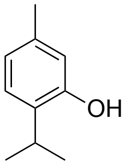

827

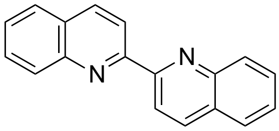

828

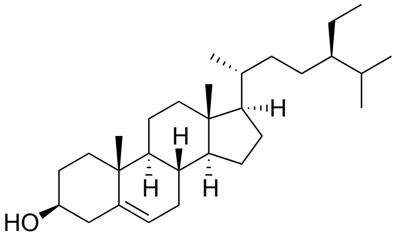

829

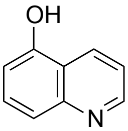

830

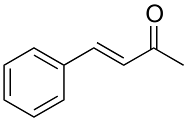

831

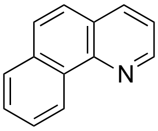

832

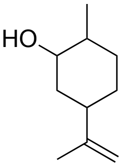

833

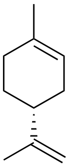

834

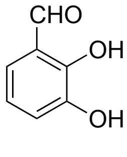

835

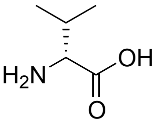

836

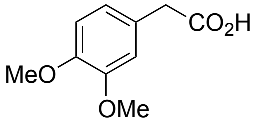

837

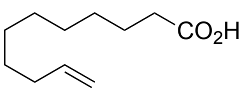

838

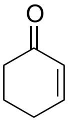

839

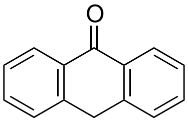

840

Supporting Figure 3-35 Structure of in-house library compound **817** - **840**

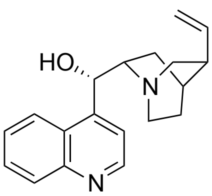

841

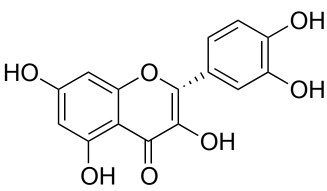

842

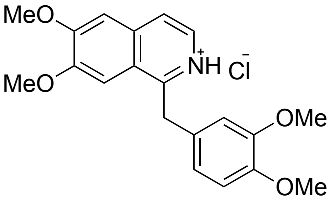

843

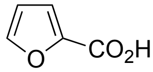

844

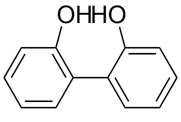

845

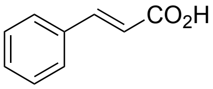

846

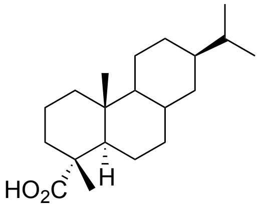

847

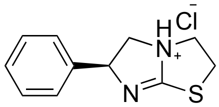

848

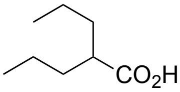

849

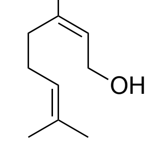

850

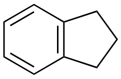

851

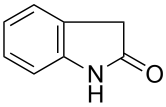

852

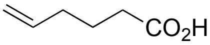

853

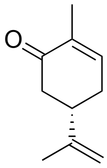

854

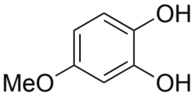

855

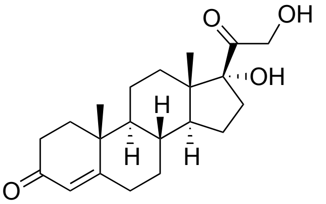

856

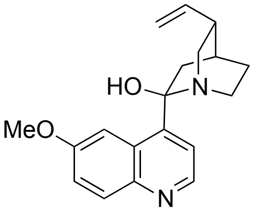

857

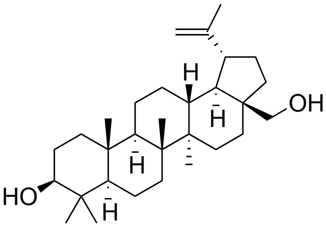

858

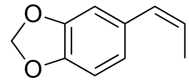

859

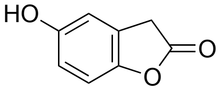

860

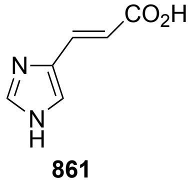

861

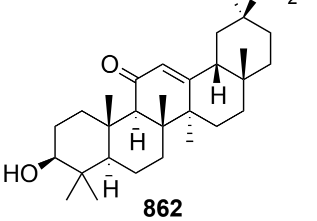

862

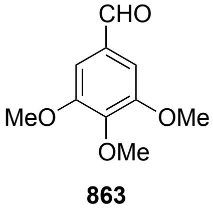

863

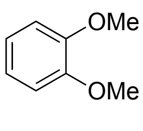

864

Supporting Figure 3-36 Structure of in-house library compound **841** - **867**

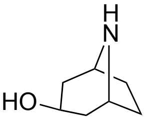

865

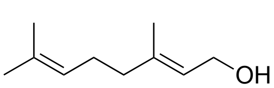

866

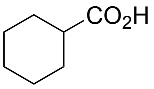

867

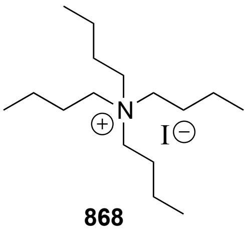

868

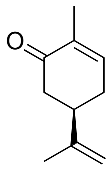

869

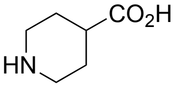

870

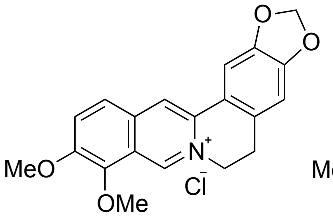

871

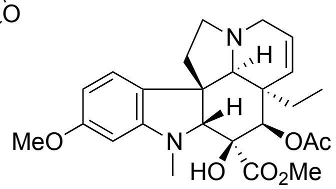

872

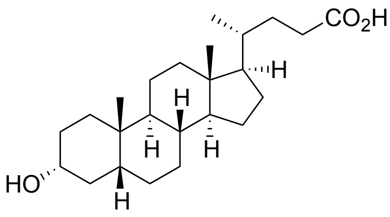

873

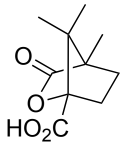

874

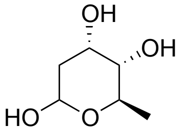

875

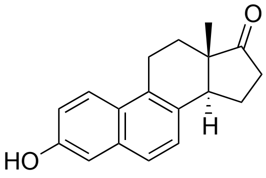

876

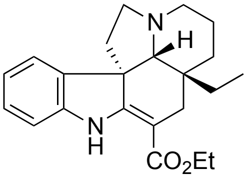

877

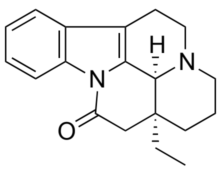

878

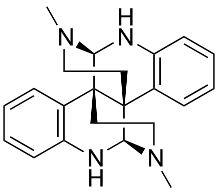

879

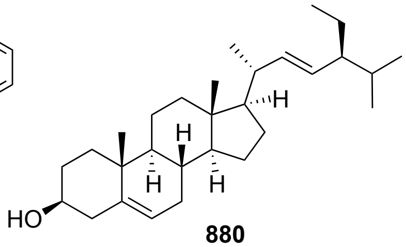

880

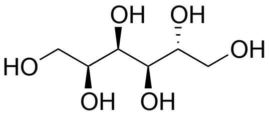

881

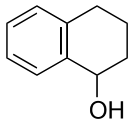

882

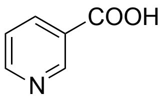

883

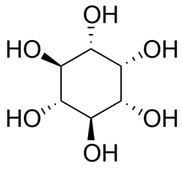

884

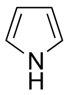

885

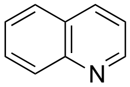

886

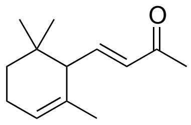

887

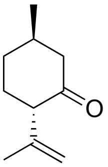

888

Supporting Figure 3-37 Structure of in-house library compound **865** - **888**

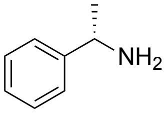

889

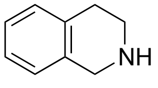

890

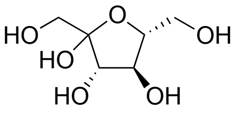

891

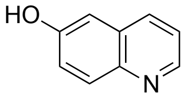

892

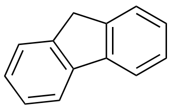

893

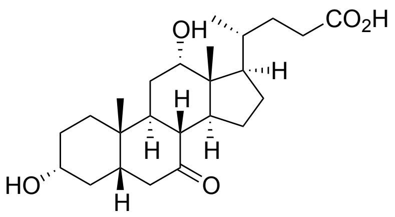

894

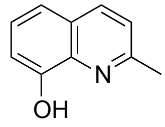

895

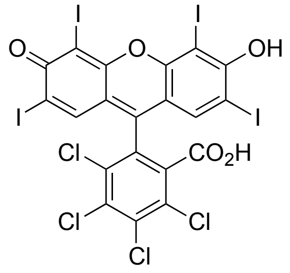

896

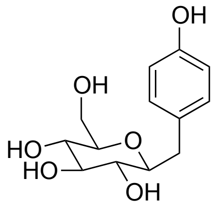

897

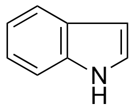

898

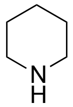

899

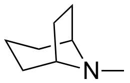

900

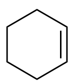

901

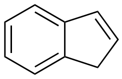

902

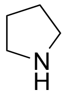

903

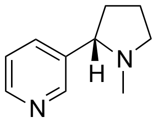

904

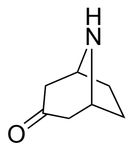

905

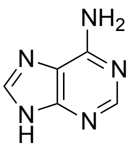

906

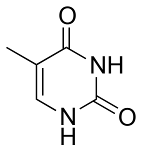

907

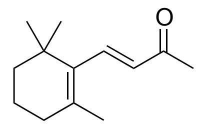

908

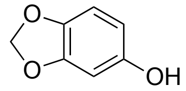

909

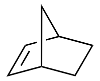

910

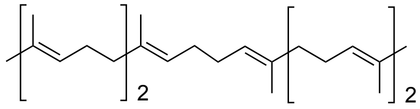

911

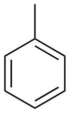

912

Supporting Figure 3-38 Structure of in-house library compound **889** - **912**

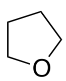

913

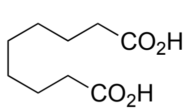

914

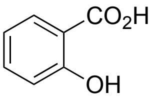

915

Chlorophyll

916

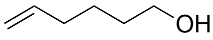

917

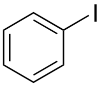

918

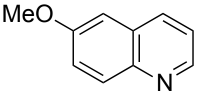

919

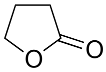

920

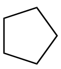

921

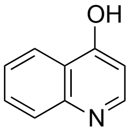

922

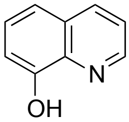

923

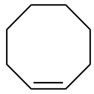

924

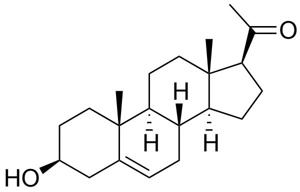

925

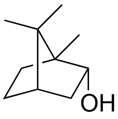

926

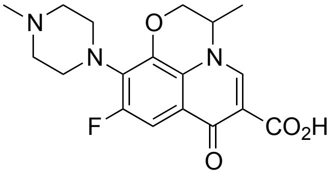

927

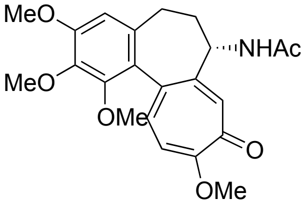

928

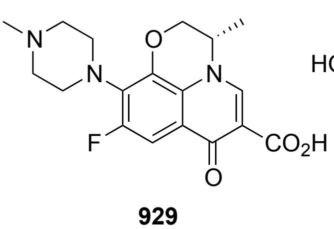

929

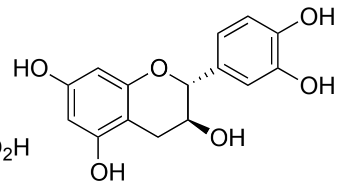

930

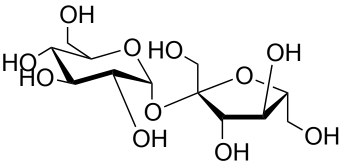

931

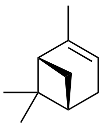

932

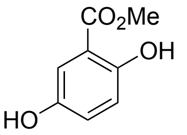

933

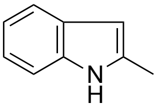

934

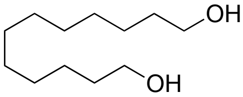

935

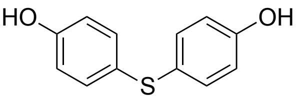

936

Supporting Figure 3-39 Structure of in-house library compound **913** - **936**

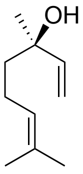

937

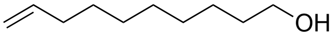

938

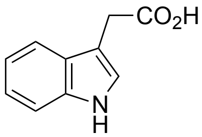

939

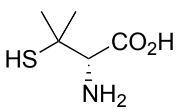

940

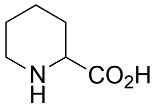

941

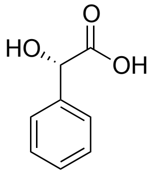

942

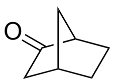

943

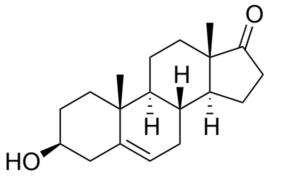

944

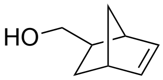

945

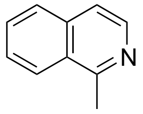

946

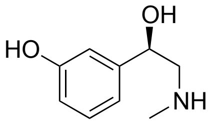

947

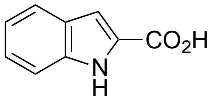

948

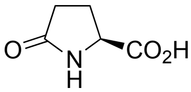

949

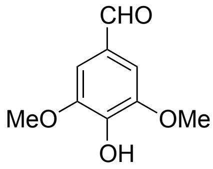

950

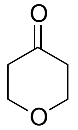

951

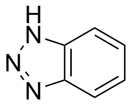

952

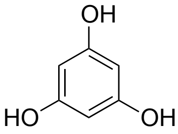

953

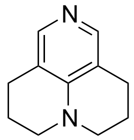

954

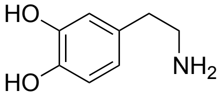

955

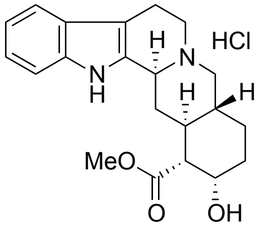

956

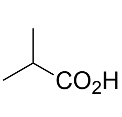

957

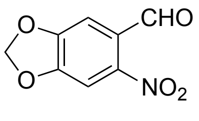

958

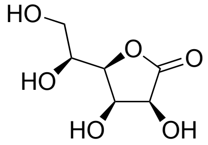

959

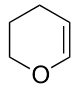

960

Supporting Figure 3-40 Structure of in-house library compound **937** - **960**

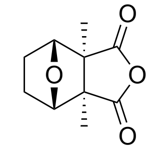

961

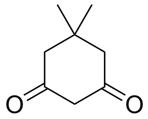

962

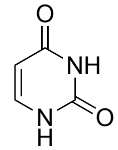

963

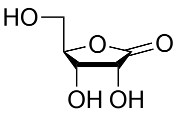

964

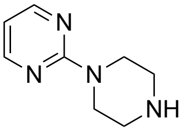

965

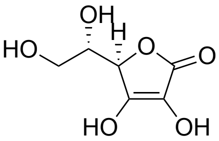

966

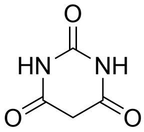

967

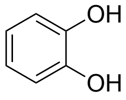

968

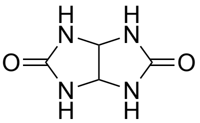

969

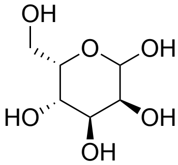

970

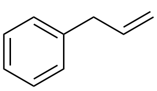

971

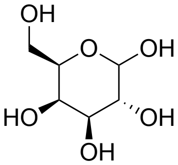

972

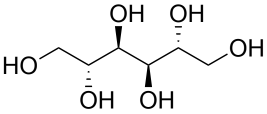

973

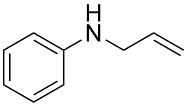

974

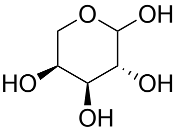

975

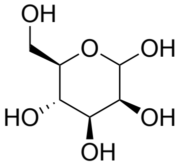

976

(blank)

977

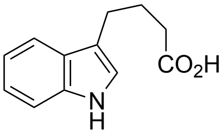

978

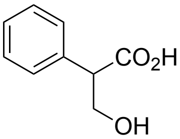

979

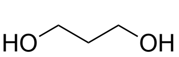

980

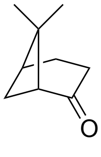

981

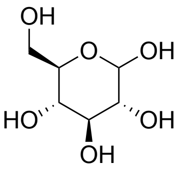

982

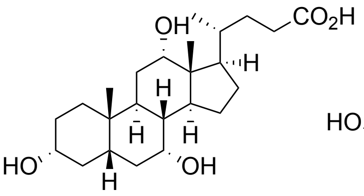

983

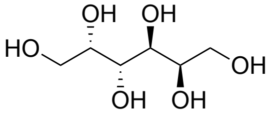

984

Supporting Figure 3-41 Structure of in-house library compound **961** - **984**

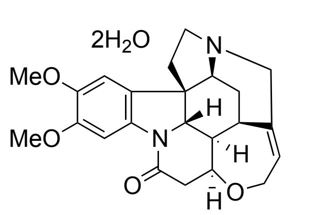
985

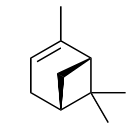
986

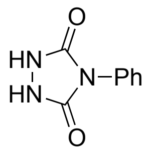
987

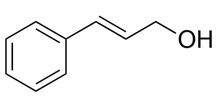
988

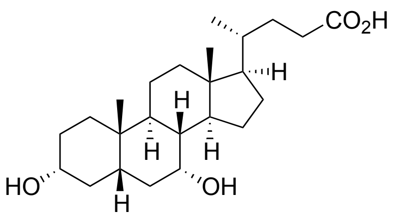
989

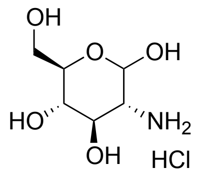
990

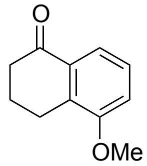
991

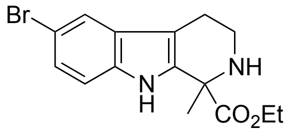
992

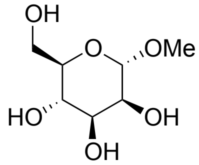
993

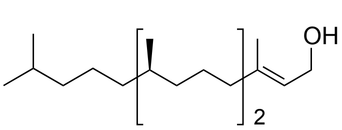
994

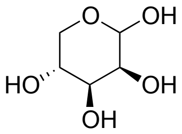
995

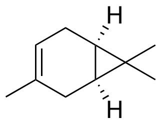
996

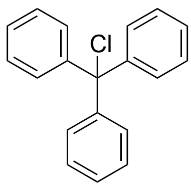
997

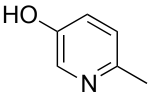
998

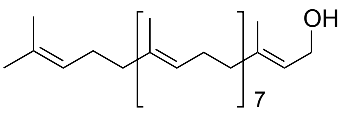
999

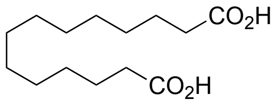
1000

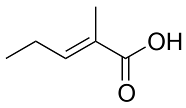
1001

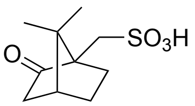
1002

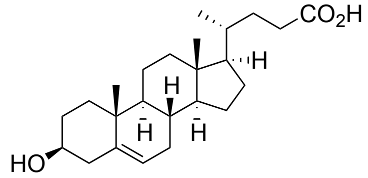
1003

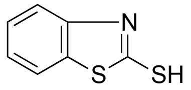
1004

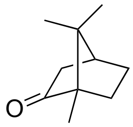
1005

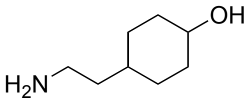
1006

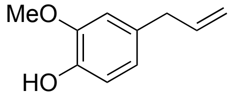
1007

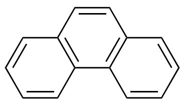
1008

Supporting Figure 3-42 Structure of in-house library compound **985** - **1008**

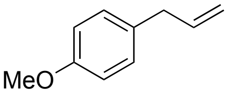

1009

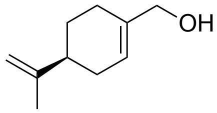

1010

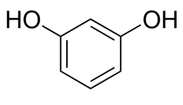

1011

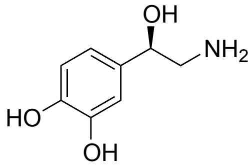

1012

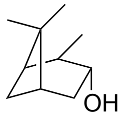

1013

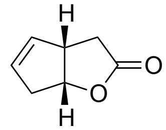

1014

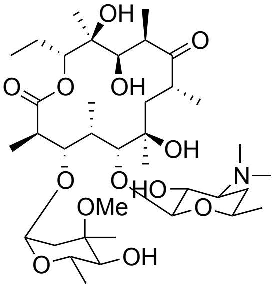

1015

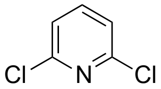

1016

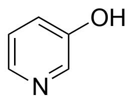

1017

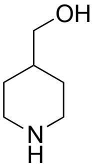

1018

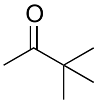

1019

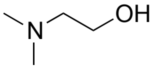

1020

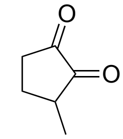

1021

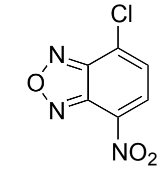

1022

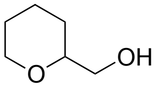

1023

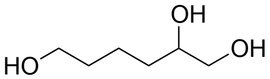

1024

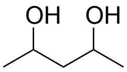

1025

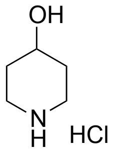

1026

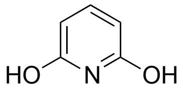

1027

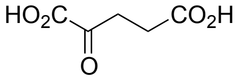

1028

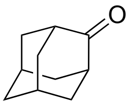

1029

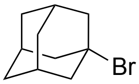

1030

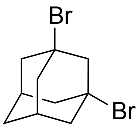

1031

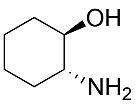

1032

Supporting Figure 3-43 Structure of in-house library compound **1009** - **1032**

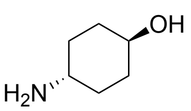

1033

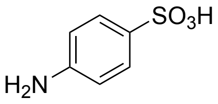

1034

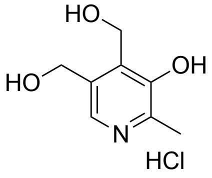

1035

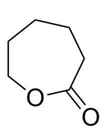

1036

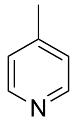

1037

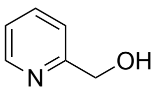

1038

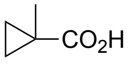

1039

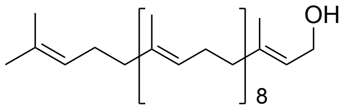

1040

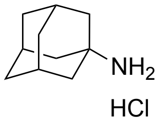

1041

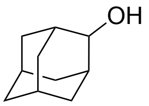

1042

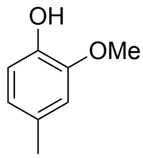

1043

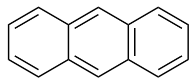

1044

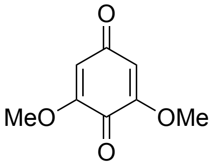

1045

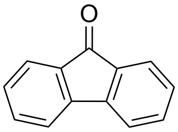

1046

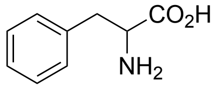

1047

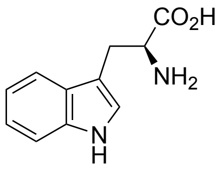

1048

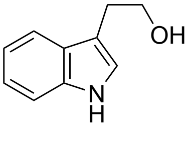

1049

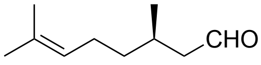

1050

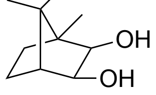

1051

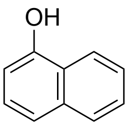

1052

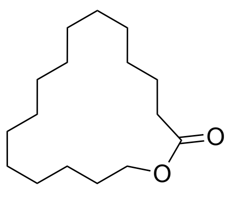

1053

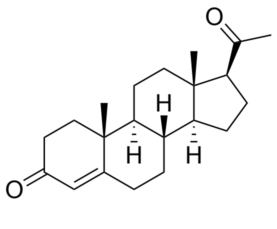

1054

Supporting Figure 3-44 Structure of in-house library compound **1033** - **1053**

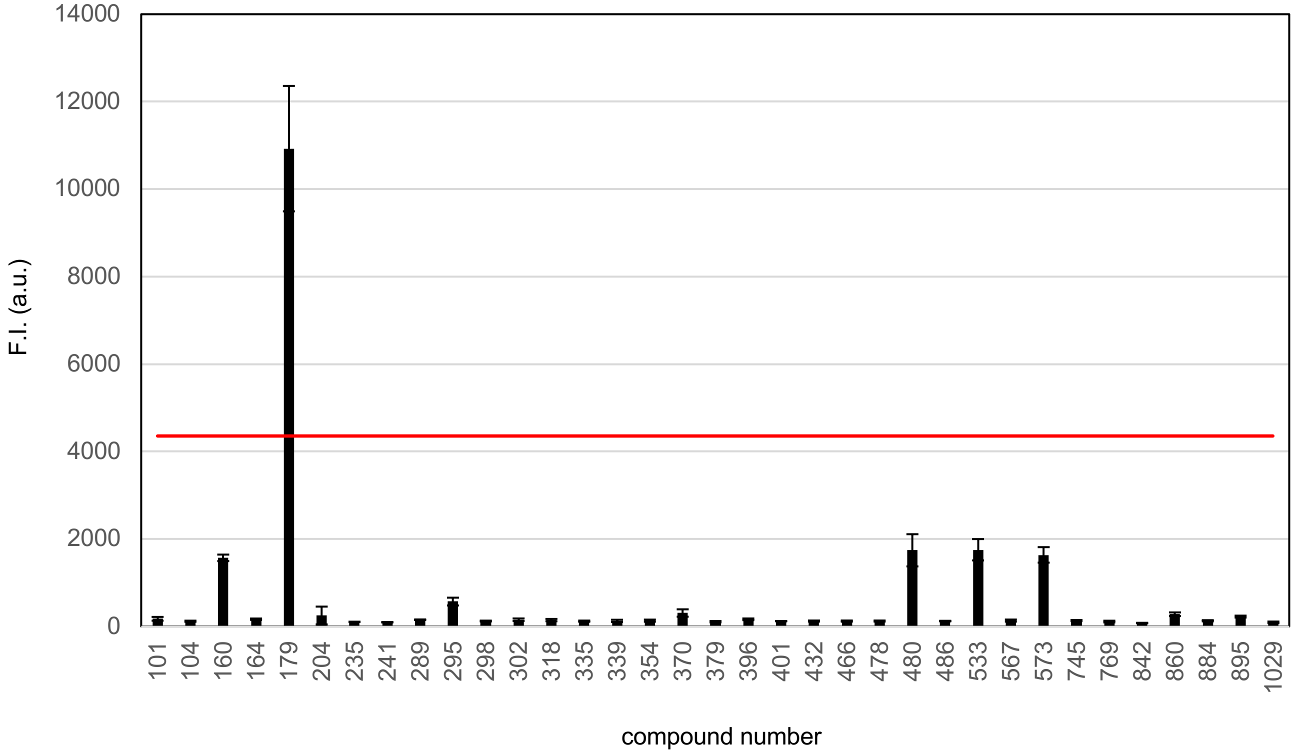

Supporting Figure 4 Results of the second screening of P450 cam substrates by using PF-1 as an indicator (Mean of 3 replicates, error bars show standard deviation). Red line indicates threshold (mean + 2SD). Commpound #769 = D-camphor.

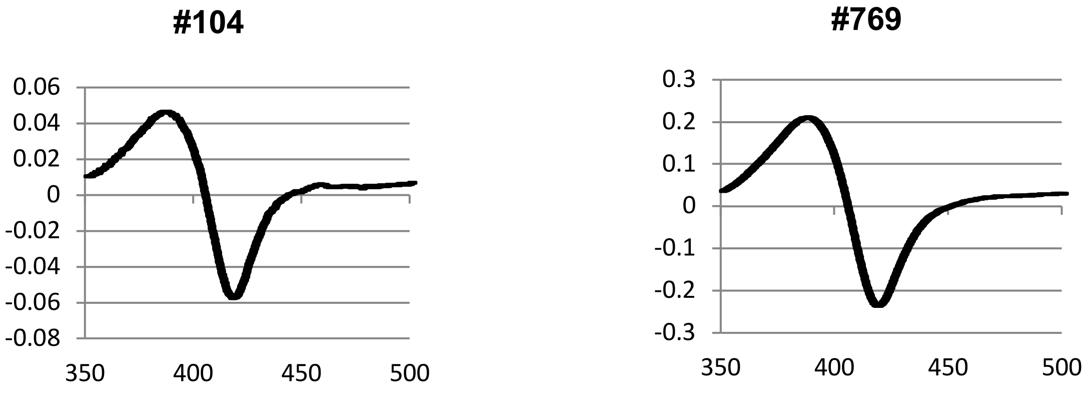

Supporting Figure 5 Substrate-induced spectral changes observed for an addition of a hit compound (10 μM) to P450 cam (3 μM). Y-axis = Δ Absorbance, X-axis = wavelength (nm).

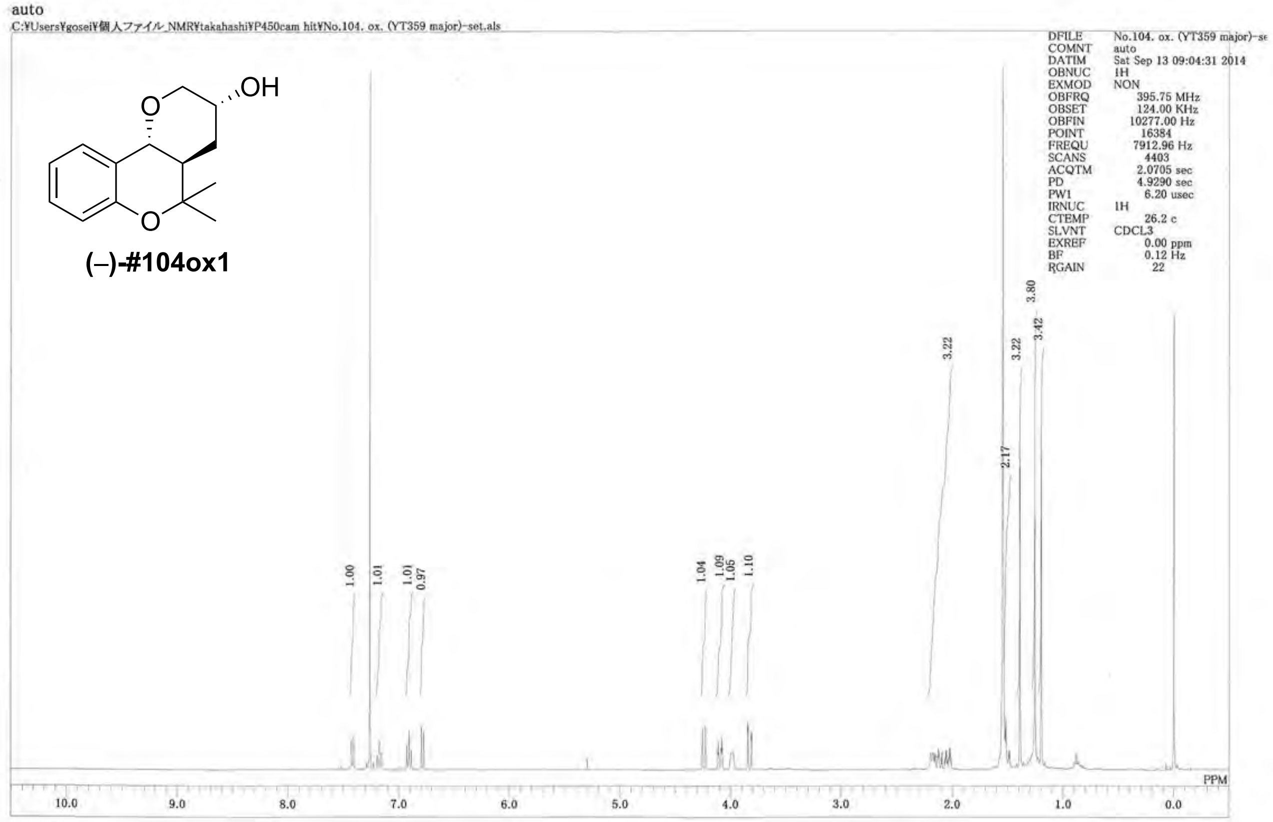

Supporting Figure 6-1 ^{1}H-NMR spectrum of **#104ox1** (400 MHz, $CDCl_3$)

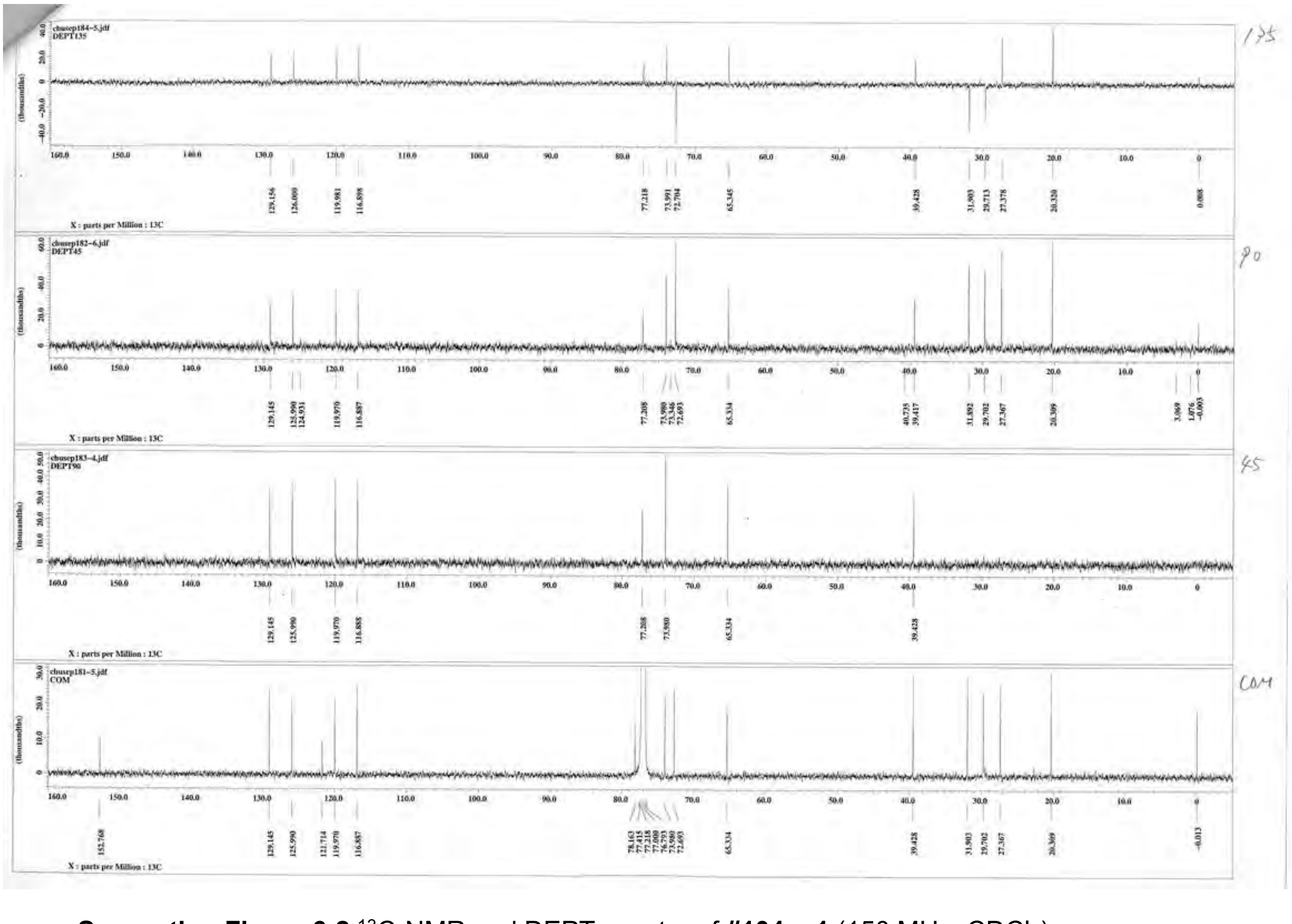

Supporting Figure 6-2 ^{13}C-NMR and DEPT spectra of **#104ox1** (150 MHz, $CDCl_3$)

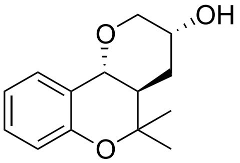

(–)-#104ox1

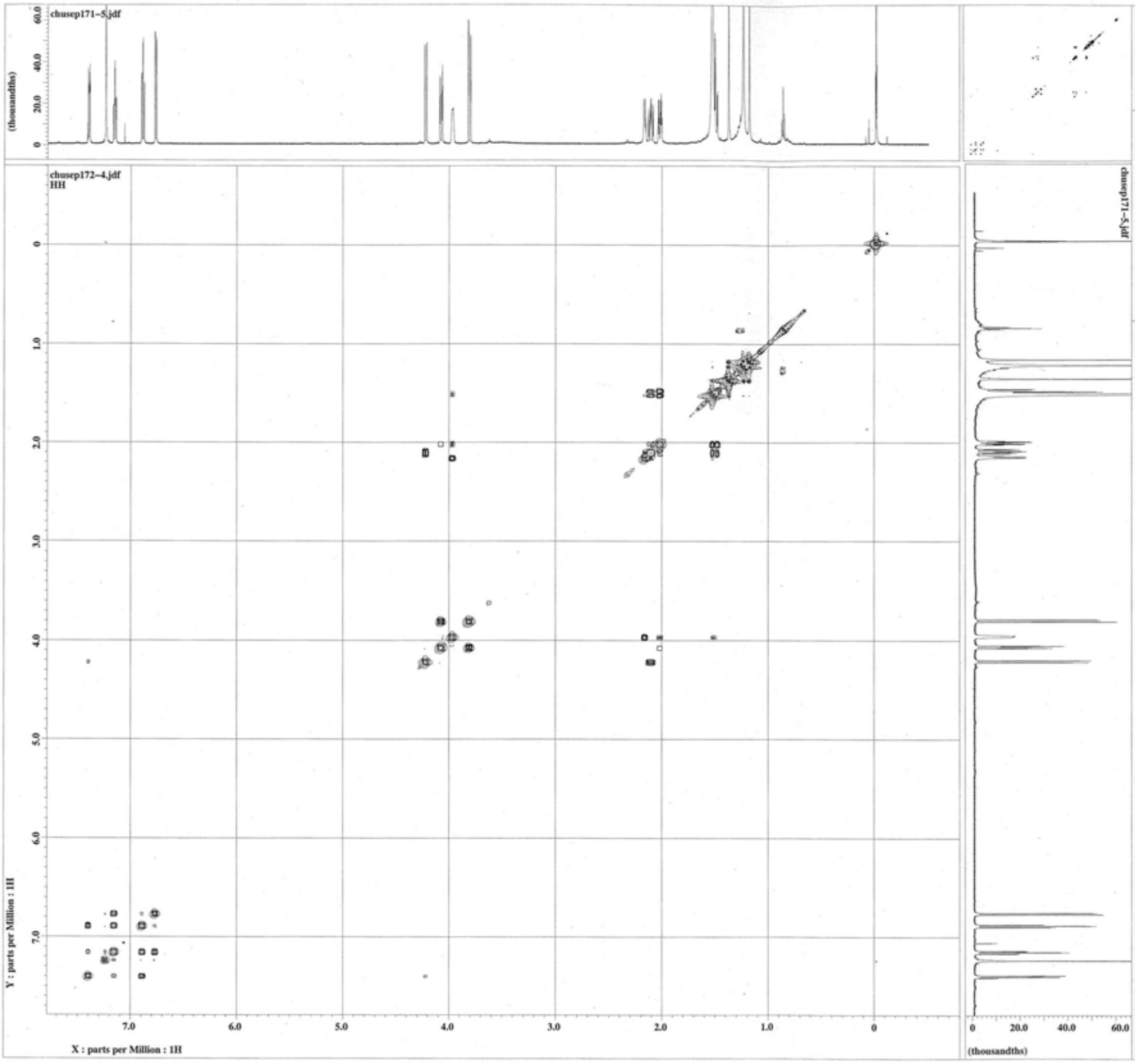

Supporting Figure 6-3 H-H COSY spectrum of **#104ox1** (600 MHz, $CDCl_3$)

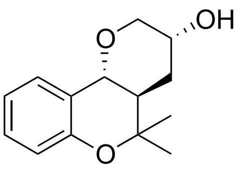

(–)-#104ox1

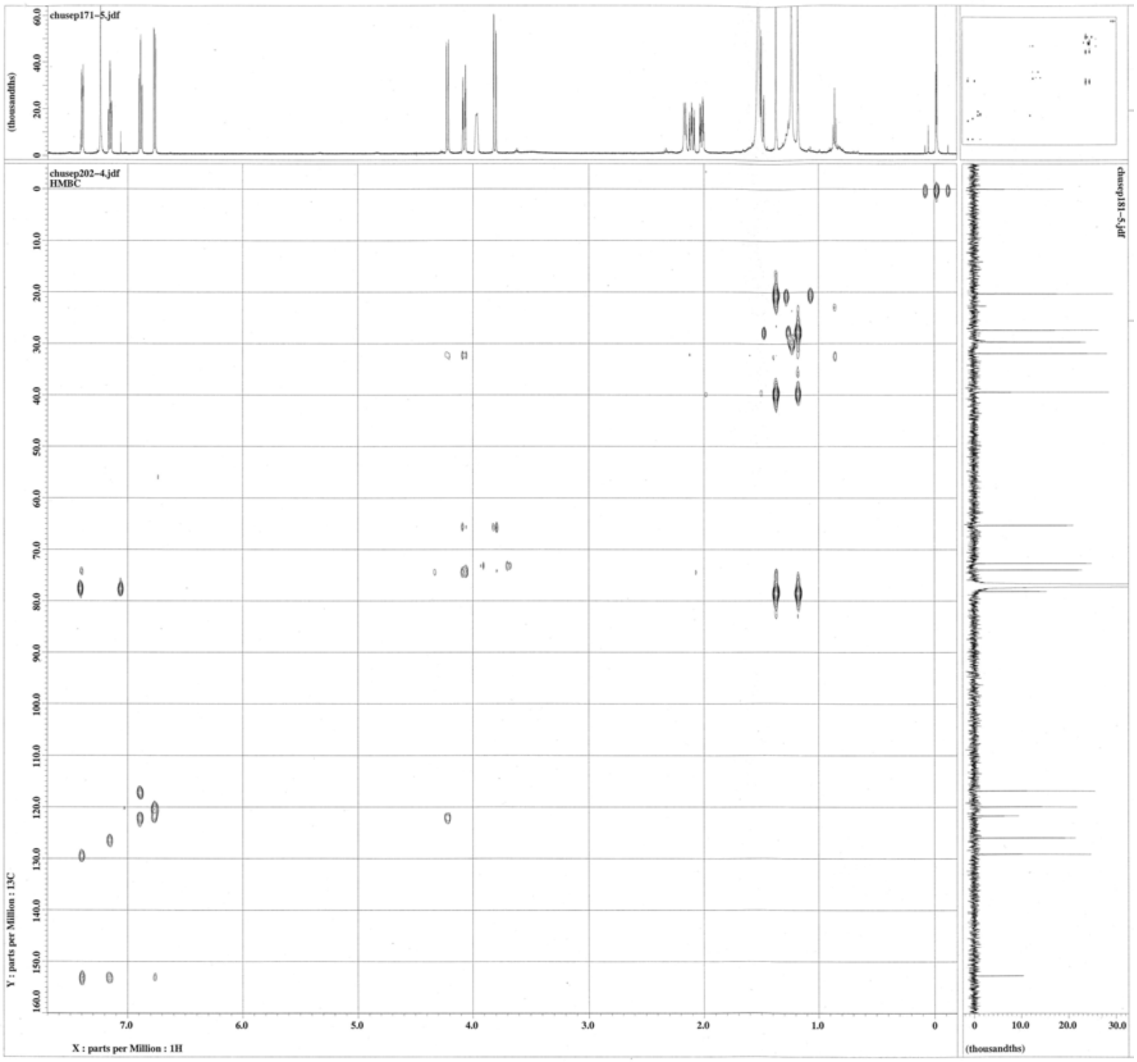

Supporting Figure 6-4 HMBC spectrum of **#104ox1** (600 MHz, $CDCl_3$)

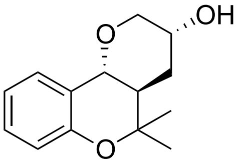

(–)-#104ox1

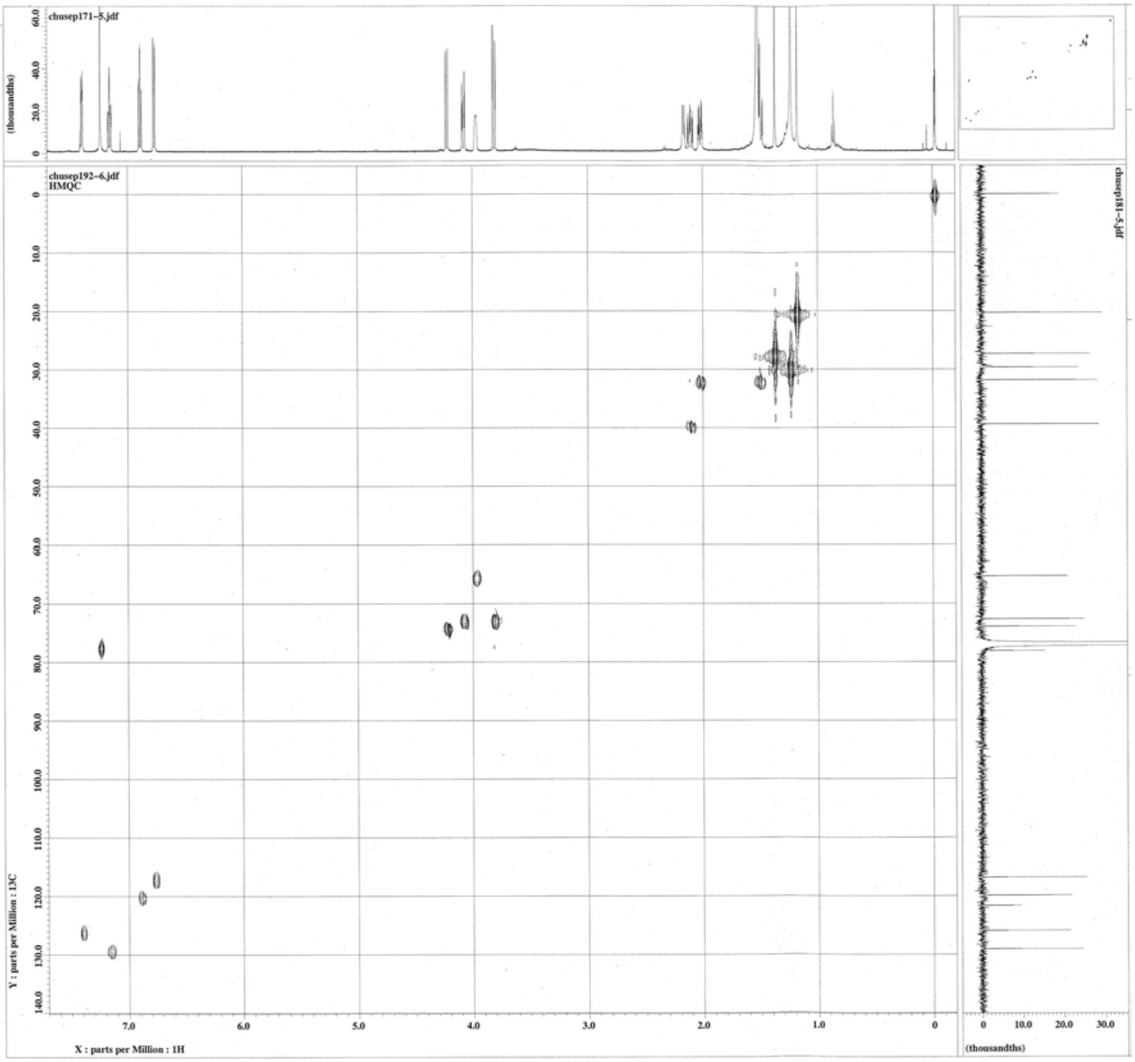

Supporting Figure 6-5 HMQC spectrum of **#104ox1** (600 MHz, $CDCl_3$)

a)

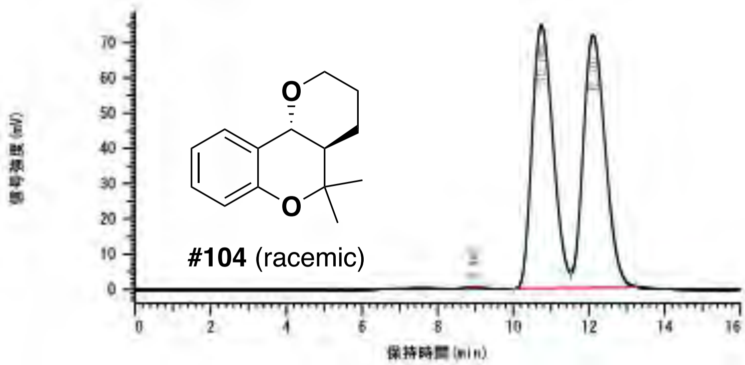

ﾋﾟｰｸ定量: 面積
定量計算方法: 面積%

NO	RT	面積	濃度1	BC
1	8.960	2428	0.042	BB
2	10.743	2860603	49.761	BV
3	12.107	2885705	50.197	VB
		5748736	100.000	

b)

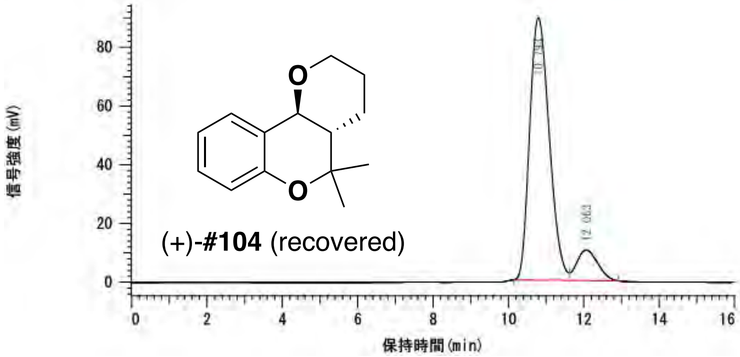

ﾋﾟｰｸ定量: 面積
定量計算方法: 面積%

NO	RT	面積	濃度1	BC
1	10.793	3213968	88.857	BV
2	12.063	403054	11.143	VB
		3617022	100.000	

c)

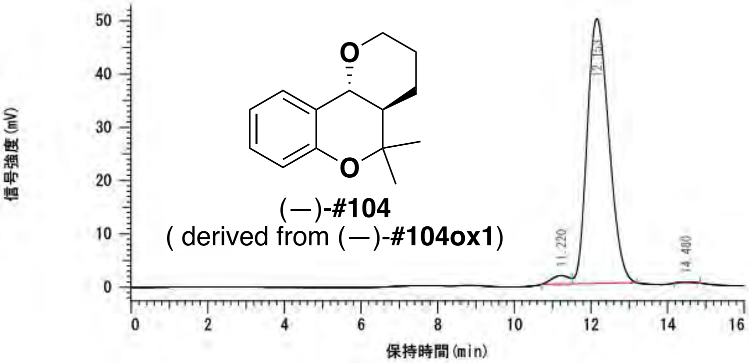

ﾋﾟｰｸ定量: 面積
定量計算方法: 面積%

NO	RT	面積	濃度1	BC
1	11.220	49166	2.456	BV
2	12.153	1946808	97.259	VB
3	14.480	5696	0.285	BB
		2001670	100.000	

Supporting Figure 7-1 Chiral HPLC analysis. (a) Racemic **#104**, (b) (+)-**#104** recovered from P450 cam-mediated oxidation of racemic **#104**, (c) (–)-**#104** derived from (–)-**#104ox1**. See supporting methods for conversion of (–)-**#104ox1** to (–)-**#104**.

a)

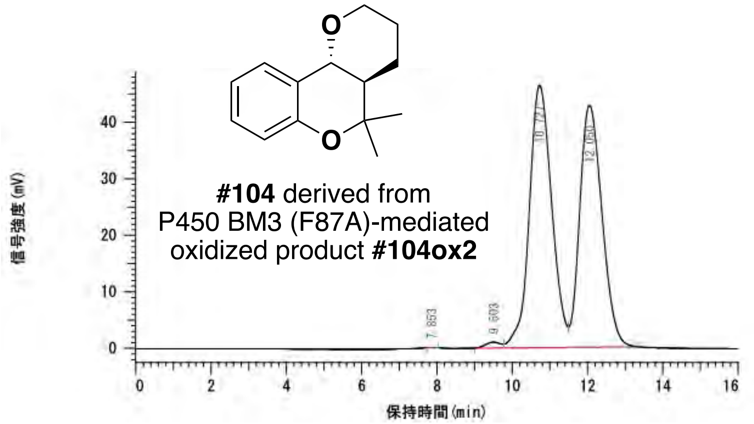

ピークの定量: 面積
定量計算方法: 面積%

NO	RT	面積	濃度1	BC
1	7.853	349	0.009	BB
2	9.503	27781	0.754	BV
3	10.727	1905769	51.695	VV
4	12.050	1752690	47.542	VB
		3686589	100.000	

b)

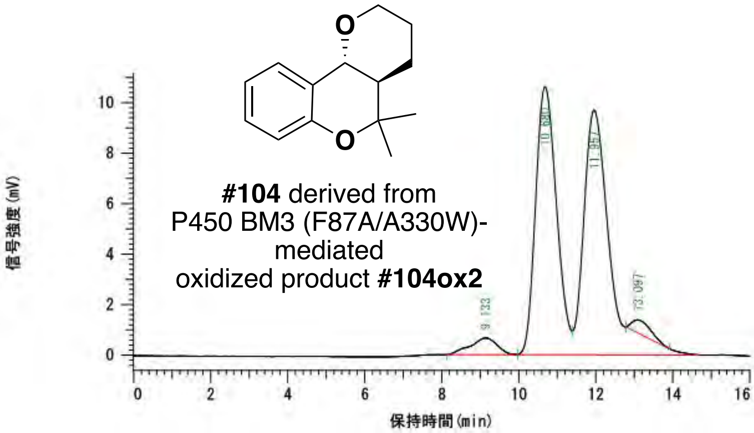

ピークの定量: 面積
定量計算方法: 面積%

NO	RT	面積	濃度1	BC
1	9.133	32442	3.579	BB
2	10.680	399131	44.029	BV
3	11.957	456282	50.333	VV
4	13.097	18665	2.059	TBB
		906520	100.000	

Supporting Figure 7-2 Chiral HPLC analysis. (a) **#104** derived from P450 BM3(F87A)-mediated oxidized product **#104ox2.** (b) **#104** derived from P450 BM3(F87A/A330W)-mediated oxidized product **#104ox2.** See supporting methods for conversion of **#104ox2** to **#104**.

a)

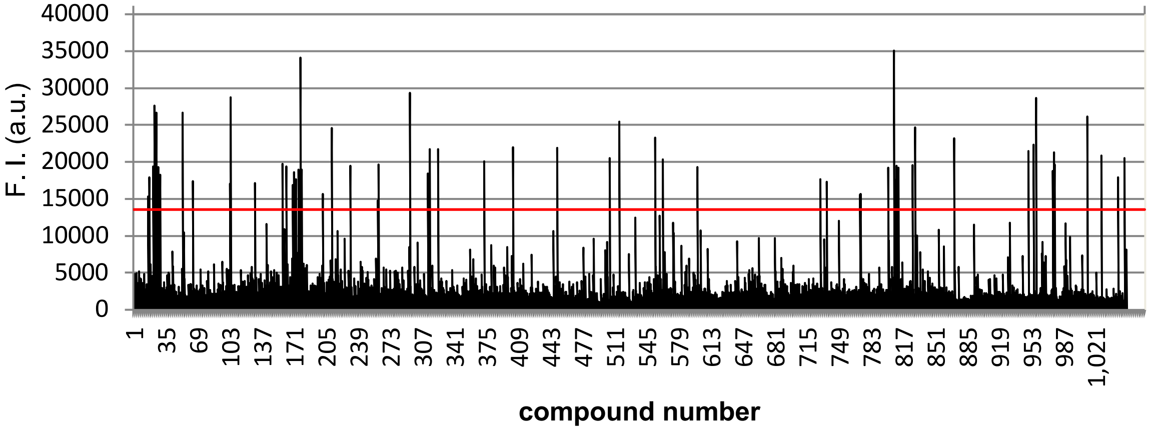

b)

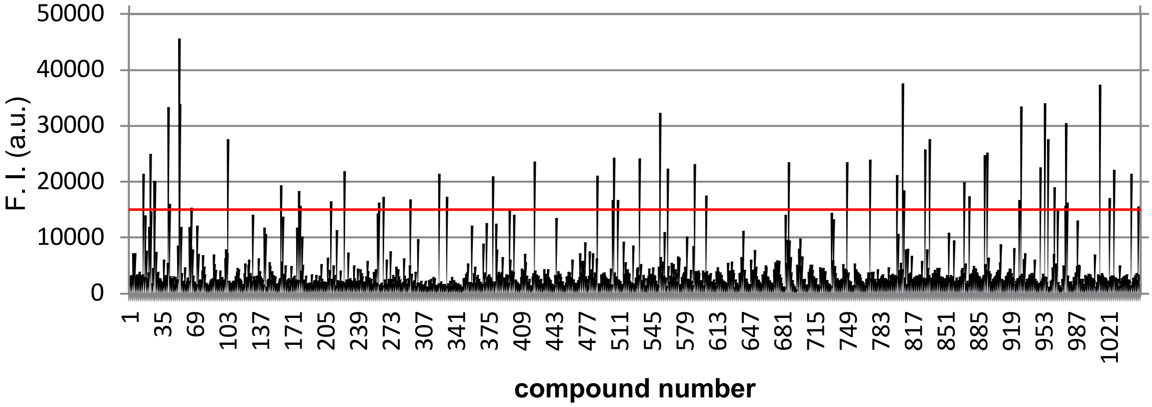

Supporting Figure 8 Results of first screening of P450 substrates by using 2-acetylbenzofuran (2-ABF) method. Compounds in 96 well plates were incubated with NADPH in the presence of (a) P450 BM3 (F87A) or (b) P450 BM3 (F87A/A330W) . Generated $NADP^+$ in each well was reacted with 2-ABF to generate fluorescent products, and their fluorescence was shown (Mean of 2 replicates). Red lines indicate the threshold in each case (mean + 2SD).

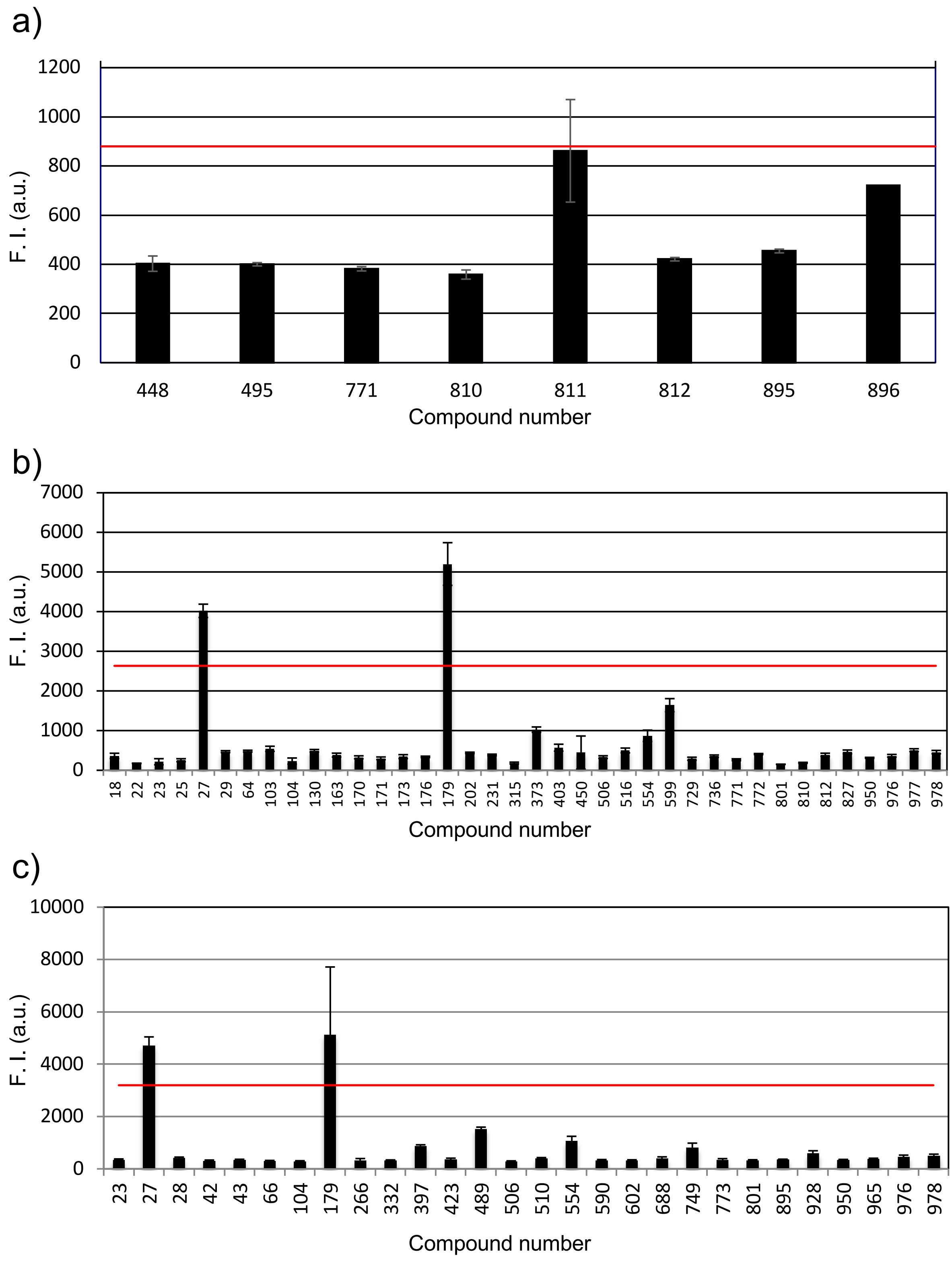

Supporting Figure 9 Results of second screening of P450 substrates by using PF-1. Compounds in 96 well plates were incubated with NADPH and PF-1 in the presence of (a) P450 BM3 (WT) or (b) P450 BM3 (F87A) or (c) P450 BM3 (F87A/A330W) . Generated H_2O_2 in each well was reacted with PF-1 to generate fluorescent products, and their fluorescence was shown (Mean of 3 replicates, error bars show standard deviation). Red lines indicate the threshold in each case (mean + 2SD).

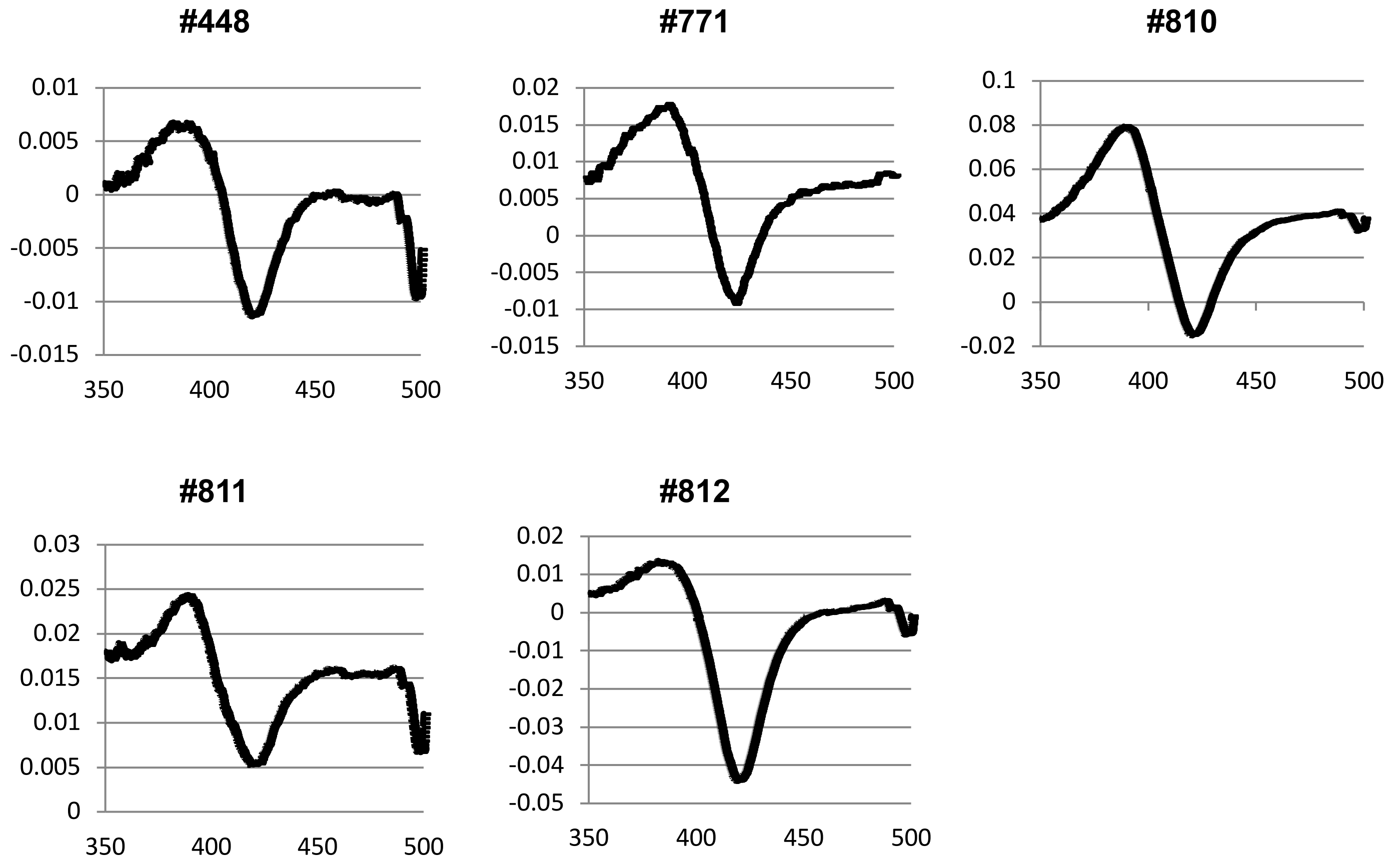

Supporting Figure 10 Substrate-induced spectral changes observed for an addition of the hit compound (10 μM) to P450 BM3 (WT) (3 μM). Y-axis = Δ Absorbance, X-axis = wavelength (nm).

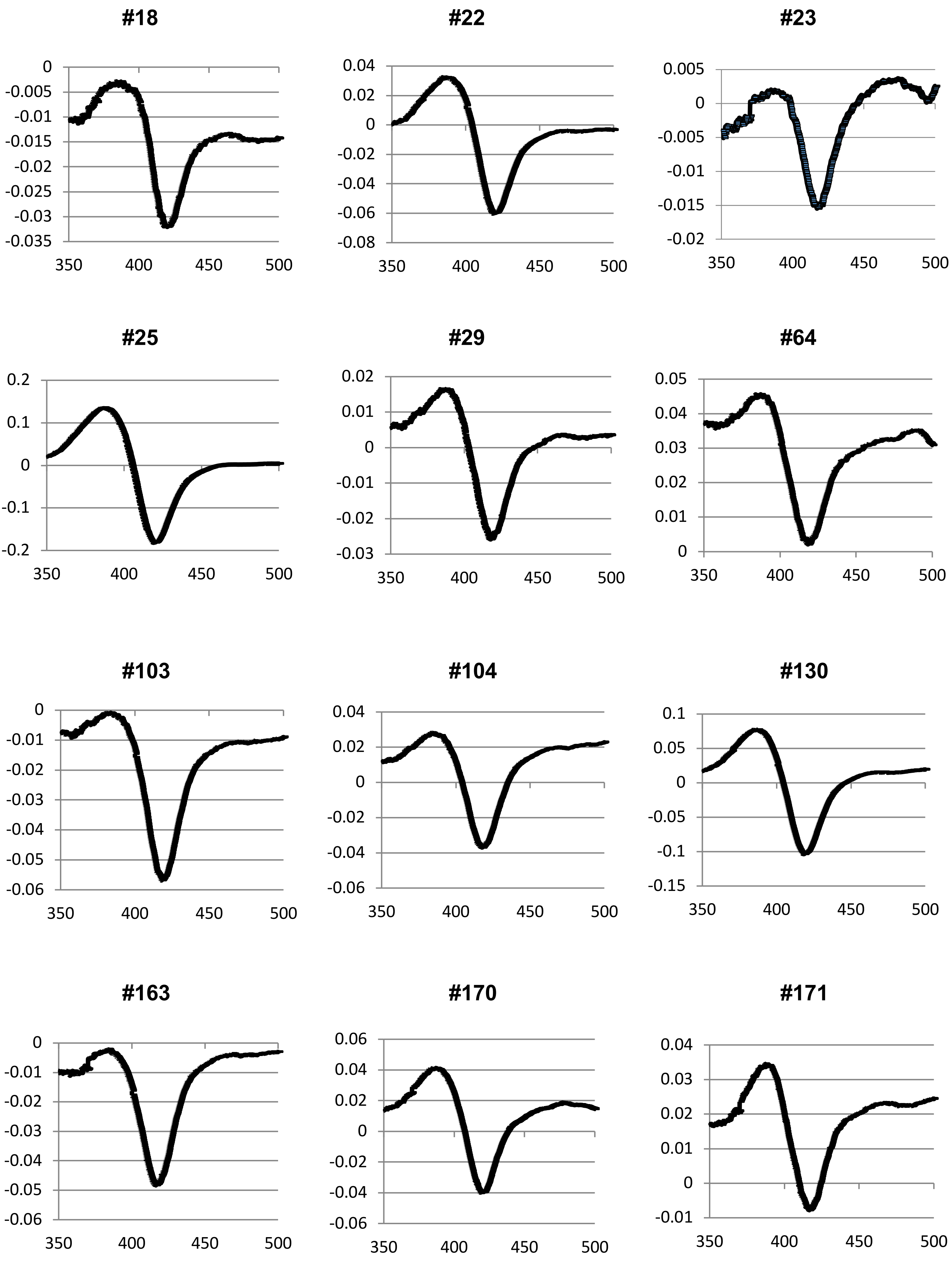

Supporting Figure 11-1 Substrate-induced spectral changes observed for an addition of the hit compound (10 μM) to P450 BM3(F87A) (5 μM). Y-axis = Δ Absorbance, X-axis = wavelength (nm).

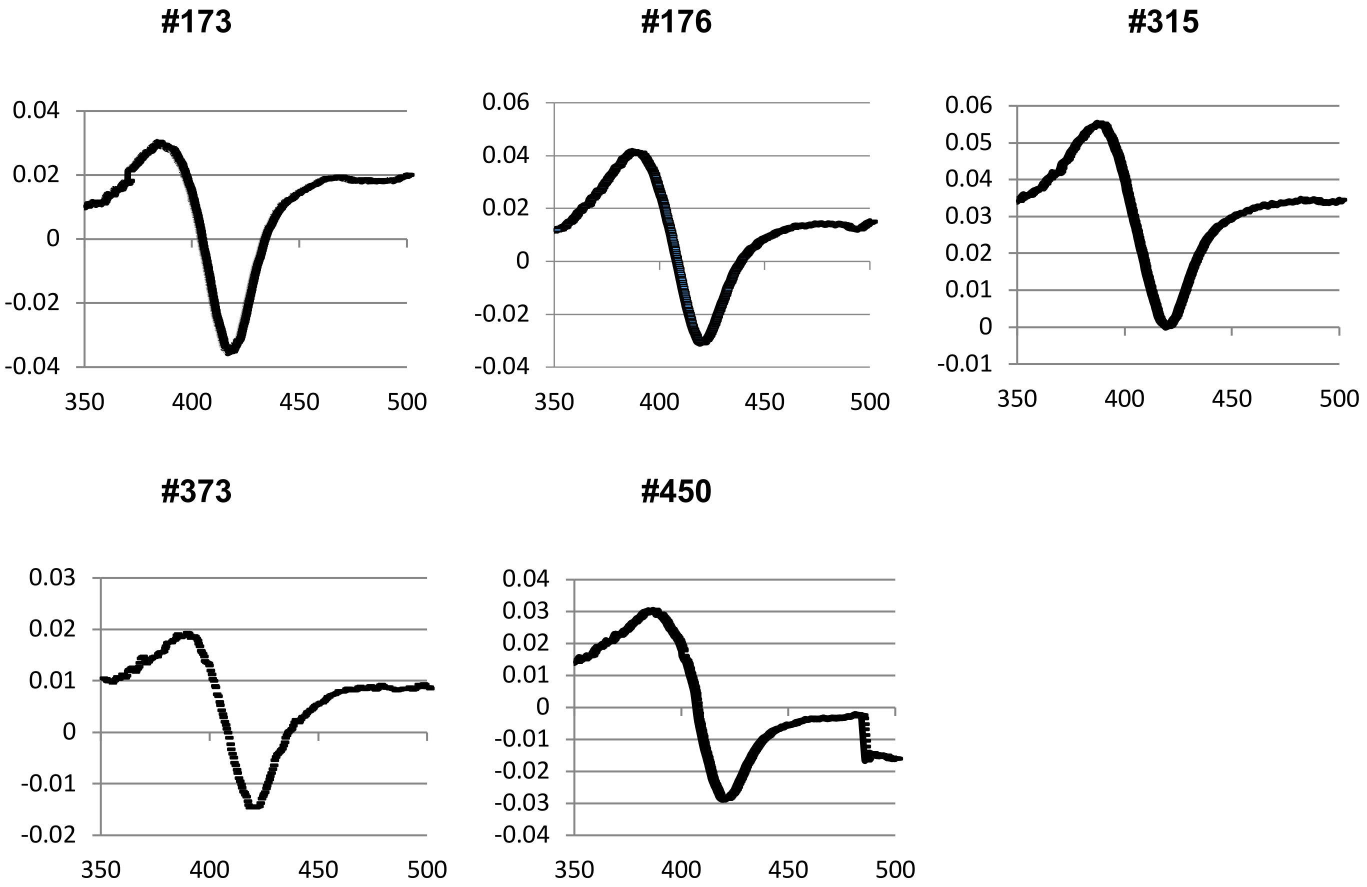

Supporting Figure 11-2 Substrate-induced spectral changes observed for an addition of the hit compound (10 μM) to P450 BM3(F87A) (5 μM) (continued). Y-axis = Δ Absorbance, X-axis = wavelength (nm).

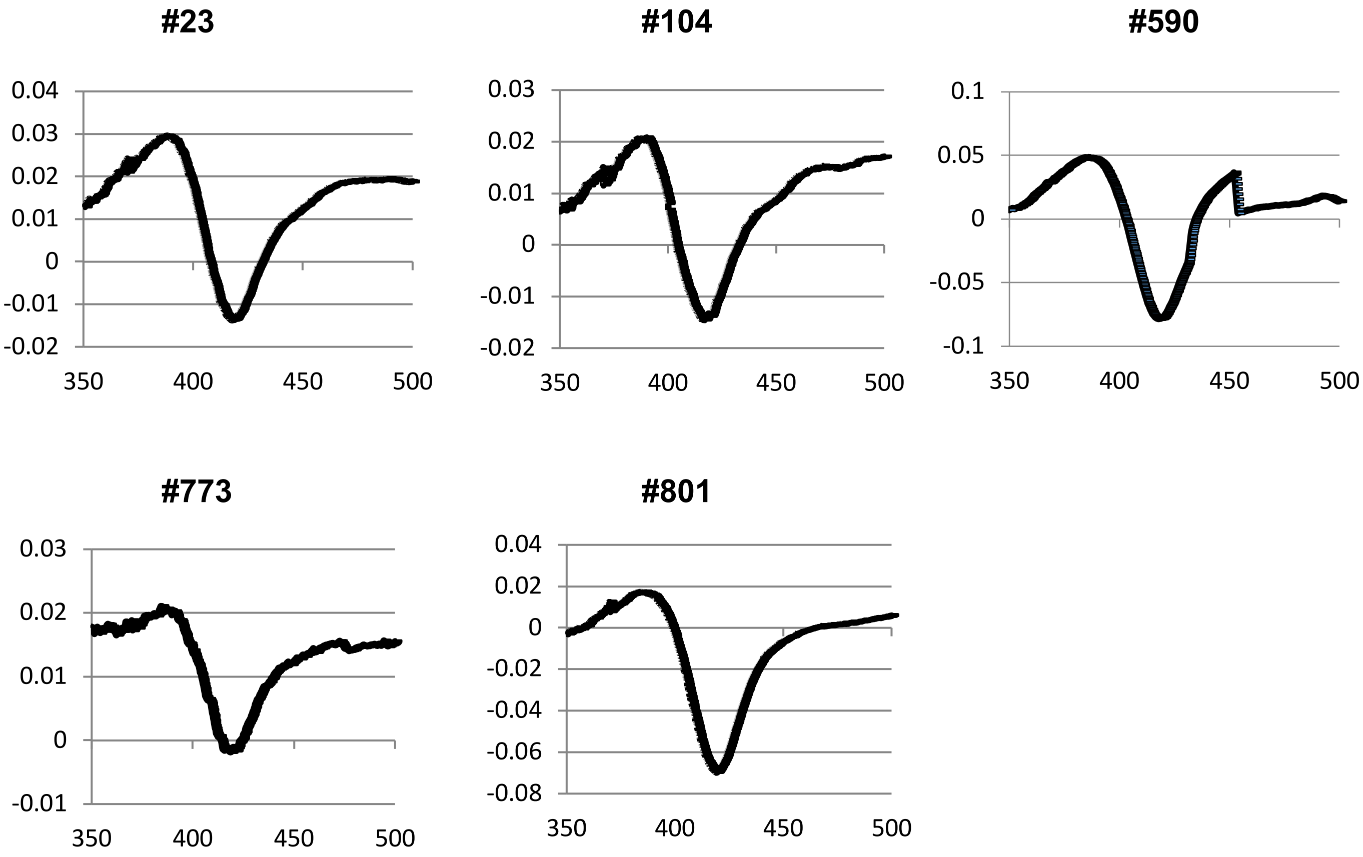

Supporting Figure 12 Substrate-induced spectral changes observed for an addition of the hit compound (10 μM) to P450 BM3(F87A/A330W) (5 μM). Y-axis = Δ Absorbance, X-axis = wavelength (nm).

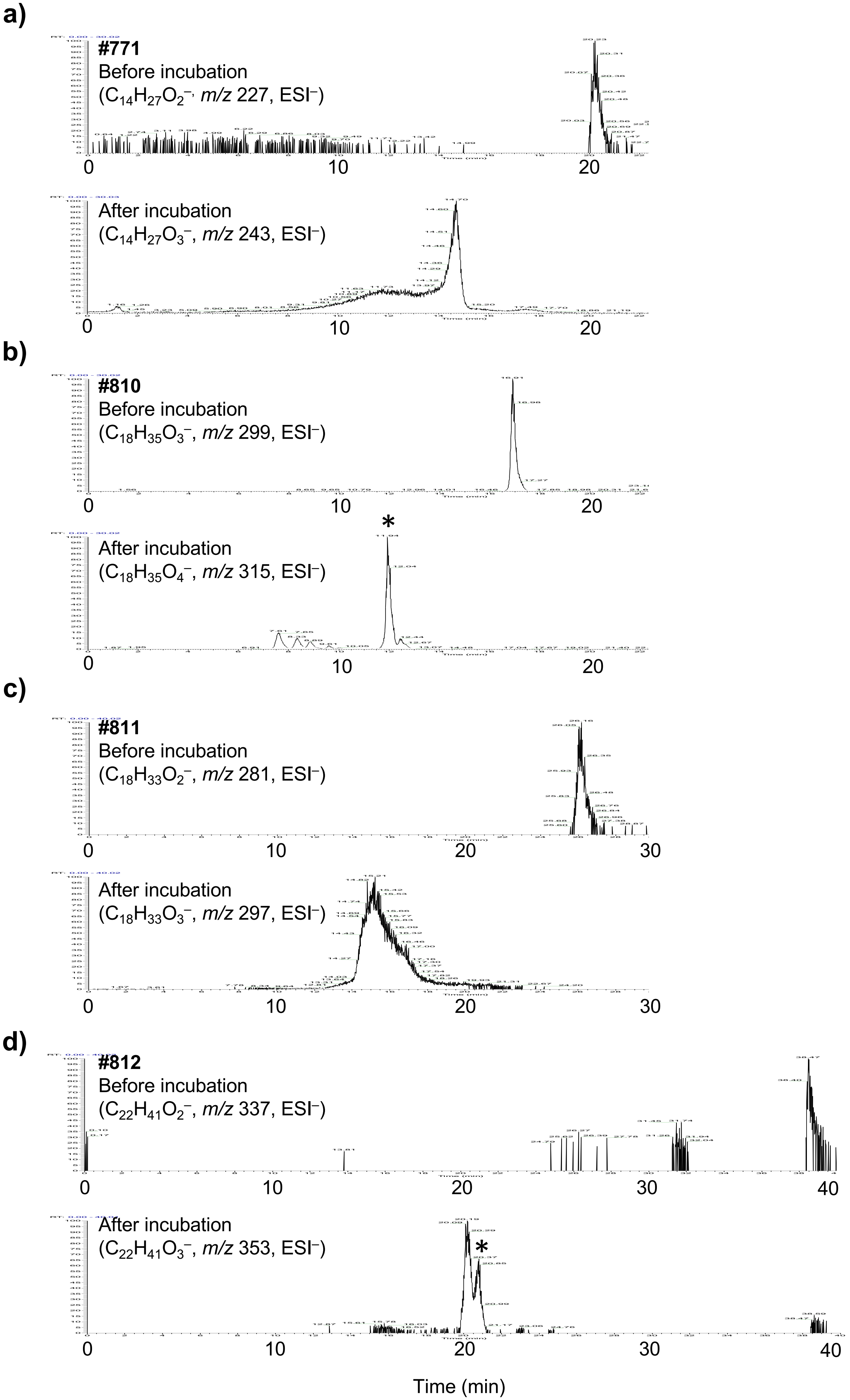

Supporting Figure 13 LC/MS analysis of P450 BM3 (WT)-mediated mono-oxidation products of (a) **#771**, (b) **#810**, (c) **#811**, and (d) **#812.** Asterisks (*) indicate the peaks also found in the starting materials.

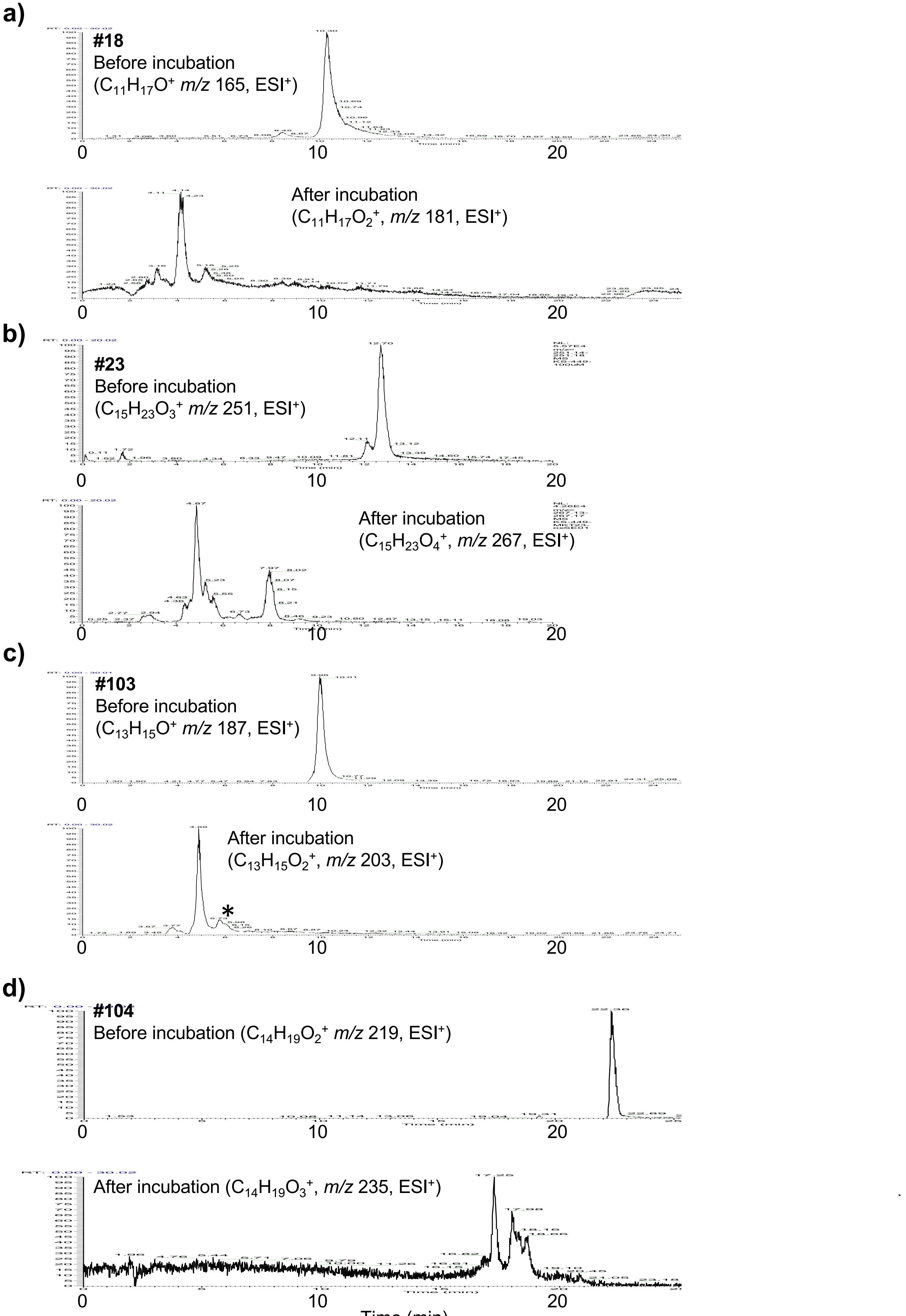

Supporting Figure 14-1 LC/MS analysis of P450 BM3(F87A)-mediated mono-oxidation products of (a) **#18**, (b) **#23**, (c) **#103**, and (d) **#104.** Asterisks (*) indicate the peaks also found in the starting materials.

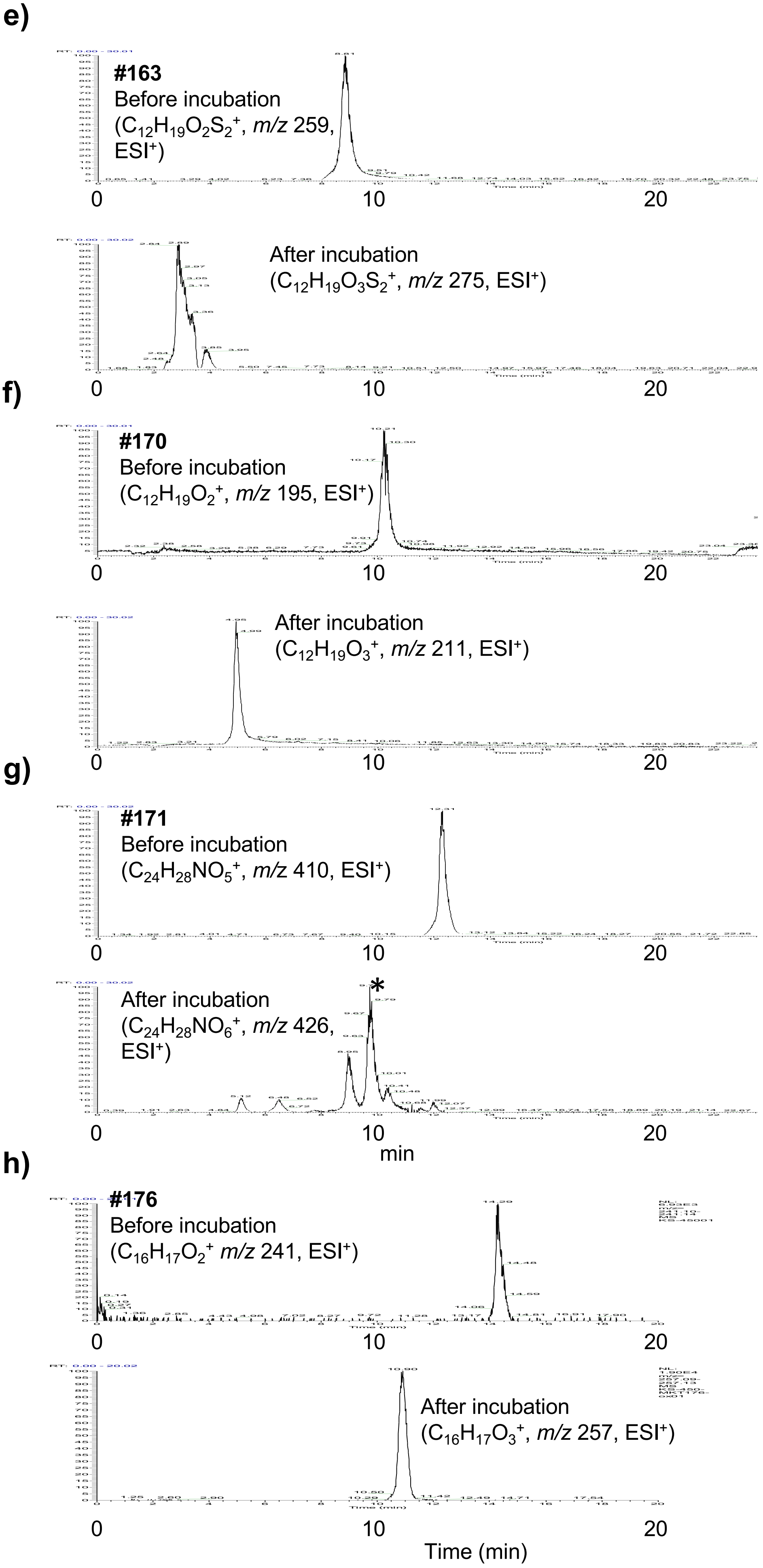

Supporting Figure 14-2 LC/MS analysis of P450 BM3(F87A)-mediated mono-oxidation products of (e) **#163**, (f) **#170**, (g) **#171**, and (h) **#176.** Asterisks (*) indicate the peaks also found in the starting materials.

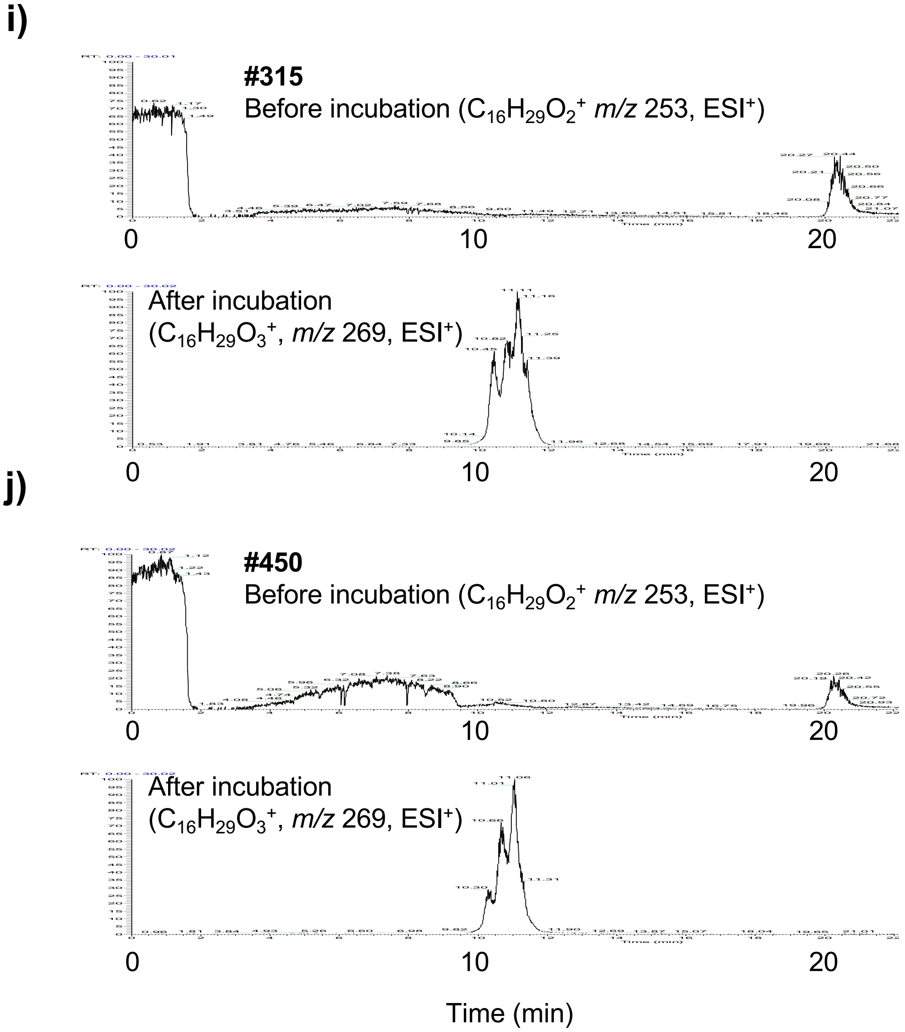

Supporting Figure 14-3 LC/MS analysis of P450 BM3(F87A)-mediated mono-oxidation products of (i) **#315** and (j) **#450**.

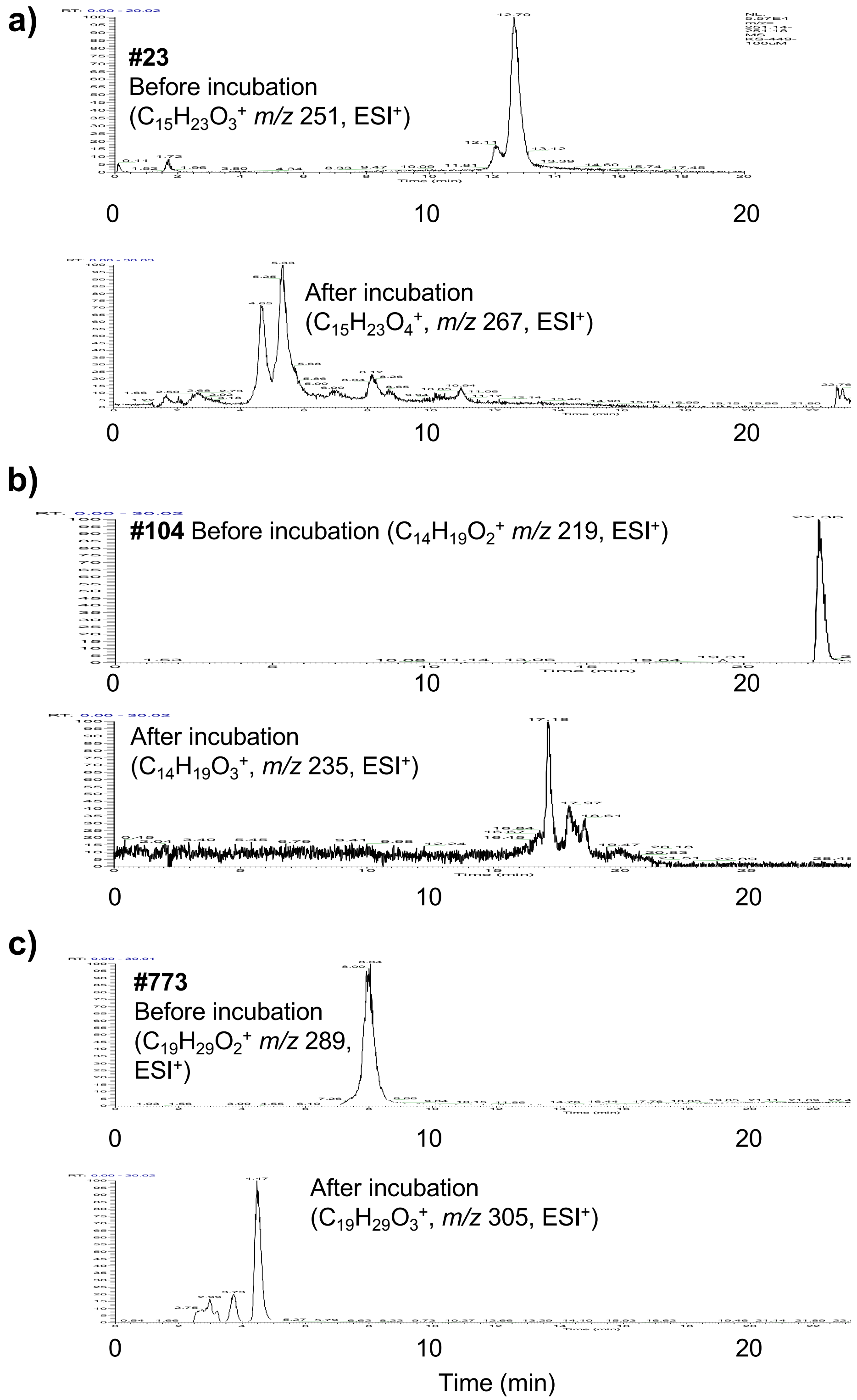

Supporting Figure 15 LC/MS analysis of P450 BM3 (F87A/A330W)-mediated mono-oxidation products of (a) **#23**, (b) **#104**, and (c) **#773**.

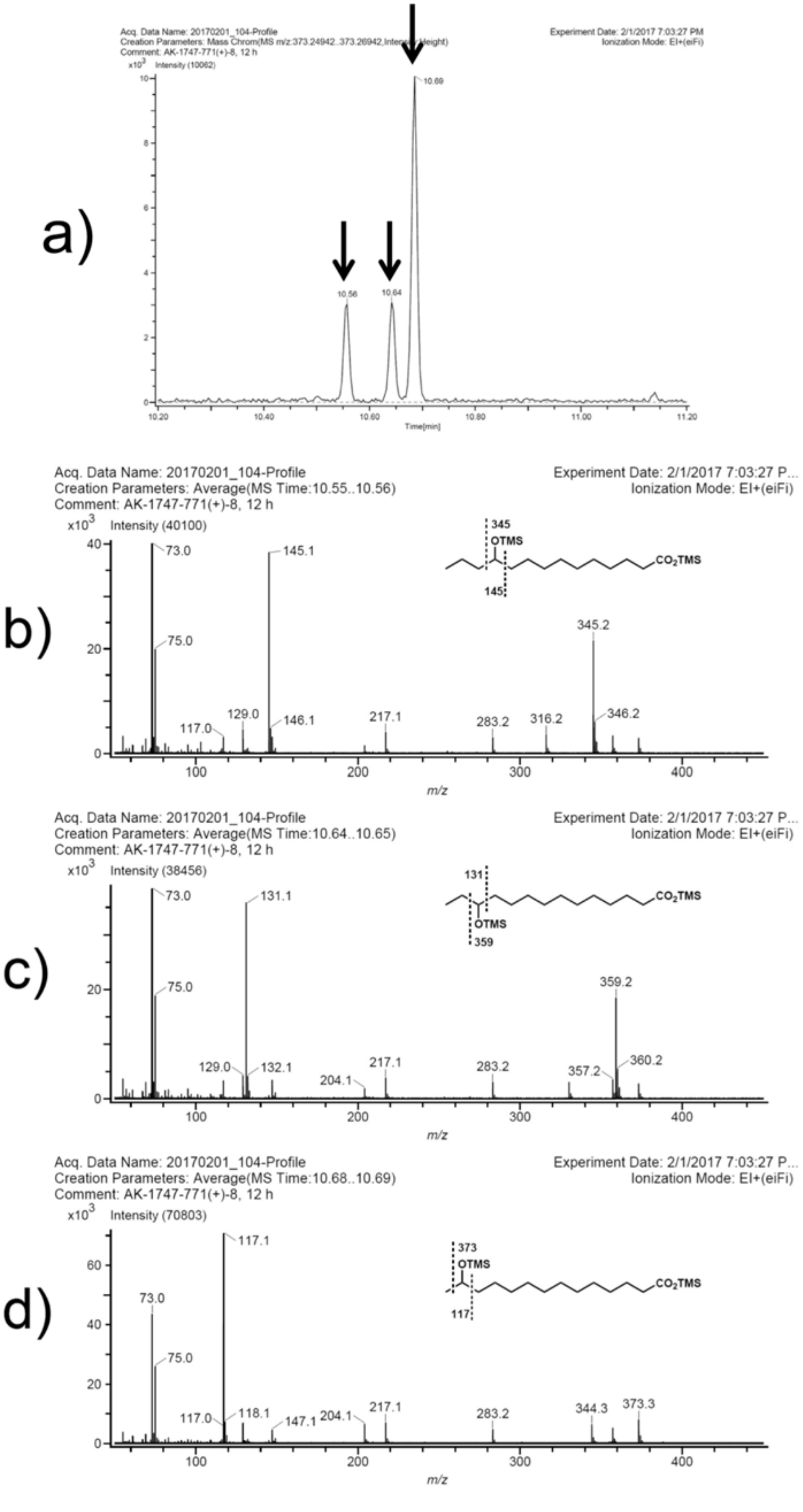

Supporting Figure 16 GC/MS analysis of P450 BM3(WT)-mediated monooxidation products of compound **#771**. (a) Accurate mass chromatogram for $[M-15]^+$ of the disilylated products (*m/z* 373.259). Black arrows indicate peaks increased with incubation time. (b-d) EI mass spectrum at (b) t_R 10.56 min, (c) 10.64 min, and (d) 10.69 min.

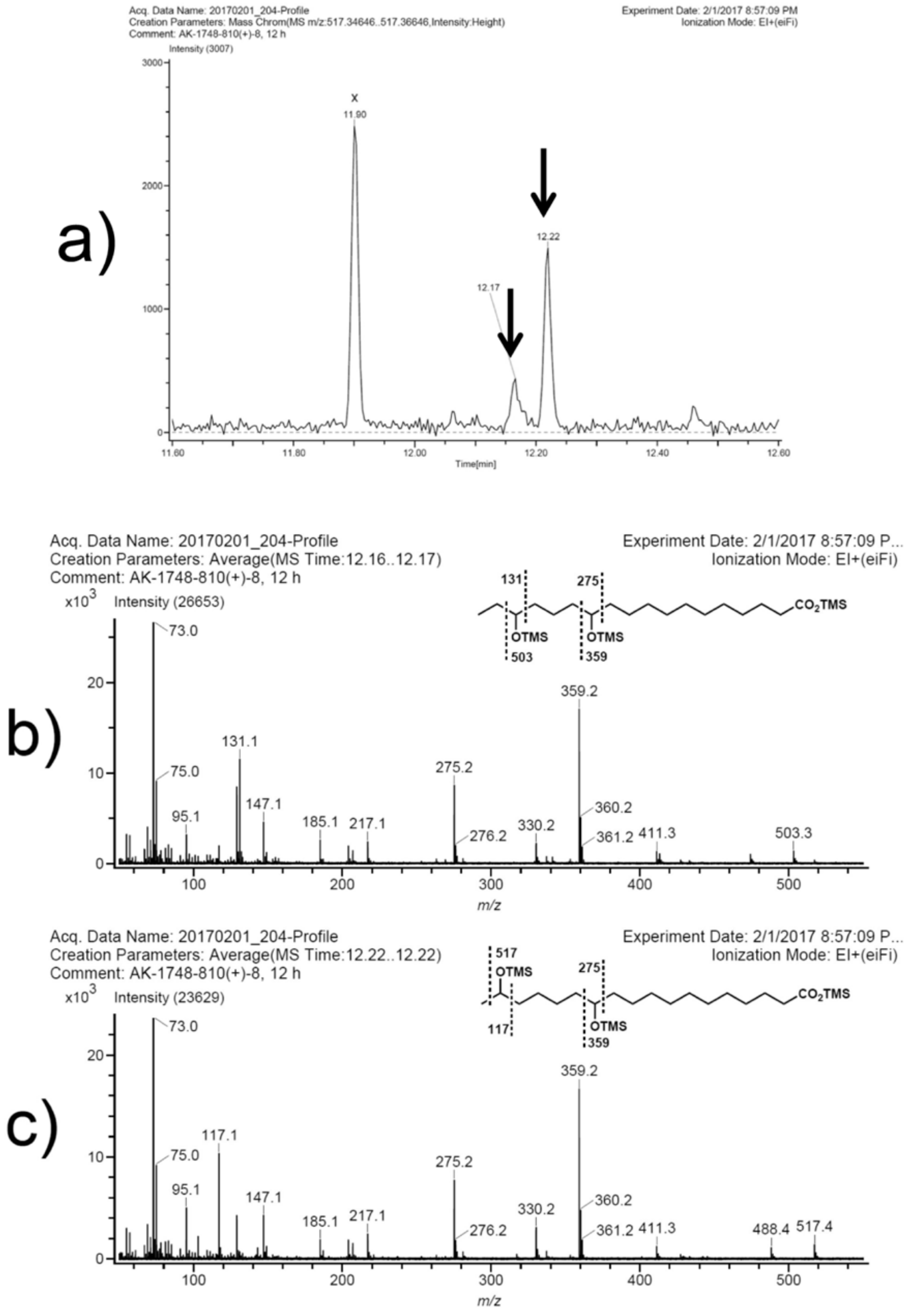

Supporting Figure 17 GC/MS analysis of P450 BM3 (WT)-mediated monooxidation products of compound **#810**. (a) Accurate mass chromatogram for $[M-15]^+$ of the trisilylated products (*m/z* 517.356). Black arrows indicate peaks increased with incubation time. A peak marked with “x” in panel (a) is an unknown component that did not increased during the time-course experiment thus is not assigned to be a monooxidation product. (b-c) EI mass spectrum at (b) t_R 12.17 min, and (c) 12.22 min.

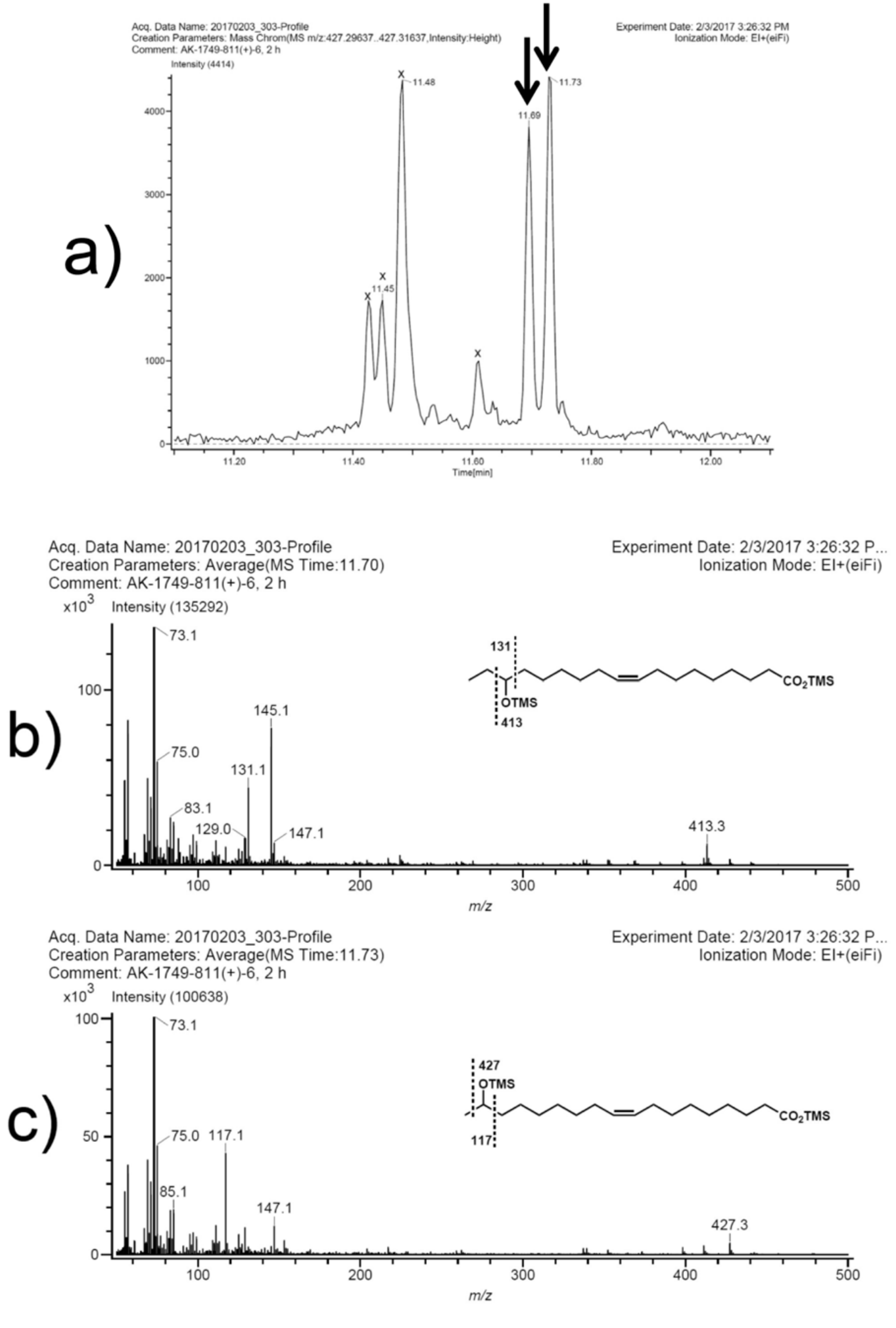

Supporting Figure 18 GC/MS analysis of P450 BM3(WT)-mediated monooxidation products of compound **#811**. (a) Accurate mass chromatogram for $[M-15]^+$ of the disilylated products (*m/z* 427.306). Black arrows indicate peaks increased with incubation time. Peaks marked with “x” in panel (a) are unknown components that did not increased during the time-course experiment thus are not assigned to be monooxidation products. (b-c) EI mass spectrum at (b) t_R: 11.69 min and (c) 11.73 min.

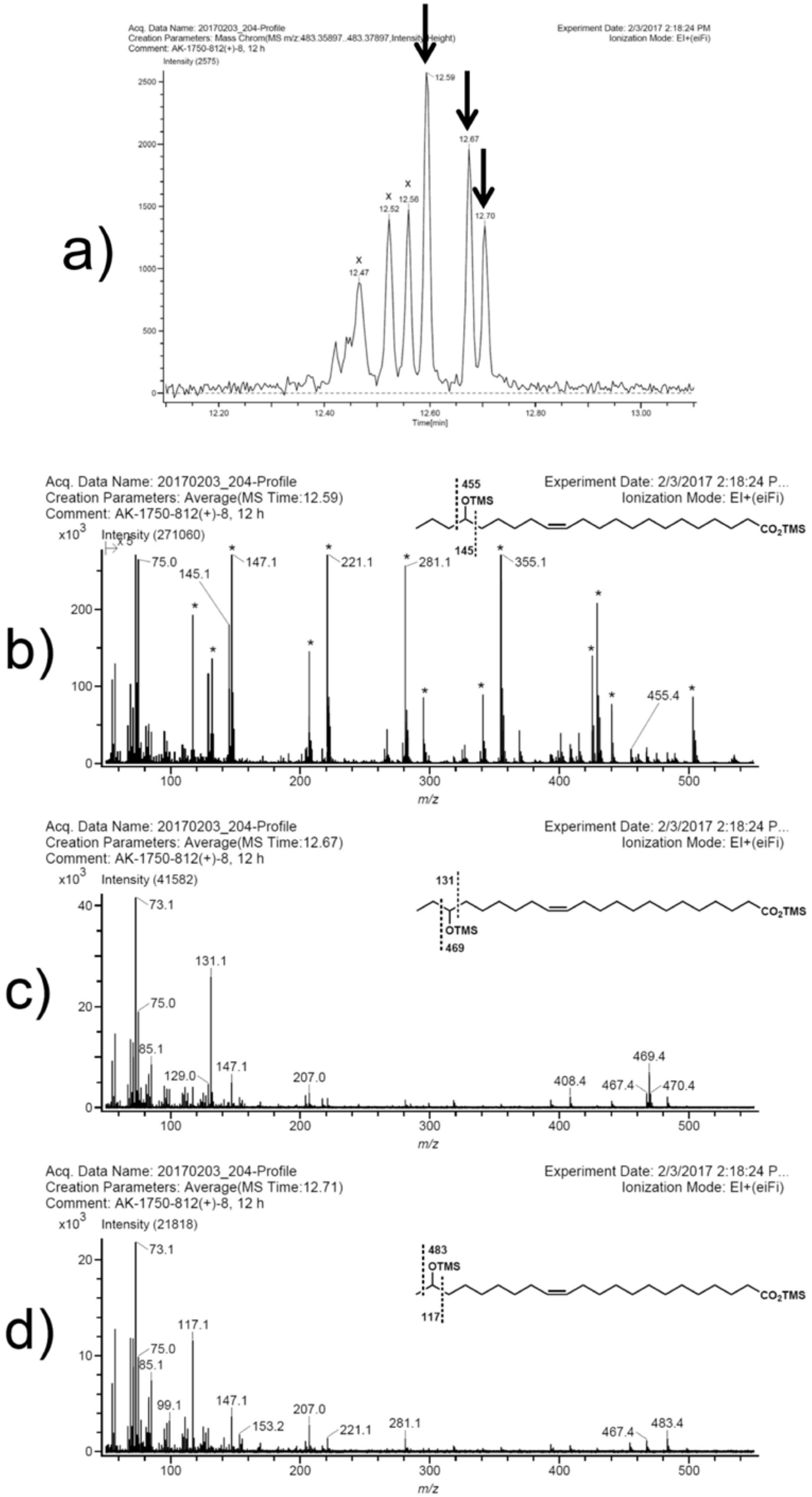

Supporting Figure 19 GC/MS analysis of P450 BM3(WT)-mediated monooxidation products of compound **#812**. (a) Accurate mass chromatogram for $[M-15]^+$ of the disilylated products (*m/z* 483.369). Black arrows indicate peaks increased with incubation time. Peaks marked with "x" in panel (a) are unknown components that did not increased during the time-course experiment thus are not assigned to be monooxidation products.(b-d) EI mass spectrum at (b) t_R: 12.59 min, (c) 12.67 min, and (d) 12.70 min. Peaks marked with small asterisk in panel (b) are ions attributable to unknown co-eluting compound(s).

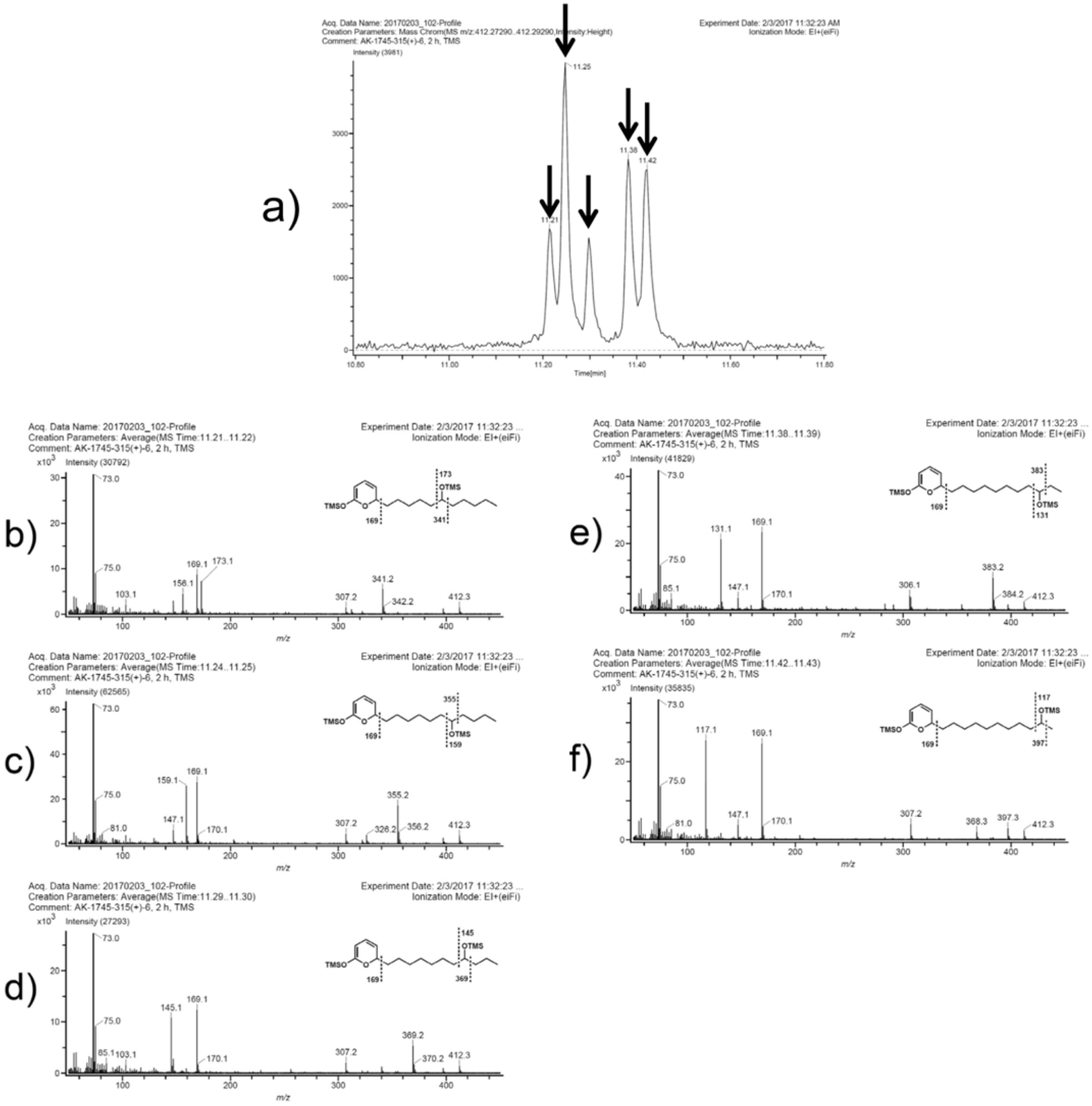

Supporting Figure 20 GC/MS analysis of P450 BM3(WT)-mediated monooxidation products of compound **#315**. (a) Accurate mass chromatogram for $M^{+\bullet}$ of the disilylated products (*m/z* 412.283). Black arrows indicate peaks increased with incubation time. (b-f) EI mass spectrum at (b) t_R: 11.21 min, (c) 11.25 min, (d) 11.30 min, (e) 11.38 min, and (f) 11.42 min.

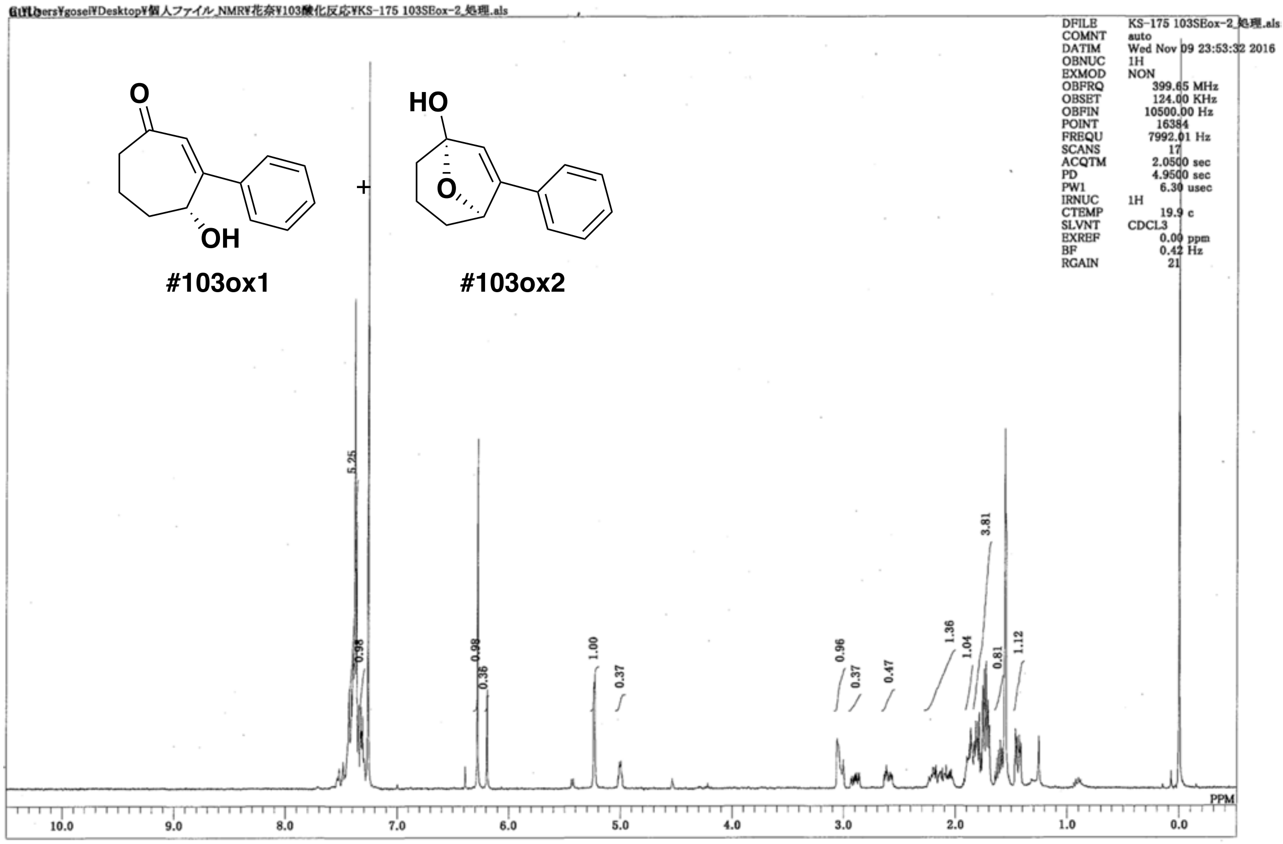

Supporting Figure 21 ^{1}H-NMR spectrum of an equilibrium mixture of **#103ox**1 and **#103ox2** (400 MHz, $CDCl_3$)

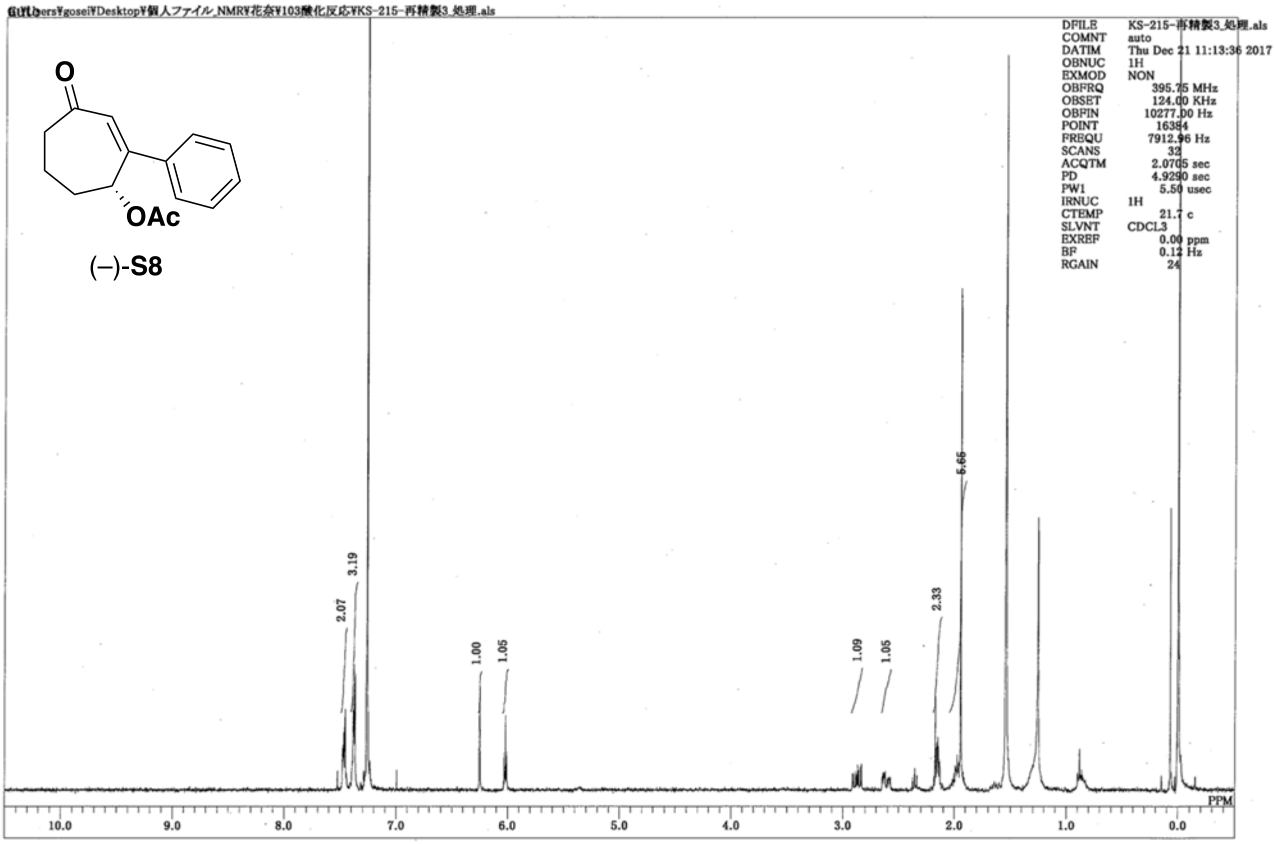

Supporting Figure 22-1 ^{1}H-NMR spectrum of (–)-**S8** (400 MHz, $CDCl_3$)

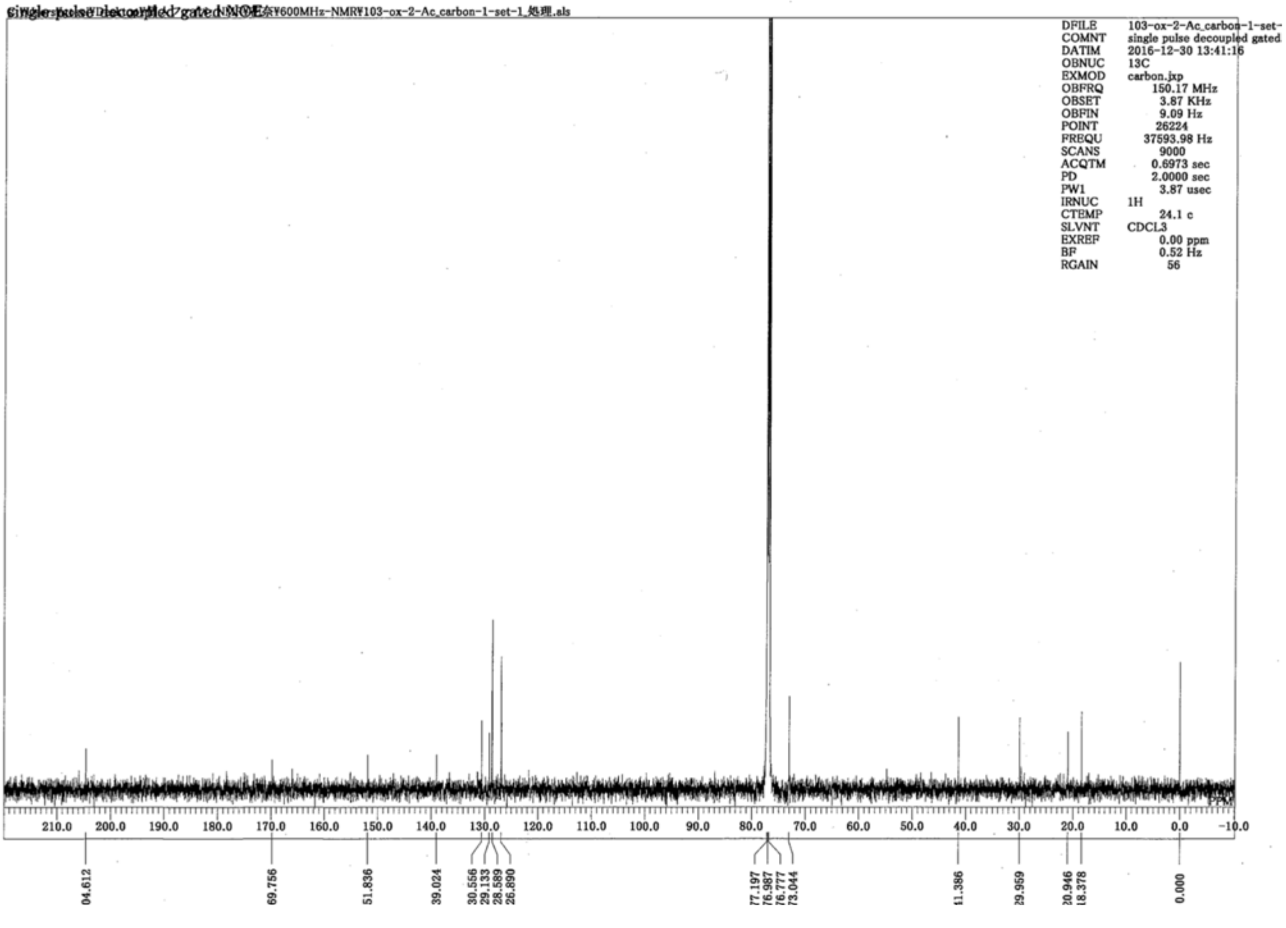

Supporting Figure 22-2 ^{13}C-NMR spectra of (–)-**S8** (150 MHz, $CDCl_3$)

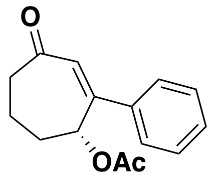

(–)-**S8**

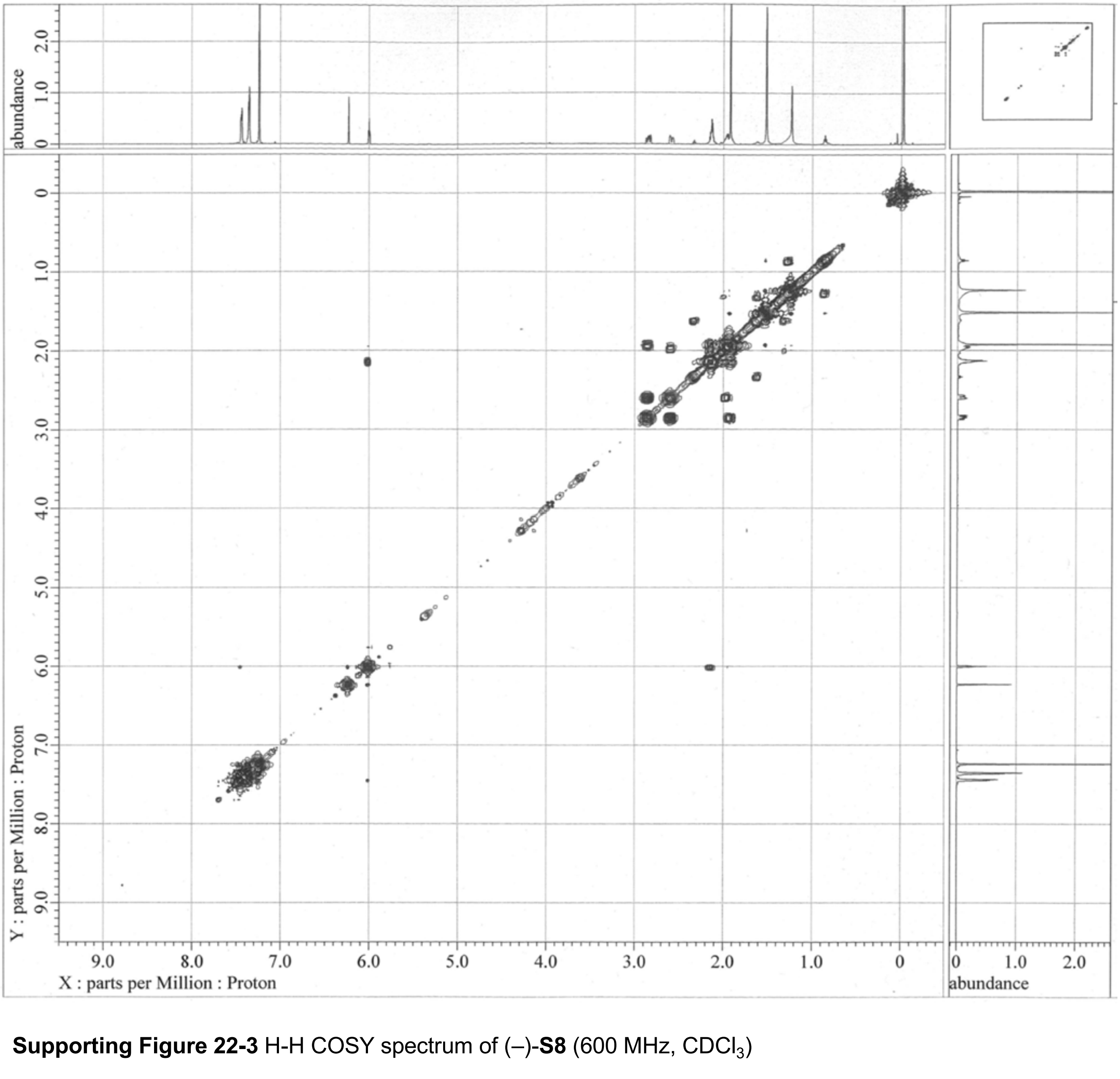

Supporting Figure 22-3 H-H COSY spectrum of (–)-**S8** (600 MHz, $CDCl_3$)

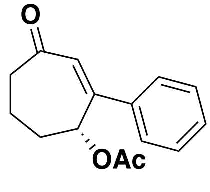

(–)-**S8**

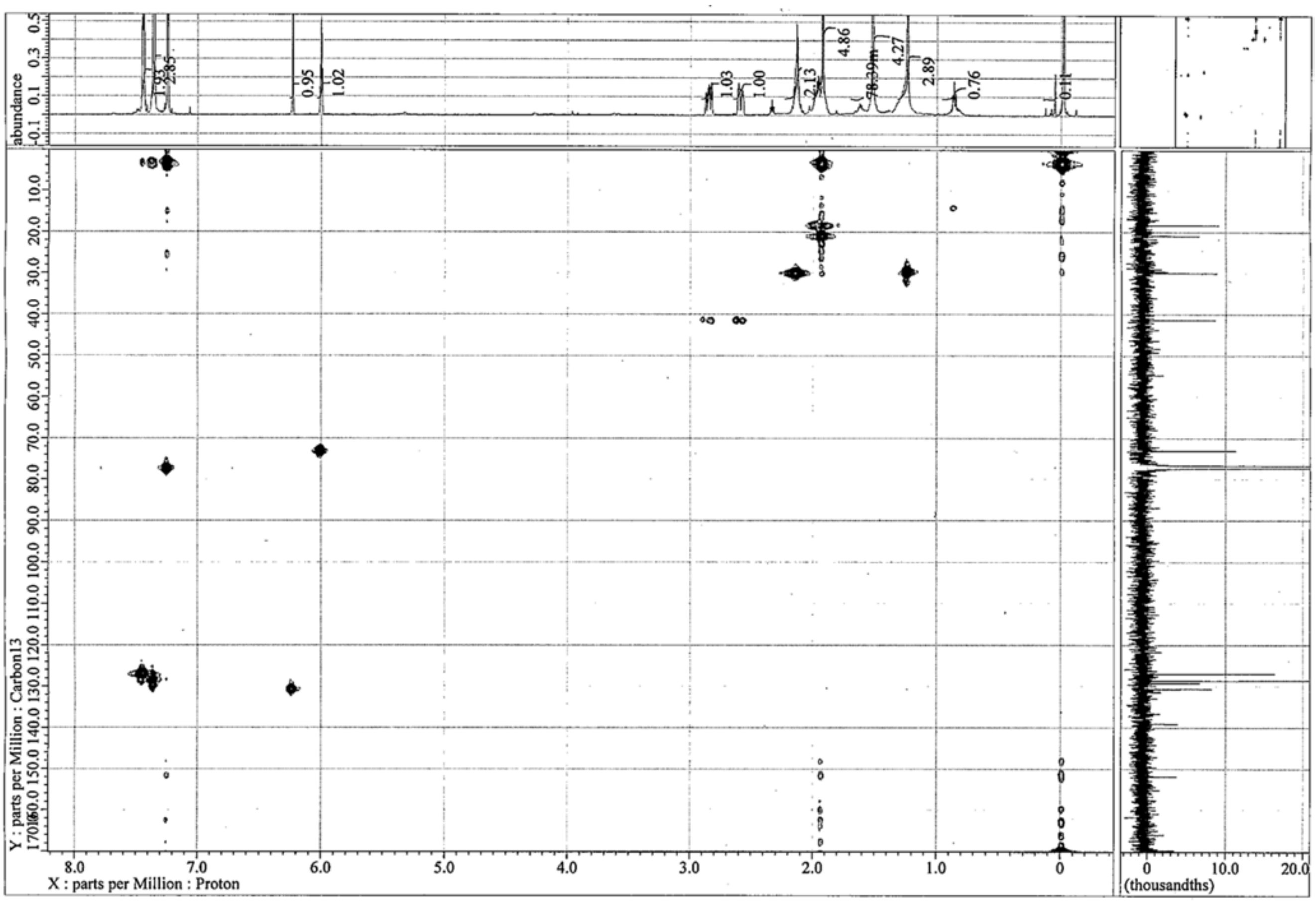

Supporting Figure 22-4 HMQC spectrum of (–)-**S8** (600 MHz, $CDCl_3$)

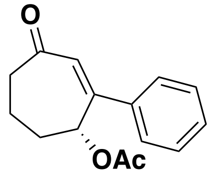

(–)-**S8**

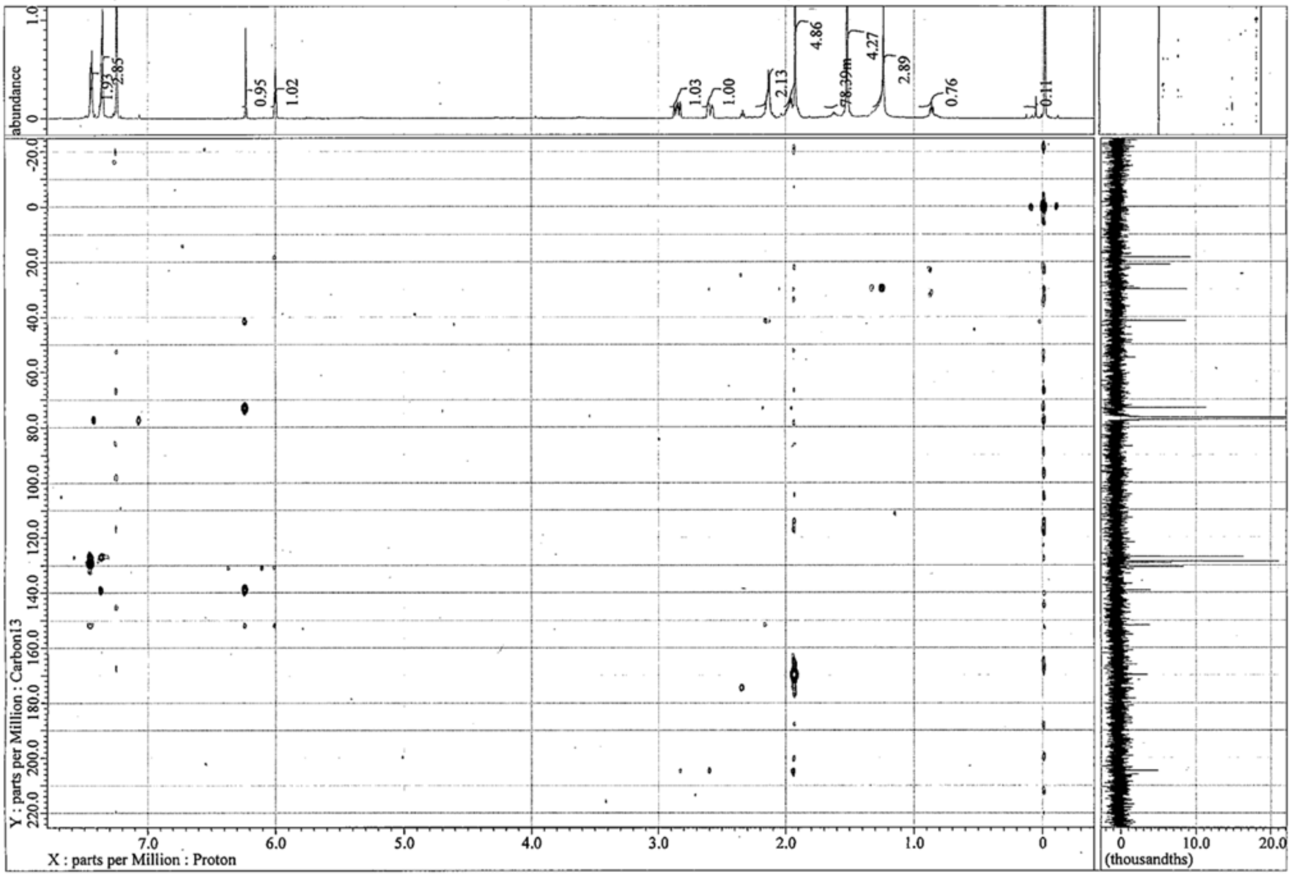

Supporting Figure 22-5 HMBC spectrum of (–)-**S8** (600 MHz, $CDCl_3$)

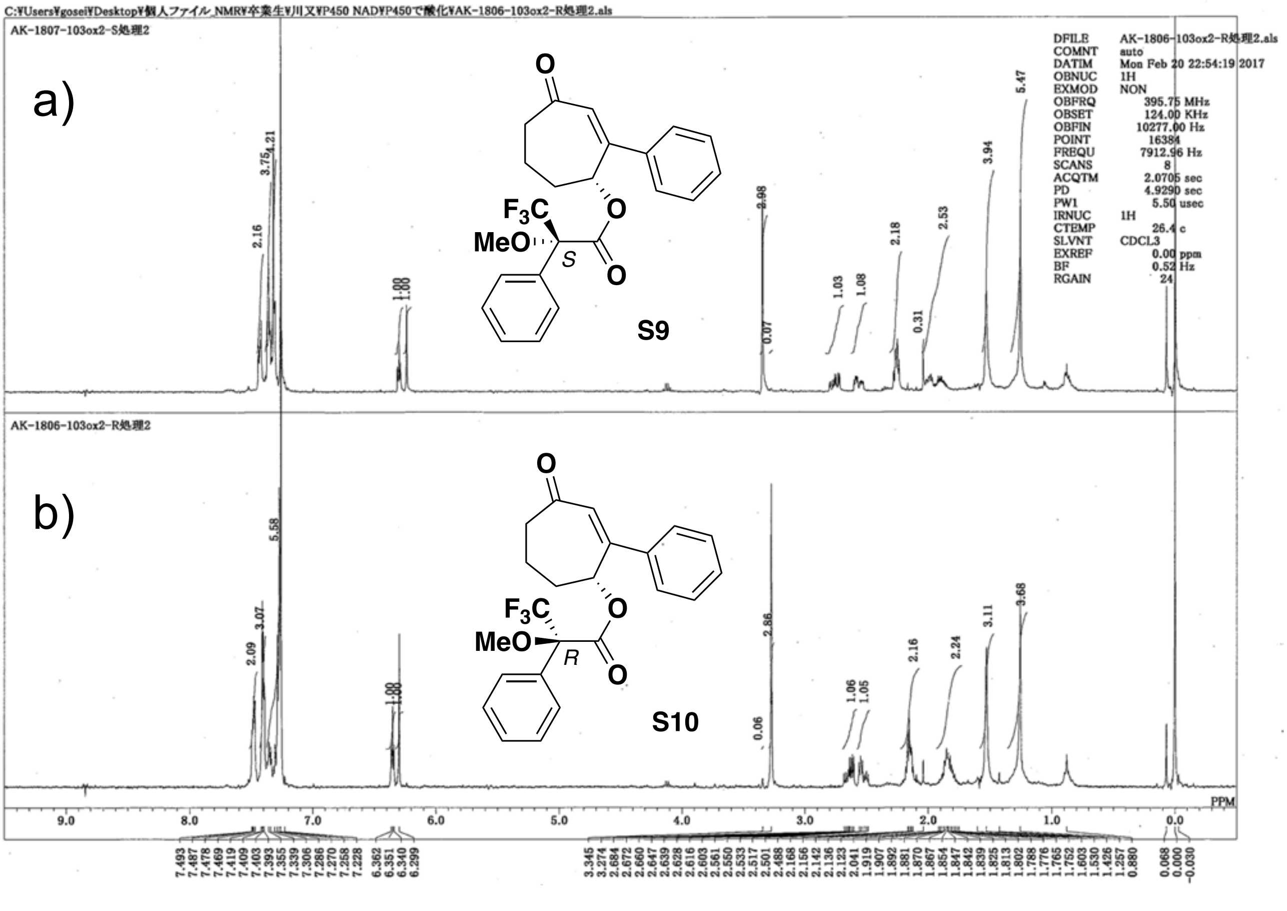

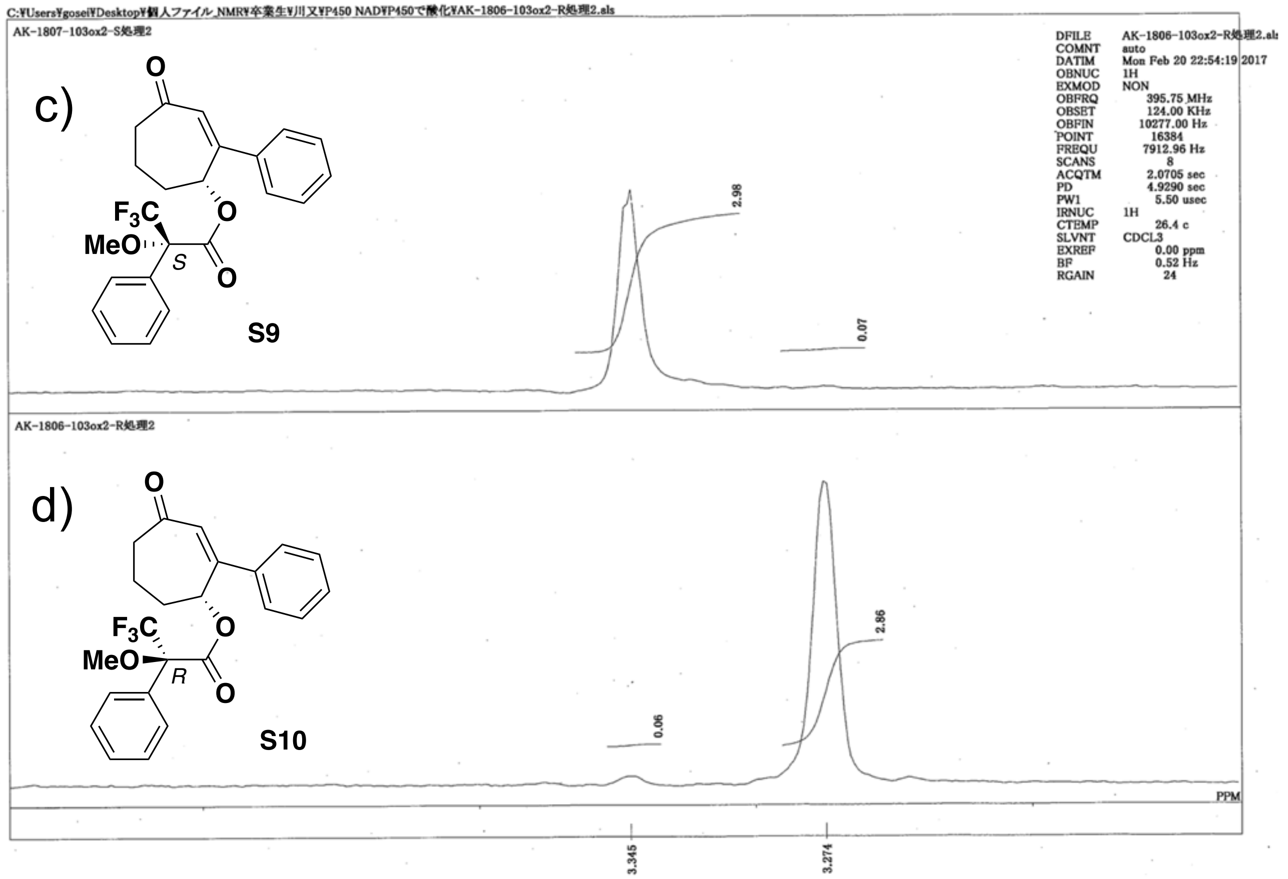

Supporting Figure 23-1 ^{1}H-NMR spectra of **S9** (a, c) and **S10** (b, d) (400 MHz, $CDCl_3$). (a, b) Whole spectrum. (c, d) Expansion of signals at 3.3 ppm.

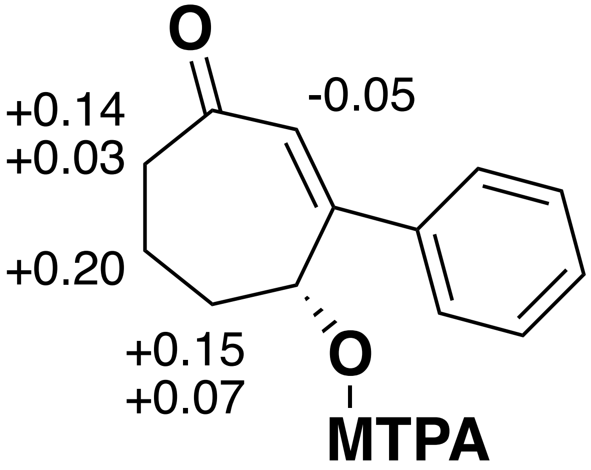

$$\Delta\delta = \delta_S - \delta_R$$

Supporting Figure 23-2 Mosher ester analysis of **#103ox1**.

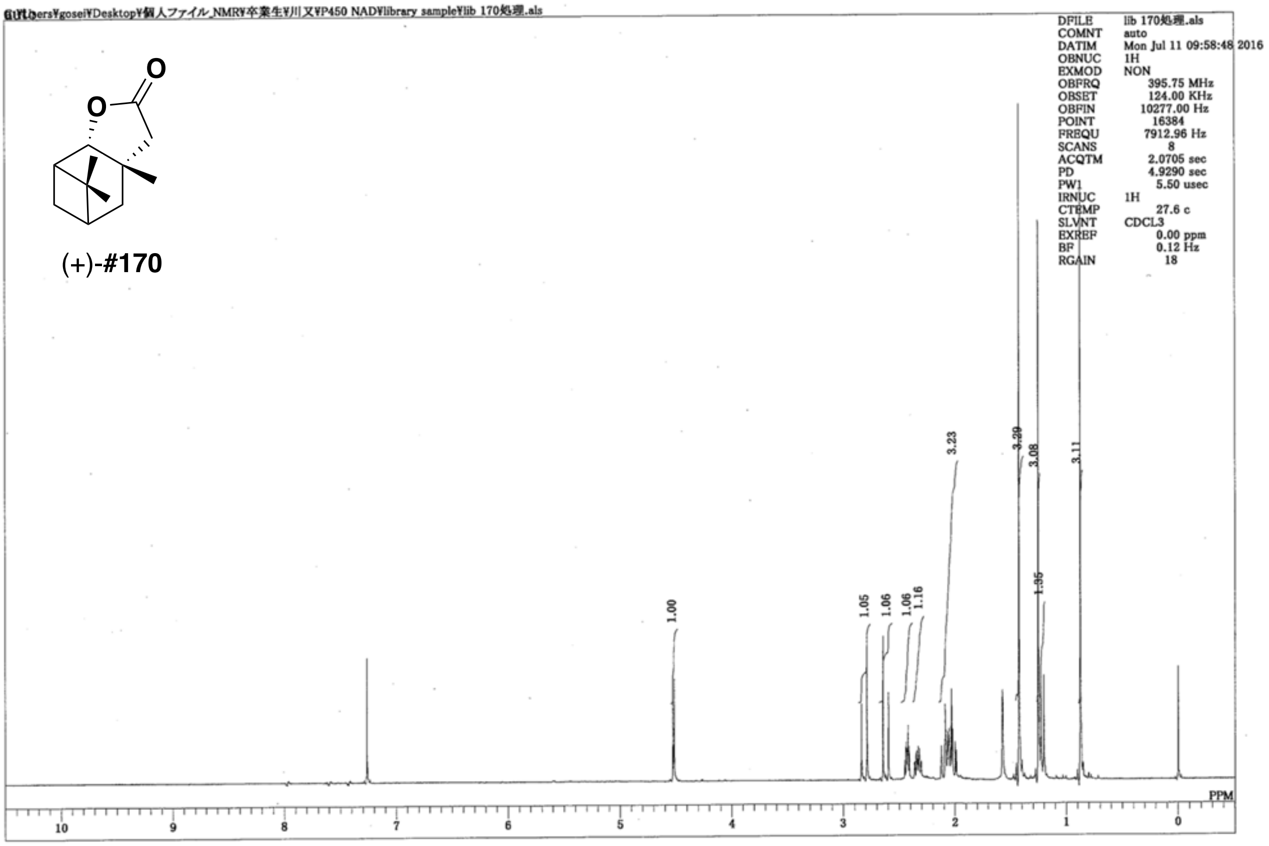

Supporting Figure 24-1 ^{1}H-NMR spectrum of **#170** (400 MHz, $CDCl_3$)

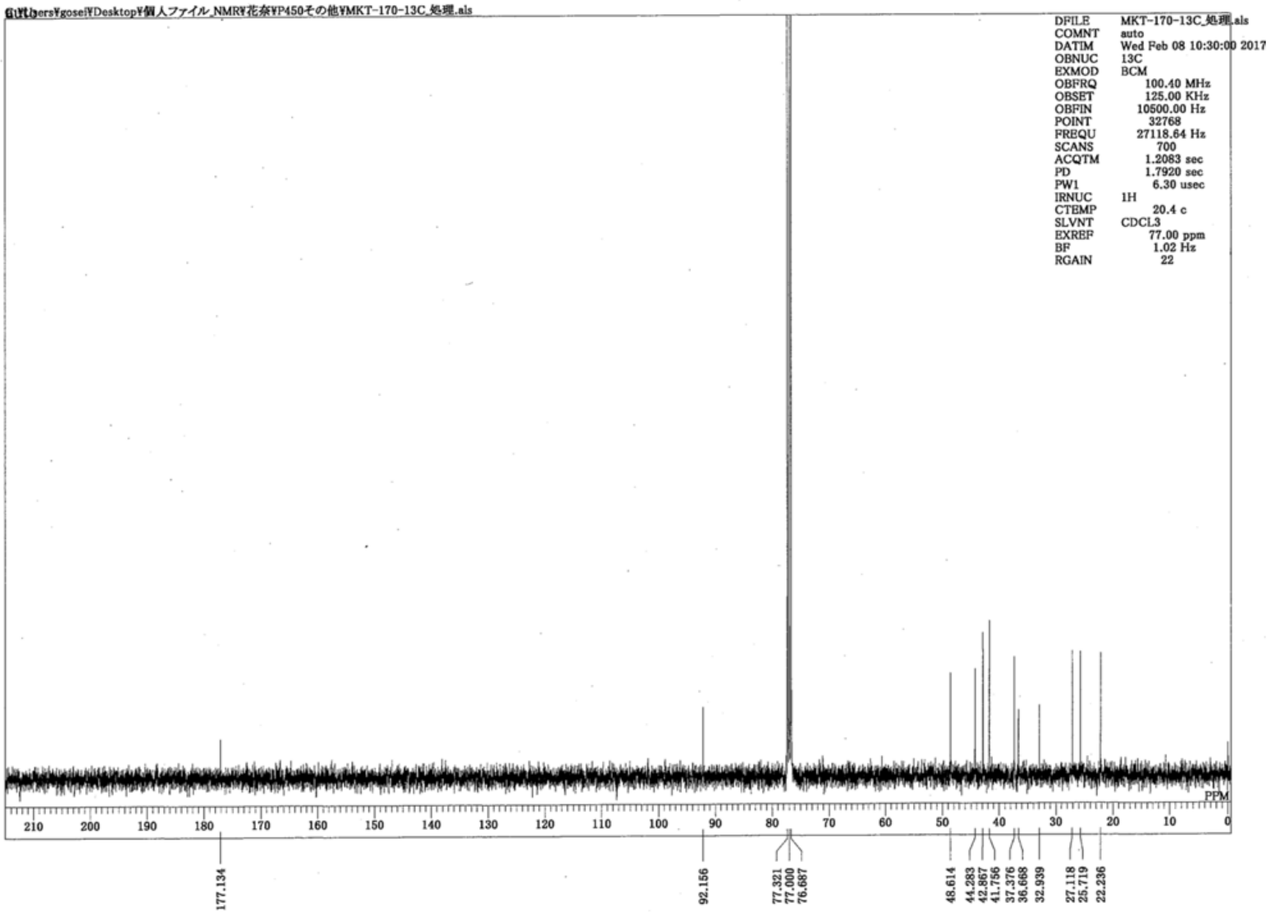

Supporting Figure 24-2 ^{13}C-NMR spectra of **#170** (100 MHz, $CDCl_3$)

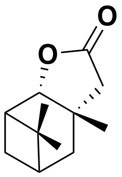

(+)-**#170**

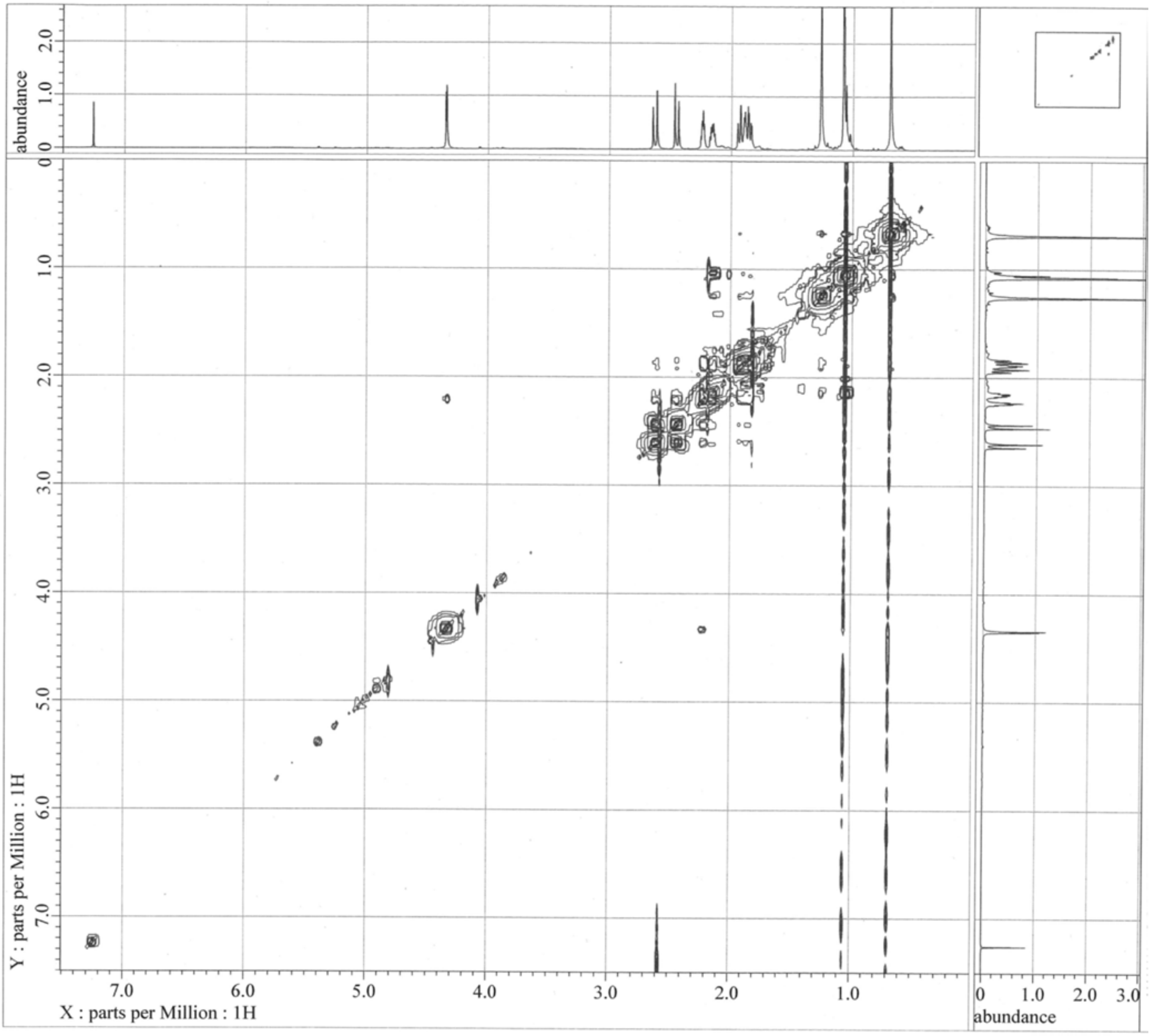

Supporting Figure 24-3 H-H COSY spectrum of **#170** (600 MHz, $CDCl_3$)

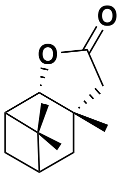

(+)-**#170**

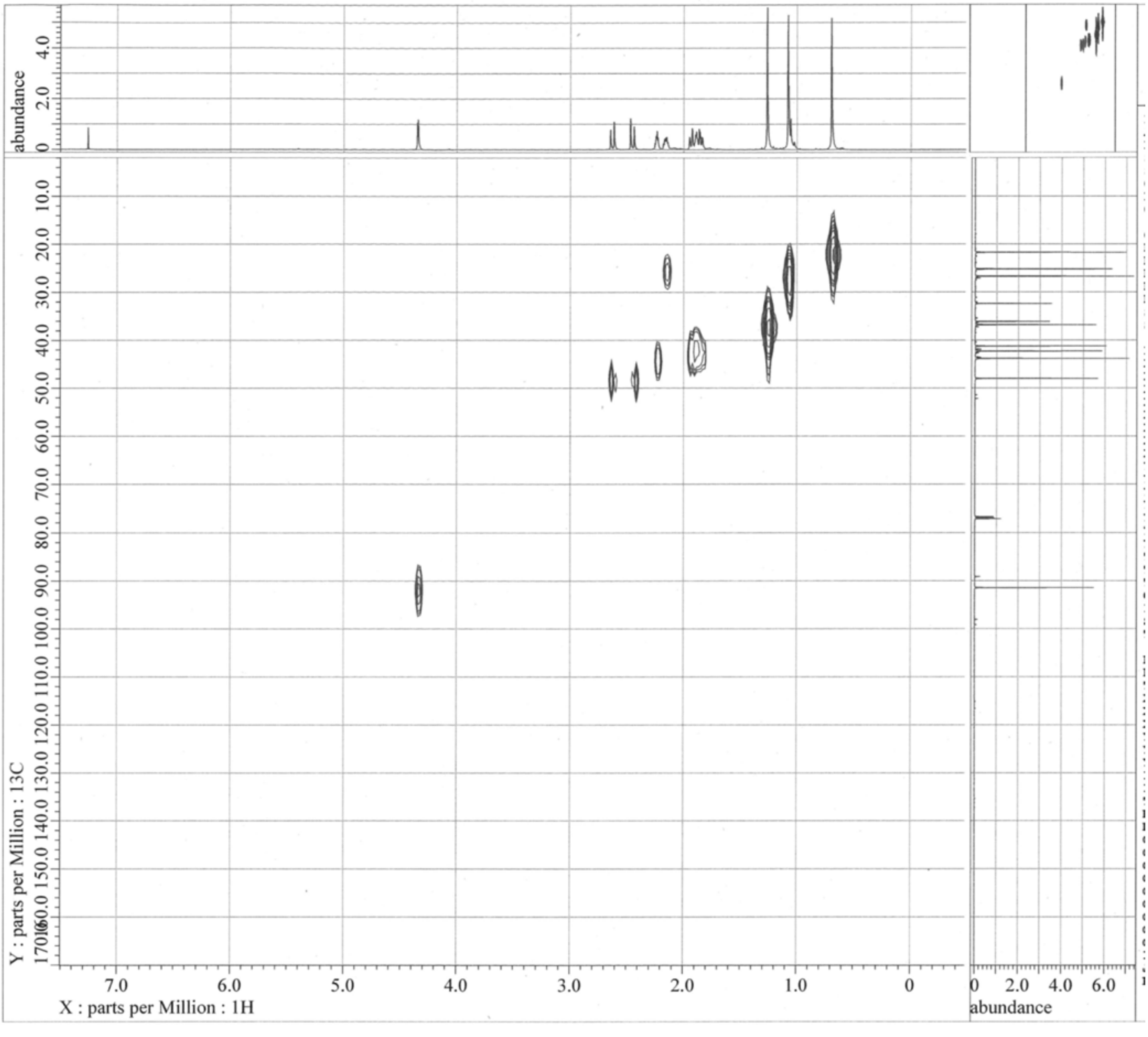

Supporting Figure 24-4 HMQC spectrum of **#170** (600 MHz, $CDCl_3$)

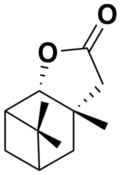

(+)-**#170**

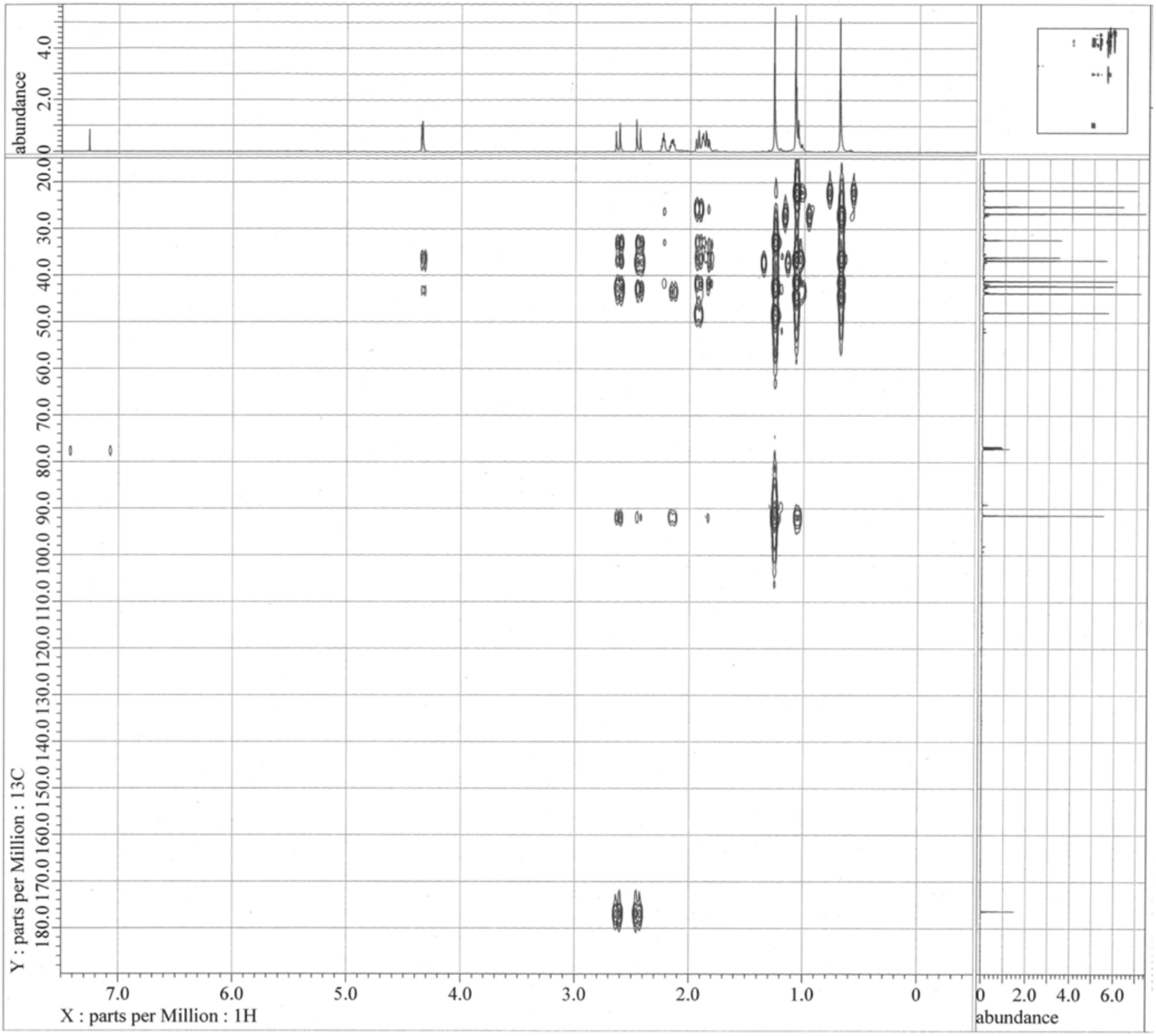

Supporting Figure 24-5 HMBC spectrum of **#170** (600 MHz, $CDCl_3$)

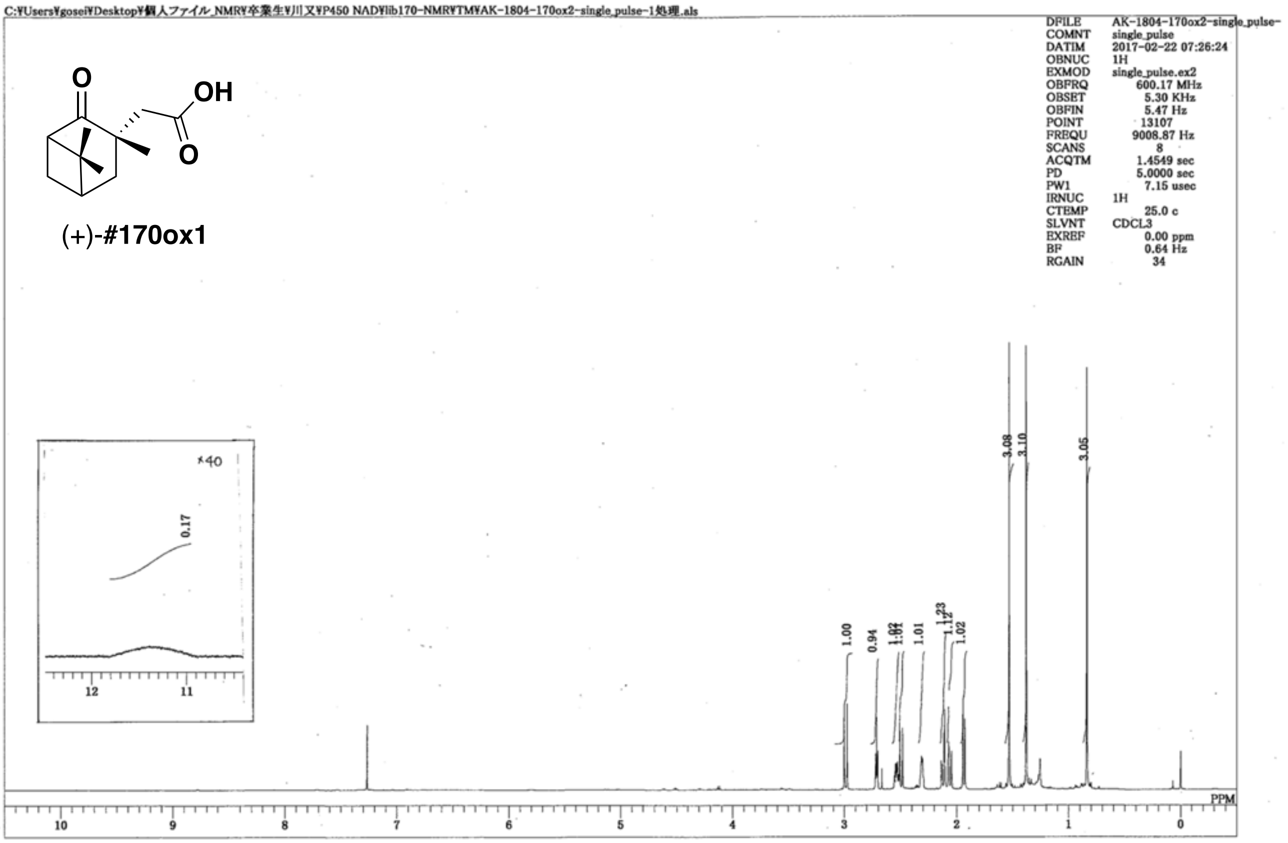

Supporting Figure 25-1 ^{1}H-NMR spectrum of **#170ox1** (400 MHz, $CDCl_3$)

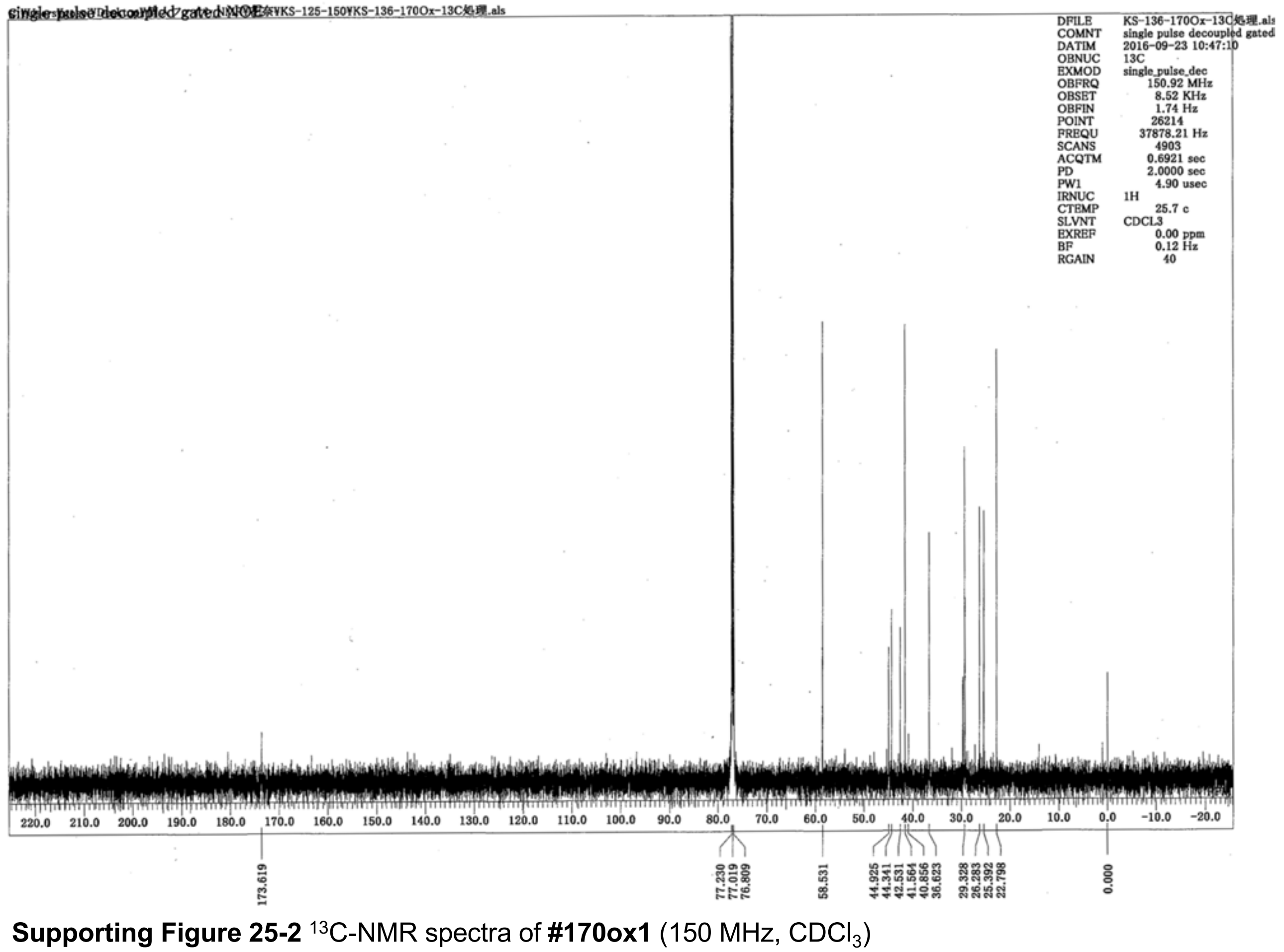

Supporting Figure 25-2 ^{13}C-NMR spectra of **#170ox1** (150 MHz, $CDCl_3$)

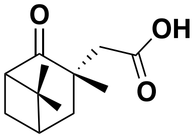

(+)-**#170ox1**

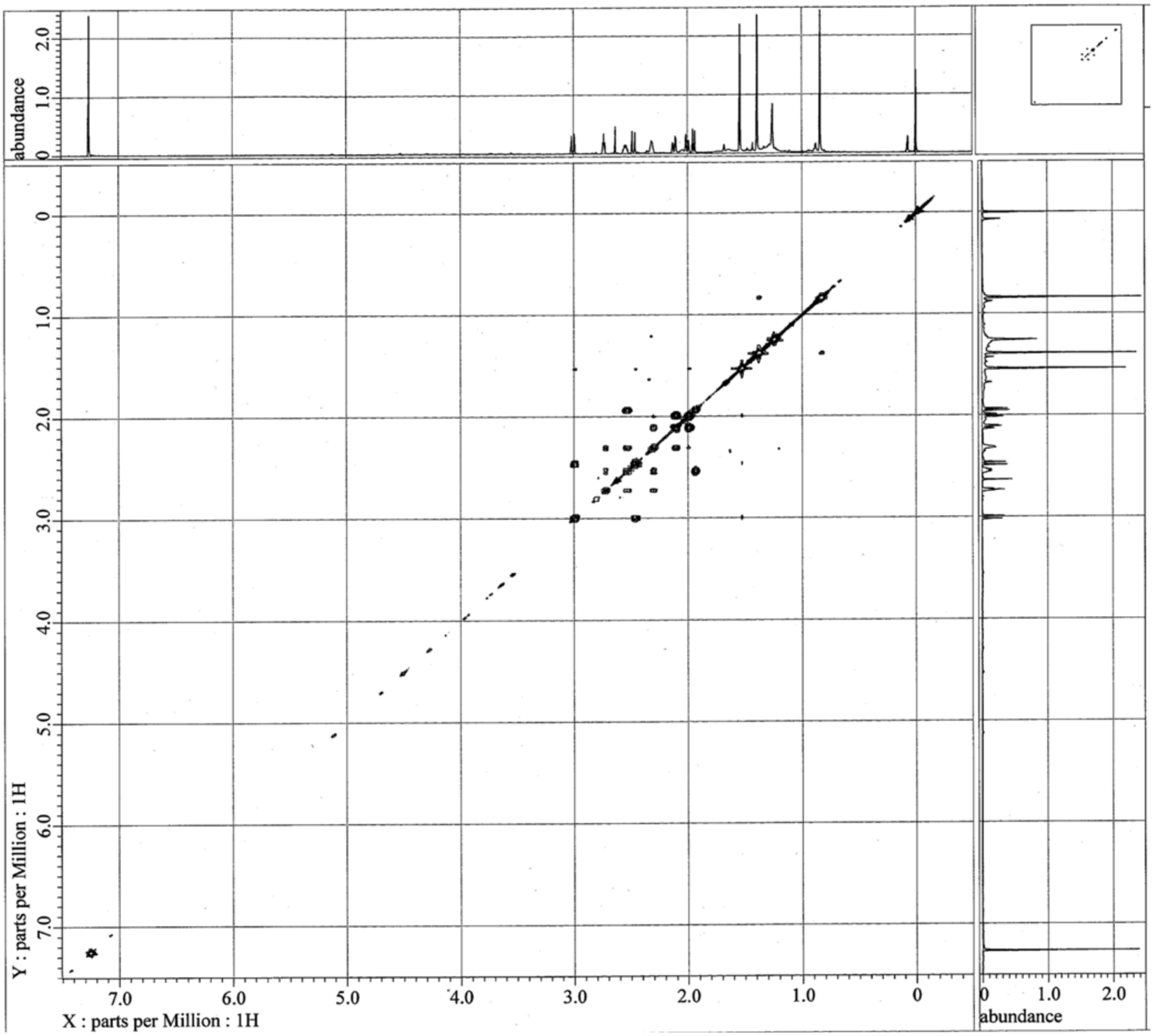

Supporting Figure 25-3 H-H COSY spectrum of **#170ox1** (600 MHz, $CDCl_3$)

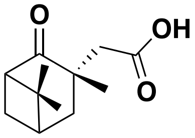

(+)-**#170ox1**

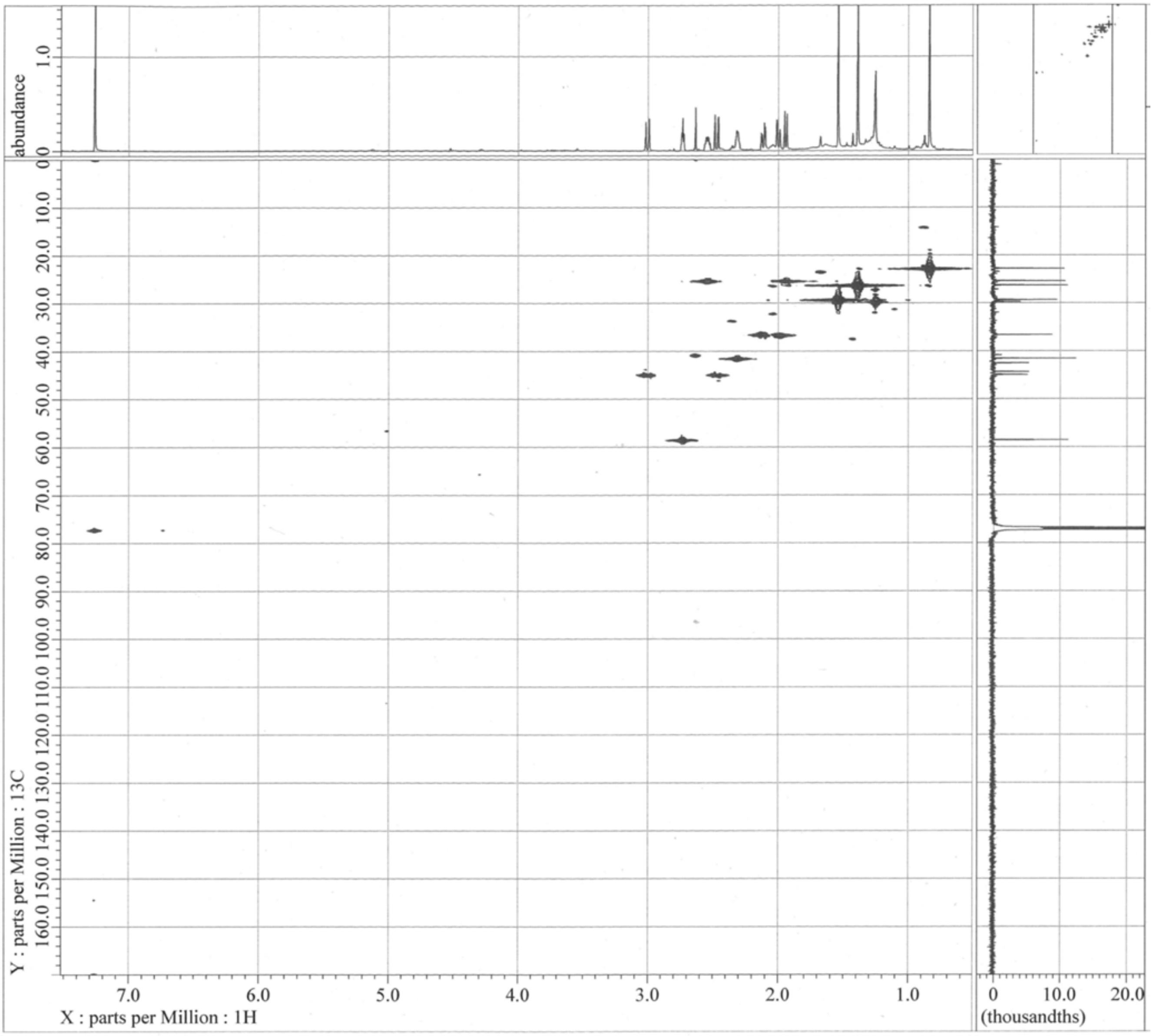

Supporting Figure 25-4 HMQC spectrum of **#170ox1** (600 MHz, $CDCl_3$)

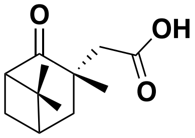

(+)-**#170ox1**

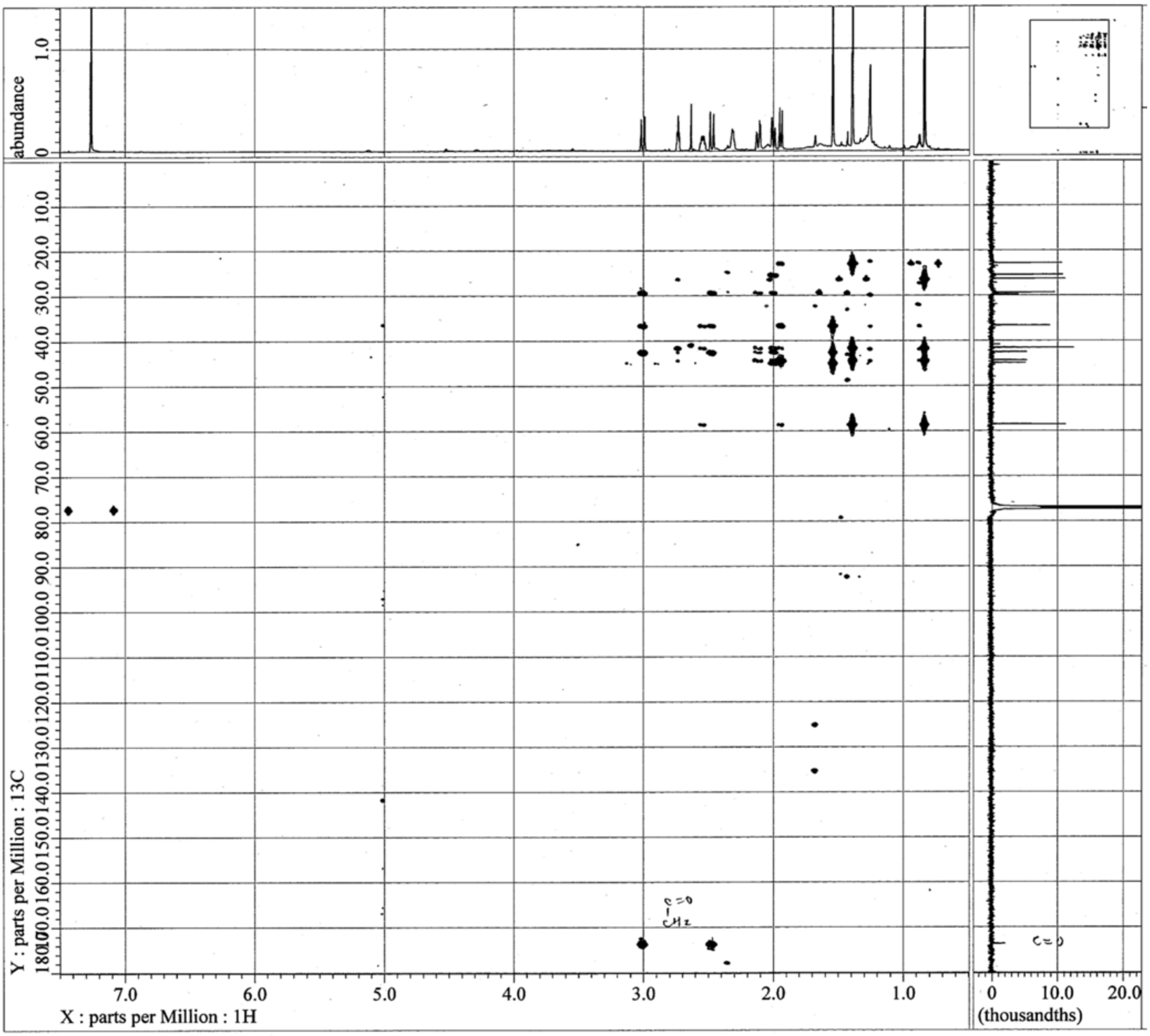

Supporting Figure 25-5 HMBC spectrum of **#170ox1** (600 MHz, $CDCl_3$)

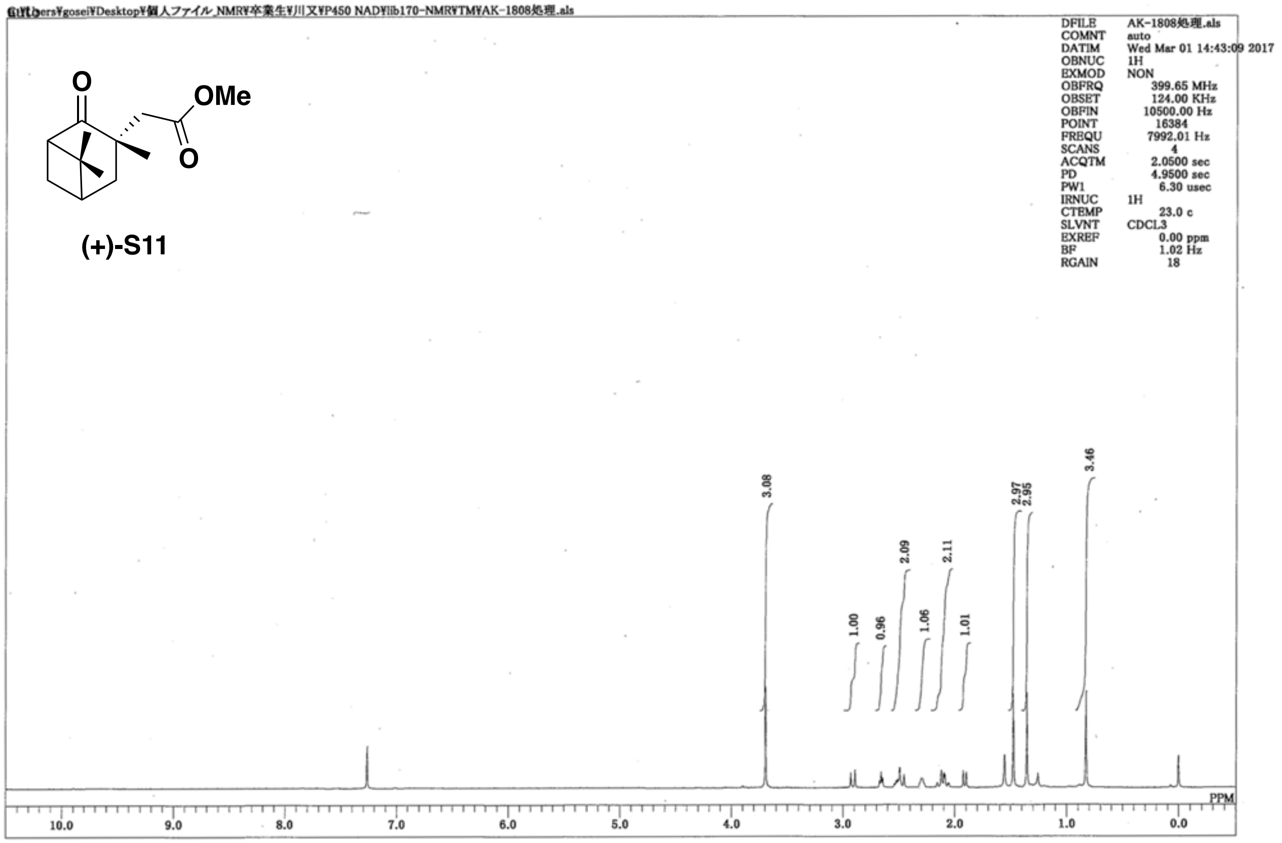

Supporting Figure 26-1 ^{1}H-NMR spectrum of (+)-**S11** (400 MHz, $CDCl_3$)

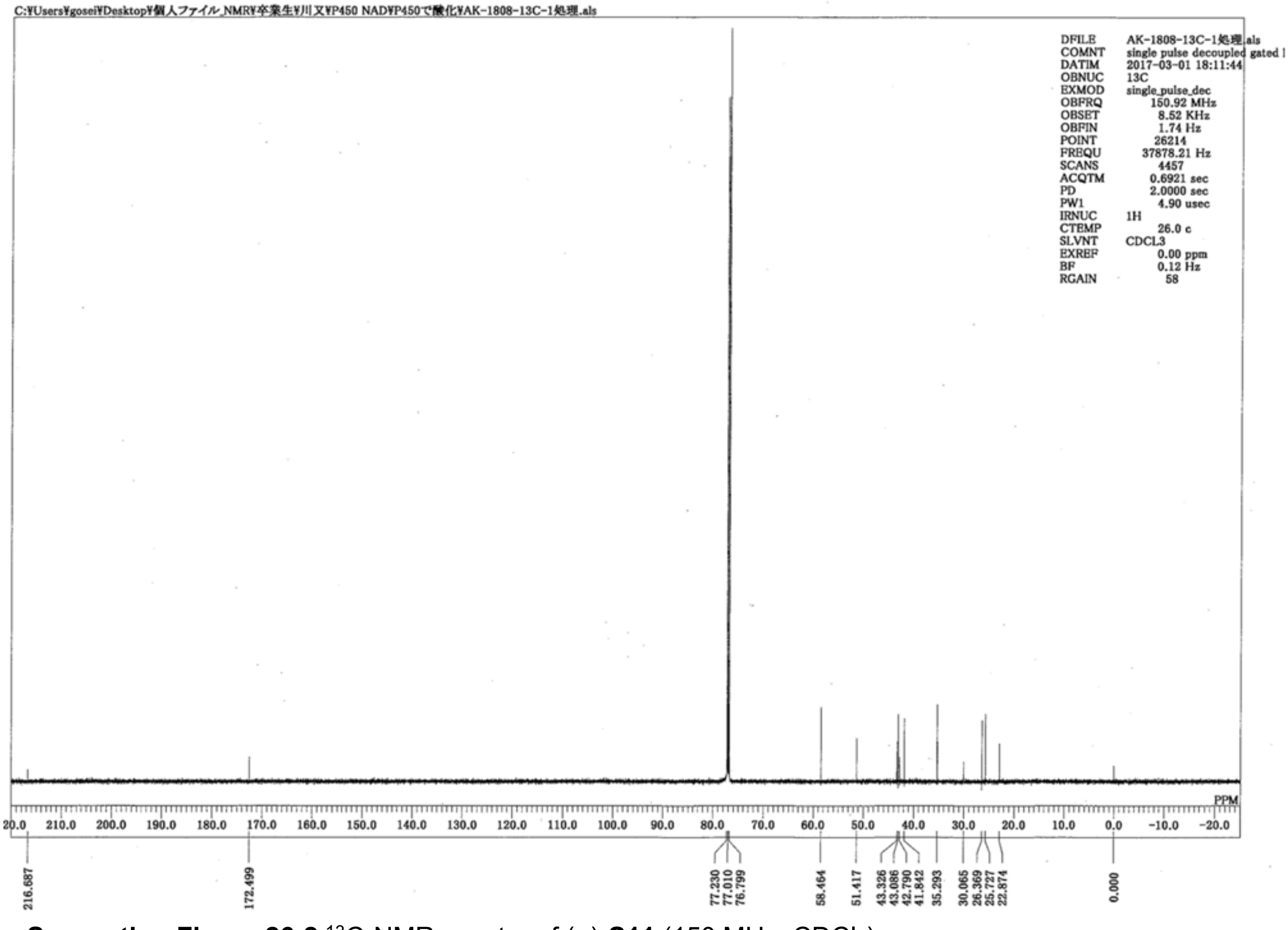

Supporting Figure 26-2 ^{13}C-NMR spectra of (+)-**S11** (150 MHz, $CDCl_3$)

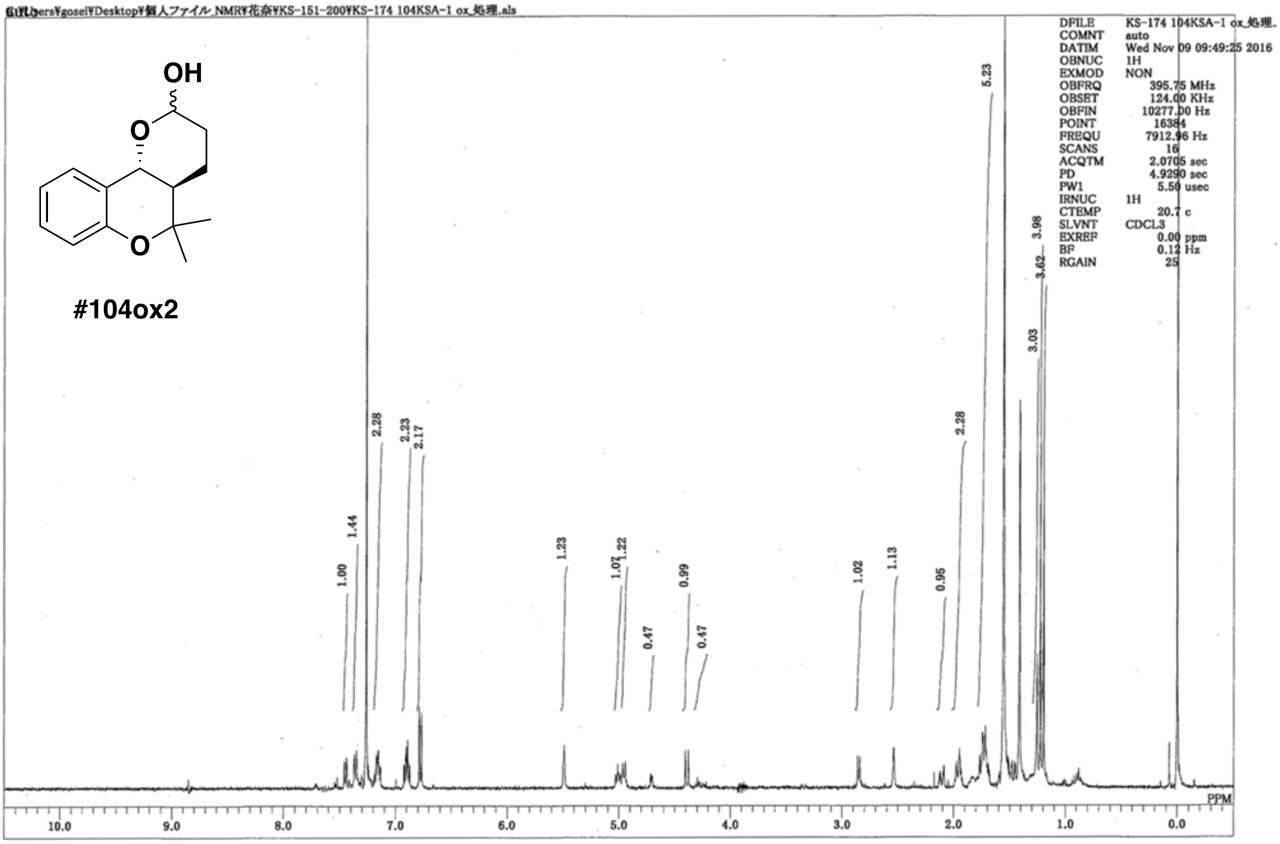

Supporting Figure 27-1 ^{1}H-NMR spectrum of **#104ox2** (400 MHz, $CDCl_3$)

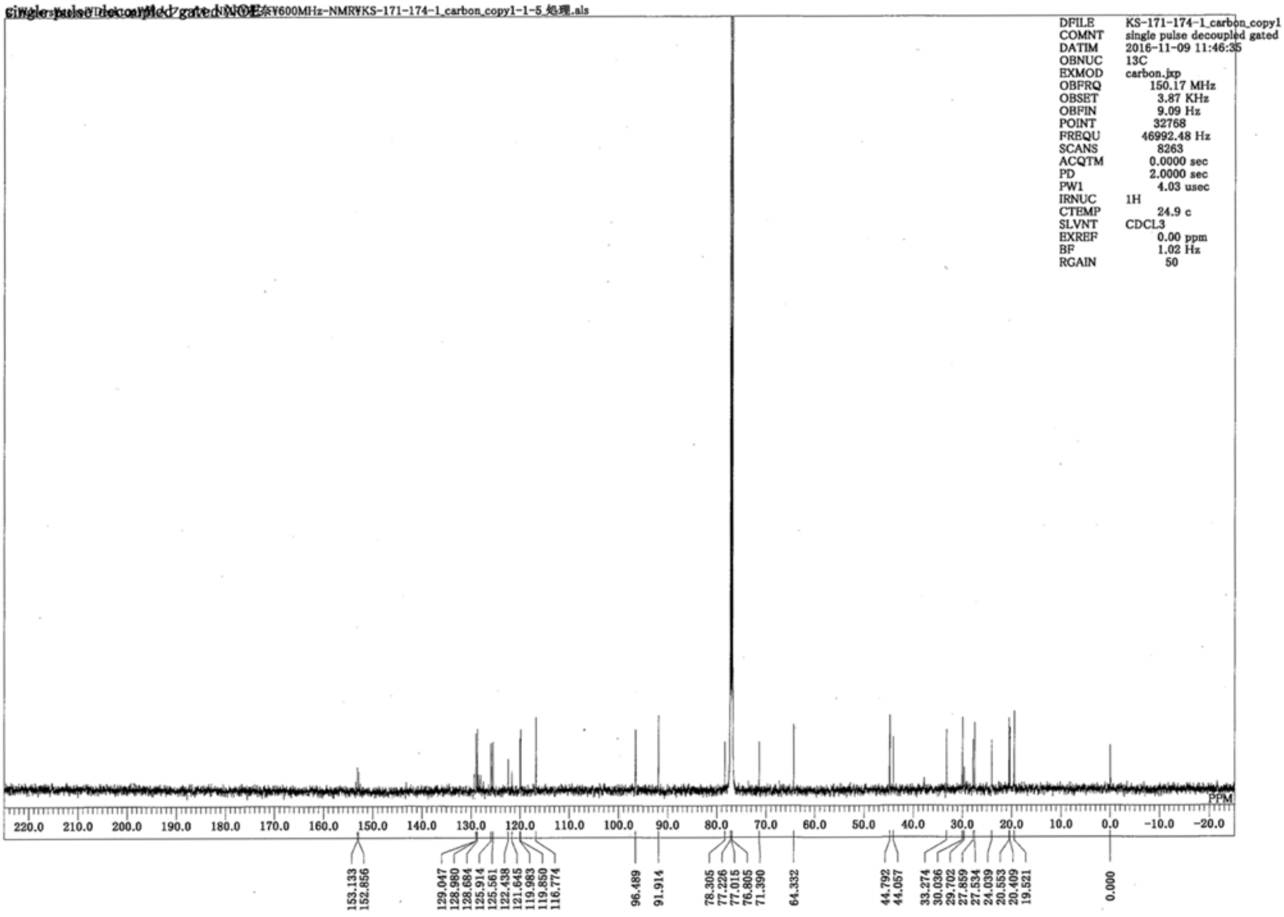

Supporting Figure 27-2 ^{13}C-NMR spectra of **#104ox2** (150 MHz, $CDCl_3$)

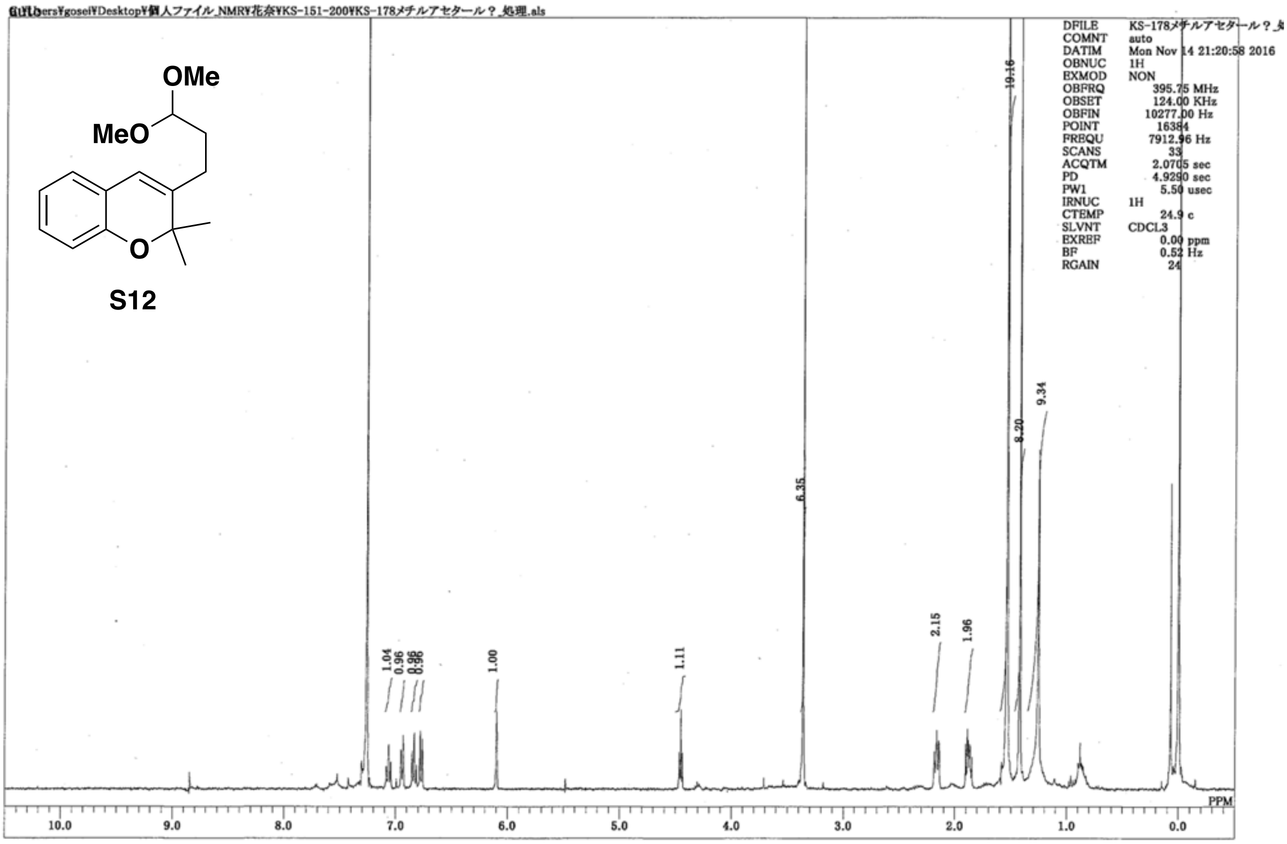

Supporting Figure 28-1 ^{1}H-NMR spectrum of **S12** (400 MHz, $CDCl_3$)

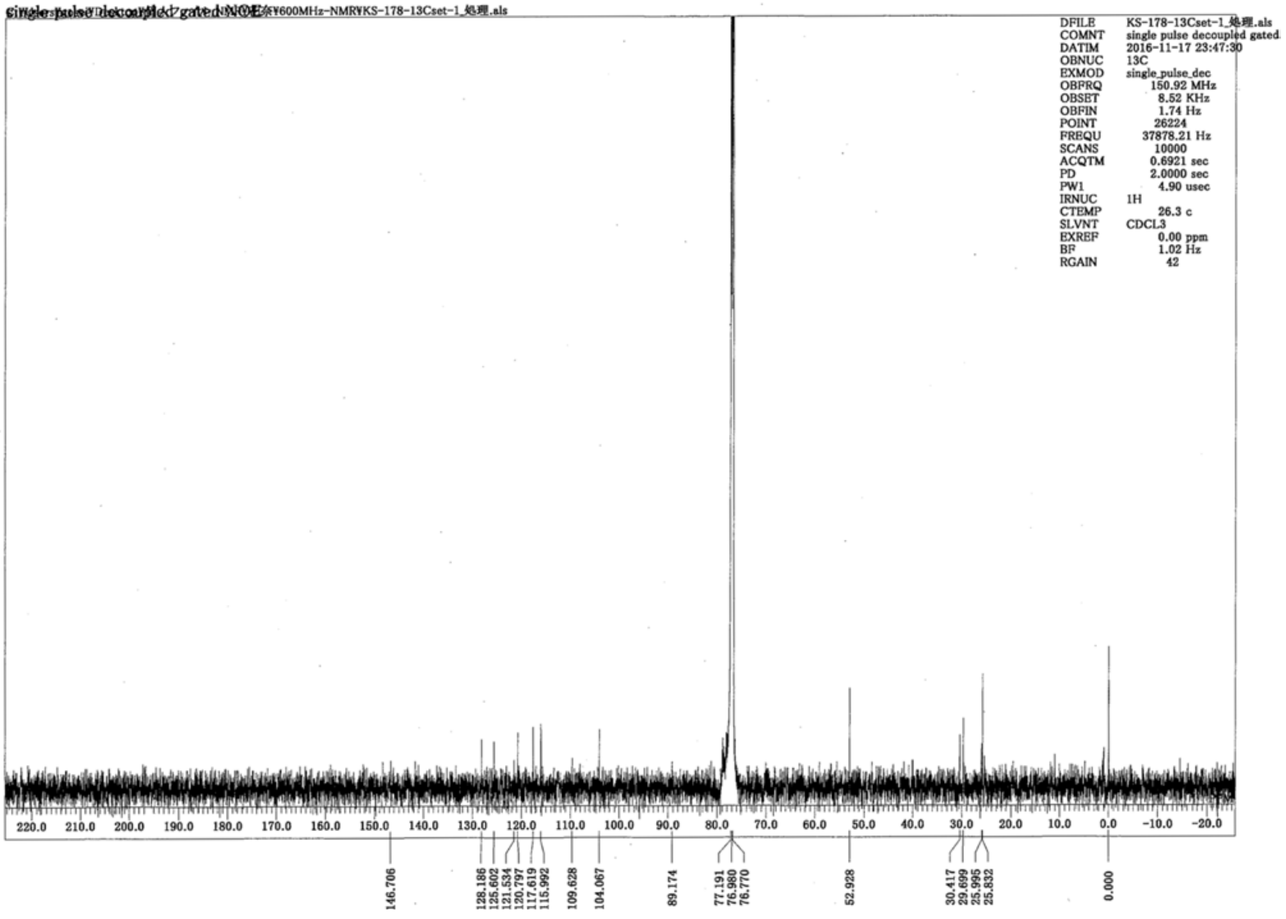

Supporting Figure 28-2 ^{13}C-NMR spectrum of **S12** (150 MHz, $CDCl_3$)

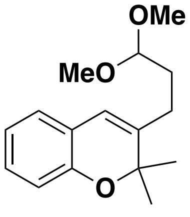

S12

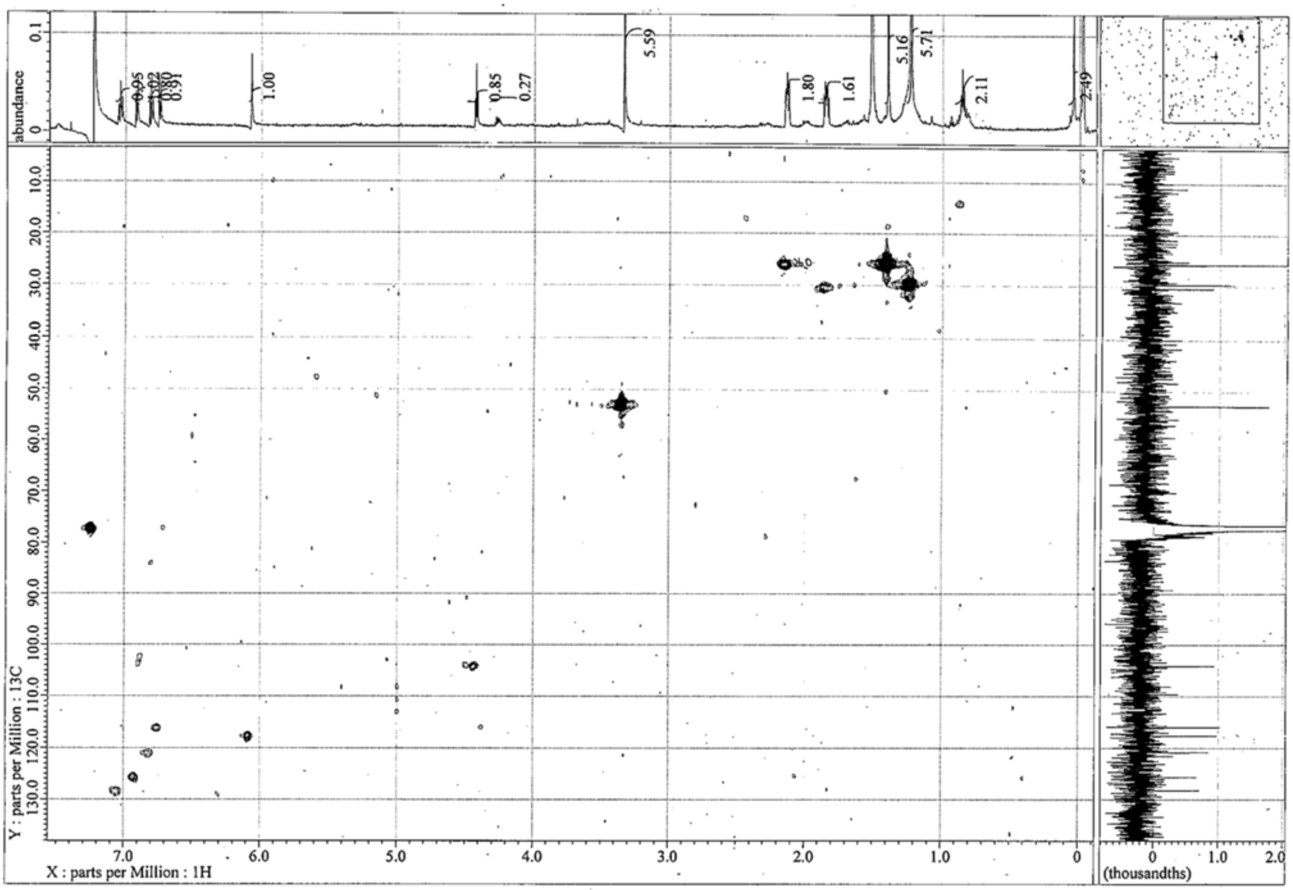

Supporting Figure 28-3 HMQC spectrum of **S12** (600 MHz, $CDCl_3$)

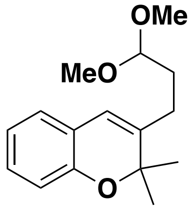

S12

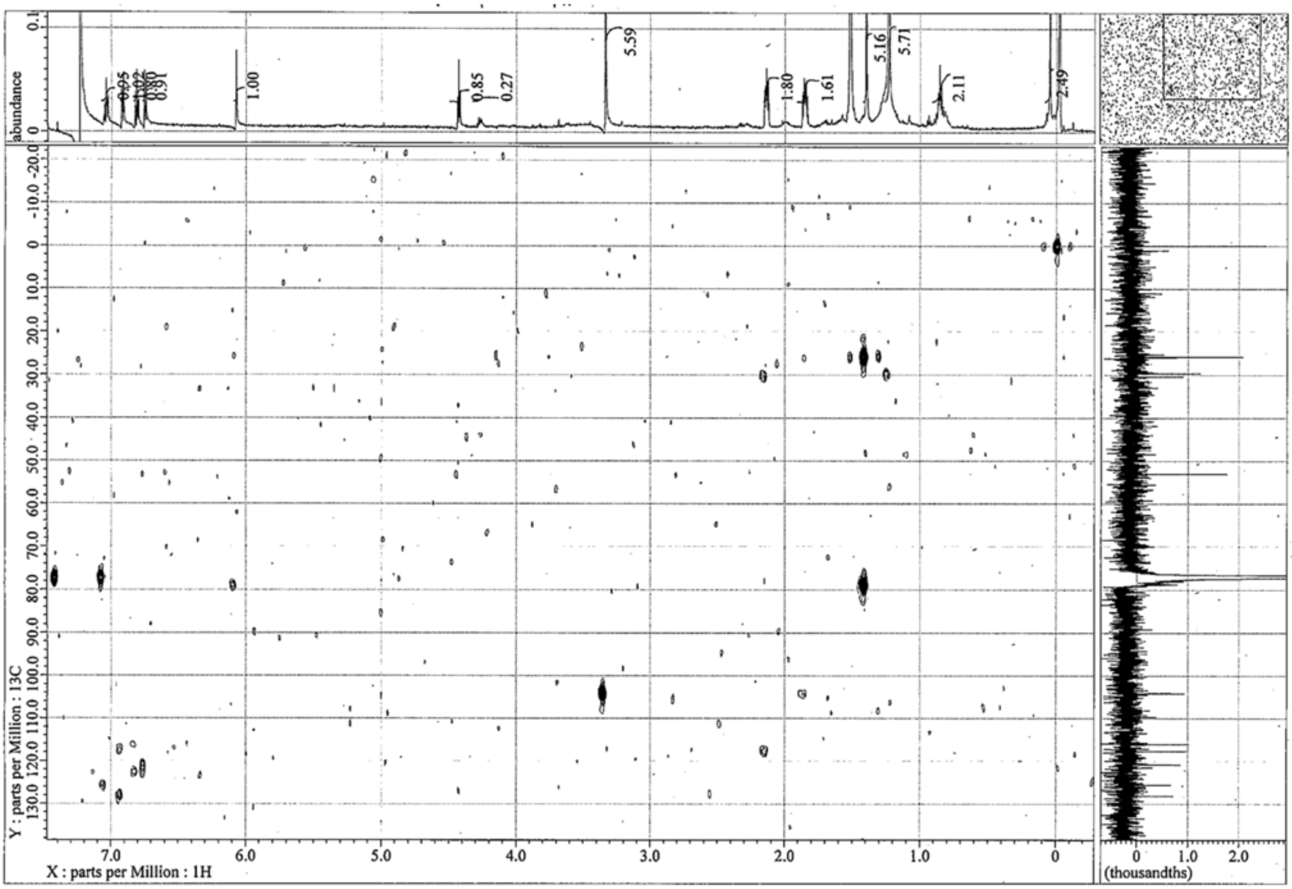

Supporting Figure 28-4 HMBC spectrum of **S12** (600 MHz, $CDCl_3$)

a)

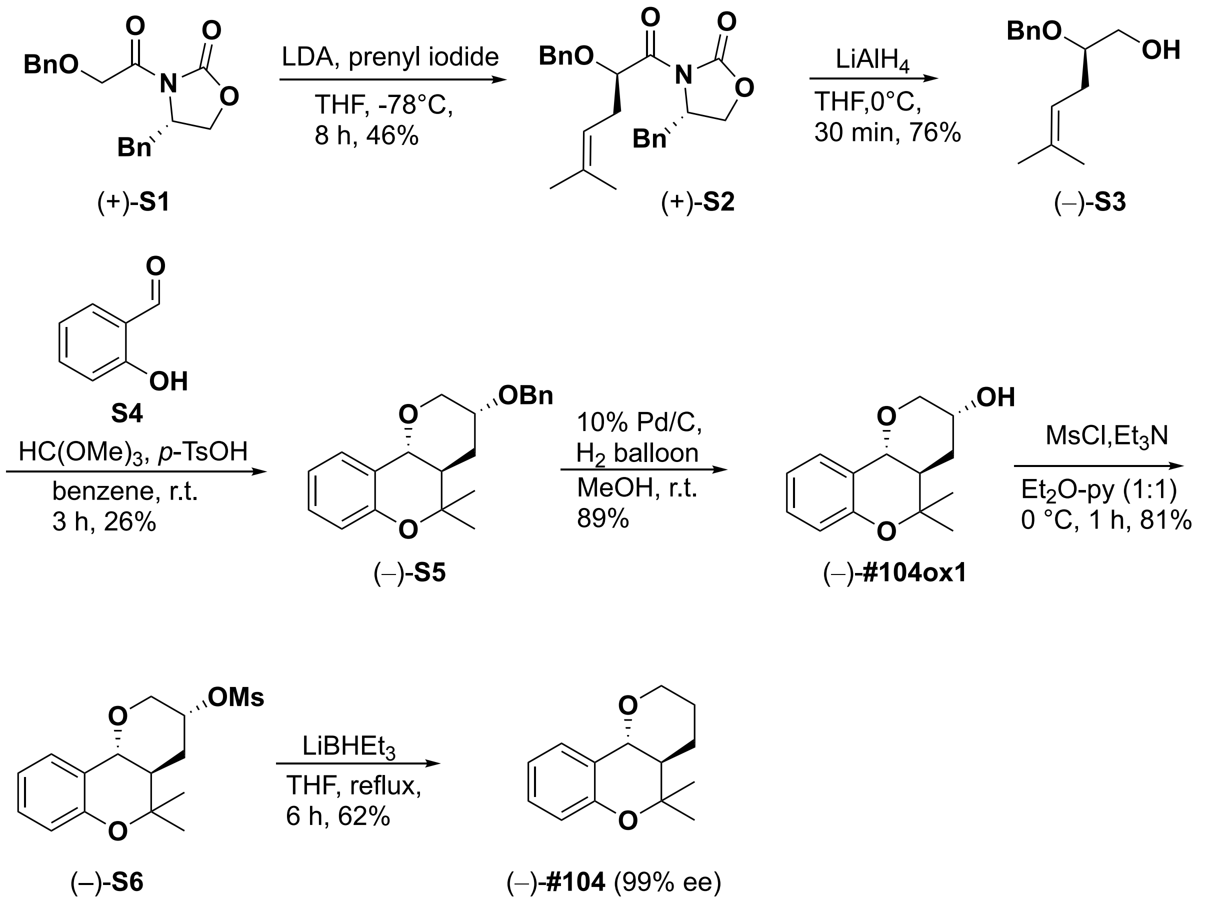

b)

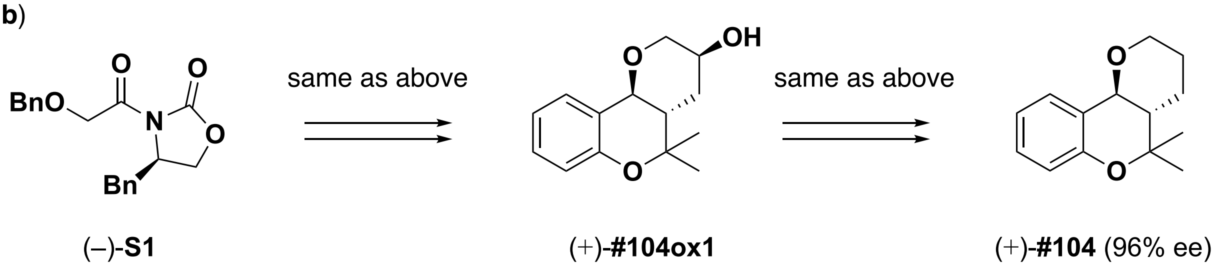

Supporting Scheme 1 Synthesis of optically active **#104** and **#104ox1**

a

Compd #	FI (Av.)	
382	62693	×
369	61532	×
808	60132	×
835	59765	×
1012	59191	×
291	57398	×
816	56688	×
855	54451	×
475	50028	×
460	49305	×
553	48838	×
561	47414	×
958	47131	×
571	46995	×
178	45003	×
324	44415	×
159	44283	×
1022	43327	×
532	43279	×
161	42645	×
422	41284	×
505	41262	×
1045	40782	×
370	40417	
515	39954	×
142	38189	×
955	37874	×
830	37440	×
1027	36933	×
179	35943	
807	35876	×
372	34485	×
260	34360	×
930	34223	×
871	34148	×
54	34132	×
479	34033	×
188	33185	×
968	33095	×
802	32754	×
285	32182	×
211	31949	×
261	31905	×
225	31890	×
53	31212	×
1052	30975	×
502	30430	×
302	29694	
30	29633	×
509	29077	×
32	28904	×
892	28789	×
34	28769	×
876	28686	× mean+2SD
525	27839	×
546	27338	×
294	27000	×

Compd #	FI (Av.)	
289	26706	
562	26613	×
52	26410	×
339	26236	
318	26216	
572	26014	×
393	25941	×
484	25900	×
581	25654	×
558	25552	×
47	25501	×
396	25362	
241	25207	
445	24520	×
860	24035	
204	23228	
478	22946	
354	21656	
573	21636	
466	21583	
567	21347	
164	21138	
745	21071	
895	20778	
769	20771	
298	20729	
259	20691	×
101	20460	
533	20371	
295	20244	
379	20212	
65	20205	×
16	20057	×
1007	19881	×
432	19879	
160	19831	
104	19709	
480	19666	
884	19531	
842	19428	
401	19383	
154	19258	×
486	19212	
1029	18814	
235	18799	
923	18770	×
335	18752	Q3+1.5IQR

b

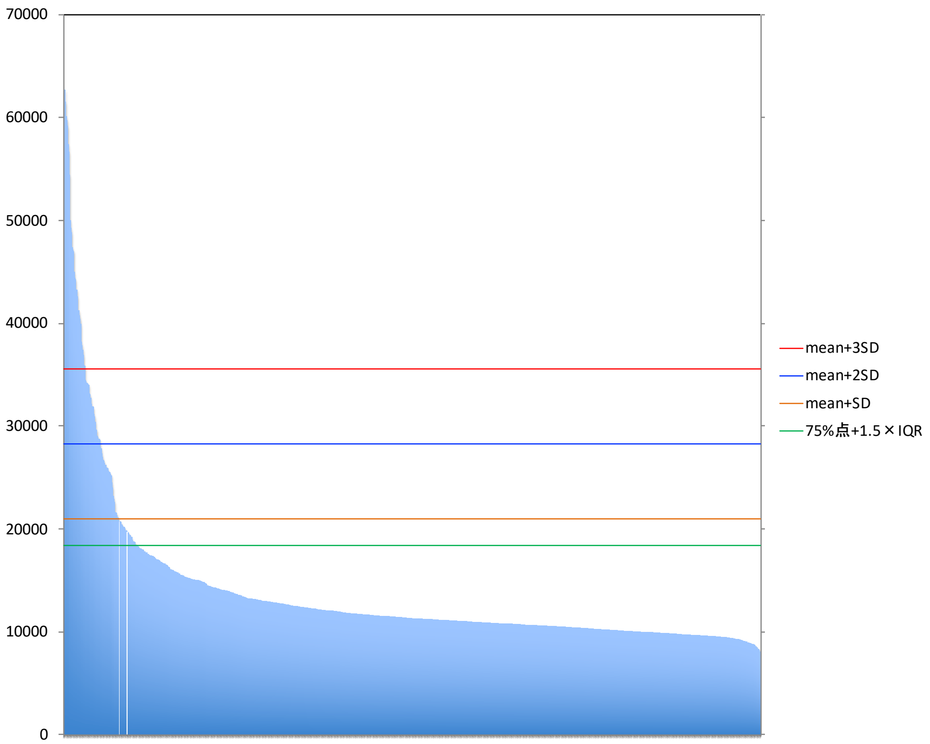

mean	mean+2SD
13724	28296
SD	mean+SD
7286	21010
Q3 (75%)	Q3+1.5IQR
13595	18425
Q2 (50%)	
11324	
Q1 (25%)	
10374	
IQR	
3221	

Supporting Table 1-1 1st screening results of P450 cam substrates (Mean of 2 replicates). (a) Compounds that promote the generation of NADP$^+$ are listed in descenting order of the fluorescent signal intensity of NADP$^+$. Compounds having value above Q3 +1.5 IQR are shown. Compounds marked in yellow are hit compounds. X-marks (×) indicate positives for all assessor P450s. (b) Bar graph representation of all data in descending order of the fluorescent signal intensity.

a

Compd #	FI (Av.)	
291	61217	×
382	61040	×
369	58918	×
553	58899	×
955	57800	×
855	57235	×
968	56508	×
571	56014	×
1045	55413	×
**480**	**54491**	
479	54434	×
178	53706	×
808	53594	×
958	52167	×
54	51915	×
558	50824	×
561	49650	×
1027	49600	×
802	48358	×
581	47714	×
285	47430	×
509	47246	×
484	46870	×
930	45671	×
572	45574	×
32	45502	×
816	45364	×
161	44278	×
159	44009	×
259	43167	×
188	43034	×
261	42468	×
532	42245	×
16	42087	×
53	41299	×
211	40831	×
505	40807	×
225	40513	×
34	40510	×
30	40483	×
830	40199	×
**573**	**40141**	
324	39731	×
**370**	**39724**	
142	39415	×
562	39307	×
**3**	**39182**	
515	39092	×
525	38939	×
393	38384	×
422	38368	×
1052	37749	×
**24**	**37583**	
**473**	**37006**	
460	36440	×
**676**	**36314**	
892	36273	×
154	36060	×
294	35409	×
260	35237	×
**70**	**35154**	
**266**	**35134**	
1022	34987	×
546	34885	×
47	34699	×

Compd #	FI (Av.)		
**860**	**34635**		
**71**	**34515**		
372	34209	×	mean+2SD
1012	34058	×	
**27**	**34037**		
**204**	**33637**		
807	33627	×	
445	33594	×	
**72**	**33463**		
**258**	**33201**		
**73**	**32580**		
**267**	**32401**		
871	32401	×	
**181**	**32221**		
**18**	**32159**		
**35**	**32139**		
**287**	**32002**		
**164**	**31869**		
65	31833	×	
**182**	**31806**		
**235**	**31743**		
**215**	**31695**		
**19**	**31509**		
**101**	**31453**		
835	31447	×	
**242**	**31295**		
**69**	**30567**		
**896**	**30493**		
**549**	**29928**		
**302**	**29923**		
**481**	**29888**		
**432**	**29524**		
**898**	**28905**		
**283**	**28721**		
**210**	**28641**		
475	28627	×	
**548**	**28404**		
**565**	**28343**		
**552**	**28336**		
**928**	**28326**		
**796**	**28172**		
**223**	**28111**		
**585**	**28083**		
**529**	**28041**		
**919**	**27831**		
**537**	**27484**		
**950**	**27438**		
52	27343	×	
**290**	**27322**		
**872**	**27157**		
**284**	**27129**		
**121**	**27090**		
**533**	**27066**		
**221**	**26900**		
**254**	**26873**		
**295**	**26730**		
**478**	**26616**		Q3+1.5IQR

b

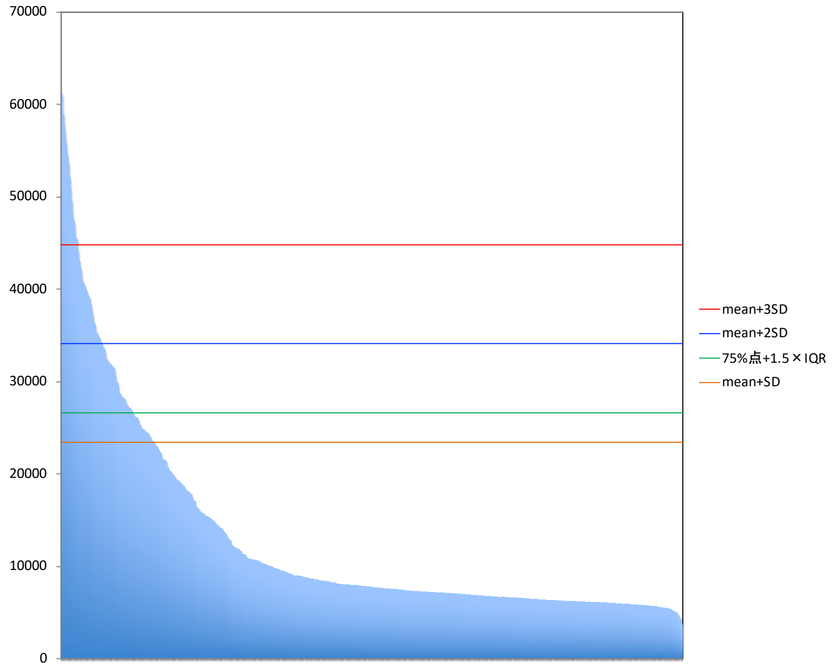

mean	mean+2SD
12734	34118
SD	mean+SD
10692	23426
Q3 (75%)	Q3+1.5IQR
14548	26612
Q2 (50%)	
7740	
Q1 (25%)	
6506	
IQR	
8043	

Supporting Table 1-2 1st screening results of P450 RevI substrates (Mean of 2 replicates). (a) Compounds that promote the generation of $NADP^+$ are listed in descenting order of the fluorescent signal intensity of $NADP^+$. Compounds having value above Q3 +1.5 IQR are shown. Compounds marked in yellow are hit compounds. X-marks (×) indicate positives for all assessor P450s. (b) Bar graph representation of all data in descending order of the fluorescent signal intensity.

a

Compd #	FI (Av.)	
553	43695	×
1012	40709.5	×
1045	40141.5	×
855	37868.5	×
1022	35893.5	×
808	35566	×
382	33938.5	×
369	30113.5	×
532	29914	×
1052	28397.5	×
771	27785	
1027	27413	×
816	25546.5	×
811	25368.5	
448	24275	
324	24031	×
810	23361	
571	23244	×
830	23195	×
34	22656.5	×
505	22440.5	×
509	20433	×
558	20364	×
291	19980	×
871	17962	×
958	17075.5	×
32	16841	×
896	15954	
812	14992.5	
53	14910.5	×
178	14575	×
154	14179	×
895	13503.5	
515	13043	×
495	12157	
475	11868.5	×
30	11789	× mean+2SD
47	11307.5	×
35	11082.5	
561	10881.5	×
460	10537	×
54	10460.5	×
285	10207.5	×
159	9360	×
807	9357.5	×
968	9307	×
261	9046	×
772	8978.5	
950	8951	
876	8097.5	×
892	8097.5	×
479	7819	×
260	7742	×
315	7526.5	
188	7434	×
507	7369.5	
930	7336.5	×
24	7186.5	
955	6889	×
502	6448	×
625	6056	
449	6032.5	
567	5977.5	

Compd #	FI (Av.)	
16	5874.5	×
711	5516.5	
1007	5514	×
44	5490	
835	5487	×
621	5284	
572	5254.5	×
211	5187.5	×
623	5134	
562	5099.5	×
749	5087.5	
161	5022.5	×
225	4952	×
397	4949	×
372	4891.5	×
525	4796.5	×
45	4734	
71	4530.5	
258	4464	
585	4425	
546	4385.5	×
484	4349.5	×
313	4325.5	
422	4301	×
52	4274	×
622	4178.5	
393	4165.5	×
795	4080.5	
142	4033.5	×
1039	4012.5	
187	3983.5	
648	3905.5	
801	3890.5	
802	3863	×
842	3851	
923	3851	×
239	3773	
379	3718	
839	3632.5	
556	3611	
294	3605.5	×
27	3576	
914	3551	
581	3458	×
728	3434	
651	3329	
402	3313.5	
653	3225	
65	3223	×
652	3197	
815	3189	
641	3179.5	
529	3141	
70	3133.5	
29	3104	
259	3067	×
1010	3031.5	
445	3000	×
598	2994.5	Q3+1.5IQR

b

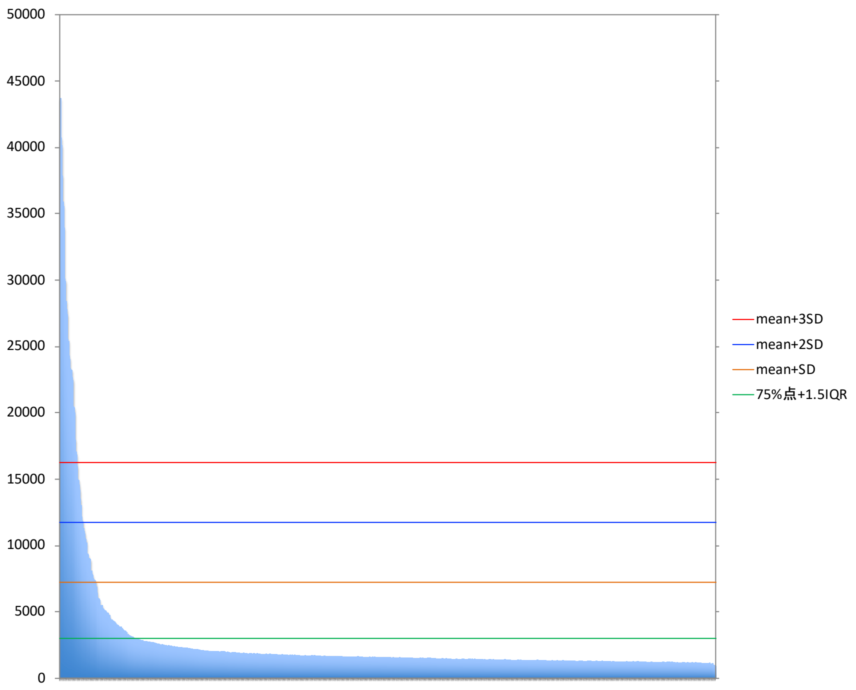

mean	mean+2SD
2702	11731
SD	mean+SD
4514	7217
Q3 (75%)	Q3+1.5IQR
2003	2988
Q2 (50%)	
1587	
Q1 (25%)	
1347	
IQR	
657	

Supporting Table 1-3 1st screening results of P450 BM3(WT) substrates (Mean of 2 replicates). (a) Compounds that promote the generation of $NADP^+$ are listed in descenting order of the fluorescent signal intensity of $NADP^+$. Compounds having value above Q3 +1.5 IQR are shown. Compounds marked in yellow are hit compounds. X-marks (×) indicate positives for all assessor P450s. (b) Bar graph representation of all data in descending order of the fluorescent signal intensity.

a

Compd #	FI (Av.)	
807	35083	×
178	34134	×
294	29402	×
104	28788	
958	28703	×
23	27644	
25	26684	
53	26679	×
1012	26193	×
516	25458	
830	24688	×
211	24643	×
554	23343	
871	23204	×
955	22394	×
403	22019	
450	21965	
315	21729	
324	21716	×
950	21528	
977	21306	
1027	20917	×
1052	20580	×
506	20537	
562	20373	×
373	20116	
159	19724	×
978	19710	
261	19672	×
827	19575	
231	19532	
810	19485	
22	19441	
163	19395	
599	19338	
27	19334	
801	19271	
812	19270	
176	19024	
179	18980	
976	18820	
171	18645	
29	18323	
18	17963	
1045	17912	×
173	17716	
729	17649	
808	17638	×
64	17445	
736	17359	
130	17181	
103	17062	
170	16930	
202	15663	
772	15653	
771	15578	
16	15383	×
260	14859	× mean+2SD
559	12720	
533	12467	
749	12031	
573	11805	

Compd #	FI (Av.)	
930	11783.5	×
989	11742.5	
142	11655	×
892	11562	×
161	10919.5	×
855	10855	×
602	10733.5	
217	10675	
446	10643.5	
54	10467.5	×
574	10401.5	
832	10024.5	
994	9902	
664	9741	
681	9690.5	
225	9661.5	×
489	9646	
733	9567	
802	9409	×
641	9266.5	
503	9225	
26	9187.5	
965	9155.5	
302	9114.5	
177	9010	
380	8782	
582	8685.5	
860	8581	
293	8535.5	
397	8475	
478	8436.5	
610	8221	
358	8176	
501	8089.5	
42	7940.5	
835	7842	
564	7783	
175	7768.5	
563	7707.5	
964	7602	
526	7571	
423	7470.5	
1007	7347	
944	7334.5	
402	7325.5	
968	7279	
928	7159.5	Q3+1.5IQR

b

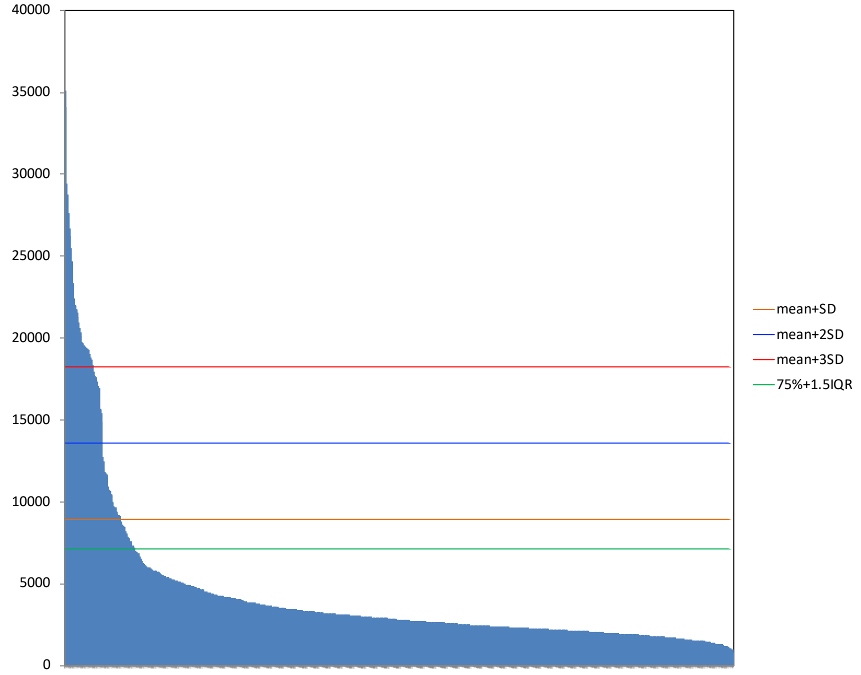

mean	mean+2SD
4264	13579
SD	Q3+1.5IQR
4658	7120
Q3 (75%)	
4136	
Q2 (50%)	
2796	
Q1 (25%)	
2150	
IQR	
1986	

Supporting Table 1-4 1st screening results of P450 BM3(F87A) substrates (Mean of 2 replicates). (a) Compounds that promote the generation of $NADP^+$ are listed in descenting order of the fluorescent signal intensity of $NADP^+$. Compounds having value above Q3 +1.5 IQR are shown. Compounds marked in yellow are hit compounds. X-marks (×) indicate positives for all assessor P450s. Compounds of red font color are good substrates for P450 BM3 (WT). (b) Bar graph representation of all data in descending order of the fluorescent signal intensity.

a

Compd #	FI (Av.)	
53	45665.5	×
807	37627.5	×
1012	37346.5	×
955	33998.5	×
54	33954	×
930	33515.5	×
42	33416	
554	32386.5	
104	27618	
958	27575	×
835	27574.5	×
830	25788.5	×
895	25201.5	
23	25039	
892	24768.5	×
506	24333.5	
773	23977.5	
423	23674	
749	23545	
688	23477	
590	23198	
950	22581	
562	22390.5	×
1027	22168	×
225	21927	×
324	21431.5	×
16	21431	×
1045	21404	×
801	21168	
489	21088.5	
380	21008.5	×
27	20161.5	
28	20071.5	
871	19931	×
159	19380.5	×
965	19048	
808	18485	×
178	18353.5	×
602	17593	
876	17384.5	×
266	17344	
332	17316.5	
1022	17083.5	×
294	16867	×
510	16797	
928	16759.5	
505	16716	×
211	16553	×
261	16333	×
978	16264	
43	16093.5	
976	15731	
179	15689	
1052	15610.5	×
66	15400	
397	15146.5	mean+2SD
968	14786	×
24	14730.5	
733	14399	
260	14314	×
130	14152	
685	14118	

Compd #	FI (Av.)	
402	14108	×
18	13982	
161	13731	×
446	13492	
735	13339.5	
989	13087	
373	12604.5	
383	12494	
358	12203	
72	12158	
22	11970.5	
55	11919.5	
64	11901.5	
142	11847	×
176	11772	
217	11322	
641	11231.5	
559	11028.5	
855	10882	×
143	10664.5	
802	10641	×
181	10253	
582	10232.5	
700	9828	×
302	9727	×
687	9639	
689	9514	
860	9493.5	
516	9242.5	
476	9204.5	
370	8949	
909	8868	
52	8626	×
526	8600.5	
589	8496	
65	8392.5	×
923	8165.5	×
812	8067	
699	8035	
832	7963	
384	7880	
67	7877.5	
810	7876	
102	7864.5	
653	7756	
19	7737.5	
481	7594	
273	7542	
29	7442.5	
229	7313	
7	7261	
103	7253.5	Q3+1.5IQR

b

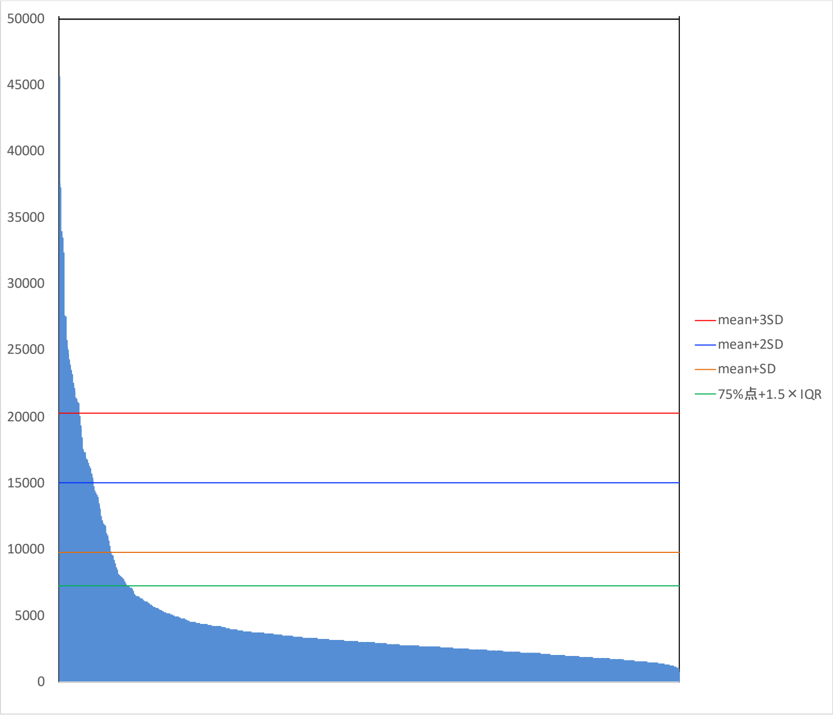

mean	mean+2SD
4525	15026
SD	Q3+1.5IQR
5250	7243
Q3 (75%)	
4230	
Q2 (50%)	
2977	
Q1 (25%)	
2222	
IQR	
2009	

Supporting Table 1-5 1st screening results of P450 BM3(F87A/A330W) substrates (Mean of 2 replicates). (a) Compounds that promote the generation of $NADP^+$ are listed in descenting order of the fluorescent signal intensity of $NADP^+$. Compounds having value above Q3 +1.5 IQR are shown. Compounds marked in yellow are hit compounds. X-marks (×) indicate positives for all assessor P450s. (b) Bar graph representation of all data in descending order of the fluorescent signal intensity.

	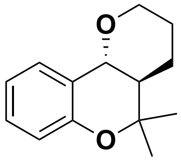	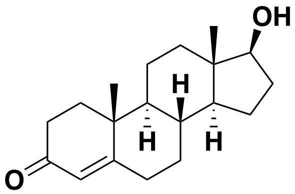	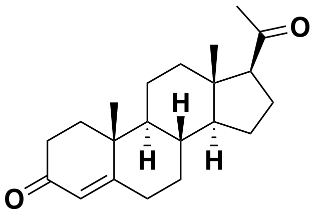
	#104	testosterone	progesterone
Coupling efficiency (%) a)	59.4 ± 2.4	48.5 ± 5.6	9.1 ± 0.6
Conversion 1 (%) b)	31.0 ± 1.8	67.9 ± 3.1	11.6 ± 5.3
Conversion 2 (%) c)	82.9 ± 3.3	36.8 ± 1.4	39.7 ± 8.6
Product formation rate 1 d) (nmol / min / nmol P450)	77.8 ± 18.1	26.3 ± 3.7	0.18 ± 0.04
Product formation rate 2 (s^{-1}) e)	0.512 ± 0.05	0.258 ±0.001	0.0015 ± 0.0001

Supporting Table 2 Oxidation rates, conversions and coupling efficiencies of P450 BM3 F87A/A330W toward compound #104, testosterone, and progesterone

a) Coupling efficiency (%) = produced oxidized product (mol) / consumed NAD(P)H (mol) x 100 (%)
b) Conversion 1 (%) = formed product (mol) / consumed starting material (mol) x 100 (%)
c) Conversion 2 (%) = consumed starting material (mol) / starting material (mol) x 100 (%)
d) Product formation rate 1 (nmol / min / nmol P450)
= rate of product formation (nmol) / min / nmol P450
e) Product formation rate 1 (s^{-1})
= NADPH absorbance decreasing rate at 340 nm (s^{-1}) x coupling efficiency (%) / 100
